# Coordination of LiH Molecules to Mo≣Mo Bonds: Experimental and Computational Studies on Mo_2_LiH_2_, Mo_2_Li_2_H_4_, and Mo_6_Li_9_H_18_ Clusters

**DOI:** 10.1021/jacs.1c01602

**Published:** 2021-03-23

**Authors:** Marina Perez-Jimenez, Natalia Curado, Celia Maya, Jesus Campos, Jesus Jover, Santiago Alvarez, Ernesto Carmona

**Affiliations:** † Instituto de Investigaciones Químicas (IIQ), Departamento de Química Inorgánica and Centro de Innovación en Química Avanzada (ORFEO-CINQA), Consejo Superior de Investigaciones Científicas (CSIC), University of Sevilla, Avda. Américo Vespucio, 49, 41092 Sevilla, Spain; ‡ Department de Química Inorgànica i Orgànica, Secció de Química Inorgànica, and Institut de Química Teòrica i Computacional, Universitat de Barcelona, Martí i Franquès 1-11, 08028 Barcelona, Spain

## Abstract

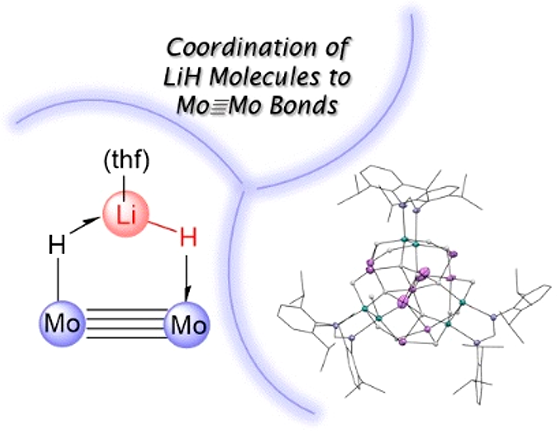

The reactions of LiAlH_4_ as the source of LiH with complexes that contain (H)Mo≣Mo and (H)Mo≣Mo(H) cores stabilized by the coordination of bulky Ad^Dipp2^ ligands result in the respective coordination of one and two molecules of (thf)LiH, with the generation of complexes exhibiting one and two HLi(thf)H ligands extending across the Mo≣Mo bond (Ad^Dipp2^ = HC(NDipp)_2_; Dipp = 2,6-*^i^*Pr_2_C_6_H_3_; thf = tetrahydrofuran, C_4_H_8_O). A theoretical study reveals the formation of Mo–H–Li three-center–two-electron bonds, supplemented by the coordination of the Mo≣Mo bond to the Li ion. Attempts to construct a [Mo_2_{HLi(thf)H}_3_(Ad^Dipp2^)] molecular architecture led to spontaneous trimerization and the formation of a chiral, hydride-rich Mo_6_Li_9_H_18_ supramolecular organization that is robust enough to withstand the substitution of lithium-solvating molecules of tetrahydrofuran by pyridine or 4-dimethylaminopyridine.

## Introduction

Along with noble gas helium, hydrogen and lithium are the simplest, lightest elements and the only ones that existed in the young universe.^1^ Helium hydride, HeH^+^, is a molecule of astrophysical importance,^2^ whereas LiH is the lightest metal hydride and arouses considerable interest due to its many applications.^3−5^ In the gas phase, molecules of LiH exist as a result of the overlap of the singly occupied H 1s and Li 2s atomic orbitals,^6^ with an experimentally determined interatomic distance of *ca*. 1.60 Å.^7^ In the solid state, LiH adopts a cubic NaCl-type structure, characterized by long Li···H contacts of approximately 2.04 Å.^3^

Molecular hydrides of the s-block elements have been intensively investigated in recent years. For group 2 metals, new, uncommon structures and a diversity of useful applications in hydrometalation, hydrogenation, and other reactions have been uncovered, thanks in no small part to the use of sterically encumbered auxiliary ligands.^3,8−19^ Progress for the alkali metals has been more limited, although with notable exceptions. These include Stasch’s hydrocarbon-soluble lithium hydride cluster [{(DippNPPh_2_)Li}_4_(LiH)_4_], containing a (LiH)_4_ central cube (Dipp = 2,6-*^i^*Pr_2_C_6_H_3_),^20^ as well as the generation by Mulvey and co-workers of hexane-soluble lithium hydride transfer reagents.^21,22^ Of particular relevance is the synthesis of the dilithiozincate hydride [(tmed)Li]_2_[{*^i^*PrNCH_2_CH_2_N(*^i^*Pr)}Zn(*^t^*Bu)H] that retains the Li–H bond in solution and undergoes the dynamic association and dissociation of (tmed)LiH.^21^ Also noteworthy are reports on hydride encapsulation by molecular alkali metal clusters,^23^ the structural characterization of the LiH and LiO*^t^*Bu agglomerate Li_33_H_17_(O*^t^*Bu)_16_,^24^ and the synthesis of a (LiH)_4_ cube coordinated to three bis(amido)alane units.^25^

Transition-metal complexes allegedly containing coordinated monomeric molecules of LiH are sparse. There are, however, some reports describing M–H–Li systems where a degree of covalent bonding within the bridging bond can be proposed on the basis of the observation of one-bond ^1^H–^6,7^Li NMR coupling constants.^21,26−35^ Despite the scarcity of complexes of this type, it is conceivable that, like other main-group metal–hydrogen bonds (e.g., Mg–H, Al–H, and Zn–H),^36−44^ a molecule of lithium hydride might bind to a transition-metal fragment through its Li–H bond, assisted by an interaction with an adjoining ligand that could compensate for the unsaturation of the lithium coordination shell and further heighten the σ-donor strength of the polar Li–H bond.

**Figure 1:**
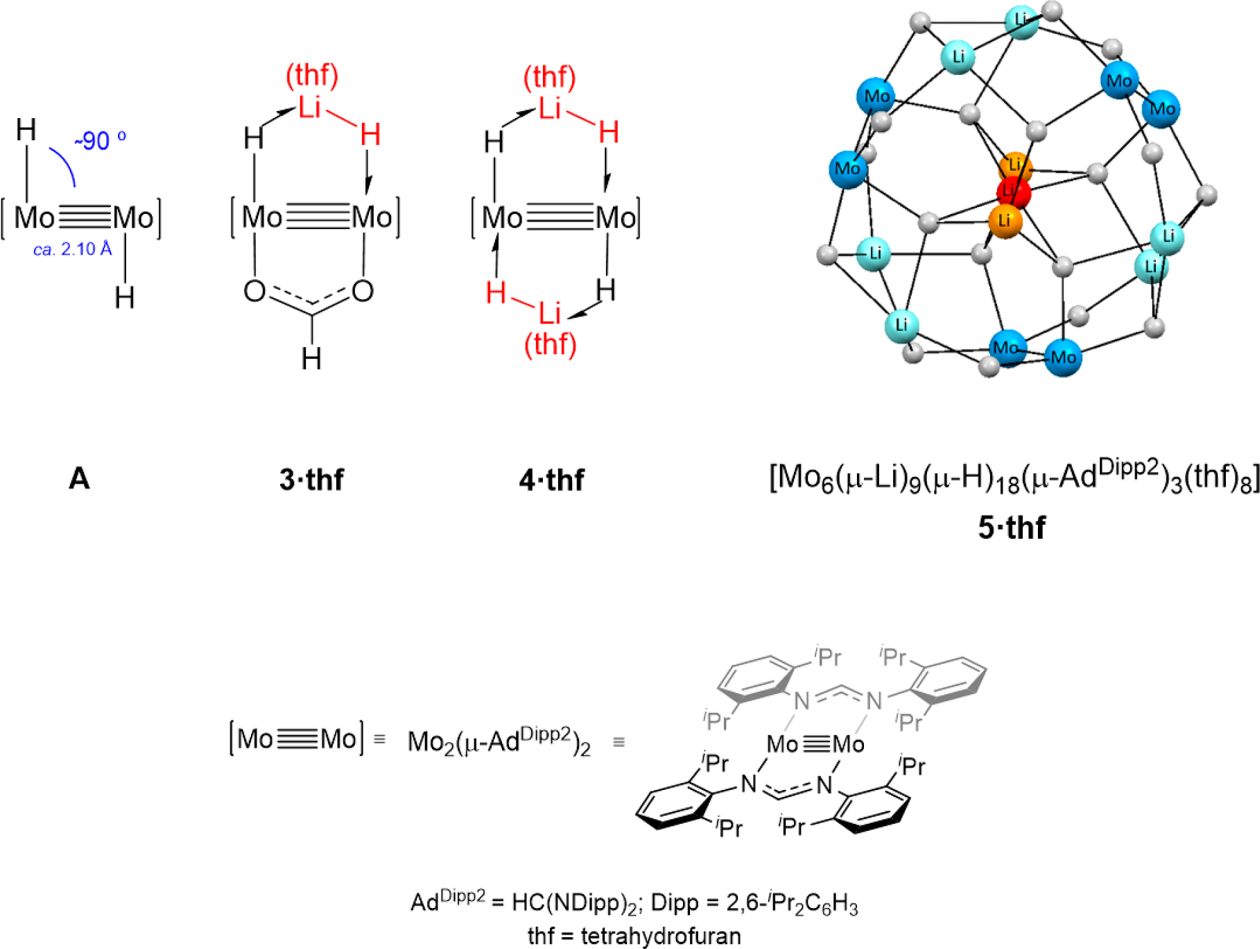
Simplified representations of the structures of complexes **3·thf**-**5·thf**. The three structural types originate from [Mo_2_(H)*_n_*] cores by the incorporation of one, two, or three molecules of (thf)LiH (*n* = 1, complex **3·thf**; *n* = 2, **A** and complex **4·thf**; when *n* = 3, the unobserved monomer trimerizes to complex **5·thf** with the loss of a molecule of tetrahydrofuran). In the structure of **5·thf**, symmetry-related lithium atoms share the same color.

In this context, we envisioned that quadruply bonded hydride central units [Mo_2_(H)_*n*_] (*n* = 1, 2) could be utilized to build the target molecular architectures. As represented in structure **A** of Figure 1, such dimolybdenum dihydride units possess strong hydride character^45^ and feature Mo–Mo separations of close to 2.10 Å, with Mo–H vectors nearly perpendicular to the Mo–Mo bond.^45^ Here, we discuss the synthesis and structure of complexes **3·thf** and **4·thf** (Figure 1) that contain one and two formally monoanionic, bridging H–Li(thf)–H ligands, respectively, spanning the Mo≣Mo bond. We also study the unexpected formation of a unique, hydrocarbon-soluble, hydride-rich Mo_6_Li_9_H_18_ cluster, **5·thf**, formally resulting from the trimerization of unobserved monomer [Mo_2_{μ-HLi(thf)H}_3_(μ-Ad^Dipp2^)], with the loss of a molecule of tetrahydrofuran. Throughout this article, three-center–two-electron (3c–2e) interactions implicating Mo–H and Li–H bonds are represented with the aid of the half-arrow formalism proposed by Green, Green, and Parkin.^46^

## Results and Discussion

**Scheme 1:**
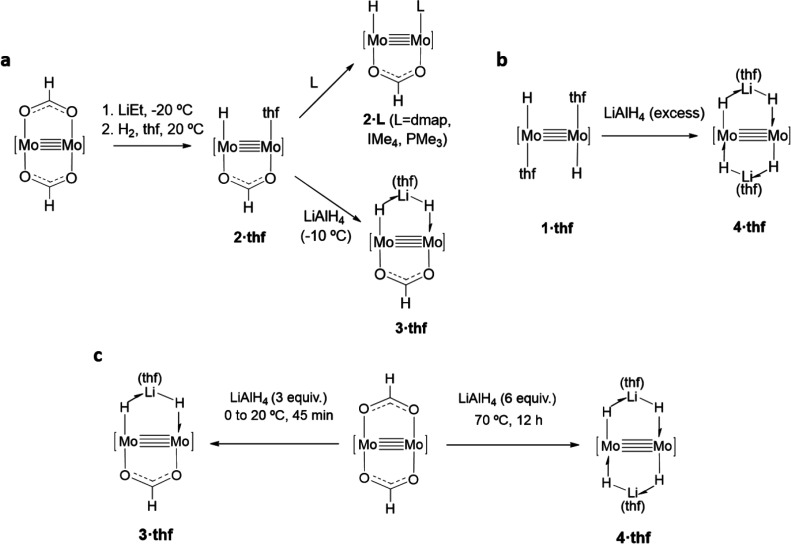
Synthesis of Hydride Complexes with Mo≣Mo Bonds (a) Compounds **2·thf**, **2·L**, and **3·thf**. (b and c) Direct synthesis of complexes **3·thf** and **4·thf**. [Mo≣Mo] is an abbreviation for Mo_2_(μ-Ad^Dipp2^)_2_. L is 4-dimethylaminopyridine (dmap), 1,3,4,5-tetramethylimidazol-2-ylidene, (IMe_4_), and PMe_3_.

In recent work, we showed that tetrahydrofuran adduct [Mo_2_(H)_2_(μ-Ad^Dipp2^)_2_(thf)_2_] (**1·thf**, where Ad^Dipp2^ = HC(NDipp)_2_ and Dipp = 2,6-*^i^*PrC_6_H_3_) is a convenient source of unsaturated dihydride [Mo_2_(H)_2_(μ-Ad^Dipp2^)_2_] containing a *trans*-(H)Mo≣Mo(H) core (structure **A** in Figure 1).^45^ The Mo_2_(H)_2_ functionality of this complex was engendered by hydrogenolysis of the Mo–C bonds of the [(Me)Mo≣Mo(Me)] homologue,^47^ a method that continues to be a main vehicle for the synthesis of transition-metal hydrides. Searching for a related monohydride [(H)Mo≣Mo] core, we carried out the two-step transformation shown in Scheme 1a. Low-temperature alkylation of [Mo_2_(μ-O_2_CH)_2_(μ-Ad^Dipp2^)_2_] with equimolar amounts of LiEt yielded an ethyl-formate intermediate that was reacted *in situ* with H_2_ and converted to the hydride-formate product, **2·thf** (Scheme 1), in good isolated yields (*ca*. 70%). The coordinated tetrahydrofuran molecule of **2·thf** is highly labile, and it was readily replaced by Lewis bases such as 4-dimethylaminopyridine (dmap), 1,3,4,5-tetramethylimidazol-2-ylidene (IMe_4_), and PMe_3_, giving complexes **2·L** (Scheme 1a, top). Similarly, the use of LiAlH_4_ as a source of LiH permitted the isolation of complex **3·thf** that was obtained as a yellow solid in yields of around 60%. This reaction was not, however, simple and also produced related derivative **4·thf**, along with minute amounts of a tetrahydroaluminate complex to be described elsewhere. Complex **3·thf** possesses a H–Mo≣Mo–H–Li(thf) chelate moiety resulting from the substitution of the coordinated tetrahydrofuran of **2·thf** by a molecule of (thf)LiH, with the formation of a σ-Li–H complex, that becomes stabilized by the concomitant formation of a 3c–2e Mo–H⇀Li bond involving the adjacent Mo–H terminus.

Next, **1·thf** was utilized as a source of the [Mo_2_(H)_2_] center (Scheme 1b). Mixing a tetrahydrofuran solution of this complex with a solution of LiAlH_4_ in the same solvent caused the immediate precipitation of a bright-yellow solid that was identified as dilithium tetrahydride dimolybdenum complex [Mo_2_{μ-HLi(thf)H}_2_(μ-Ad^Dipp2^)_2_] (**4·thf**), that is, as a Mo_2_Li_2_H_4_ cluster. As drawn in Scheme 1b, the compound contains two *trans*-[μ-HLi(thf)H] ligands that extend across the Mo≣Mo bond. Thus, it can be related to **3·thf** by means of formal replacement of the bridging formate of the latter by a second μ-Li(thf)H_2_^–^ three-atom chelating ligand. In agreement with this rationale, complexes **3·thf** and **4·thf** were generated in high yields (70–85%) by the more direct method summarized in Scheme 1c, based on the reaction of readily available [Mo_2_(μ-O_2_CH)_2_(μ-Ad^Dipp2^)_2_] with LiAlH_4_, under appropriate conditions.

**Figure 2:**
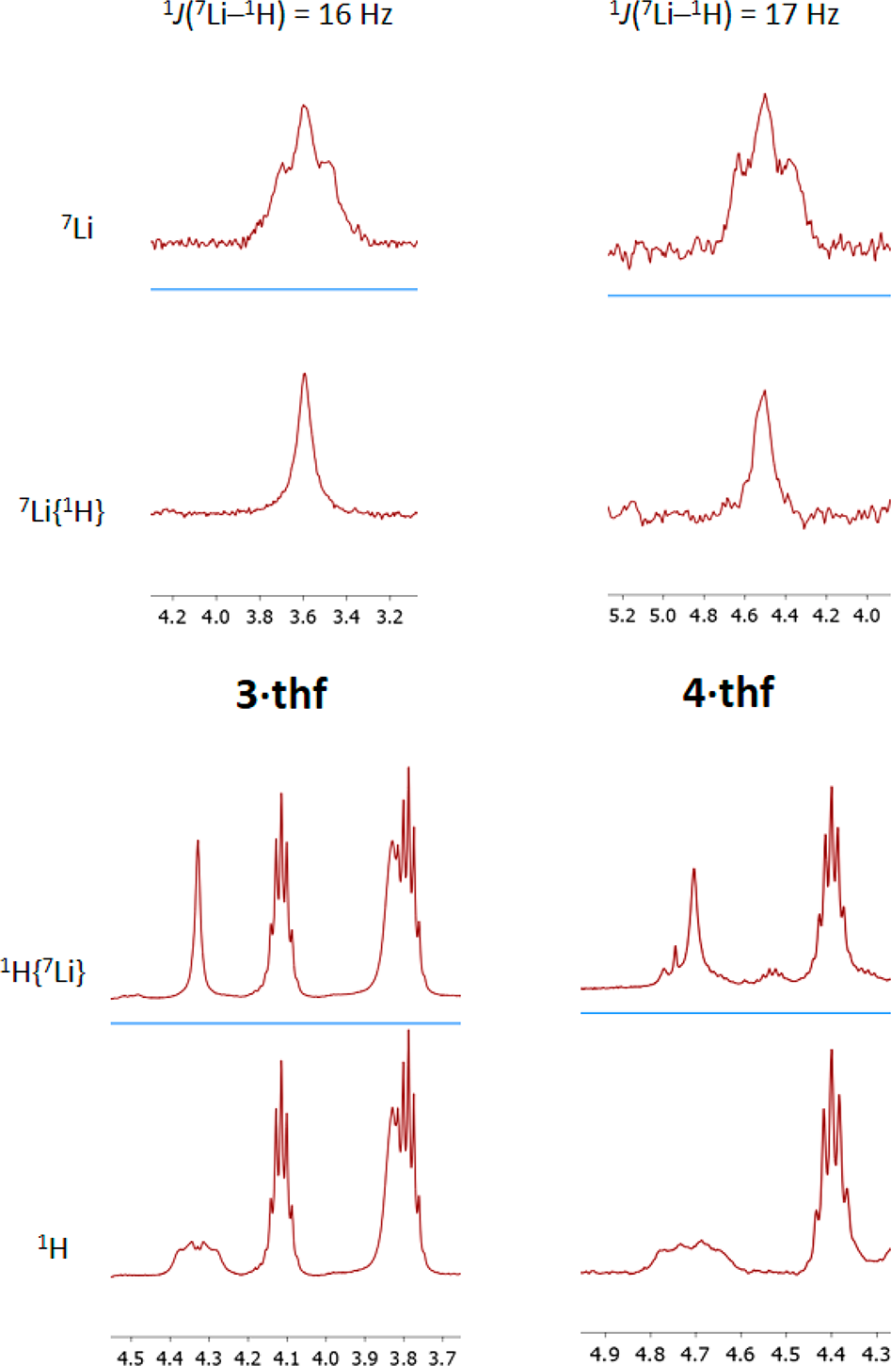
From bottom to top, ^1^H, ^1^H{^7^Li}, ^7^Li{^1^H}, and ^7^Li NMR spectra of the HLiH moieties of complexes **3·thf** (left) and **4·thf** (right). ^1^H resonances at lower frequency relative to Mo–H–Li are due to methine protons of the Ad^Dipp2^ ligands or to tetrahydrofuran.

Complexes **2·L**, **3·thf**, and **4·thf** were characterized with the aid of microanalytical, spectroscopic, and X-ray data and were additionally studied by computational methods. For molecules of **2·L**, the proposed structure is based on IR and NMR data and was unmistakably confirmed by X-ray crystallography for **2·IMe**_**4**_ (Figure S1). Regarding complexes **3·thf** and **4·thf**, their hydride signals were not readily apparent in the IR spectra, possibly because of the Mo–H–Li bridging character, so the unambiguous identification of the three-atom HLiH chains in **3·thf** and **4·thf** owes much to the ^1^H and ^7^Li NMR experiments developed. Surprisingly more soluble in benzene and toluene than in tetrahydrofuran, the H atoms of the HLi(thf)H ligand in **3·thf** resonate at δ 4.33 (C_6_D_6_), appearing as a partially resolved multiplet due to coupling to the ^7^Li (92.6%, *I* = 3/2) and ^6^Li (7.4%, *I* = 1) nuclei. As can be seen in Figure 2, this signal becomes a singlet in the ^1^H{^7^Li} NMR spectrum. Moreover, the 4.33 multiplet is absent in the ^1^H NMR spectrum of the DLiD isotopologue of **3·thf**, prepared by the reaction of [Mo_2_(μ-O_2_CH)_2_(μ-Ad^Dipp2^)_2_]^48^ with LiAlD_4_. The ^7^Li{^1^H} NMR spectrum is a somewhat broad singlet at 3.6 ppm that transforms into a 1:2:1 triplet in the proton-coupled ^7^Li NMR experiment, with a one-bond ^7^Li–^1^H coupling constant of 16 Hz.

Complex **4·thf** is only scarcely soluble in common solvents such as benzene, toluene, and tetrahydrofuran, but it is just sufficiently soluble in C_6_H_5_F for NMR studies. Pertinent NMR data are also included in Figure 2. In particular, the Mo_2_LiH_2_ moieties exhibit comparable ^1^*J*(^7^Li–^1^H) couplings of 17 Hz. These observations categorically demonstrate the existence of HLiH entities coordinated to the Mo–Mo quadruple bond in the **3·thf** and **4·thf** molecules. Besides, they attest without a doubt to the fact that, although probably mainly Coulombic in nature (*vide infra*), the Mo–H–Li–H–Mo bonding interactions involve a considerable degree of covalency, that is, of substantial electron density shared among the molybdenum, hydrogen, and lithium valence orbitals. It is pertinent to remark that the observation of scalar coupling in lithium hydride complexes is rare, to the point that few ^1^*J*(^6,7^Li–^1^H) values can be found in the literature.^21,29−35^ Previously observed couplings range from approximately 6 to 15 Hz such that the 16 to 17 Hz values found for **3·thf** and **4·thf** are among the highest thus far reported. For the LiH molecule, a ^1^*J*(^7^Li–^1^H) coupling constant of 159 Hz has been calculated.^49^

**Figure 3:**
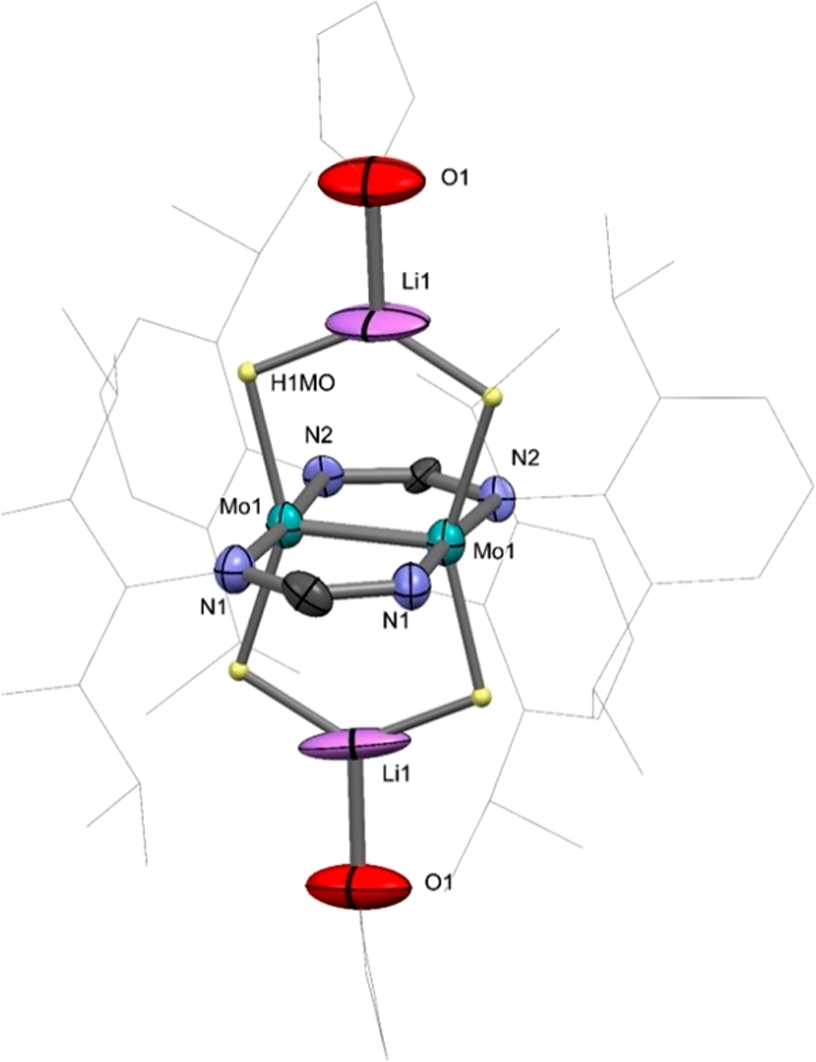
Solid-state structure of **4·thf**. Some atoms have been omitted for clarity. Thermal ellipsoids are shown at 50%. Selected bond distances (Å) and a bond angle (deg): Mo1–Li1, 2.91(2) and 2.97(2); Mo1–Mo1, 2.1006(7); Mo1–N1, 2.10(1); Mo1–N2, 2.20(1); Li1–O1, 1.86(2) Å; and N1–Mo1–Li1, 92.1(5).

Complexes **3·thf** and **4·thf** possess good thermal stability. Their C_6_D_6_ and C_6_D_5_F solutions appear to be stable for 1 day at room temperature, though decomposition occurs upon heating at 70 °C for 3 to 4 h. In the solid state, decomposition is apparent only at *T* ≥ 150 °C. The two compounds behave as soluble LiH carriers, particularly **4·thf**, which is the more reactive of the two. For instance, complex **4·thf** reacted with Ph_2_C(O) to give the expected alkoxide Ph_2_C(H)(OLi).^20,22^ Their molecular structures were investigated by X-ray crystallography and optimized by means of DFT calculations. Owing to poor crystal properties, the data collected for the former do not permit a rigorous structural discussion, particularly with respect to what concerns the geometric parameters of H atoms. Nonetheless, they allow us to define beyond any doubt the connectivity represented in Figure S2. Figure 3 contains an ORTEP representation of the molecules of **4·thf**, along with selected metrics. A more complete set of bond distances is collected in Table 1, which contains both experimental and computational data. When this manuscript was being prepared, there was no precedent for a structural motif of this kind in the Cambridge Structural Database (CSD).^50^ The two bridging H–Li(thf)–H and Ad^Dipp2^ ligands of complex **4·thf** occupy mutually trans positions, originating a typical paddle-wheel structure^51−53^ around a Mo–Mo quadruple bond of length 2.1006(7) Å. The discrepancy observed between the experimental and calculated Mo–H and Li–H distances collected in Table 1 is most likely due to the incertitude in the localization by X-ray diffraction of hydride ligands bound to a heavy atom such as molybdenum. The computed distances are 1.85 and 1.78 Å, respectively. The first is almost coincident with the average Mo–H–Mo bond lengths determined by neutron diffraction,^54^ while the second is somewhat longer than the 1.60 Å value measured for the molecule of LiH in the gas phase but significantly shorter than the interatomic separation of 2.04 Å found for this hydride in the solid state.^3^ Regarding the Mo–Li distances, the experimental values of 2.91(2) and 2.97(2) Å are indistinguishable within experimental error, whereas in the optimized structure this slight asymmetry vanishes, leading to a separation of *ca*. 2.97 Å. For comparison, the sum of the covalent radii of the atoms is 2.82 Å.^55^

**Table 1:** Selected Experimental and Computational Bond Distances (Å) for Complexes **4·thf** and **5·thf**^a^

	**4·thf**		**5·thf**	
	calcd	exp	calcd	exp
Mo–Mo	2.134	2.1006(7)	2.14–2.15	2.10 (av.)
Mo–H	1.852	2.05	1.83–1.84 (Mo–H^cent^)	1.67–2.04
Mo–Li	2.971	2.97(2)	3.21–3.25 (Mo–Li9)	3.15–3.24
	2.968	2.91(2)		
Li–H	1.787	1.74	1.97–2.07 (Li9–H^cent^)	1.81–2.09
	1.784	1.85		

a For the latter complex, the Li7/8–Li9 distances are 2.44 and 2.46 (calcd), 2.45 and 2.50 Å (exp), while corresponding values for the Li7–Li9–Li8 angle are 176.5 and 176.3°.

**Figure 4:**
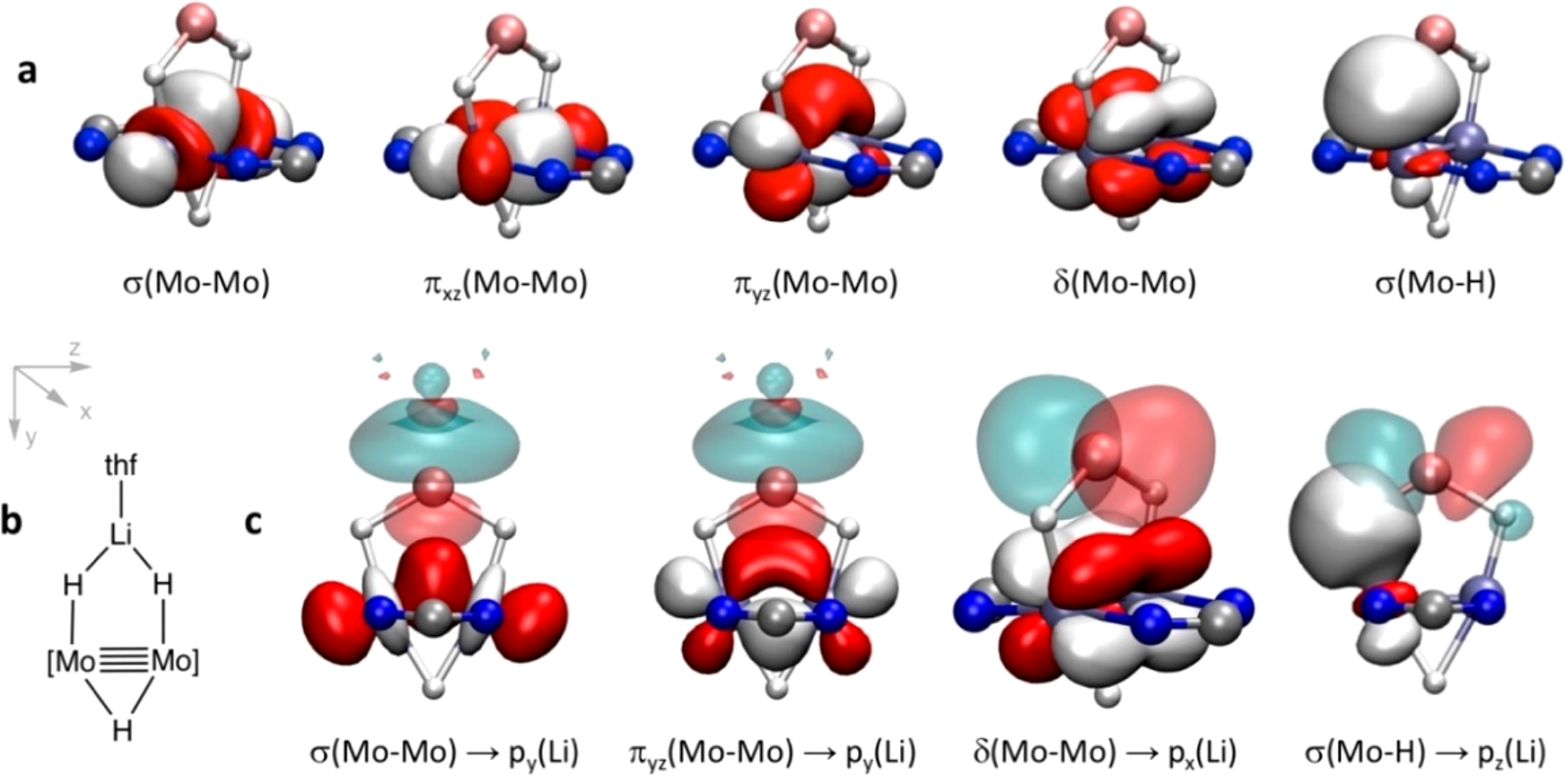
(a) Four NBO orbitals corresponding to the σ component, two π components, and one δ component of the quadruple Mo≣Mo bond in **4·thf′** and one of the Mo–H σ bonding orbitals composed by the δ(Mo–Mo)-type *x*^2^–*y*^2^ and the hydride 1s orbitals. (b) Coordinate orientation and composition of the central fragment of the molecule shown in the orbital plots. (c) Some representative interactions between donor (white and red) and acceptor (light blue and pink) natural orbitals in **4·thf′**.

We have carried out geometry optimization and an NBO analysis of chemical bonding within the Mo–H–Li–H–Mo rings of **4·thf**. For simplicity, we describe here the comparable results obtained for monolithiated species **4·thf′**, whose structure (Figure 4b) finds precedent in that of methyl complex analog [Mo_2_{μ-MeLi(thf)Me}(μ-Me)(μ-Ad^Dipp2^)_2_].^47,56^ The energy for the dissociation of **4·thf′** to (thf)Li–H and dihydride [Mo_2_(H)_2_(μ-Ad^Dipp2^)_2_] given by our calculations is 27.9 kcal/mol, while the dissociation of two molecules of (thf)Li–H from **4·thf** is 55.1 kcal/mol. The NBO analysis discloses four orbitals that are responsible for the σ component, two π components, and one δ component of the quadruple Mo–Mo bond (Figure 4a). In addition, we find that the d*x*^2^–*y*^2^ orbitals, not involved in Mo–Mo bonding, form spd hybrids directed toward the hydrides^47^ and combine with s(H) orbitals to form the two Mo–H bonds (one of which is shown in Figure 4a).

The NBO approach results in limited participation of the lithium atomic orbitals in occupied MOs. However, this does not mean that its interactions with the hydrides and the molybdenum atoms are strictly ionic, since the calculated charge on Li is +0.67, indicative of a non-negligible covalent contribution. The reduced charge of the lithium “ion” is thus associated, in addition to thf → Li donation, with two sets of donor–acceptor interactions: (i) donation from σ(Mo–H) to Li and (ii) donation from the components of the Mo≣Mo bond to Li (Figure 4c).

**Figure 5:**
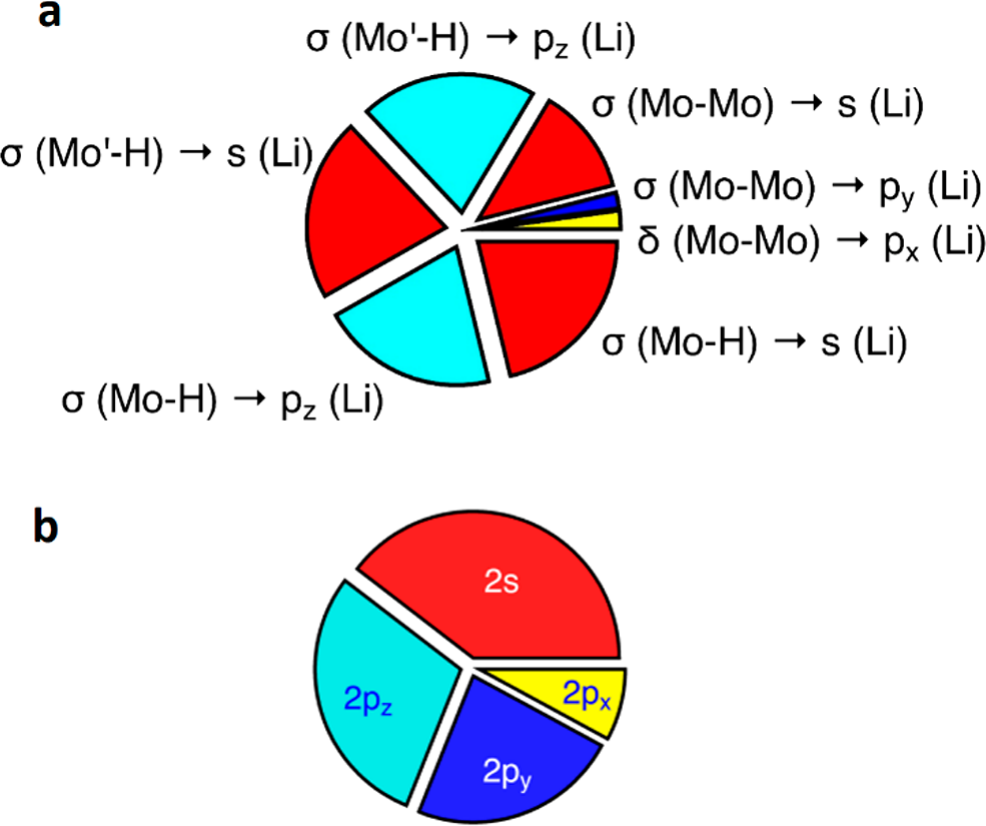
(a) Relative energy contributions of natural orbital donor–acceptor interactions between [Mo_2_(H)_2_(μ-H)(μ-Ad^Dipp2^)_2_]^−^ and (thf)Li^+^ fragments in **4·thf′**. (b) Share of the Li valence electron density at each of its atomic orbitals, resulting from σ(Mo–H) → Li, Mo≣Mo → Li, and thf → Li donor–acceptor interactions.

From the energy point of view, there are two sets of dominant interactions (Figure 5a) that imply donations from the σ(Mo–H) and σ(Mo’–H) bonds to both s(Li) and p_*z*_(Li) and from the σ component of the Mo≣Mo bond to the atomic orbitals of Li. In the first set, we find donation from σ(Mo–H) to both s(Li) and p_*z*_(Li), which are responsible for 84% of the interaction energy. Among the second set of interactions, donation from σ(Mo–Mo) to s(Li) (Figure 4c) makes a significant contribution of 12%; smaller contributions come from the donations of δ(Mo–Mo) to p_*x*_(Li) and of σ(Mo–Mo) to p_*y*_(Li), while almost negligible contributions appear for π(Mo–Mo) and for p_*y*_ and s(Li).

As a result of all of these donor–acceptor interactions from the Mo_2_H_2_ moiety to the lithium ion, the distribution of the 0.33 valence electron held by the Li atomic orbitals (Figure 5b) reflects the major role played by the 2s and 2p_*z*_ Li AOs as acceptors. The high population of the lithium p_*y*_ orbital compared to its minor acceptor role toward the Mo≣Mo group is undoubtedly due to the donation from its thf ligand. Finally, the lowest atomic orbital population in p_*x*_ results from the interesting donation from the δ(Mo≣Mo) bonding orbital (Figure 4c).

We can therefore conclude that the stability of the Mo–H–Li–H–Mo ring results mainly from the formation of two 3c–2e Mo–H–Li bonds, supplemented by σ coordination of the Mo≣Mo bond to the Li atom. The latter bonding component is consistent with a short distance between Li and the Mo≣Mo centroid of 2.77 Å (Mo–Li = 2.97 Å), to be compared with a covalent radii sum of 2.82 Å.^55^ Although of lesser quantitative importance, the existence of non-negligible electron donation from the bonding π and δ(Mo≣Mo) orbitals is worth being stressed. The fact that the calculated dissociation energy of **4·thf′** into (thf)Li–H and dihydride [Mo_2_(H)_2_(μ-Ad^Dipp2^)_2_] is 27.9 kcal/mol, smaller than the sum of NBO interaction energies shown in Figure 5a (98.7 kcal/mol), is explained by the high energy required to deform the (thf)Li–H group from linear in the free molecule to a highly bent (120°) geometry in **4·thf′** as well as to modify the second coordination sphere of the Mo atoms to make room for the Li–thf moiety.

**Scheme 2:**
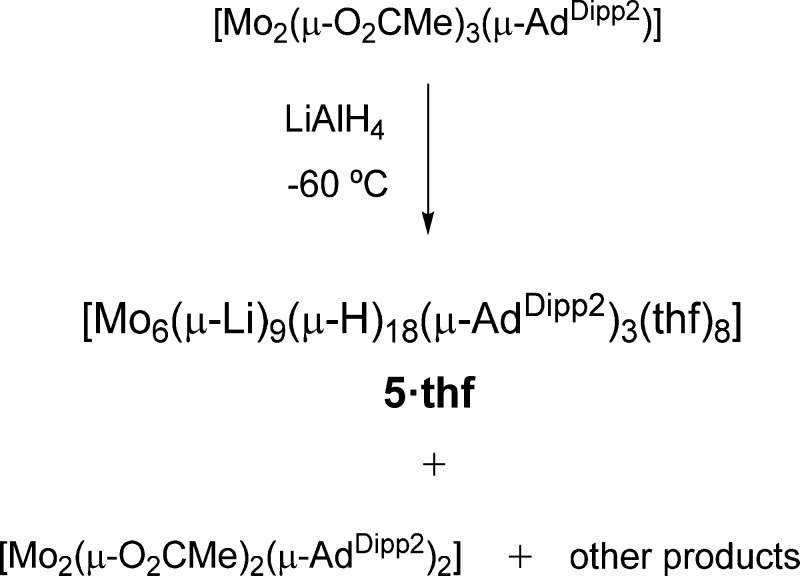
Formation of Hexamolybdenum Nonalithium Dodecaoctahydride Cluster **5·thf**

Having successfully built Mo_2_LiH_2_ and Mo_2_Li_2_H_4_ platforms based on Mo≣Mo bonds coordinated to one and two H–Li(thf)–H units, respectively, our next goal was to explore the possibility of reaching a Mo_2_Li_3_H_6_ organization in a purported [Mo_2_{HLi(thf)H}_3_(μ-Ad^Dipp2^)] complex. To this end and taking into account the successful synthesis of complexes **3·thf** and **4·thf** by the procedure shown in Scheme 1c, we prepared tris(acetate) precursor [Mo_2_(μ-O_2_CMe)_3_(μ-Ad^Dipp2^)] and performed its reaction with an excess of LiAlH_4_. Although the above Mo_2_Li_3_H_6_ complex could not be observed, the transformation led to complex **5·thf**, identified as a polymetallic hydride cluster Mo_6_Li_9_H_18_ (Scheme 2), that probably results from spontaneous trimerization of the targeted Mo_2_Li_3_H_6_ monomer, with the loss of a molecule of tetrahydrofuran. The reaction was, however, complex and gave in addition compound [Mo_2_(μ-O_2_CMe)_2_(μ-Ad^Dipp2^)_2_] through an undisclosed reaction path. Like the bis(formate) analogue (Scheme 1c), the latter may react further with LiAlH_4_, justifying that isolated yields of **5·thf** are about 25%. Complex **5·thf** is very air-sensitive and decomposes instantly in the presence of oxygen and water, both in solution and in the solid state. Under strict anaerobic conditions, solutions in tetrahydrofuran or aromatic hydrocarbons remain unchanged at 25 °C for at least 24 h, although decomposition is fast above 50 °C.

The new supramolecular entity can be understood as a triangular array of [Mo_2_(μ-Ad^Dipp2^)]^3+^ components^51,57,58^ connected by a [Li_9_H_18_]^9–^ linker in a fairly robust manner. The Li-coordinated molecules of tetrahydrofuran were readily substituted by pyridine and 4-dimethylaminopyridine, giving complexes **5·py** and **5·dmap** without the alteration of the molecular skeleton. Notwithstanding the foregoing, complex **5·thf** acted as an efficient source of LiH in the hydrolithiation of Ph_2_C(O) to give Ph_2_C(H)(OLi).^20,22^ Somewhat unexpectedly, solutions of **5·thf** decomposed gradually upon stirring at room temperature under an atmosphere of H_2_, generating LiAd^Dipp2^ as a byproduct. Dideuterium acted similarly and showed that H/D exchange took place, as attested to by NMR detection of HD along with H_2_. The H_2_-promoted cluster breakup was not investigated any further. Nevertheless, it seems plausible that H_2_ may disrupt the cluster structure by displacing LiH molecules from the [Li_9_H_18_]^9–^ linker, eliminating LiAd^Dipp2^. As an extension of these studies, various attempts were made to produce an alleged {Mo_2_(H)_8_[Li(thf)]_4_} complex (i.e., the hydride analogue of known methyl compound {Mo_2_(CH_3_)_8_[Li(OEt_2_)]_4_}).^59^ As detailed in the SI, all essayed trials were unsuccessful.

The room-temperature ^1^H NMR spectra of complexes **5·L** in C_6_D_6_ or thf-*d*_8_ solution show two septets and four doublets for the 12 isopropyl groups of the amidinate spectator ligands, in accordance with the proposed *D*_3_ molecular symmetry (details in Supporting Information). The 18 H atoms that make up the [Li_9_H_18_] linker are expected to give rise to three resonances of equal relative intensity. Whereas for **5·thf** one of these signals seems to be hidden underneath other resonances, the three are clearly observed for complex **5·py** with chemical shifts of 2.04, 5.21, and 5.41 ppm. They appear as broad multiplets, but while the 2.04 peak becomes a singlet in the ^1^H{^7^Li} NMR spectrum, the other two are converted to doublets with ^2^*J*_HH_ = 4 Hz. The ^7^Li NMR spectrum contains three resonances centered at 5.4, 4.7, and 2.7 ppm, with relative intensities approaching roughly 6:2:1, once more in agreement with the proposed structure.

**Figure 6:**
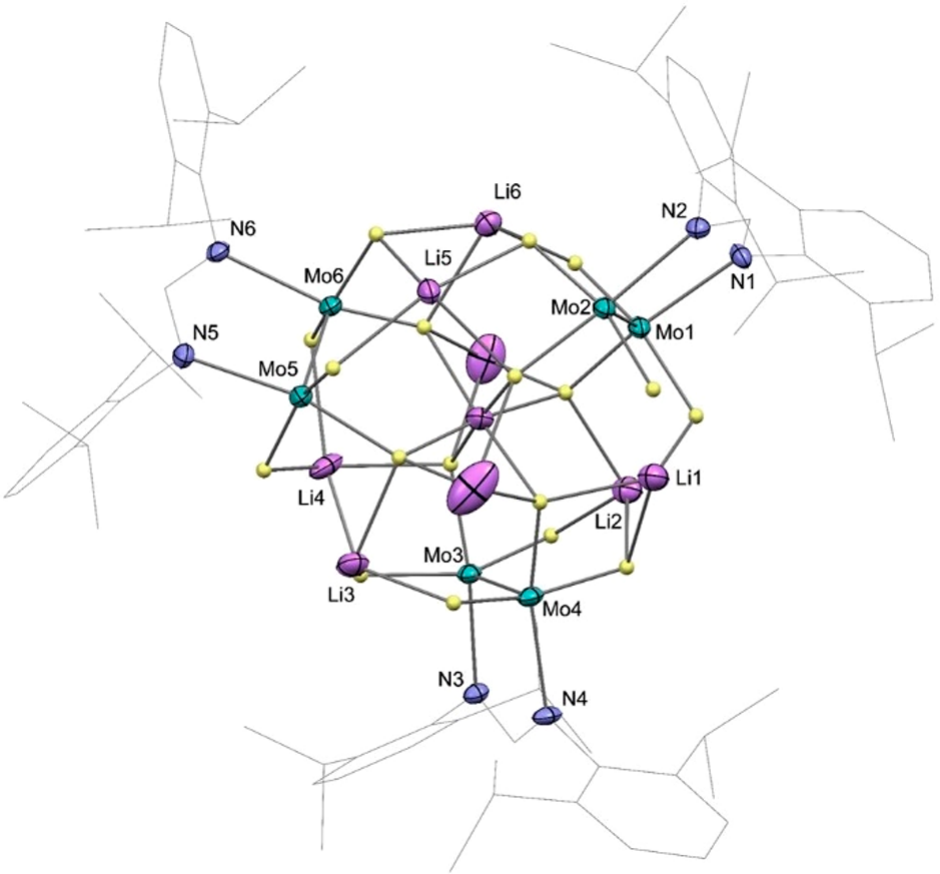
Solid-state structure of **5·thf** as determined by X-ray diffraction. Thermal ellipsoids are shown at 30%. Hydrogen atoms (except the hydride ligands) are omitted for clarity, as are thf molecules. Selected bond lengths (Å) and a bond angle (deg): Mo–Mo, 2.10 av.; Mo–Li9, 3.20 av.; Mo–N, 2.13 av.; Li9–Li7, 2.45(2); Li9–Li8, 2.50(2); Li7–Li9–Li8, 176.3(9).

**Figure 7:**
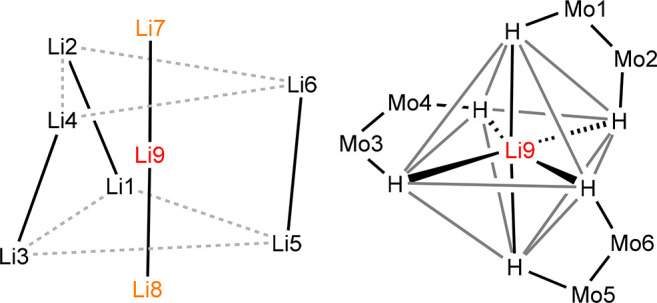
Li_9_ polyhedron present in the molecules of complex **5·thf** (left) and the distribution of H and Li atoms in the vicinity of central lithium atom Li9 (right).

The molecular structure of complex **5·thf** was determined by X-ray crystallography (Figure 6) and computational studies. Since the calculated and experimental structures are very similar (Table 1), all of the features that are discussed here based on the X-ray data also apply to the optimized geometry. The whole cluster is built up by successive concentric groups around a central Li_3_ unit (Figure 7, left) formed by Li7, Li9, and Li8 with a nearly linear arrangement (176(1)°) and distances of 2.50(2) and 2.45(2) Å, which are slightly shorter than twice the lithium covalent radius (2.56 Å).^55^ We have been unable to locate a solid-state or gas-phase structure in which such a Li_3_ rod is present. The only Li_3_ group whose structure we are aware of appears in the crystal structure of Li_3_[IrD_6_], with Li–Li distances of 2.58 and 2.76 Å and a Li–Li–Li angle of 75.7°.^60^ The first concentric group around the central axis is composed of six H^cent^ atoms that provide a nearly octahedral coordination sphere to the central Li9 atom (Figure 7, right) and act as bridging atoms with the terminal atoms of the Li_3_ rod, with Li–H separations in the 1.70–2.20 Å interval. These hydrides are connected to the molybdenum atoms of the three Mo_2_ units that constitute the second concentric ring, with the shape of a slightly twisted trigonal prism and Mo–H distances in the range of 1.67–2.05 Å. The Mo–Mo bond length of 2.1020(7) Å is coherent with 4-fold bonding.^51^

Leaving aside the Li atoms, an ionic description of the cluster leaves us with three [Mo_2_(μ-Ad^Dipp2^)H_6_]^3–^ blocks, in which each Mo atom bears two cis hydrides and one trans hydride relative to the N atoms of the μ-Ad^Dipp2^ ligand. The latter have just been described as forming an H_6_ octahedron around the inner Li_3_ rod and being bonded to the three Mo_2_ units as well. The 12 cis hydrides can be described as distorted trigonal prisms, one with the trigonal faces roughly at the height of the external atoms of the central Li_3_ rod, or H_6_^ext^ group, and the other with the trigonal faces very close to the central Li9 atom, or H_6_^int^. Finally, the six peripheral Li atoms form another trigonal prism (Figure 7, left) with one of the triangular faces (Li1, Li3, and Li5) rotated *ca*. 13° relative to the other (Li2, Li4, and Li6). Those Li atoms form three Li_2_ dumbbells with Li···Li distances of 2.83–2.90 Å and are supported by hydride bridges to neighboring Li and Mo atoms, with Li–H separations in the range of 1.74–2.29 Å (section 5 in the Supporting Information).

## Conclusions

We have demonstrated that a monomeric molecule of LiH can bind to the unsaturated molybdenum atom of [(H)Mo≣Mo] entities by means of a 3c–2e Mo–H⇀Li interaction combined with a σ-Li–H⇀Mo bond. [Mo_2_{μ*-*HLi(thf)H}_*n*_] skeletons containing five-membered H–Mo≣Mo–H–Li rings have been constructed in this manner for *n* = 1 and 2. When *n* = 3, trimerization of the purported [Mo_2_{μ-HLi(thf)H}_3_(μ-Ad^Dipp2^)] monomer occurs spontaneously, leading to a hydride-rich Mo_6_Li_9_H_18_ supramolecular organization that features an uncommon linear Li_3_ group around which are organized Mo_6_, Li_6_, and two H_6_ polyhedra with shapes intermediate between an octahedron and compressed trigonal prisms.

## Supporting Information Available

Relevant experimental and calculated bonding parameters for **3·thf**, **4·thf** and **5·thf**; computational details; and atomic coordinates for the optimized geometries of the same compounds; synthesis and characterization of compounds (PDF)
Olex2 1.3 data (PDF)

## Accession Codes

CCDC 2059184–2059187 contain the supplementary crystallographic data for this paper. These data can be obtained free of charge via www.ccdc.cam.ac.uk/data_request/cif, or by emailing data_request@ccdc.cam.ac.uk, or by contacting The Cambridge Crystallographic Data Centre, 12 Union Road, Cambridge CB2 1EZ, UK; fax: +44 1223 336033.

## Dedication

This paper is dedicated to the memory of our esteemed colleague and friend Professor Malcolm L. H. Green, in recognition of his monumental contributions to inorganic chemistry, organometallic chemistry, and catalysis.
