# Supplementary material for: Coordination of LiH Molecules to Mo≣Mo Bonds: Experimental and Computational Studies on Mo2LiH2, Mo2Li2H4, and Mo6Li9H18 Clusters
Source: J Am Chem Soc. 2021 Mar 23;143(13):5222–30. doi: 10.1021/jacs.1c01602 (PMC9157502; doi:10.1021/jacs.1c01602)
Supplement: Supplementary file 1 — ja1c01602_si_005.pdf [file ja1c01602_si_005.pdf]

```
data_12_sqp
_audit_creation_date              2020-11-21
_audit_creation_method
;
Olex2 1.3
(compiled 2020.02.04 svn.rd84adfe8 for OlexSys, GUI svn.r6032)
;
_shelx_SHELXL_version_number      '2014/7'
_audit_contact_author_address     'Av. Americo Vespucio 49, 41092,
Sevilla, Spain'
_audit_contact_author_email       'marina.perez@iiq.csic.es'
_audit_contact_author_name        'Marina Perez-Jimenez'
_audit_contact_author_phone       .
_publ_contact_author_id_orcid     '0000-0001-7891-4273'
_publ_section_references
;
Dolomanov, O.V., Bourhis, L.J., Gildea, R.J, Howard, J.A.K. &
Puschmann, H.
 (2009), J. Appl. Cryst. 42, 339-341.

Sheldrick, G.M. (2008). Acta Cryst. A64, 112-122.
;
_chemical_name_common             .
_chemical_name_systematic         .
_chemical_formula_moiety          'C107 H187 Li9 Mo6 N6 O8'
_chemical_formula_sum             'C107 H187 Li9 Mo6 N6 O8'
_chemical_formula_weight          2323.74
_chemical_melting_point           'not measured'
loop_
  _atom_type_symbol
  _atom_type_description
  _atom_type_scat_dispersion_real
  _atom_type_scat_dispersion_imag
  _atom_type_scat_source
 'C' 'C' 0.0033 0.0016 'International Tables Vol C Tables 4.2.6.8
and 6.1.1.4'
 'H' 'H' 0.0000 0.0000 'International Tables Vol C Tables 4.2.6.8
and 6.1.1.4'
 'Li' 'Li' -0.0003 0.0001
 'International Tables Vol C Tables 4.2.6.8 and 6.1.1.4'
 'Mo' 'Mo' -1.6832 0.6857
 'International Tables Vol C Tables 4.2.6.8 and 6.1.1.4'
 'N' 'N' 0.0061 0.0033 'International Tables Vol C Tables 4.2.6.8
and 6.1.1.4'
 'O' 'O' 0.0106 0.0060 'International Tables Vol C Tables 4.2.6.8
and 6.1.1.4'

_shelx_space_group_comment
;
The symmetry employed for this shelxl refinement is uniquely
defined
by the following loop, which should always be used as a source of
symmetry information in preference to the above space-group names.
They are only intended as comments.
;
_space_group_crystal_system       'triclinic'
_space_group_IT_number            2
_space_group_name_H-M_alt         'P -1'
```

```
_space_group_name_Hall            '-P 1'
loop_
  _space_group_symop_operation_xyz
 'x, y, z'
 '-x, -y, -z'

_cell_length_a                    17.0778(11)
_cell_length_b                    17.0833(11)
_cell_length_c                    25.8714(17)
_cell_angle_alpha                 77.107(3)
_cell_angle_beta                  76.123(3)
_cell_angle_gamma                 60.217(2)
_cell_volume                      6311.1(7)
_cell_formula_units_Z             2
_cell_measurement_reflns_used     9891
_cell_measurement_temperature     193(2)
_cell_measurement_theta_max       28.29
_cell_measurement_theta_min       2.38
_exptl_absorpt_coefficient_mu     0.626
_exptl_absorpt_correction_T_max   0.7457
_exptl_absorpt_correction_T_min   0.6666
_exptl_absorpt_correction_type    multi-scan
_exptl_absorpt_process_details
;
SADABS-2016/2 (Bruker,2016/2) was used for absorption correction.
wR2(int) was 0.1535 before and 0.0794 after correction.
The Ratio of minimum to maximum transmission is 0.8939.
The \l/2 correction factor is Not present.
;
_exptl_absorpt_special_details    .
_exptl_crystal_colour             red
_exptl_crystal_colour_primary     red
_exptl_crystal_density_diffrn     1.223
_exptl_crystal_density_meas       .
_exptl_crystal_density_method     .
_exptl_crystal_description        'prism'
_exptl_crystal_F_000              2428
_exptl_crystal_size_max           0.28
_exptl_crystal_size_mid           0.25
_exptl_crystal_size_min           0.10
_exptl_transmission_factor_max    .
_exptl_transmission_factor_min    .
_diffrn_reflns_av_R_equivalents   0.1573
_diffrn_reflns_av_unetI/netI      0.1242
_diffrn_reflns_Laue_measured_fraction_full  0.999
_diffrn_reflns_Laue_measured_fraction_max  0.997
_diffrn_reflns_limit_h_max        21
_diffrn_reflns_limit_h_min        -22
_diffrn_reflns_limit_k_max        22
_diffrn_reflns_limit_k_min        -22
_diffrn_reflns_limit_l_max        34
_diffrn_reflns_limit_l_min        -34
_diffrn_reflns_number             142142
_diffrn_reflns_point_group_measured_fraction_full  0.999
_diffrn_reflns_point_group_measured_fraction_max  0.997
_diffrn_reflns_theta_full         25.242
_diffrn_reflns_theta_max          28.314
_diffrn_reflns_theta_min          2.007
```

```
_diffrn_ambient_temperature        193.0
_diffrn_detector_area_resol_mean   .
_diffrn_measured_fraction_theta_full  0.999
_diffrn_measured_fraction_theta_max  0.997
_diffrn_measurement_device_type    'Bruker APEX-II CCD'
_diffrn_measurement_method         '\f and \w scans'
_diffrn_radiation_type             MoK\a
_diffrn_radiation_wavelength       0.71073
_diffrn_source                     .
_diffrn_source_current             1.4
_diffrn_source_power               0.07
_diffrn_source_voltage             50.0
_diffrn_standards_number           0
_reflns_Friedel_coverage           0.000
_reflns_Friedel_fraction_full      .
_reflns_Friedel_fraction_max       .
_reflns_number_gt                  18339
_reflns_number_total               31329
_reflns_special_details
;
 Reflections were merged by SHELXL according to the crystal
 class for the calculation of statistics and refinement.

 Structure factors included contributions from the .fab file.

 _reflns_Friedel_fraction is defined as the number of unique
 Friedel pairs measured divided by the number that would be
 possible theoretically, ignoring centric projections and
 systematic absences.
;
_reflns_threshold_expression       'I > 2\s(I)'
_computing_cell_refinement         'SAINT V8.38A (Bruker, 2018)'
_computing_data_collection         'Apex3'
_computing_data_reduction          'SAINT V8.38A (Bruker, 2018)'
_computing_molecular_graphics      'Olex2 1.3 (Dolomanov et al.,
2009)'
_computing_publication_material    'Olex2 1.3 (Dolomanov et al.,
2009)'
_computing_structure_refinement    'XL  (Sheldrick, 2008)'
_computing_structure_solution      'SHELXT 2014/5 (Sheldrick,
2014)'
_refine_diff_density_max           2.108
_refine_diff_density_min           -1.523
_refine_diff_density_rms           0.124
_refine_ls_extinction_coef         .
_refine_ls_extinction_method       none
_refine_ls_goodness_of_fit_ref     1.017
_refine_ls_hydrogen_treatment      mixed
_refine_ls_matrix_type             full
_refine_ls_number_parameters       1303
_refine_ls_number_reflns           31329
_refine_ls_number_restraints       314
_refine_ls_R_factor_all            0.1379
_refine_ls_R_factor_gt             0.0756
_refine_ls_restrained_S_all        1.021
_refine_ls_shift/su_max            0.002
_refine_ls_shift/su_mean           0.000
_refine_ls_structure_factor_coef   Fsqd
```

```
_refine_ls_weighting_details
 'w=1/[\s^2^(Fo^2^)+(0.0686P)^2^+8.6451P] where P=(Fo^2^+2Fc^2^)/3'
_refine_ls_weighting_scheme        calc
_refine_ls_wR_factor_gt            0.1547
_refine_ls_wR_factor_ref           0.1807
_refine_special_details            .

loop_
  _platon_squeeze_void_nr
  _platon_squeeze_void_average_x
  _platon_squeeze_void_average_y
  _platon_squeeze_void_average_z
  _platon_squeeze_void_volume
  _platon_squeeze_void_count_electrons
  _platon_squeeze_void_content
   1  0.066  0.504  0.527        51          3 ' '
   2 -0.073  0.495  0.470        47          3 ' '
   3  0.083  0.917  0.508         6          0 ' '
   4  0.500  0.000  0.000       376         99 ' '
   5  0.917  0.083  0.492         6          0 ' '
_platon_squeeze_void_probe_radius                  1.20
_platon_squeeze_details
;
The unit cell contains 3 molecules of THF which has been
treated as a diffuse contribution to the overall scattering without
specific atom position by SQUEEZE PLATON.
;

_olex2_refinement_description
;
1. Fixed Uiso
 At 1.2 times of:
  All C(H) groups, All C(H,H) groups
 At 1.5 times of:
  All C(H,H,H) groups, All Mo(H,H,H) groups
2. Restrained distances
 C88-C89 = C89-C90 = C90-C91
 1.54 with sigma of 0.02
 C100-C101 = C101-C102 = C102-C103
 1.54 with sigma of 0.02
 C96-C97 = C97-C98 = C98-C99
 1.54 with sigma of 0.02
 C92-C93 = C93-C94 = C94-C95
 1.54 with sigma of 0.02
 C104-C105 = C105-C106 = C106-C107
 1.54 with sigma of 0.02
 C76-C77 = C77-C78 = C78-C79
 1.54 with sigma of 0.02
 C84-C85 = C85-C86 = C86-C87
 1.54 with sigma of 0.02
 C80-C81 = C81-C82 = C82-C83
 1.54 with sigma of 0.02
3. Rigid bond restraints
 O4, C88, C89, C90, C91
 with sigma for 1-2 distances of 0.01 and sigma for 1-3 distances
of 0.01
 O5, C92, C93, C94, C95
```

with sigma for 1-2 distances of 0.01 and sigma for 1-3 distances of 0.01
O8, C104, C105, C106, C107
with sigma for 1-2 distances of 0.01 and sigma for 1-3 distances of 0.01
O1, C76, C77, C78, C79
with sigma for 1-2 distances of 0.01 and sigma for 1-3 distances of 0.01
O2, C80, C81, C82, C83
with sigma for 1-2 distances of 0.01 and sigma for 1-3 distances of 0.01
4. Uiso/Uaniso restraints and constraints
O4 \\sim C88 \\sim C89 \\sim C90 \\sim C91: within 2A with sigma of 0.04 and
sigma for terminal atoms of 0.08 within 2A
O7 \\sim C100 \\sim C101 \\sim C102 \\sim C103: within 2A with sigma of 0.00001
and sigma for terminal atoms of 0.00002 within 2A
O6 \\sim C96 \\sim C97 \\sim C98 \\sim C99: within 2A with sigma of 0.00001 and
sigma for terminal atoms of 0.00002 within 2A
O5 \\sim C92 \\sim C93 \\sim C94 \\sim C95: within 2A with sigma of 0.04 and
sigma for terminal atoms of 0.08 within 2A
O8 \\sim C104 \\sim C105 \\sim C106 \\sim C107: within 2A with sigma of 0.04
and sigma for terminal atoms of 0.08 within 2A
O1 \\sim C76 \\sim C77 \\sim C78 \\sim C79: within 2A with sigma of 0.04 and
sigma for terminal atoms of 0.08 within 2A
O3 \\sim C84 \\sim C85 \\sim C86 \\sim C87: within 2A with sigma of 0.00001 and
sigma for terminal atoms of 0.00002 within 2A
O2 \\sim C80 \\sim C81 \\sim C82 \\sim C83: within 2A with sigma of 0.04 and
sigma for terminal atoms of 0.08 within 2A
5.a Ternary CH refined with riding coordinates:
C8(H8), C11(H11), C20(H20), C23(H23), C33(H33), C36(H36), C45(H45), C48(H48),
C58(H58), C61(H61), C70(H70), C73(H73)
5.b Secondary CH2 refined with riding coordinates:
C76(H76A,H76B), C77(H77A,H77B), C78(H78A,H78B), C79(H79A,H79B), C80(H80A,
H80B), C81(H81A,H81B), C82(H82A,H82B), C83(H83A,H83B), C84(H84A,H84B),
C85(H85A,H85B), C86(H86A,H86B), C87(H87A,H87B), C88(H88A,H88B), C89(H89A,H89B),
C90(H90A,H90B), C91(H91A,H91B), C92(H92A,H92B), C93(H93A,H93B), C94(H94A,
H94B), C95(H95A,H95B), C96(H96A,H96B), C97(H97A,H97B), C98(H98A,H98B),
C99(H99A,H99B), C100(H10D,H10E), C101(H10F,H10G), C102(H10H,H10I), C103(H10J,
H10K), C104(H10L,H10M), C107(H10N,H10O), C105(H10P,H10Q), C106(H10R,H10S)
5.c Aromatic/amide H refined with riding coordinates:
C1(H1), C4(H4), C5(H5), C6(H6), C16(H16), C17(H17), C18(H18), C26(H26),

```
 C29(H29), C30(H30), C31(H31), C41(H41), C42(H42), C43(H43),
C51(H51), C54(H54),
  C55(H55), C56(H56), C66(H66), C67(H67), C68(H68)
5.d Idealised Me refined as rotating group:
 C9(H9A,H9B,H9C), C10(H10A,H10B,H10C), C12(H12A,H12B,H12C),
C13(H13A,H13B,
 H13C), C21(H21A,H21B,H21C), C22(H22A,H22B,H22C),
C24(H24A,H24B,H24C), C25(H25A,
 H25B,H25C), C34(H34A,H34B,H34C), C35(H35A,H35B,H35C),
C37(H37A,H37B,H37C),
 C38(H38A,H38B,H38C), C46(H46A,H46B,H46C), C47(H47A,H47B,H47C),
C49(H49A,H49B,
 H49C), C50(H50A,H50B,H50C), C59(H59A,H59B,H59C),
C60(H60A,H60B,H60C), C62(H62A,
 H62B,H62C), C63(H63A,H63B,H63C), C71(H71A,H71B,H71C),
C72(H72A,H72B,H72C),
 C74(H74A,H74B,H74C), C75(H75A,H75B,H75C)
;
_atom_sites_solution_hydrogens     mixed
_atom_sites_solution_primary       ?
_atom_sites_solution_secondary     ?
loop_
  _atom_site_label
  _atom_site_type_symbol
  _atom_site_fract_x
  _atom_site_fract_y
  _atom_site_fract_z
  _atom_site_U_iso_or_equiv
  _atom_site_adp_type
  _atom_site_occupancy
  _atom_site_site_symmetry_order
  _atom_site_calc_flag
  _atom_site_refinement_flags_posn
  _atom_site_refinement_flags_adp
  _atom_site_refinement_flags_occupancy
  _atom_site_disorder_assembly
  _atom_site_disorder_group
 Mo1 Mo 0.51969(3) 0.65410(3) 0.77005(2) 0.02775(12) Uani 1 1 d . .
. . .
 H H 0.555(4) 0.543(4) 0.754(3) 0.042 Uiso 1 1 d . U . . .
 HA H 0.409(4) 0.656(4) 0.791(3) 0.042 Uiso 1 1 d . U . . .
 HB H 0.459(4) 0.740(4) 0.813(3) 0.042 Uiso 1 1 d . U . . .
 Mo2 Mo 0.51157(3) 0.73363(3) 0.69479(2) 0.02716(12) Uani 1 1 d . .
. . .
 HC H 0.554(4) 0.654(4) 0.637(3) 0.041 Uiso 1 1 d . U . . .
 HD H 0.393(4) 0.762(4) 0.682(3) 0.041 Uiso 1 1 d . U . . .
 HE H 0.450(4) 0.845(4) 0.707(3) 0.041 Uiso 1 1 d . U . . .
 Mo3 Mo 0.23801(3) 0.59392(3) 0.79354(2) 0.02679(12) Uani 1 1 d . .
. . .
 HF H 0.326(4) 0.532(4) 0.842(3) 0.040 Uiso 1 1 d . U . . .
 HG H 0.253(4) 0.691(4) 0.806(3) 0.040 Uiso 1 1 d . U . . .
 HH H 0.124(4) 0.694(4) 0.776(3) 0.040 Uiso 1 1 d . U . . .
 Mo4 Mo 0.31982(4) 0.55759(3) 0.71920(2) 0.02987(13) Uani 1 1 d . .
. . .
 HI H 0.248(4) 0.640(4) 0.672(3) 0.045 Uiso 1 1 d . U . . .
 HJ H 0.431(4) 0.492(4) 0.734(3) 0.045 Uiso 1 1 d . U . . .
 HK H 0.370(4) 0.633(4) 0.700(3) 0.045 Uiso 1 1 d . U . . .
```

```
Mo5 Mo 0.14370(3) 0.91631(3) 0.70920(2) 0.02834(12) Uani 1 1 d . .
. . .
HL H 0.209(4) 0.941(4) 0.644(3) 0.043 Uiso 1 1 d . U . . .
HM H 0.068(4) 0.877(4) 0.737(3) 0.043 Uiso 1 1 d . U . . .
HN H 0.234(4) 0.791(4) 0.694(3) 0.043 Uiso 1 1 d . U . . .
Mo6 Mo 0.18863(3) 0.93325(3) 0.77267(2) 0.02727(12) Uani 1 1 d . .
. . .
HO H 0.274(4) 0.965(4) 0.742(3) 0.041 Uiso 1 1 d . U . . .
HP H 0.278(4) 0.829(4) 0.790(3) 0.041 Uiso 1 1 d . U . . .
HQ H 0.136(4) 0.894(4) 0.836(3) 0.041 Uiso 1 1 d . U . . .
Li1 Li 0.5062(9) 0.5596(9) 0.6907(6) 0.050(3) Uani 1 1 d . . . . .
Li2 Li 0.4388(8) 0.5222(8) 0.8000(5) 0.041(3) Uani 1 1 d . . . . .
Li3 Li 0.1406(9) 0.7404(8) 0.7068(5) 0.045(3) Uani 1 1 d . . . . .
Li4 Li 0.1152(9) 0.7946(8) 0.8081(5) 0.046(3) Uani 1 1 d . . . . .
Li5 Li 0.3319(7) 0.9055(7) 0.6753(5) 0.036(2) Uani 1 1 d . . . . .
Li6 Li 0.3886(7) 0.8609(7) 0.7795(5) 0.038(3) Uani 1 1 d . . . . .
Li7 Li 0.3197(12) 0.7296(12) 0.8370(6) 0.078(5) Uani 1 1 d . . . .
.
Li8 Li 0.3185(17) 0.7259(13) 0.6464(7) 0.110(8) Uani 1 1 d . . . .
.
Li9 Li 0.3212(8) 0.7312(7) 0.7418(5) 0.039(3) Uani 1 1 d . . . . .
N1 N 0.6570(3) 0.6244(3) 0.7682(2) 0.0296(11) Uani 1 1 d . . . . .
N2 N 0.6448(3) 0.7192(3) 0.6893(2) 0.0292(11) Uani 1 1 d . . . . .
N3 N 0.1998(3) 0.4896(3) 0.8022(2) 0.0269(10) Uani 1 1 d . . . . .
N4 N 0.2939(4) 0.4476(3) 0.7232(2) 0.0325(12) Uani 1 1 d . . . . .
N5 N 0.0300(3) 1.0489(3) 0.7088(2) 0.0319(12) Uani 1 1 d . . . . .
N6 N 0.0848(3) 1.0720(3) 0.7738(2) 0.0295(11) Uani 1 1 d . . . . .
O1 O 0.5621(5) 0.4815(5) 0.6349(3) 0.099(3) Uani 1 1 d . U . . .
O2 O 0.5075(4) 0.4371(4) 0.8577(3) 0.0759(19) Uani 1 1 d . U . . .
O3 O 0.0561(6) 0.7799(5) 0.6558(3) 0.1084(7) Uani 1 1 d . U . . .
O4 O 0.0339(4) 0.7823(4) 0.8753(2) 0.0716(18) Uani 1 1 d . U . . .
O5 O 0.3760(3) 0.9666(3) 0.6103(2) 0.0535(14) Uani 1 1 d . U . . .
O6 O 0.3775(5) 0.9295(5) 0.8333(3) 0.1083(8) Uani 1 1 d . U . . .
O7 O 0.3068(7) 0.7158(7) 0.9129(4) 0.1524(10) Uani 1 1 d . U . . .
O8 O 0.3420(5) 0.7398(5) 0.5702(3) 0.087(2) Uani 1 1 d . U . . .
C1 C 0.6928(4) 0.6641(4) 0.7280(3) 0.0320(14) Uani 1 1 d . . . . .
H1 H 0.7535 0.6535 0.7264 0.038 Uiso 1 1 calc R U . . .
C2 C 0.7165(4) 0.5540(4) 0.8042(3) 0.0362(15) Uani 1 1 d . . . . .
C3 C 0.7754(4) 0.4689(5) 0.7861(3) 0.0459(18) Uani 1 1 d . . . . .
C4 C 0.8339(5) 0.4011(5) 0.8232(4) 0.060(2) Uani 1 1 d . . . . .
H4 H 0.8766 0.3429 0.8124 0.072 Uiso 1 1 calc R U . . .
C5 C 0.8301(6) 0.4173(6) 0.8724(4) 0.059(2) Uani 1 1 d . . . . .
H5 H 0.8704 0.3711 0.8955 0.071 Uiso 1 1 calc R U . . .
C6 C 0.7688(5) 0.4999(5) 0.8895(3) 0.051(2) Uani 1 1 d . . . . .
H6 H 0.7656 0.5093 0.9250 0.062 Uiso 1 1 calc R U . . .
C7 C 0.7107(4) 0.5707(5) 0.8559(3) 0.0426(17) Uani 1 1 d . . . . .
C8 C 0.7804(5) 0.4477(5) 0.7322(4) 0.058(2) Uani 1 1 d . . . . .
H8 H 0.7281 0.5004 0.7158 0.069 Uiso 1 1 calc R U . . .
C9 C 0.8671(7) 0.4393(8) 0.6947(4) 0.098(4) Uani 1 1 d . . . . .
H9A H 0.8670 0.4982 0.6875 0.147 Uiso 1 1 calc R U . . .
H9B H 0.8695 0.4207 0.6609 0.147 Uiso 1 1 calc R U . . .
H9C H 0.9204 0.3937 0.7115 0.147 Uiso 1 1 calc R U . . .
C10 C 0.7688(8) 0.3628(6) 0.7349(5) 0.101(4) Uani 1 1 d . . . . .
H10A H 0.8159 0.3103 0.7535 0.151 Uiso 1 1 calc R U . . .
H10B H 0.7747 0.3509 0.6984 0.151 Uiso 1 1 calc R U . . .
H10C H 0.7083 0.3733 0.7545 0.151 Uiso 1 1 calc R U . . .
C11 C 0.6468(5) 0.6617(5) 0.8759(3) 0.0504(19) Uani 1 1 d . . . .
.
```

H11 H 0.6058 0.7005 0.8484 0.061 Uiso 1 1 calc R U . . .
C12 C 0.6975(7) 0.7102(7) 0.8823(5) 0.097(4) Uani 1 1 d . . . . .
H12A H 0.7421 0.6712 0.9067 0.145 Uiso 1 1 calc R U . . .
H12B H 0.6539 0.7672 0.8972 0.145 Uiso 1 1 calc R U . . .
H12C H 0.7292 0.7233 0.8473 0.145 Uiso 1 1 calc R U . . .
C13 C 0.5871(7) 0.6549(8) 0.9285(4) 0.097(4) Uani 1 1 d . . . . .
H13A H 0.5551 0.6223 0.9252 0.146 Uiso 1 1 calc R U . . .
H13B H 0.5423 0.7160 0.9375 0.146 Uiso 1 1 calc R U . . .
H13C H 0.6251 0.6218 0.9569 0.146 Uiso 1 1 calc R U . . .
C14 C 0.6797(4) 0.7722(4) 0.6514(3) 0.0353(15) Uani 1 1 d . . . .
.
C15 C 0.7226(5) 0.7468(5) 0.5998(3) 0.0497(19) Uani 1 1 d . . . .
.
C16 C 0.7490(6) 0.8065(7) 0.5624(3) 0.063(2) Uani 1 1 d . . . . .
H16 H 0.7778 0.7907 0.5273 0.075 Uiso 1 1 calc R U . . .
C17 C 0.7340(6) 0.8866(7) 0.5755(4) 0.070(3) Uani 1 1 d . . . . .
H17 H 0.7524 0.9258 0.5496 0.084 Uiso 1 1 calc R U . . .
C18 C 0.6924(6) 0.9102(6) 0.6259(4) 0.062(2) Uani 1 1 d . . . . .
H18 H 0.6820 0.9661 0.6346 0.074 Uiso 1 1 calc R U . . .
C19 C 0.6651(5) 0.8540(5) 0.6647(3) 0.0405(16) Uani 1 1 d . . . .
.
C20 C 0.7437(7) 0.6569(6) 0.5850(4) 0.069(3) Uani 1 1 d . . . . .
H20 H 0.7228 0.6235 0.6173 0.083 Uiso 1 1 calc R U . . .
C21 C 0.8484(9) 0.5980(9) 0.5691(6) 0.153(7) Uani 1 1 d . . . . .
H21A H 0.8700 0.6277 0.5363 0.230 Uiso 1 1 calc R U . . .
H21B H 0.8611 0.5379 0.5629 0.230 Uiso 1 1 calc R U . . .
H21C H 0.8799 0.5912 0.5982 0.230 Uiso 1 1 calc R U . . .
C22 C 0.6919(11) 0.6718(8) 0.5409(5) 0.122(5) Uani 1 1 d . . . . .
H22A H 0.6272 0.7141 0.5507 0.184 Uiso 1 1 calc R U . . .
H22B H 0.6981 0.6137 0.5359 0.184 Uiso 1 1 calc R U . . .
H22C H 0.7167 0.6974 0.5074 0.184 Uiso 1 1 calc R U . . .
C23 C 0.6232(6) 0.8816(5) 0.7202(3) 0.0507(19) Uani 1 1 d . . . .
.
H23 H 0.6014 0.8374 0.7410 0.061 Uiso 1 1 calc R U . . .
C24 C 0.6920(8) 0.8776(8) 0.7495(4) 0.096(4) Uani 1 1 d . . . . .
H24A H 0.7456 0.8174 0.7489 0.144 Uiso 1 1 calc R U . . .
H24B H 0.6645 0.8885 0.7868 0.144 Uiso 1 1 calc R U . . .
H24C H 0.7104 0.9242 0.7318 0.144 Uiso 1 1 calc R U . . .
C25 C 0.5403(7) 0.9770(6) 0.7194(4) 0.083(3) Uani 1 1 d . . . . .
H25A H 0.5599 1.0220 0.6998 0.125 Uiso 1 1 calc R U . . .
H25B H 0.5134 0.9910 0.7563 0.125 Uiso 1 1 calc R U . . .
H25C H 0.4947 0.9788 0.7016 0.125 Uiso 1 1 calc R U . . .
C26 C 0.2362(4) 0.4354(4) 0.7643(3) 0.0312(13) Uani 1 1 d . . . .
.
H26 H 0.2211 0.3880 0.7665 0.037 Uiso 1 1 calc R U . . .
C27 C 0.1270(4) 0.4853(4) 0.8418(3) 0.0320(14) Uani 1 1 d . . . .
.
C28 C 0.1427(5) 0.4421(5) 0.8934(3) 0.0394(16) Uani 1 1 d . . . .
.
C29 C 0.0694(5) 0.4438(5) 0.9318(3) 0.0468(18) Uani 1 1 d . . . .
.
H29 H 0.0795 0.4150 0.9673 0.056 Uiso 1 1 calc R U . . .
C30 C -0.0178(5) 0.4866(6) 0.9191(3) 0.056(2) Uani 1 1 d . . . . .
H30 H -0.0675 0.4877 0.9457 0.067 Uiso 1 1 calc R U . . .
C31 C -0.0322(5) 0.5274(5) 0.8681(3) 0.051(2) Uani 1 1 d . . . . .
H31 H -0.0922 0.5552 0.8595 0.062 Uiso 1 1 calc R U . . .
C32 C 0.0387(5) 0.5295(4) 0.8280(3) 0.0390(16) Uani 1 1 d . . . .
.

C33 C 0.2377(5) 0.3907(6) 0.9087(3) 0.055(2) Uani 1 1 d . . . . .
H33 H 0.2803 0.4011 0.8774 0.066 Uiso 1 1 calc R U . . .
C34 C 0.2444(7) 0.4240(9) 0.9559(4) 0.104(4) Uani 1 1 d . . . . .
H34A H 0.1997 0.4194 0.9866 0.156 Uiso 1 1 calc R U . . .
H34B H 0.2321 0.4875 0.9465 0.156 Uiso 1 1 calc R U . . .
H34C H 0.3060 0.3867 0.9654 0.156 Uiso 1 1 calc R U . . .
C35 C 0.2683(8) 0.2903(7) 0.9193(6) 0.119(5) Uani 1 1 d . . . . .
H35A H 0.2256 0.2783 0.9486 0.178 Uiso 1 1 calc R U . . .
H35B H 0.3295 0.2583 0.9294 0.178 Uiso 1 1 calc R U . . .
H35C H 0.2696 0.2689 0.8868 0.178 Uiso 1 1 calc R U . . .
C36 C 0.0172(5) 0.5774(5) 0.7719(3) 0.0465(18) Uani 1 1 d . . . .
.
H36 H 0.0740 0.5780 0.7503 0.056 Uiso 1 1 calc R U . . .
C37 C -0.0072(8) 0.5238(8) 0.7449(4) 0.091(4) Uani 1 1 d . . . . .
H37A H 0.0439 0.4624 0.7425 0.137 Uiso 1 1 calc R U . . .
H37B H -0.0199 0.5543 0.7088 0.137 Uiso 1 1 calc R U . . .
H37C H -0.0615 0.5201 0.7661 0.137 Uiso 1 1 calc R U . . .
C38 C -0.0565(6) 0.6748(7) 0.7732(4) 0.091(4) Uani 1 1 d . . . . .
H38A H -0.0704 0.7020 0.7368 0.137 Uiso 1 1 calc R U . . .
H38B H -0.0353 0.7090 0.7869 0.137 Uiso 1 1 calc R U . . .
H38C H -0.1115 0.6765 0.7966 0.137 Uiso 1 1 calc R U . . .
C39 C 0.3435(5) 0.3790(5) 0.6886(3) 0.0456(18) Uani 1 1 d . . . .
.
C40 C 0.3222(6) 0.3932(6) 0.6372(3) 0.059(2) Uani 1 1 d . . . . .
C41 C 0.3754(8) 0.3258(7) 0.6034(4) 0.082(3) Uani 1 1 d . . . . .
H41 H 0.3606 0.3350 0.5686 0.098 Uiso 1 1 calc R U . . .
C42 C 0.4477(9) 0.2478(7) 0.6190(5) 0.096(4) Uani 1 1 d . . . . .
H42 H 0.4825 0.2025 0.5955 0.116 Uiso 1 1 calc R U . . .
C43 C 0.4708(7) 0.2338(6) 0.6687(4) 0.074(3) Uani 1 1 d . . . . .
H43 H 0.5224 0.1791 0.6789 0.088 Uiso 1 1 calc R U . . .
C44 C 0.4195(6) 0.2987(5) 0.7050(4) 0.058(2) Uani 1 1 d . . . . .
C45 C 0.2404(7) 0.4792(6) 0.6174(4) 0.070(3) Uani 1 1 d . . . . .
H45 H 0.2129 0.5212 0.6456 0.083 Uiso 1 1 calc R U . . .
C46 C 0.2711(10) 0.5276(8) 0.5659(4) 0.112(5) Uani 1 1 d . . . . .
H46A H 0.3116 0.5476 0.5734 0.168 Uiso 1 1 calc R U . . .
H46B H 0.2175 0.5805 0.5527 0.168 Uiso 1 1 calc R U . . .
H46C H 0.3038 0.4857 0.5387 0.168 Uiso 1 1 calc R U . . .
C47 C 0.1671(9) 0.4596(9) 0.6089(5) 0.110(4) Uani 1 1 d . . . . .
H47A H 0.1953 0.4083 0.5879 0.165 Uiso 1 1 calc R U . . .
H47B H 0.1225 0.5134 0.5894 0.165 Uiso 1 1 calc R U . . .
H47C H 0.1363 0.4445 0.6437 0.165 Uiso 1 1 calc R U . . .
C48 C 0.4477(6) 0.2803(6) 0.7595(4) 0.071(3) Uani 1 1 d . . . . .
H48 H 0.4101 0.3377 0.7765 0.085 Uiso 1 1 calc R U . . .
C49 C 0.4243(9) 0.2065(7) 0.7957(5) 0.111(4) Uani 1 1 d . . . . .
H49A H 0.3623 0.2205 0.7929 0.166 Uiso 1 1 calc R U . . .
H49B H 0.4279 0.2058 0.8331 0.166 Uiso 1 1 calc R U . . .
H49C H 0.4679 0.1469 0.7840 0.166 Uiso 1 1 calc R U . . .
C50 C 0.5472(8) 0.2566(10) 0.7549(7) 0.144(6) Uani 1 1 d . . . . .
H50A H 0.5860 0.2021 0.7367 0.217 Uiso 1 1 calc R U . . .
H50B H 0.5629 0.2449 0.7909 0.217 Uiso 1 1 calc R U . . .
H50C H 0.5570 0.3074 0.7343 0.217 Uiso 1 1 calc R U . . .
C51 C 0.0237(4) 1.1030(4) 0.7415(3) 0.0332(14) Uani 1 1 d . . . .
.
H51 H -0.0246 1.1635 0.7415 0.040 Uiso 1 1 calc R U . . .
C52 C -0.0477(5) 1.0789(4) 0.6836(3) 0.0412(16) Uani 1 1 d . . . .
.
C53 C -0.0470(6) 1.1153(6) 0.6297(4) 0.062(2) Uani 1 1 d . . . . .
C54 C -0.1205(8) 1.1372(8) 0.6048(4) 0.090(3) Uani 1 1 d . . . . .

H54 H -0.1199 1.1608 0.5679 0.108 Uiso 1 1 calc R U . . .
C55 C -0.1939(7) 1.1252(8) 0.6330(5) 0.089(3) Uani 1 1 d . . . . .
H55 H -0.2431 1.1391 0.6155 0.107 Uiso 1 1 calc R U . . .
C56 C -0.1952(5) 1.0929(6) 0.6864(4) 0.071(3) Uani 1 1 d . . . . .
H56 H -0.2472 1.0872 0.7061 0.085 Uiso 1 1 calc R U . . .
C57 C -0.1225(5) 1.0683(5) 0.7125(3) 0.0503(19) Uani 1 1 d . . . .
.
C58 C 0.0331(7) 1.1305(7) 0.5975(4) 0.072(3) Uani 1 1 d . . . . .
H58 H 0.0770 1.1134 0.6226 0.086 Uiso 1 1 calc R U . . .
C59 C 0.0836(8) 1.0704(9) 0.5530(5) 0.113(4) Uani 1 1 d . . . . .
H59A H 0.1077 1.0065 0.5685 0.170 Uiso 1 1 calc R U . . .
H59B H 0.1341 1.0821 0.5332 0.170 Uiso 1 1 calc R U . . .
H59C H 0.0415 1.0838 0.5285 0.170 Uiso 1 1 calc R U . . .
C60 C 0.0022(10) 1.2312(8) 0.5747(5) 0.122(5) Uani 1 1 d . . . . .
H60A H -0.0324 1.2472 0.5454 0.183 Uiso 1 1 calc R U . . .
H60B H 0.0559 1.2408 0.5611 0.183 Uiso 1 1 calc R U . . .
H60C H -0.0365 1.2696 0.6032 0.183 Uiso 1 1 calc R U . . .
C61 C -0.1262(5) 1.0312(5) 0.7711(3) 0.0500(19) Uani 1 1 d . . . .
.
H61 H -0.0642 1.0075 0.7806 0.060 Uiso 1 1 calc R U . . .
C62 C -0.1932(7) 1.1055(7) 0.8061(4) 0.085(3) Uani 1 1 d . . . . .
H62A H -0.1770 1.1550 0.7983 0.128 Uiso 1 1 calc R U . . .
H62B H -0.1905 1.0805 0.8440 0.128 Uiso 1 1 calc R U . . .
H62C H -0.2551 1.1289 0.7986 0.128 Uiso 1 1 calc R U . . .
C63 C -0.1490(7) 0.9510(7) 0.7837(4) 0.076(3) Uani 1 1 d . . . . .
H63A H -0.2094 0.9719 0.7745 0.113 Uiso 1 1 calc R U . . .
H63B H -0.1490 0.9282 0.8220 0.113 Uiso 1 1 calc R U . . .
H63C H -0.1030 0.9021 0.7625 0.113 Uiso 1 1 calc R U . . .
C64 C 0.0842(4) 1.1361(4) 0.8006(3) 0.0372(15) Uani 1 1 d . . . .
.
C65 C 0.0458(5) 1.1435(5) 0.8548(3) 0.0477(18) Uani 1 1 d . . . .
.
C66 C 0.0482(6) 1.2072(5) 0.8796(4) 0.059(2) Uani 1 1 d . . . . .
H66 H 0.0224 1.2128 0.9163 0.071 Uiso 1 1 calc R U . . .
C67 C 0.0861(6) 1.2611(6) 0.8529(4) 0.065(2) Uani 1 1 d . . . . .
H67 H 0.0865 1.3039 0.8706 0.077 Uiso 1 1 calc R U . . .
C68 C 0.1241(6) 1.2529(5) 0.7995(4) 0.060(2) Uani 1 1 d . . . . .
H68 H 0.1500 1.2912 0.7808 0.072 Uiso 1 1 calc R U . . .
C69 C 0.1256(4) 1.1907(4) 0.7724(3) 0.0430(17) Uani 1 1 d . . . .
.
C70 C 0.0043(6) 1.0843(5) 0.8859(3) 0.055(2) Uani 1 1 d . . . . .
H70 H 0.0045 1.0484 0.8601 0.066 Uiso 1 1 calc R U . . .
C71 C 0.0627(9) 1.0155(7) 0.9292(4) 0.097(4) Uani 1 1 d . . . . .
H71A H 0.1257 0.9804 0.9121 0.146 Uiso 1 1 calc R U . . .
H71B H 0.0373 0.9742 0.9468 0.146 Uiso 1 1 calc R U . . .
H71C H 0.0620 1.0486 0.9559 0.146 Uiso 1 1 calc R U . . .
C72 C -0.0964(7) 1.1403(8) 0.9115(5) 0.106(4) Uani 1 1 d . . . . .
H72A H -0.0987 1.1752 0.9377 0.159 Uiso 1 1 calc R U . . .
H72B H -0.1217 1.0991 0.9294 0.159 Uiso 1 1 calc R U . . .
H72C H -0.1322 1.1822 0.8834 0.159 Uiso 1 1 calc R U . . .
C73 C 0.1723(5) 1.1814(5) 0.7152(3) 0.052(2) Uani 1 1 d . . . . .
H73 H 0.1803 1.1235 0.7063 0.063 Uiso 1 1 calc R U . . .
C74 C 0.2675(6) 1.1744(7) 0.7064(4) 0.078(3) Uani 1 1 d . . . . .
H74A H 0.2627 1.2280 0.7175 0.117 Uiso 1 1 calc R U . . .
H74B H 0.2934 1.1712 0.6683 0.117 Uiso 1 1 calc R U . . .
H74C H 0.3071 1.1195 0.7278 0.117 Uiso 1 1 calc R U . . .
C75 C 0.1130(7) 1.2584(6) 0.6768(4) 0.079(3) Uani 1 1 d . . . . .
H75A H 0.0602 1.2511 0.6754 0.118 Uiso 1 1 calc R U . . .

H75B H 0.1488 1.2569 0.6408 0.118 Uiso 1 1 calc R U . . .
H75C H 0.0920 1.3167 0.6893 0.118 Uiso 1 1 calc R U . . .
C76 C 0.6439(9) 0.4674(10) 0.6031(6) 0.143(5) Uani 1 1 d D U . . .
H76A H 0.6872 0.4622 0.6248 0.171 Uiso 1 1 calc R U . . .
H76B H 0.6348 0.5188 0.5742 0.171 Uiso 1 1 calc R U . . .
C77 C 0.6798(10) 0.3827(10) 0.5801(7) 0.165(6) Uani 1 1 d D U . .
.
H77A H 0.7159 0.3291 0.6043 0.198 Uiso 1 1 calc R U . . .
H77B H 0.7179 0.3834 0.5446 0.198 Uiso 1 1 calc R U . . .
C78 C 0.5935(11) 0.3841(12) 0.5751(7) 0.192(7) Uani 1 1 d D U . .
.
H78A H 0.5803 0.4055 0.5378 0.230 Uiso 1 1 calc R U . . .
H78B H 0.5981 0.3227 0.5856 0.230 Uiso 1 1 calc R U . . .
C79 C 0.5229(10) 0.4470(11) 0.6116(6) 0.159(6) Uani 1 1 d D U . .
.
H79A H 0.4749 0.4971 0.5916 0.191 Uiso 1 1 calc R U . . .
H79B H 0.4943 0.4151 0.6396 0.191 Uiso 1 1 calc R U . . .
C80 C 0.5899(7) 0.4226(8) 0.8706(6) 0.109(4) Uani 1 1 d D U . . .
H80A H 0.5772 0.4591 0.8992 0.130 Uiso 1 1 calc R U . . .
H80B H 0.6260 0.4401 0.8387 0.130 Uiso 1 1 calc R U . . .
C81 C 0.6400(8) 0.3248(9) 0.8891(7) 0.149(6) Uani 1 1 d D U . . .
H81A H 0.6774 0.3137 0.9164 0.179 Uiso 1 1 calc R U . . .
H81B H 0.6801 0.2902 0.8588 0.179 Uiso 1 1 calc R U . . .
C82 C 0.5665(9) 0.2997(9) 0.9124(7) 0.153(6) Uani 1 1 d D U . . .
H82A H 0.5834 0.2394 0.9032 0.184 Uiso 1 1 calc R U . . .
H82B H 0.5546 0.2974 0.9519 0.184 Uiso 1 1 calc R U . . .
C83 C 0.4861(7) 0.3703(7) 0.8890(5) 0.097(4) Uani 1 1 d D U . . .
H83A H 0.4680 0.3438 0.8666 0.117 Uiso 1 1 calc R U . . .
H83B H 0.4345 0.3973 0.9179 0.117 Uiso 1 1 calc R U . . .
C84 C -0.0024(9) 0.8698(7) 0.6381(5) 0.1085(7) Uani 1 1 d D U . .
.
H84A H 0.0337 0.9020 0.6206 0.130 Uiso 1 1 calc R U . . .
H84B H -0.0445 0.9011 0.6691 0.130 Uiso 1 1 calc R U . . .
C85 C -0.0528(9) 0.8725(7) 0.6016(5) 0.1085(7) Uani 1 1 d D U . .
.
H85A H -0.0391 0.9032 0.5660 0.130 Uiso 1 1 calc R U . . .
H85B H -0.1191 0.9054 0.6146 0.130 Uiso 1 1 calc R U . . .
C86 C -0.0239(9) 0.7755(6) 0.5982(5) 0.1085(7) Uani 1 1 d D U . .
.
H86A H -0.0755 0.7619 0.6132 0.130 Uiso 1 1 calc R U . . .
H86B H -0.0036 0.7632 0.5604 0.130 Uiso 1 1 calc R U . . .
C87 C 0.0511(8) 0.7196(7) 0.6292(5) 0.1085(7) Uani 1 1 d D U . . .
H87A H 0.0389 0.6735 0.6554 0.130 Uiso 1 1 calc R U . . .
H87B H 0.1091 0.6881 0.6052 0.130 Uiso 1 1 calc R U . . .
C88 C -0.0218(7) 0.8534(7) 0.9079(4) 0.088(3) Uani 1 1 d D U . . .
H88A H 0.0139 0.8808 0.9142 0.105 Uiso 1 1 calc R U . . .
H88B H -0.0741 0.9014 0.8902 0.105 Uiso 1 1 calc R U . . .
C89 C -0.0533(13) 0.8144(10) 0.9572(5) 0.179(7) Uani 1 1 d D U . .
.
H89A H -0.1195 0.8531 0.9680 0.215 Uiso 1 1 calc R U . . .
H89B H -0.0206 0.8087 0.9858 0.215 Uiso 1 1 calc R U . . .
C90 C -0.0358(14) 0.7245(10) 0.9491(6) 0.202(8) Uani 1 1 d D U . .
.
H90A H -0.0041 0.6778 0.9778 0.243 Uiso 1 1 calc R U . . .
H90B H -0.0937 0.7243 0.9493 0.243 Uiso 1 1 calc R U . . .
C91 C 0.0215(10) 0.7068(8) 0.8973(5) 0.130(5) Uani 1 1 d D U . . .
H91A H 0.0812 0.6526 0.9016 0.155 Uiso 1 1 calc R U . . .
H91B H -0.0081 0.6953 0.8734 0.155 Uiso 1 1 calc R U . . .

C92 C 0.3190(6) 1.0510(5) 0.5830(4) 0.064(2) Uani 1 1 d D U . . .
H92A H 0.2661 1.0878 0.6085 0.077 Uiso 1 1 calc R U . . .
H92B H 0.2960 1.0410 0.5547 0.077 Uiso 1 1 calc R U . . .
C93 C 0.3754(6) 1.1004(6) 0.5581(4) 0.069(2) Uani 1 1 d D U . . .
H93A H 0.3805 1.1102 0.5185 0.083 Uiso 1 1 calc R U . . .
H93B H 0.3476 1.1599 0.5716 0.083 Uiso 1 1 calc R U . . .
C94 C 0.4666(6) 1.0383(6) 0.5753(4) 0.066(2) Uani 1 1 d D U . . .
H94A H 0.4722 1.0569 0.6073 0.079 Uiso 1 1 calc R U . . .
H94B H 0.5163 1.0376 0.5461 0.079 Uiso 1 1 calc R U . . .
C95 C 0.4691(5) 0.9461(5) 0.5882(3) 0.0522(19) Uani 1 1 d D U . . .
H95A H 0.4894 0.9161 0.5554 0.063 Uiso 1 1 calc R U . . .
H95B H 0.5106 0.9062 0.6146 0.063 Uiso 1 1 calc R U . . .
C96 C 0.4397(8) 0.8977(8) 0.8685(5) 0.1084(8) Uani 1 1 d D U . . .
H96A H 0.4858 0.9194 0.8546 0.130 Uiso 1 1 calc R U . . .
H96B H 0.4713 0.8305 0.8745 0.130 Uiso 1 1 calc R U . . .
C97 C 0.3829(7) 0.9362(7) 0.9192(4) 0.1084(8) Uani 1 1 d D U . . .
H97A H 0.3551 0.8981 0.9408 0.130 Uiso 1 1 calc R U . . .
H97B H 0.4193 0.9423 0.9411 0.130 Uiso 1 1 calc R U . . .
C98 C 0.3127(8) 1.0260(7) 0.8989(5) 0.1084(8) Uani 1 1 d D U . . .
H98A H 0.2533 1.0435 0.9227 0.130 Uiso 1 1 calc R U . . .
H98B H 0.3313 1.0735 0.8956 0.130 Uiso 1 1 calc R U . . .
C99 C 0.3077(8) 1.0121(8) 0.8466(5) 0.1084(8) Uani 1 1 d D U . . .
H99A H 0.2483 1.0145 0.8473 0.130 Uiso 1 1 calc R U . . .
H99B H 0.3120 1.0612 0.8192 0.130 Uiso 1 1 calc R U . . .
C100 C 0.2348(11) 0.7506(10) 0.9472(6) 0.1524(10) Uani 1 1 d D U . . .
H10D H 0.1806 0.7893 0.9289 0.183 Uiso 1 1 calc R U . . .
H10E H 0.2408 0.7881 0.9686 0.183 Uiso 1 1 calc R U . . .
C101 C 0.2259(10) 0.6716(9) 0.9827(6) 0.1525(10) Uani 1 1 d D U . . .
H10F H 0.2050 0.6830 1.0207 0.183 Uiso 1 1 calc R U . . .
H10G H 0.1834 0.6578 0.9709 0.183 Uiso 1 1 calc R U . . .
C102 C 0.3209(9) 0.5978(10) 0.9745(6) 0.1525(10) Uani 1 1 d D U . . .
H10H H 0.3464 0.5745 1.0086 0.183 Uiso 1 1 calc R U . . .
H10I H 0.3239 0.5470 0.9603 0.183 Uiso 1 1 calc R U . . .
C103 C 0.3670(10) 0.6378(10) 0.9380(6) 0.1524(10) Uani 1 1 d D U . . .
H10J H 0.4028 0.6510 0.9565 0.183 Uiso 1 1 calc R U . . .
H10K H 0.4099 0.5959 0.9114 0.183 Uiso 1 1 calc R U . . .
C104 C 0.2678(10) 0.7858(11) 0.5440(5) 0.124(4) Uani 1 1 d D U . . .
H10L H 0.2128 0.8212 0.5688 0.148 Uiso 1 1 calc R U . . .
H10M H 0.2571 0.7424 0.5308 0.148 Uiso 1 1 calc R U . . .
C107 C 0.4172(10) 0.7512(13) 0.5399(6) 0.145(5) Uani 1 1 d D U . . .
H10N H 0.4561 0.7035 0.5159 0.174 Uiso 1 1 calc R U . . .
H10O H 0.4548 0.7527 0.5631 0.174 Uiso 1 1 calc R U . . .
C105 C 0.2854(14) 0.8473(13) 0.4986(7) 0.197(8) Uani 1 1 d D U . . .
H10P H 0.2954 0.8263 0.4638 0.236 Uiso 1 1 calc R U . . .
H10Q H 0.2349 0.9103 0.4989 0.236 Uiso 1 1 calc R U . . .
C106 C 0.3677(14) 0.8400(13) 0.5099(8) 0.197(8) Uani 1 1 d D U . . .
H10R H 0.3521 0.8887 0.5311 0.237 Uiso 1 1 calc R U . . .
H10S H 0.4065 0.8470 0.4758 0.237 Uiso 1 1 calc R U . . .

```
loop_
  _atom_site_aniso_label
  _atom_site_aniso_U_11
  _atom_site_aniso_U_22
  _atom_site_aniso_U_33
  _atom_site_aniso_U_23
  _atom_site_aniso_U_13
  _atom_site_aniso_U_12
 Mo1 0.0237(2) 0.0230(2) 0.0369(3) 0.0019(2) -0.0069(2) -0.0125(2)
 Mo2 0.0246(3) 0.0249(3) 0.0339(3) -0.0002(2) -0.0060(2) -0.0137(2)
 Mo3 0.0283(3) 0.0231(2) 0.0338(3) -0.0067(2) 0.0004(2) -0.0166(2)
 Mo4 0.0335(3) 0.0260(3) 0.0363(3) -0.0102(2) 0.0042(2) -0.0201(2)
 Mo5 0.0264(3) 0.0225(2) 0.0361(3) -0.0033(2) -0.0056(2) -0.0111(2)
 Mo6 0.0268(3) 0.0188(2) 0.0371(3) -0.0046(2) -0.0052(2) -0.0107(2)
 Li1 0.045(7) 0.052(7) 0.062(9) -0.020(6) -0.004(6) -0.026(6)
 Li2 0.049(7) 0.039(6) 0.041(7) 0.002(5) -0.015(5) -0.024(6)
 Li3 0.055(7) 0.041(7) 0.050(7) -0.015(6) -0.012(6) -0.024(6)
 Li4 0.061(8) 0.028(6) 0.051(8) -0.016(5) 0.011(6) -0.026(6)
 Li5 0.027(5) 0.030(6) 0.050(7) 0.000(5) -0.011(5) -0.012(4)
 Li6 0.038(6) 0.037(6) 0.038(6) -0.010(5) -0.004(5) -0.016(5)
 Li7 0.088(12) 0.080(11) 0.032(8) -0.007(7) 0.005(7) -0.020(9)
 Li8 0.17(2) 0.072(12) 0.046(10) -0.006(9) -0.002(11) -0.034(13)
 Li9 0.038(6) 0.035(6) 0.054(7) -0.011(5) -0.014(5) -0.018(5)
 N1 0.021(2) 0.031(3) 0.036(3) 0.005(2) -0.010(2) -0.013(2)
 N2 0.028(3) 0.025(3) 0.038(3) -0.004(2) -0.003(2) -0.015(2)
 N3 0.033(3) 0.023(2) 0.028(3) -0.004(2) -0.001(2) -0.017(2)
 N4 0.041(3) 0.031(3) 0.037(3) -0.013(2) 0.006(2) -0.027(2)
 N5 0.025(3) 0.023(3) 0.043(3) -0.001(2) -0.008(2) -0.008(2)
 N6 0.030(3) 0.019(2) 0.040(3) -0.006(2) 0.000(2) -0.013(2)
 O1 0.096(5) 0.117(6) 0.118(6) -0.089(5) 0.063(4) -0.080(5)
 O2 0.055(3) 0.075(4) 0.104(5) 0.035(4) -0.035(3) -0.045(3)
 O3 0.1374(11) 0.0720(11) 0.1326(11) -0.0139(10) -0.0909(10) -
0.0307(10)
 O4 0.085(4) 0.060(4) 0.072(4) -0.028(3) 0.030(3) -0.046(3)
 O5 0.040(3) 0.046(3) 0.063(4) 0.023(3) -0.013(2) -0.021(2)
 O6 0.0983(11) 0.0895(11) 0.1157(11) -0.0567(10) -0.0526(10)
0.0052(10)
 O7 0.1434(12) 0.1336(12) 0.0938(12) 0.0211(11) 0.0111(12) -
0.0272(12)
 O8 0.099(5) 0.115(6) 0.044(4) 0.002(4) -0.008(3) -0.053(5)
 C1 0.024(3) 0.028(3) 0.045(4) -0.002(3) -0.008(3) -0.012(3)
 C2 0.024(3) 0.027(3) 0.055(4) 0.012(3) -0.016(3) -0.012(3)
 C3 0.027(3) 0.030(3) 0.076(6) 0.004(3) -0.011(3) -0.013(3)
 C4 0.035(4) 0.031(4) 0.099(7) 0.013(4) -0.020(4) -0.008(3)
 C5 0.048(5) 0.055(5) 0.073(6) 0.024(4) -0.025(4) -0.028(4)
 C6 0.037(4) 0.060(5) 0.052(5) 0.013(4) -0.015(3) -0.023(4)
 C7 0.031(3) 0.046(4) 0.049(4) 0.012(3) -0.011(3) -0.023(3)
 C8 0.036(4) 0.040(4) 0.089(7) -0.013(4) -0.012(4) -0.009(3)
 C9 0.062(6) 0.113(9) 0.094(8) -0.052(7) 0.013(6) -0.018(6)
 C10 0.115(9) 0.040(5) 0.156(12) -0.013(6) -0.038(9) -0.035(6)
 C11 0.048(4) 0.058(5) 0.042(4) -0.001(4) -0.014(4) -0.022(4)
 C12 0.068(7) 0.083(8) 0.142(11) -0.034(8) -0.005(7) -0.034(6)
 C13 0.076(7) 0.117(9) 0.066(7) -0.006(6) 0.010(6) -0.032(7)
 C14 0.024(3) 0.033(3) 0.041(4) 0.007(3) -0.004(3) -0.011(3)
 C15 0.043(4) 0.053(5) 0.048(5) 0.003(4) -0.005(3) -0.023(4)
 C16 0.055(5) 0.084(7) 0.043(5) 0.005(4) 0.003(4) -0.038(5)
 C17 0.072(6) 0.089(7) 0.068(6) 0.011(5) -0.004(5) -0.062(6)
 C18 0.077(6) 0.057(5) 0.073(6) 0.013(4) -0.021(5) -0.051(5)
```

C19	0.042(4)	0.043(4)	0.046(4)	0.007(3)	-0.012(3)	-0.030(3)
C20	0.088(7)	0.054(5)	0.047(5)	-0.007(4)	0.004(5)	-0.025(5)
C21	0.116(11)	0.084(9)	0.167(15)	-0.039(9)	0.060(10)	-0.004(8)
C22	0.225(16)	0.107(10)	0.074(8)	-0.020(7)	-0.023(9)	-0.103(11)
C23	0.068(5)	0.045(4)	0.052(5)	-0.004(4)	-0.017(4)	-0.034(4)
C24	0.107(9)	0.129(10)	0.084(8)	-0.012(7)	-0.034(7)	-0.071(8)
C25	0.120(9)	0.040(5)	0.078(7)	-0.011(5)	-0.007(6)	-0.031(5)
C26	0.038(3)	0.027(3)	0.036(4)	-0.001(3)	-0.003(3)	-0.023(3)
C27	0.042(4)	0.028(3)	0.035(4)	-0.004(3)	0.001(3)	-0.026(3)
C28	0.042(4)	0.046(4)	0.039(4)	-0.007(3)	0.002(3)	-0.030(3)
C29	0.061(5)	0.052(4)	0.036(4)	-0.004(3)	-0.001(3)	-0.036(4)
C30	0.046(4)	0.063(5)	0.063(6)	-0.016(4)	0.018(4)	-0.036(4)
C31	0.030(4)	0.057(5)	0.064(5)	-0.005(4)	0.005(3)	-0.025(3)
C32	0.039(4)	0.032(3)	0.051(4)	-0.001(3)	-0.004(3)	-0.023(3)
C33	0.048(4)	0.075(6)	0.042(4)	0.011(4)	-0.014(4)	-0.034(4)
C34	0.078(7)	0.191(13)	0.063(7)	-0.028(8)	-0.019(6)	-0.070(8)
C35	0.083(8)	0.074(8)	0.165(14)	0.033(8)	-0.032(9)	-0.024(7)
C36	0.042(4)	0.051(4)	0.052(5)	0.010(4)	-0.014(3)	-0.030(4)
C37	0.120(9)	0.125(10)	0.071(7)	0.019(6)	-0.047(7)	-0.087(8)
C38	0.052(6)	0.084(7)	0.096(8)	0.028(6)	-0.026(6)	-0.010(5)
C39	0.056(5)	0.040(4)	0.055(5)	-0.019(3)	0.012(4)	-0.037(4)
C40	0.089(6)	0.053(5)	0.053(5)	-0.013(4)	0.012(4)	-0.056(5)
C41	0.134(10)	0.077(7)	0.060(6)	-0.039(5)	0.021(6)	-0.071(7)
C42	0.137(10)	0.073(7)	0.094(9)	-0.060(7)	0.061(8)	-0.074(8)
C43	0.079(6)	0.044(5)	0.093(8)	-0.036(5)	0.025(6)	-0.032(5)
C44	0.056(5)	0.037(4)	0.082(6)	-0.029(4)	0.016(4)	-0.026(4)
C45	0.118(8)	0.067(6)	0.046(5)	-0.007(4)	-0.011(5)	-0.061(6)
C46	0.188(14)	0.096(9)	0.058(7)	-0.003(6)	0.003(8)	-0.083(10)
C47	0.147(12)	0.128(11)	0.109(10)	-0.013(8)	-0.034(9)	-0.099(10)
C48	0.055(5)	0.037(4)	0.109(8)	-0.023(5)	-0.010(5)	-0.010(4)
C49	0.165(13)	0.050(6)	0.108(10)	-0.002(6)	-0.017(9)	-0.048(7)
C50	0.058(7)	0.148(13)	0.205(17)	-0.084(12)	-0.028(9)	-0.003(8)
C51	0.026(3)	0.022(3)	0.049(4)	0.000(3)	-0.004(3)	-0.011(2)
C52	0.036(4)	0.027(3)	0.054(5)	-0.003(3)	-0.017(3)	-0.005(3)
C53	0.056(5)	0.061(5)	0.067(6)	0.000(4)	-0.029(5)	-0.020(4)
C54	0.087(8)	0.098(8)	0.071(7)	0.017(6)	-0.046(6)	-0.029(7)
C55	0.060(6)	0.101(8)	0.095(9)	0.001(7)	-0.044(6)	-0.020(6)
C56	0.034(4)	0.080(7)	0.097(8)	-0.015(6)	-0.017(5)	-0.022(4)
C57	0.030(4)	0.042(4)	0.072(6)	-0.011(4)	-0.015(4)	-0.007(3)
C58	0.073(6)	0.078(7)	0.052(6)	0.012(5)	-0.014(5)	-0.032(5)
C59	0.106(10)	0.128(11)	0.079(8)	-0.017(8)	0.005(7)	-0.041(9)
C60	0.157(13)	0.093(9)	0.109(10)	0.041(8)	-0.034(9)	-0.068(9)
C61	0.032(4)	0.053(5)	0.068(6)	-0.003(4)	-0.007(4)	-0.024(3)
C62	0.071(7)	0.085(7)	0.084(8)	-0.027(6)	0.023(6)	-0.033(6)
C63	0.075(6)	0.081(7)	0.089(8)	0.001(6)	-0.013(6)	-0.055(6)
C64	0.033(3)	0.020(3)	0.055(4)	-0.011(3)	-0.005(3)	-0.007(3)
C65	0.040(4)	0.035(4)	0.062(5)	-0.018(4)	-0.002(4)	-0.012(3)
C66	0.061(5)	0.046(5)	0.062(6)	-0.023(4)	-0.002(4)	-0.016(4)
C67	0.066(6)	0.045(5)	0.090(7)	-0.029(5)	-0.009(5)	-0.024(4)
C68	0.059(5)	0.037(4)	0.095(7)	-0.015(4)	-0.005(5)	-0.031(4)
C69	0.031(3)	0.023(3)	0.070(5)	-0.010(3)	-0.005(3)	-0.008(3)
C70	0.069(5)	0.049(5)	0.049(5)	-0.023(4)	0.008(4)	-0.030(4)
C71	0.154(12)	0.078(7)	0.057(7)	0.005(5)	-0.017(7)	-0.057(8)
C72	0.089(8)	0.095(8)	0.135(11)	-0.052(8)	0.044(7)	-0.054(7)
C73	0.052(5)	0.030(4)	0.075(6)	-0.006(4)	0.006(4)	-0.026(3)
C74	0.052(5)	0.092(7)	0.097(8)	-0.007(6)	0.007(5)	-0.048(5)
C75	0.088(7)	0.054(5)	0.076(7)	0.001(5)	-0.004(6)	-0.027(5)
C76	0.097(9)	0.158(12)	0.186(13)	-0.107(10)	0.066(8)	-0.072(8)

```
 C77 0.152(10) 0.132(11) 0.183(14) -0.092(10) 0.073(11) -0.059(10)
 C78 0.212(14) 0.226(15) 0.202(14) -0.172(12) 0.102(12) -0.150(13)
 C79 0.154(10) 0.218(14) 0.181(13) -0.149(11) 0.076(9) -0.136(10)
 C80 0.074(7) 0.105(8) 0.163(11) 0.043(8) -0.058(7) -0.059(6)
 C81 0.079(7) 0.123(9) 0.193(14) 0.065(10) -0.049(9) -0.029(7)
 C82 0.137(10) 0.109(9) 0.208(15) 0.081(9) -0.080(10) -0.070(8)
 C83 0.087(7) 0.071(7) 0.135(10) 0.042(6) -0.032(7) -0.053(6)
 C84 0.1375(11) 0.0720(11) 0.1326(11) -0.0139(10) -0.0909(10) -
0.0306(10)
 C85 0.1375(11) 0.0720(11) 0.1326(11) -0.0139(10) -0.0908(10) -
0.0306(10)
 C86 0.1375(11) 0.0720(11) 0.1326(11) -0.0139(10) -0.0908(10) -
0.0306(10)
 C87 0.1375(11) 0.0720(11) 0.1326(11) -0.0139(10) -0.0909(10) -
0.0306(10)
 C88 0.090(7) 0.079(6) 0.086(7) -0.040(5) 0.033(6) -0.041(6)
 C89 0.262(17) 0.123(10) 0.085(9) -0.043(8) 0.080(10) -0.074(12)
 C90 0.286(18) 0.118(10) 0.139(12) -0.034(9) 0.128(12) -0.110(12)
 C91 0.194(13) 0.094(7) 0.108(9) -0.037(6) 0.076(8) -0.105(9)
 C92 0.059(5) 0.047(5) 0.076(6) 0.019(4) -0.026(5) -0.021(4)
 C93 0.090(6) 0.048(5) 0.060(6) 0.014(4) -0.012(5) -0.033(4)
 C94 0.085(6) 0.077(6) 0.056(5) 0.018(4) -0.023(5) -0.058(5)
 C95 0.043(4) 0.051(4) 0.051(5) 0.013(4) -0.002(3) -0.024(3)
 C96 0.0983(11) 0.0896(11) 0.1157(11) -0.0566(10) -0.0526(10)
0.0052(10)
 C97 0.0983(11) 0.0896(11) 0.1157(11) -0.0566(10) -0.0526(10)
0.0052(10)
 C98 0.0983(11) 0.0896(11) 0.1157(11) -0.0566(10) -0.0526(10)
0.0052(10)
 C99 0.0983(11) 0.0896(11) 0.1157(11) -0.0567(10) -0.0526(10)
0.0052(10)
 C100 0.1434(12) 0.1336(12) 0.0938(12) 0.0211(11) 0.0111(12) -
0.0272(12)
 C101 0.1434(12) 0.1336(12) 0.0938(12) 0.0211(11) 0.0111(12) -
0.0272(12)
 C102 0.1434(12) 0.1336(12) 0.0938(12) 0.0211(11) 0.0111(12) -
0.0272(12)
 C103 0.1434(12) 0.1336(12) 0.0938(12) 0.0211(11) 0.0111(12) -
0.0272(12)
 C104 0.125(9) 0.162(13) 0.103(10) -0.032(8) -0.030(8) -0.069(9)
 C107 0.135(10) 0.257(16) 0.082(9) -0.058(10) 0.005(7) -0.115(11)
 C105 0.273(19) 0.145(14) 0.099(11) 0.022(8) -0.068(12) -0.041(13)
 C106 0.29(2) 0.185(14) 0.148(16) -0.025(10) 0.060(13) -0.172(15)

_geom_special_details
;
 All esds (except the esd in the dihedral angle between two l.s.
planes)
 are estimated using the full covariance matrix.  The cell esds are
taken
 into account individually in the estimation of esds in distances,
angles
 and torsion angles; correlations between esds in cell parameters
are only
 used when they are defined by crystal symmetry.  An approximate
(isotropic)
 treatment of cell esds is used for estimating esds involving l.s.
planes.
```

```
;
loop_
  _geom_bond_atom_site_label_1
  _geom_bond_atom_site_label_2
  _geom_bond_distance
  _geom_bond_site_symmetry_2
  _geom_bond_publ_flag
 Mo1 H 1.80(6) . ?
 Mo1 HA 1.82(6) . ?
 Mo1 HB 1.77(6) . ?
 Mo1 Mo2 2.1019(8) . ?
 Mo1 Li1 2.992(12) . ?
 Mo1 Li2 3.064(12) . ?
 Mo1 Li6 3.133(11) . ?
 Mo1 Li7 3.180(16) . ?
 Mo1 Li9 3.176(11) . ?
 Mo1 N1 2.129(5) . ?
 Mo2 HC 2.01(6) . ?
 Mo2 HD 1.92(6) . ?
 Mo2 HE 1.71(6) . ?
 Mo2 Li1 3.046(12) . ?
 Mo2 Li5 3.070(10) . ?
 Mo2 Li6 3.053(11) . ?
 Mo2 Li9 3.205(11) . ?
 Mo2 N2 2.135(5) . ?
 Mo3 HF 1.93(6) . ?
 Mo3 HG 1.90(6) . ?
 Mo3 HH 1.92(6) . ?
 Mo3 Mo4 2.1019(8) . ?
 Mo3 Li2 3.049(12) . ?
 Mo3 Li3 3.035(13) . ?
 Mo3 Li4 3.057(11) . ?
 Mo3 Li9 3.210(12) . ?
 Mo3 N3 2.136(5) . ?
 Mo4 HI 1.79(6) . ?
 Mo4 HJ 1.74(6) . ?
 Mo4 HK 1.80(7) . ?
 Mo4 Li1 3.105(12) . ?
 Mo4 Li2 3.020(11) . ?
 Mo4 Li3 3.123(12) . ?
 Mo4 Li8 3.06(2) . ?
 Mo4 Li9 3.159(10) . ?
 Mo4 N4 2.112(5) . ?
 Mo5 HL 1.87(7) . ?
 Mo5 HM 1.69(7) . ?
 Mo5 HN 1.98(6) . ?
 Mo5 Mo6 2.0990(8) . ?
 Mo5 Li3 3.045(11) . ?
 Mo5 Li4 3.023(12) . ?
 Mo5 Li5 3.043(11) . ?
 Mo5 Li9 3.227(11) . ?
 Mo5 N5 2.129(5) . ?
 Mo6 HO 1.76(6) . ?
 Mo6 HP 1.72(6) . ?
 Mo6 HQ 1.83(6) . ?
 Mo6 Li4 3.075(11) . ?
 Mo6 Li5 2.996(12) . ?
 Mo6 Li6 3.037(11) . ?
```

Mo6 Li7 3.369(16) . ?
Mo6 Li9 3.206(11) . ?
Mo6 N6 2.143(5) . ?
Li1 H 1.92(6) . ?
Li1 HC 2.23(7) . ?
Li1 HJ 2.12(7) . ?
Li1 HK 2.01(7) . ?
Li1 Li2 2.845(19) . ?
Li1 Li8 3.30(3) . ?
Li1 Li9 3.293(17) . ?
Li1 O1 1.927(14) . ?
Li2 H 2.20(7) . ?
Li2 HA 2.05(6) . ?
Li2 HF 1.92(6) . ?
Li2 HJ 1.94(7) . ?
Li2 Li7 3.32(2) . ?
Li2 Li9 3.313(16) . ?
Li2 O2 1.974(13) . ?
Li3 HH 1.80(6) . ?
Li3 HI 1.98(7) . ?
Li3 HM 2.25(6) . ?
Li3 HN 2.09(7) . ?
Li3 Li4 2.835(17) . ?
Li3 Li8 2.98(3) . ?
Li3 Li9 3.337(16) . ?
Li3 O3 1.957(14) . ?
Li4 HG 2.12(6) . ?
Li4 HH 2.01(6) . ?
Li4 HM 2.12(7) . ?
Li4 HQ 2.19(6) . ?
Li4 Li7 3.32(2) . ?
Li4 Li9 3.261(17) . ?
Li4 O4 1.992(13) . ?
Li5 HD 2.11(6) . ?
Li5 HE 2.04(6) . ?
Li5 HL 2.18(6) . ?
Li5 HO 2.00(6) . ?
Li5 Li6 2.896(16) . ?
Li5 Li8 3.44(2) . ?
Li5 Li9 3.156(16) . ?
Li5 O5 1.969(12) . ?
Li6 HB 1.92(6) . ?
Li6 HE 1.93(6) . ?
Li6 HO 2.15(6) . ?
Li6 HP 2.15(6) . ?
Li6 Li7 2.99(2) . ?
Li6 Li9 3.351(15) . ?
Li6 O6 1.930(13) . ?
Li7 HA 1.79(6) . ?
Li7 HG 1.95(6) . ?
Li7 HP 1.79(6) . ?
Li7 Li9 2.451(19) . ?
Li7 O7 1.897(17) . ?
Li8 HD 2.11(7) . ?
Li8 HI 2.24(7) . ?
Li8 HK 1.85(7) . ?
Li8 HN 1.73(7) . ?
Li8 Li9 2.50(2) . ?

Li8 O8 1.902(19) . ?
Li9 HA 1.95(6) . ?
Li9 HD 1.89(7) . ?
Li9 HG 1.99(7) . ?
Li9 HK 1.91(6) . ?
Li9 HN 1.90(6) . ?
Li9 HP 2.04(6) . ?
N1 C1 1.308(8) . ?
N1 C2 1.443(7) . ?
N2 C1 1.329(8) . ?
N2 C14 1.412(8) . ?
N3 C26 1.322(7) . ?
N3 C27 1.430(7) . ?
N4 C26 1.321(8) . ?
N4 C39 1.418(8) . ?
N5 C51 1.337(8) . ?
N5 C52 1.429(8) . ?
N6 C51 1.318(8) . ?
N6 C64 1.416(7) . ?
O1 C76 1.375(12) . ?
O1 C79 1.379(13) . ?
O2 C80 1.412(10) . ?
O2 C83 1.397(10) . ?
O3 C84 1.395(11) . ?
O3 C87 1.406(11) . ?
O4 C88 1.419(10) . ?
O4 C91 1.385(11) . ?
O5 C92 1.416(8) . ?
O5 C95 1.441(8) . ?
O6 C96 1.388(11) . ?
O6 C99 1.372(11) . ?
O7 C100 1.280(14) . ?
O7 C103 1.352(15) . ?
O8 C104 1.366(14) . ?
O8 C107 1.413(15) . ?
C1 H1 0.9500 . ?
C2 C3 1.403(9) . ?
C2 C7 1.400(10) . ?
C3 C4 1.440(10) . ?
C3 C8 1.492(11) . ?
C4 H4 0.9500 . ?
C4 C5 1.342(12) . ?
C5 H5 0.9500 . ?
C5 C6 1.368(12) . ?
C6 H6 0.9500 . ?
C6 C7 1.398(9) . ?
C7 C11 1.509(10) . ?
C8 H8 1.0000 . ?
C8 C9 1.521(12) . ?
C8 C10 1.544(11) . ?
C9 H9A 0.9800 . ?
C9 H9B 0.9800 . ?
C9 H9C 0.9800 . ?
C10 H10A 0.9800 . ?
C10 H10B 0.9800 . ?
C10 H10C 0.9800 . ?
C11 H11 1.0000 . ?
C11 C12 1.518(12) . ?

C11 C13 1.513(12) . ?
C12 H12A 0.9800 . ?
C12 H12B 0.9800 . ?
C12 H12C 0.9800 . ?
C13 H13A 0.9800 . ?
C13 H13B 0.9800 . ?
C13 H13C 0.9800 . ?
C14 C15 1.408(10) . ?
C14 C19 1.401(9) . ?
C15 C16 1.410(11) . ?
C15 C20 1.510(11) . ?
C16 H16 0.9500 . ?
C16 C17 1.367(12) . ?
C17 H17 0.9500 . ?
C17 C18 1.369(12) . ?
C18 H18 0.9500 . ?
C18 C19 1.392(10) . ?
C19 C23 1.506(10) . ?
C20 H20 1.0000 . ?
C20 C21 1.556(14) . ?
C20 C22 1.510(14) . ?
C21 H21A 0.9800 . ?
C21 H21B 0.9800 . ?
C21 H21C 0.9800 . ?
C22 H22A 0.9800 . ?
C22 H22B 0.9800 . ?
C22 H22C 0.9800 . ?
C23 H23 1.0000 . ?
C23 C24 1.510(11) . ?
C23 C25 1.539(11) . ?
C24 H24A 0.9800 . ?
C24 H24B 0.9800 . ?
C24 H24C 0.9800 . ?
C25 H25A 0.9800 . ?
C25 H25B 0.9800 . ?
C25 H25C 0.9800 . ?
C26 H26 0.9500 . ?
C27 C28 1.390(9) . ?
C27 C32 1.407(9) . ?
C28 C29 1.392(9) . ?
C28 C33 1.517(10) . ?
C29 H29 0.9500 . ?
C29 C30 1.380(11) . ?
C30 H30 0.9500 . ?
C30 C31 1.365(11) . ?
C31 H31 0.9500 . ?
C31 C32 1.403(9) . ?
C32 C36 1.528(10) . ?
C33 H33 1.0000 . ?
C33 C34 1.507(12) . ?
C33 C35 1.503(13) . ?
C34 H34A 0.9800 . ?
C34 H34B 0.9800 . ?
C34 H34C 0.9800 . ?
C35 H35A 0.9800 . ?
C35 H35B 0.9800 . ?
C35 H35C 0.9800 . ?
C36 H36 1.0000 . ?

C36 C37 1.519(11) . ?
C36 C38 1.508(11) . ?
C37 H37A 0.9800 . ?
C37 H37B 0.9800 . ?
C37 H37C 0.9800 . ?
C38 H38A 0.9800 . ?
C38 H38B 0.9800 . ?
C38 H38C 0.9800 . ?
C39 C40 1.399(11) . ?
C39 C44 1.409(11) . ?
C40 C41 1.395(11) . ?
C40 C45 1.530(13) . ?
C41 H41 0.9500 . ?
C41 C42 1.353(16) . ?
C42 H42 0.9500 . ?
C42 C43 1.371(15) . ?
C43 H43 0.9500 . ?
C43 C44 1.409(10) . ?
C44 C48 1.515(13) . ?
C45 H45 1.0000 . ?
C45 C46 1.528(13) . ?
C45 C47 1.520(13) . ?
C46 H46A 0.9800 . ?
C46 H46B 0.9800 . ?
C46 H46C 0.9800 . ?
C47 H47A 0.9800 . ?
C47 H47B 0.9800 . ?
C47 H47C 0.9800 . ?
C48 H48 1.0000 . ?
C48 C49 1.557(14) . ?
C48 C50 1.518(14) . ?
C49 H49A 0.9800 . ?
C49 H49B 0.9800 . ?
C49 H49C 0.9800 . ?
C50 H50A 0.9800 . ?
C50 H50B 0.9800 . ?
C50 H50C 0.9800 . ?
C51 H51 0.9500 . ?
C52 C53 1.392(11) . ?
C52 C57 1.392(10) . ?
C53 C54 1.394(12) . ?
C53 C58 1.532(13) . ?
C54 H54 0.9500 . ?
C54 C55 1.378(15) . ?
C55 H55 0.9500 . ?
C55 C56 1.367(14) . ?
C56 H56 0.9500 . ?
C56 C57 1.390(10) . ?
C57 C61 1.506(11) . ?
C58 H58 1.0000 . ?
C58 C59 1.515(14) . ?
C58 C60 1.543(13) . ?
C59 H59A 0.9800 . ?
C59 H59B 0.9800 . ?
C59 H59C 0.9800 . ?
C60 H60A 0.9800 . ?
C60 H60B 0.9800 . ?
C60 H60C 0.9800 . ?

C61 H61 1.0000 . ?
C61 C62 1.521(11) . ?
C61 C63 1.550(11) . ?
C62 H62A 0.9800 . ?
C62 H62B 0.9800 . ?
C62 H62C 0.9800 . ?
C63 H63A 0.9800 . ?
C63 H63B 0.9800 . ?
C63 H63C 0.9800 . ?
C64 C65 1.404(10) . ?
C64 C69 1.417(9) . ?
C65 C66 1.402(10) . ?
C65 C70 1.499(11) . ?
C66 H66 0.9500 . ?
C66 C67 1.355(12) . ?
C67 H67 0.9500 . ?
C67 C68 1.384(12) . ?
C68 H68 0.9500 . ?
C68 C69 1.384(10) . ?
C69 C73 1.505(11) . ?
C70 H70 1.0000 . ?
C70 C71 1.555(13) . ?
C70 C72 1.553(12) . ?
C71 H71A 0.9800 . ?
C71 H71B 0.9800 . ?
C71 H71C 0.9800 . ?
C72 H72A 0.9800 . ?
C72 H72B 0.9800 . ?
C72 H72C 0.9800 . ?
C73 H73 1.0000 . ?
C73 C74 1.534(10) . ?
C73 C75 1.522(11) . ?
C74 H74A 0.9800 . ?
C74 H74B 0.9800 . ?
C74 H74C 0.9800 . ?
C75 H75A 0.9800 . ?
C75 H75B 0.9800 . ?
C75 H75C 0.9800 . ?
C76 H76A 0.9900 . ?
C76 H76B 0.9900 . ?
C76 C77 1.467(13) . ?
C77 H77A 0.9900 . ?
C77 H77B 0.9900 . ?
C77 C78 1.498(14) . ?
C78 H78A 0.9900 . ?
C78 H78B 0.9900 . ?
C78 C79 1.455(13) . ?
C79 H79A 0.9900 . ?
C79 H79B 0.9900 . ?
C80 H80A 0.9900 . ?
C80 H80B 0.9900 . ?
C80 C81 1.474(12) . ?
C81 H81A 0.9900 . ?
C81 H81B 0.9900 . ?
C81 C82 1.477(13) . ?
C82 H82A 0.9900 . ?
C82 H82B 0.9900 . ?
C82 C83 1.460(12) . ?

C83 H83A 0.9900 . ?
C83 H83B 0.9900 . ?
C84 H84A 0.9900 . ?
C84 H84B 0.9900 . ?
C84 C85 1.402(10) . ?
C85 H85A 0.9900 . ?
C85 H85B 0.9900 . ?
C85 C86 1.492(11) . ?
C86 H86A 0.9900 . ?
C86 H86B 0.9900 . ?
C86 C87 1.460(11) . ?
C87 H87A 0.9900 . ?
C87 H87B 0.9900 . ?
C88 H88A 0.9900 . ?
C88 H88B 0.9900 . ?
C88 C89 1.416(12) . ?
C89 H89A 0.9900 . ?
C89 H89B 0.9900 . ?
C89 C90 1.465(14) . ?
C90 H90A 0.9900 . ?
C90 H90B 0.9900 . ?
C90 C91 1.451(12) . ?
C91 H91A 0.9900 . ?
C91 H91B 0.9900 . ?
C92 H92A 0.9900 . ?
C92 H92B 0.9900 . ?
C92 C93 1.522(10) . ?
C93 H93A 0.9900 . ?
C93 H93B 0.9900 . ?
C93 C94 1.493(10) . ?
C94 H94A 0.9900 . ?
C94 H94B 0.9900 . ?
C94 C95 1.515(9) . ?
C95 H95A 0.9900 . ?
C95 H95B 0.9900 . ?
C96 H96A 0.9900 . ?
C96 H96B 0.9900 . ?
C96 C97 1.494(12) . ?
C97 H97A 0.9900 . ?
C97 H97B 0.9900 . ?
C97 C98 1.477(11) . ?
C98 H98A 0.9900 . ?
C98 H98B 0.9900 . ?
C98 C99 1.453(11) . ?
C99 H99A 0.9900 . ?
C99 H99B 0.9900 . ?
C100 H10D 0.9900 . ?
C100 H10E 0.9900 . ?
C100 C101 1.508(14) . ?
C101 H10F 0.9900 . ?
C101 H10G 0.9900 . ?
C101 C102 1.479(13) . ?
C102 H10H 0.9900 . ?
C102 H10I 0.9900 . ?
C102 C103 1.367(14) . ?
C103 H10J 0.9900 . ?
C103 H10K 0.9900 . ?
C104 H10L 0.9900 . ?

```
 C104 H10M 0.9900 . ?
 C104 C105 1.472(15) . ?
 C107 H10N 0.9900 . ?
 C107 H10O 0.9900 . ?
 C107 C106 1.460(15) . ?
 C105 H10P 0.9900 . ?
 C105 H10Q 0.9900 . ?
 C105 C106 1.443(16) . ?
 C106 H10R 0.9900 . ?
 C106 H10S 0.9900 . ?

loop_
  _geom_angle_atom_site_label_1
  _geom_angle_atom_site_label_2
  _geom_angle_atom_site_label_3
  _geom_angle
  _geom_angle_site_symmetry_1
  _geom_angle_site_symmetry_3
  _geom_angle_publ_flag
 H Mo1 HA 83(3) . . ?
 H Mo1 HB 151(3) . . ?
 HA Mo1 HB 76(3) . . ?
 Mo2 Mo1 H 104(2) . . ?
 Mo2 Mo1 HA 105(2) . . ?
 Mo2 Mo1 HB 101(2) . . ?
 Mo2 Mo1 Li1 71.0(3) . . ?
 Mo2 Mo1 Li2 121.1(2) . . ?
 Mo2 Mo1 Li6 68.1(2) . . ?
 Mo2 Mo1 Li7 107.7(3) . . ?
 Mo2 Mo1 Li9 71.5(2) . . ?
 Mo2 Mo1 N1 92.48(14) . . ?
 Li1 Mo1 H 38(2) . . ?
 Li1 Mo1 HA 74(2) . . ?
 Li1 Mo1 HB 146(2) . . ?
 Li1 Mo1 Li2 56.0(3) . . ?
 Li1 Mo1 Li6 121.6(3) . . ?
 Li1 Mo1 Li7 101.4(4) . . ?
 Li1 Mo1 Li9 64.5(3) . . ?
 Li2 Mo1 H 45(2) . . ?
 Li2 Mo1 HA 40(2) . . ?
 Li2 Mo1 HB 108(2) . . ?
 Li2 Mo1 Li6 118.7(3) . . ?
 Li2 Mo1 Li7 64.2(4) . . ?
 Li2 Mo1 Li9 64.1(3) . . ?
 Li6 Mo1 H 156(2) . . ?
 Li6 Mo1 HA 78(2) . . ?
 Li6 Mo1 HB 34(2) . . ?
 Li6 Mo1 Li7 56.5(4) . . ?
 Li6 Mo1 Li9 64.2(3) . . ?
 Li7 Mo1 H 109(2) . . ?
 Li7 Mo1 HA 28(2) . . ?
 Li7 Mo1 HB 48(2) . . ?
 Li9 Mo1 H 92(2) . . ?
 Li9 Mo1 HA 34(2) . . ?
 Li9 Mo1 HB 81(2) . . ?
 Li9 Mo1 Li7 45.4(4) . . ?
 N1 Mo1 H 92(2) . . ?
 N1 Mo1 HA 162(2) . . ?
```

N1 Mo1 HB 102(2) . . ?
N1 Mo1 Li1 111.7(3) . . ?
N1 Mo1 Li2 128.1(3) . . ?
N1 Mo1 Li6 109.9(2) . . ?
N1 Mo1 Li7 145.5(3) . . ?
N1 Mo1 Li9 164.0(3) . . ?
Mo1 Mo2 HC 109.2(18) . . ?
Mo1 Mo2 HD 102.1(19) . . ?
Mo1 Mo2 HE 107(2) . . ?
Mo1 Mo2 Li1 68.3(3) . . ?
Mo1 Mo2 Li5 119.2(2) . . ?
Mo1 Mo2 Li6 72.2(2) . . ?
Mo1 Mo2 Li9 70.0(2) . . ?
Mo1 Mo2 N2 92.35(14) . . ?
HC Mo2 HD 82(3) . . ?
HC Mo2 HE 142(3) . . ?
HD Mo2 HE 79(3) . . ?
Li1 Mo2 HC 46.9(19) . . ?
Li1 Mo2 HD 70.1(19) . . ?
Li1 Mo2 HE 147(2) . . ?
Li1 Mo2 Li5 112.9(3) . . ?
Li1 Mo2 Li6 122.5(3) . . ?
Li1 Mo2 Li9 63.5(3) . . ?
Li5 Mo2 HC 110.6(18) . . ?
Li5 Mo2 HD 42.8(19) . . ?
Li5 Mo2 HE 39(2) . . ?
Li5 Mo2 Li9 60.4(3) . . ?
Li6 Mo2 HC 161.8(18) . . ?
Li6 Mo2 HD 79.6(19) . . ?
Li6 Mo2 HE 35(2) . . ?
Li6 Mo2 Li5 56.5(3) . . ?
Li6 Mo2 Li9 64.7(3) . . ?
Li9 Mo2 HC 98.2(18) . . ?
Li9 Mo2 HD 32.6(19) . . ?
Li9 Mo2 HE 84(2) . . ?
N2 Mo2 HC 91.9(18) . . ?
N2 Mo2 HD 165.5(19) . . ?
N2 Mo2 HE 98(2) . . ?
N2 Mo2 Li1 115.2(3) . . ?
N2 Mo2 Li5 129.6(2) . . ?
N2 Mo2 Li6 106.3(2) . . ?
N2 Mo2 Li9 161.8(3) . . ?
HF Mo3 HG 78(3) . . ?
HF Mo3 HH 149(3) . . ?
HG Mo3 HH 81(3) . . ?
Mo4 Mo3 HF 102.3(19) . . ?
Mo4 Mo3 HG 104.3(19) . . ?
Mo4 Mo3 HH 104.8(19) . . ?
Mo4 Mo3 Li2 69.0(2) . . ?
Mo4 Mo3 Li3 72.3(2) . . ?
Mo4 Mo3 Li4 119.7(2) . . ?
Mo4 Mo3 Li9 69.4(2) . . ?
Mo4 Mo3 N3 92.07(13) . . ?
Li2 Mo3 HF 37.4(19) . . ?
Li2 Mo3 HG 71.9(19) . . ?
Li2 Mo3 HH 149.1(19) . . ?
Li2 Mo3 Li4 115.2(3) . . ?
Li2 Mo3 Li9 63.9(3) . . ?

Li3 Mo3 HF 155.2(19) . . ?
Li3 Mo3 HG 79.6(19) . . ?
Li3 Mo3 HH 34.1(19) . . ?
Li3 Mo3 Li2 123.3(3) . . ?
Li3 Mo3 Li4 55.5(3) . . ?
Li3 Mo3 Li9 64.5(3) . . ?
Li4 Mo3 HF 112.4(19) . . ?
Li4 Mo3 HG 43.3(19) . . ?
Li4 Mo3 HH 39.9(18) . . ?
Li4 Mo3 Li9 62.7(3) . . ?
Li9 Mo3 HF 90.8(19) . . ?
Li9 Mo3 HG 35.3(19) . . ?
Li9 Mo3 HH 85.5(19) . . ?
N3 Mo3 HF 96.8(19) . . ?
N3 Mo3 HG 163.5(19) . . ?
N3 Mo3 HH 96.4(19) . . ?
N3 Mo3 Li2 113.8(3) . . ?
N3 Mo3 Li3 107.4(3) . . ?
N3 Mo3 Li4 128.5(3) . . ?
N3 Mo3 Li9 161.2(2) . . ?
Mo3 Mo4 HI 103(2) . . ?
Mo3 Mo4 HJ 104(2) . . ?
Mo3 Mo4 HK 104(2) . . ?
Mo3 Mo4 Li1 119.9(2) . . ?
Mo3 Mo4 Li2 70.5(2) . . ?
Mo3 Mo4 Li3 67.8(2) . . ?
Mo3 Mo4 Li8 110.1(3) . . ?
Mo3 Mo4 Li9 72.0(2) . . ?
Mo3 Mo4 N4 92.75(14) . . ?
HI Mo4 HJ 147(3) . . ?
HI Mo4 HK 80(3) . . ?
HJ Mo4 HK 75(3) . . ?
Li1 Mo4 HI 108(2) . . ?
Li1 Mo4 HJ 41(2) . . ?
Li1 Mo4 HK 38(2) . . ?
Li1 Mo4 Li3 119.2(3) . . ?
Li1 Mo4 Li9 63.4(3) . . ?
Li2 Mo4 HI 147(2) . . ?
Li2 Mo4 HJ 37(2) . . ?
Li2 Mo4 HK 70(2) . . ?
Li2 Mo4 Li1 55.3(4) . . ?
Li2 Mo4 Li3 121.3(3) . . ?
Li2 Mo4 Li8 103.4(5) . . ?
Li2 Mo4 Li9 64.8(3) . . ?
Li3 Mo4 HI 36(2) . . ?
Li3 Mo4 HJ 153(2) . . ?
Li3 Mo4 HK 82(2) . . ?
Li3 Mo4 Li9 64.2(3) . . ?
Li8 Mo4 HI 47(2) . . ?
Li8 Mo4 HJ 106(2) . . ?
Li8 Mo4 HK 34(2) . . ?
Li8 Mo4 Li1 64.8(6) . . ?
Li8 Mo4 Li3 57.6(5) . . ?
Li8 Mo4 Li9 47.4(4) . . ?
Li9 Mo4 HI 82(2) . . ?
Li9 Mo4 HJ 89(2) . . ?
Li9 Mo4 HK 33(2) . . ?
N4 Mo4 HI 101(2) . . ?

N4 Mo4 HJ 96(2) . . ?
N4 Mo4 HK 162(2) . . ?
N4 Mo4 Li1 128.3(3) . . ?
N4 Mo4 Li2 111.9(3) . . ?
N4 Mo4 Li3 109.6(3) . . ?
N4 Mo4 Li8 142.8(5) . . ?
N4 Mo4 Li9 164.7(3) . . ?
HL Mo5 HM 143(3) . . ?
HL Mo5 HN 80(3) . . ?
HM Mo5 HN 88(3) . . ?
Mo6 Mo5 HL 109.1(19) . . ?
Mo6 Mo5 HM 107(2) . . ?
Mo6 Mo5 HN 103.4(18) . . ?
Mo6 Mo5 Li3 121.1(2) . . ?
Mo6 Mo5 Li4 71.2(2) . . ?
Mo6 Mo5 Li5 68.5(2) . . ?
Mo6 Mo5 Li9 70.43(19) . . ?
Mo6 Mo5 N5 92.61(14) . . ?
Li3 Mo5 HL 108.0(19) . . ?
Li3 Mo5 HM 46(2) . . ?
Li3 Mo5 HN 42.9(19) . . ?
Li3 Mo5 Li9 64.2(3) . . ?
Li4 Mo5 HL 153.6(19) . . ?
Li4 Mo5 HM 43(2) . . ?
Li4 Mo5 HN 74.8(19) . . ?
Li4 Mo5 Li3 55.7(3) . . ?
Li4 Mo5 Li5 118.0(3) . . ?
Li4 Mo5 Li9 62.8(3) . . ?
Li5 Mo5 HL 45.2(19) . . ?
Li5 Mo5 HM 156(2) . . ?
Li5 Mo5 HN 71.3(19) . . ?
Li5 Mo5 Li3 114.2(3) . . ?
Li5 Mo5 Li9 60.4(3) . . ?
Li9 Mo5 HL 91.9(19) . . ?
Li9 Mo5 HM 96(2) . . ?
Li9 Mo5 HN 33.0(18) . . ?
N5 Mo5 HL 96.2(19) . . ?
N5 Mo5 HM 87(2) . . ?
N5 Mo5 HN 164.0(18) . . ?
N5 Mo5 Li3 126.6(3) . . ?
N5 Mo5 Li4 110.2(3) . . ?
N5 Mo5 Li5 116.6(2) . . ?
N5 Mo5 Li9 162.9(2) . . ?
Mo5 Mo6 HO 105(2) . . ?
Mo5 Mo6 HP 106(2) . . ?
Mo5 Mo6 HQ 109(2) . . ?
Mo5 Mo6 Li4 68.5(3) . . ?
Mo5 Mo6 Li5 70.9(2) . . ?
Mo5 Mo6 Li6 123.4(2) . . ?
Mo5 Mo6 Li7 110.3(3) . . ?
Mo5 Mo6 Li9 71.5(2) . . ?
Mo5 Mo6 N6 92.52(14) . . ?
HO Mo6 HP 85(3) . . ?
HO Mo6 HQ 145(3) . . ?
HP Mo6 HQ 79(3) . . ?
Li4 Mo6 HO 153(2) . . ?
Li4 Mo6 HP 72(2) . . ?
Li4 Mo6 HQ 45(2) . . ?

Li4 Mo6 Li7 61.8(4) . . ?
Li4 Mo6 Li9 62.5(3) . . ?
Li5 Mo6 HO 40(2) . . ?
Li5 Mo6 HP 76(2) . . ?
Li5 Mo6 HQ 154(2) . . ?
Li5 Mo6 Li4 117.9(3) . . ?
Li5 Mo6 Li6 57.4(3) . . ?
Li5 Mo6 Li7 92.4(3) . . ?
Li5 Mo6 Li9 61.1(3) . . ?
Li6 Mo6 HO 44(2) . . ?
Li6 Mo6 HP 44(2) . . ?
Li6 Mo6 HQ 108(2) . . ?
Li6 Mo6 Li4 116.0(3) . . ?
Li6 Mo6 Li7 55.3(4) . . ?
Li6 Mo6 Li9 64.9(3) . . ?
Li7 Mo6 HO 99(2) . . ?
Li7 Mo6 HP 17(2) . . ?
Li7 Mo6 HQ 62(2) . . ?
Li9 Mo6 HO 90(2) . . ?
Li9 Mo6 HP 35(2) . . ?
Li9 Mo6 HQ 93(2) . . ?
Li9 Mo6 Li7 43.7(3) . . ?
N6 Mo6 HO 92(2) . . ?
N6 Mo6 HP 161(2) . . ?
N6 Mo6 HQ 94(2) . . ?
N6 Mo6 Li4 114.0(3) . . ?
N6 Mo6 Li5 112.8(2) . . ?
N6 Mo6 Li6 126.2(2) . . ?
N6 Mo6 Li7 150.8(3) . . ?
N6 Mo6 Li9 163.9(2) . . ?
Mo1 Li1 H 35.1(19) . . ?
Mo1 Li1 Mo2 40.73(16) . . ?
Mo1 Li1 HC 78.5(16) . . ?
Mo1 Li1 Mo4 109.5(4) . . ?
Mo1 Li1 HJ 105.7(18) . . ?
Mo1 Li1 HK 92.0(19) . . ?
Mo1 Li1 Li8 98.7(5) . . ?
Mo1 Li1 Li9 60.5(3) . . ?
H Li1 HC 101(3) . . ?
H Li1 HJ 89(3) . . ?
H Li1 HK 112(3) . . ?
Mo2 Li1 H 72.5(19) . . ?
Mo2 Li1 HC 41.3(16) . . ?
Mo2 Li1 Mo4 119.1(4) . . ?
Mo2 Li1 HJ 136.6(18) . . ?
Mo2 Li1 HK 87.3(19) . . ?
Mo2 Li1 Li8 74.9(5) . . ?
Mo2 Li1 Li9 60.6(3) . . ?
HC Li1 HJ 166(2) . . ?
HC Li1 HK 104(3) . . ?
Mo4 Li1 H 111(2) . . ?
Mo4 Li1 HC 133.6(17) . . ?
Mo4 Li1 HJ 32.6(17) . . ?
Mo4 Li1 HK 33.1(19) . . ?
Mo4 Li1 Li8 56.9(5) . . ?
Mo4 Li1 Li9 59.1(3) . . ?
HJ Li1 HK 63(3) . . ?
Li2 Li1 Mo1 63.3(4) . . ?

Li2 Li1 H 51(2) . . ?
Li2 Li1 Mo2 100.2(4) . . ?
Li2 Li1 HC 141.1(17) . . ?
Li2 Li1 Mo4 60.8(3) . . ?
Li2 Li1 HJ 42.9(18) . . ?
Li2 Li1 HK 73(2) . . ?
Li2 Li1 Li8 101.5(6) . . ?
Li2 Li1 Li9 64.9(4) . . ?
Li8 Li1 H 130(2) . . ?
Li8 Li1 HC 76.9(17) . . ?
Li8 Li1 HJ 89.4(18) . . ?
Li8 Li1 HK 29.8(19) . . ?
Li9 Li1 H 86.6(19) . . ?
Li9 Li1 HC 91.4(17) . . ?
Li9 Li1 HJ 79.7(18) . . ?
Li9 Li1 HK 31.9(18) . . ?
Li9 Li1 Li8 44.5(4) . . ?
O1 Li1 Mo1 150.7(6) . . ?
O1 Li1 H 126(2) . . ?
O1 Li1 Mo2 128.9(6) . . ?
O1 Li1 HC 87.8(17) . . ?
O1 Li1 Mo4 98.7(5) . . ?
O1 Li1 HJ 93.8(18) . . ?
O1 Li1 HK 117(2) . . ?
O1 Li1 Li2 129.1(7) . . ?
O1 Li1 Li8 103.2(7) . . ?
O1 Li1 Li9 146.6(7) . . ?
Mo1 Li2 H 35.3(16) . . ?
Mo1 Li2 HA 35.2(18) . . ?
Mo1 Li2 HF 131.7(19) . . ?
Mo1 Li2 HJ 108(2) . . ?
Mo1 Li2 Li7 59.6(4) . . ?
Mo1 Li2 Li9 59.6(3) . . ?
H Li2 HA 69(2) . . ?
H Li2 HF 167(3) . . ?
H Li2 HJ 87(3) . . ?
HA Li2 HF 99(3) . . ?
HA Li2 HJ 110(3) . . ?
Mo3 Li2 Mo1 119.2(4) . . ?
Mo3 Li2 H 138.3(17) . . ?
Mo3 Li2 HA 85.9(18) . . ?
Mo3 Li2 HF 37.7(19) . . ?
Mo3 Li2 HJ 71.4(19) . . ?
Mo3 Li2 Li7 70.9(4) . . ?
Mo3 Li2 Li9 60.4(3) . . ?
HF Li2 HJ 100(3) . . ?
Mo4 Li2 Mo1 109.9(4) . . ?
Mo4 Li2 H 106.2(17) . . ?
Mo4 Li2 HA 92.6(18) . . ?
Mo4 Li2 Mo3 40.52(15) . . ?
Mo4 Li2 HF 75.4(19) . . ?
Mo4 Li2 HJ 32.8(19) . . ?
Mo4 Li2 Li7 95.0(4) . . ?
Mo4 Li2 Li9 59.6(3) . . ?
Li1 Li2 Mo1 60.7(3) . . ?
Li1 Li2 H 42.5(17) . . ?
Li1 Li2 HA 75.3(19) . . ?
Li1 Li2 Mo3 100.6(4) . . ?

Li1 Li2 HF 138.1(19) . . ?
Li1 Li2 Mo4 63.9(3) . . ?
Li1 Li2 HJ 48(2) . . ?
Li1 Li2 Li7 101.3(5) . . ?
Li1 Li2 Li9 64.1(4) . . ?
Li7 Li2 H 94.8(17) . . ?
Li7 Li2 HA 28.0(18) . . ?
Li7 Li2 HF 72.2(19) . . ?
Li7 Li2 HJ 124(2) . . ?
Li9 Li2 H 82.0(17) . . ?
Li9 Li2 HA 33.1(18) . . ?
Li9 Li2 HF 87.9(19) . . ?
Li9 Li2 HJ 81.5(19) . . ?
Li9 Li2 Li7 43.4(4) . . ?
O2 Li2 Mo1 101.6(5) . . ?
O2 Li2 H 93.9(17) . . ?
O2 Li2 HA 119.3(19) . . ?
O2 Li2 Mo3 127.7(6) . . ?
O2 Li2 HF 90.9(19) . . ?
O2 Li2 Mo4 147.0(6) . . ?
O2 Li2 HJ 127(2) . . ?
O2 Li2 Li1 128.7(6) . . ?
O2 Li2 Li7 109.3(6) . . ?
O2 Li2 Li9 151.2(6) . . ?
Mo3 Li3 HH 37(2) . . ?
Mo3 Li3 Mo4 39.89(16) . . ?
Mo3 Li3 HI 71.6(19) . . ?
Mo3 Li3 Mo5 111.3(4) . . ?
Mo3 Li3 HM 110.0(17) . . ?
Mo3 Li3 HN 91.7(18) . . ?
Mo3 Li3 Li9 60.3(3) . . ?
HH Li3 HI 105(3) . . ?
HH Li3 HM 87(3) . . ?
HH Li3 HN 112(3) . . ?
Mo4 Li3 HH 76(2) . . ?
Mo4 Li3 HI 32.3(19) . . ?
Mo4 Li3 HM 138.8(17) . . ?
Mo4 Li3 HN 80.4(18) . . ?
Mo4 Li3 Li9 58.5(3) . . ?
HI Li3 HM 153(3) . . ?
HI Li3 HN 81(3) . . ?
Mo5 Li3 HH 106(2) . . ?
Mo5 Li3 Mo4 118.2(4) . . ?
Mo5 Li3 HI 121(2) . . ?
Mo5 Li3 HM 33.0(17) . . ?
Mo5 Li3 HN 40.3(18) . . ?
Mo5 Li3 Li9 60.5(3) . . ?
HM Li3 HN 72(2) . . ?
Li4 Li3 Mo3 62.7(4) . . ?
Li4 Li3 HH 45(2) . . ?
Li4 Li3 Mo4 97.6(4) . . ?
Li4 Li3 HI 129(2) . . ?
Li4 Li3 Mo5 61.7(3) . . ?
Li4 Li3 HM 47.5(17) . . ?
Li4 Li3 HN 77.9(18) . . ?
Li4 Li3 Li8 107.8(6) . . ?
Li4 Li3 Li9 63.2(4) . . ?
Li8 Li3 Mo3 90.4(5) . . ?

Li8 Li3 HH 125(2) . . ?
Li8 Li3 Mo4 60.1(4) . . ?
Li8 Li3 HI 49(2) . . ?
Li8 Li3 Mo5 71.9(5) . . ?
Li8 Li3 HM 104.8(18) . . ?
Li8 Li3 HN 34.5(18) . . ?
Li8 Li3 Li9 46.2(5) . . ?
Li9 Li3 HH 84(2) . . ?
Li9 Li3 HI 74.9(19) . . ?
Li9 Li3 HM 83.0(17) . . ?
Li9 Li3 HN 31.5(18) . . ?
O3 Li3 Mo3 146.0(6) . . ?
O3 Li3 HH 125(2) . . ?
O3 Li3 Mo4 128.4(5) . . ?
O3 Li3 HI 99.7(19) . . ?
O3 Li3 Mo5 101.4(5) . . ?
O3 Li3 HM 92.3(17) . . ?
O3 Li3 HN 120.1(19) . . ?
O3 Li3 Li4 131.3(7) . . ?
O3 Li3 Li8 108.8(7) . . ?
O3 Li3 Li9 151.0(7) . . ?
Mo3 Li4 HG 37.9(17) . . ?
Mo3 Li4 HH 38.0(18) . . ?
Mo3 Li4 HM 113.2(18) . . ?
Mo3 Li4 Mo6 121.3(4) . . ?
Mo3 Li4 HQ 132.7(17) . . ?
Mo3 Li4 Li7 70.8(4) . . ?
Mo3 Li4 Li9 61.0(3) . . ?
HG Li4 HH 74(2) . . ?
HG Li4 HM 120(3) . . ?
HG Li4 HQ 95(2) . . ?
HH Li4 HM 85(3) . . ?
HH Li4 HQ 168(3) . . ?
Mo5 Li4 Mo3 111.3(4) . . ?
Mo5 Li4 HG 97.7(18) . . ?
Mo5 Li4 HH 101.7(19) . . ?
Mo5 Li4 HM 32.6(18) . . ?
Mo5 Li4 Mo6 40.26(15) . . ?
Mo5 Li4 HQ 73.6(17) . . ?
Mo5 Li4 Li7 91.5(4) . . ?
Mo5 Li4 Li9 61.7(3) . . ?
HM Li4 HQ 95(2) . . ?
Mo6 Li4 HG 87.7(18) . . ?
Mo6 Li4 HH 135.6(19) . . ?
Mo6 Li4 HM 69.3(18) . . ?
Mo6 Li4 HQ 35.9(17) . . ?
Mo6 Li4 Li7 63.5(4) . . ?
Mo6 Li4 Li9 60.7(3) . . ?
Li3 Li4 Mo3 61.9(3) . . ?
Li3 Li4 HG 81.4(17) . . ?
Li3 Li4 HH 39.2(19) . . ?
Li3 Li4 Mo5 62.5(4) . . ?
Li3 Li4 HM 51.5(18) . . ?
Li3 Li4 Mo6 99.2(4) . . ?
Li3 Li4 HQ 135.0(18) . . ?
Li3 Li4 Li7 107.8(5) . . ?
Li3 Li4 Li9 65.9(4) . . ?
Li7 Li4 HG 33.8(17) . . ?

Li7 Li4 HH 107.7(18) . . ?
Li7 Li4 HM 123.5(18) . . ?
Li7 Li4 HQ 62.0(17) . . ?
Li9 Li4 HG 36.1(18) . . ?
Li9 Li4 HH 82.8(18) . . ?
Li9 Li4 HM 86.9(18) . . ?
Li9 Li4 HQ 85.3(17) . . ?
Li9 Li4 Li7 43.7(4) . . ?
O4 Li4 Mo3 99.6(5) . . ?
O4 Li4 HG 113.6(19) . . ?
O4 Li4 HH 96.0(19) . . ?
O4 Li4 Mo5 147.3(6) . . ?
O4 Li4 HM 124.2(19) . . ?
O4 Li4 Mo6 128.4(5) . . ?
O4 Li4 HQ 93.5(18) . . ?
O4 Li4 Li3 129.1(6) . . ?
O4 Li4 Li7 109.1(6) . . ?
O4 Li4 Li9 148.9(7) . . ?
Mo2 Li5 HD 38.2(17) . . ?
Mo2 Li5 HE 31.7(18) . . ?
Mo2 Li5 HL 136.8(17) . . ?
Mo2 Li5 HO 109.4(18) . . ?
Mo2 Li5 Li8 72.6(5) . . ?
Mo2 Li5 Li9 61.9(3) . . ?
HD Li5 HE 68(2) . . ?
HD Li5 HL 100(2) . . ?
HD Li5 HO 119(3) . . ?
HE Li5 HL 168(2) . . ?
HE Li5 HO 86(3) . . ?
Mo5 Li5 Mo2 124.5(4) . . ?
Mo5 Li5 HD 90.5(18) . . ?
Mo5 Li5 HE 136.8(19) . . ?
Mo5 Li5 HL 37.7(17) . . ?
Mo5 Li5 HO 71.8(18) . . ?
Mo5 Li5 Li8 65.8(5) . . ?
Mo5 Li5 Li9 62.7(3) . . ?
HL Li5 HO 99(3) . . ?
Mo6 Li5 Mo2 111.6(4) . . ?
Mo6 Li5 HD 98.3(18) . . ?
Mo6 Li5 HE 103.5(18) . . ?
Mo6 Li5 Mo5 40.67(15) . . ?
Mo6 Li5 HL 75.7(17) . . ?
Mo6 Li5 HO 34.3(18) . . ?
Mo6 Li5 Li8 93.4(5) . . ?
Mo6 Li5 Li9 62.8(3) . . ?
Li6 Li5 Mo2 61.5(3) . . ?
Li6 Li5 HD 80.9(17) . . ?
Li6 Li5 HE 41.8(18) . . ?
Li6 Li5 Mo5 99.8(4) . . ?
Li6 Li5 HL 137.2(18) . . ?
Li6 Li5 Mo6 62.0(3) . . ?
Li6 Li5 HO 47.9(18) . . ?
Li6 Li5 Li8 109.5(5) . . ?
Li6 Li5 Li9 67.1(4) . . ?
Li8 Li5 HD 35.5(17) . . ?
Li8 Li5 HE 103.6(18) . . ?
Li8 Li5 HL 64.4(17) . . ?
Li8 Li5 HO 126.5(19) . . ?

Li9 Li5 HD 35.6(18) . . ?
Li9 Li5 HE 80.5(18) . . ?
Li9 Li5 HL 88.4(17) . . ?
Li9 Li5 HO 87.7(19) . . ?
Li9 Li5 Li8 44.3(4) . . ?
O5 Li5 Mo2 99.8(4) . . ?
O5 Li5 HD 116.4(18) . . ?
O5 Li5 HE 94.9(19) . . ?
O5 Li5 Mo5 128.3(5) . . ?
O5 Li5 HL 92.2(18) . . ?
O5 Li5 Mo6 144.9(5) . . ?
O5 Li5 HO 120.5(19) . . ?
O5 Li5 Li6 126.3(5) . . ?
O5 Li5 Li8 111.2(6) . . ?
O5 Li5 Li9 151.2(6) . . ?
Mo1 Li6 HB 30.6(19) . . ?
Mo1 Li6 HE 70.1(19) . . ?
Mo1 Li6 HO 135.1(17) . . ?
Mo1 Li6 HP 86.6(17) . . ?
Mo1 Li6 Li9 58.5(3) . . ?
HB Li6 HE 99(3) . . ?
HB Li6 HO 153(3) . . ?
HB Li6 HP 88(3) . . ?
Mo2 Li6 Mo1 39.70(14) . . ?
Mo2 Li6 HB 69.6(19) . . ?
Mo2 Li6 HE 30.9(19) . . ?
Mo2 Li6 HO 105.6(17) . . ?
Mo2 Li6 HP 95.5(17) . . ?
Mo2 Li6 Li9 59.8(3) . . ?
HE Li6 HO 84(3) . . ?
HE Li6 HP 107(3) . . ?
Mo6 Li6 Mo1 117.7(4) . . ?
Mo6 Li6 HB 120.9(19) . . ?
Mo6 Li6 Mo2 110.9(3) . . ?
Mo6 Li6 HE 104.9(19) . . ?
Mo6 Li6 HO 34.5(17) . . ?
Mo6 Li6 HP 33.5(17) . . ?
Mo6 Li6 Li9 60.0(3) . . ?
HO Li6 HP 66(2) . . ?
Li5 Li6 Mo1 96.1(4) . . ?
Li5 Li6 HB 125.3(19) . . ?
Li5 Li6 Mo2 62.1(3) . . ?
Li5 Li6 HE 44.6(18) . . ?
Li5 Li6 Mo6 60.6(3) . . ?
Li5 Li6 HO 43.5(17) . . ?
Li5 Li6 HP 73.4(17) . . ?
Li5 Li6 Li7 102.9(5) . . ?
Li5 Li6 Li9 60.2(4) . . ?
Li7 Li6 Mo1 62.6(4) . . ?
Li7 Li6 HB 53.1(19) . . ?
Li7 Li6 Mo2 91.1(4) . . ?
Li7 Li6 HE 117.8(19) . . ?
Li7 Li6 Mo6 68.0(4) . . ?
Li7 Li6 HO 102.0(18) . . ?
Li7 Li6 HP 36.3(17) . . ?
Li7 Li6 Li9 45.0(4) . . ?
Li9 Li6 HB 74.6(19) . . ?
Li9 Li6 HE 76.7(19) . . ?

Li9 Li6 HO 80.4(17) . . ?
Li9 Li6 HP 35.8(17) . . ?
O6 Li6 Mo1 127.4(5) . . ?
O6 Li6 HB 99.3(19) . . ?
O6 Li6 Mo2 148.3(6) . . ?
O6 Li6 HE 135(2) . . ?
O6 Li6 Mo6 100.3(5) . . ?
O6 Li6 HO 96.9(17) . . ?
O6 Li6 HP 114.1(18) . . ?
O6 Li6 Li5 135.3(6) . . ?
O6 Li6 Li7 105.9(6) . . ?
O6 Li6 Li9 147.9(6) . . ?
Mo1 Li7 HA 29(2) . . ?
Mo1 Li7 HG 106.4(19) . . ?
Mo1 Li7 Mo6 107.5(5) . . ?
Mo1 Li7 HP 92(2) . . ?
Mo1 Li7 Li2 56.2(3) . . ?
Mo1 Li7 Li4 133.9(5) . . ?
HA Li7 HG 79(3) . . ?
HA Li7 HP 97(3) . . ?
HG Li7 HP 86(3) . . ?
Mo6 Li7 HA 111(2) . . ?
Mo6 Li7 HG 82.4(19) . . ?
Mo6 Li7 HP 16(2) . . ?
Li2 Li7 HA 33(2) . . ?
Li2 Li7 HG 64.7(19) . . ?
Li2 Li7 Mo6 132.5(5) . . ?
Li2 Li7 HP 122(2) . . ?
Li4 Li7 HA 111(2) . . ?
Li4 Li7 HG 37.2(19) . . ?
Li4 Li7 Mo6 54.7(3) . . ?
Li4 Li7 HP 65(2) . . ?
Li4 Li7 Li2 101.9(6) . . ?
Li6 Li7 Mo1 61.0(4) . . ?
Li6 Li7 HA 83(2) . . ?
Li6 Li7 HG 124(2) . . ?
Li6 Li7 Mo6 56.7(4) . . ?
Li6 Li7 HP 45(2) . . ?
Li6 Li7 Li2 115.3(6) . . ?
Li6 Li7 Li4 110.4(6) . . ?
Li9 Li7 Mo1 67.2(4) . . ?
Li9 Li7 HA 52(2) . . ?
Li9 Li7 HG 52.2(19) . . ?
Li9 Li7 Mo6 64.6(4) . . ?
Li9 Li7 HP 55(2) . . ?
Li9 Li7 Li2 68.2(5) . . ?
Li9 Li7 Li4 66.9(5) . . ?
Li9 Li7 Li6 75.4(5) . . ?
O7 Li7 Mo1 118.5(8) . . ?
O7 Li7 HA 129(2) . . ?
O7 Li7 HG 117(2) . . ?
O7 Li7 Mo6 118.7(7) . . ?
O7 Li7 HP 131(2) . . ?
O7 Li7 Li2 106.9(8) . . ?
O7 Li7 Li4 106.2(8) . . ?
O7 Li7 Li6 115.0(9) . . ?
O7 Li7 Li9 169.5(12) . . ?
HD Li8 HI 137(3) . . ?

HD Li8 HK 73(3) . . ?
HD Li8 HN 79(3) . . ?
Mo4 Li8 HD 104.4(19) . . ?
Mo4 Li8 HI 35.5(17) . . ?
Mo4 Li8 HK 33(2) . . ?
Mo4 Li8 HN 88(2) . . ?
Mo4 Li8 Li1 58.3(4) . . ?
Mo4 Li8 Li5 130.2(6) . . ?
HI Li8 HK 68(3) . . ?
HI Li8 HN 83(3) . . ?
HK Li8 HN 90(3) . . ?
Li1 Li8 HD 62.8(18) . . ?
Li1 Li8 HI 91.5(17) . . ?
Li1 Li8 HK 33(2) . . ?
Li1 Li8 HN 116(2) . . ?
Li1 Li8 Li5 98.2(7) . . ?
Li3 Li8 HD 118.5(19) . . ?
Li3 Li8 Mo4 62.3(5) . . ?
Li3 Li8 HI 41.5(17) . . ?
Li3 Li8 HK 85(2) . . ?
Li3 Li8 HN 43(2) . . ?
Li3 Li8 Li1 117.4(7) . . ?
Li3 Li8 Li5 105.3(6) . . ?
Li5 Li8 HD 35.5(17) . . ?
Li5 Li8 HI 144.7(18) . . ?
Li5 Li8 HK 104(2) . . ?
Li5 Li8 HN 63(2) . . ?
Li9 Li8 HD 47.6(18) . . ?
Li9 Li8 Mo4 68.4(5) . . ?
Li9 Li8 HI 91.6(18) . . ?
Li9 Li8 HK 49(2) . . ?
Li9 Li8 HN 49(2) . . ?
Li9 Li8 Li1 67.5(6) . . ?
Li9 Li8 Li3 74.4(6) . . ?
Li9 Li8 Li5 61.8(6) . . ?
O8 Li8 HD 114(2) . . ?
O8 Li8 Mo4 126.0(9) . . ?
O8 Li8 HI 107(2) . . ?
O8 Li8 HK 135(2) . . ?
O8 Li8 HN 135(2) . . ?
O8 Li8 Li1 107.9(9) . . ?
O8 Li8 Li3 121.9(12) . . ?
O8 Li8 Li5 102.1(9) . . ?
O8 Li8 Li9 161.1(14) . . ?
Mo1 Li9 HA 31.4(19) . . ?
Mo1 Li9 Mo2 38.46(13) . . ?
Mo1 Li9 HD 71.3(19) . . ?
Mo1 Li9 Mo3 111.3(3) . . ?
Mo1 Li9 HG 105.5(18) . . ?
Mo1 Li9 HK 88(2) . . ?
Mo1 Li9 Mo5 143.1(4) . . ?
Mo1 Li9 HN 152(2) . . ?
Mo1 Li9 Mo6 111.7(3) . . ?
Mo1 Li9 HP 87.2(18) . . ?
Mo1 Li9 Li1 55.1(3) . . ?
Mo1 Li9 Li2 56.3(3) . . ?
Mo1 Li9 Li4 136.6(5) . . ?
HA Li9 HD 102(3) . . ?

HA Li9 HG 74(3) . . ?
HA Li9 HK 91(3) . . ?
HA Li9 HN 172(3) . . ?
HA Li9 HP 84(3) . . ?
Mo2 Li9 HA 69.9(19) . . ?
Mo2 Li9 HD 33.2(19) . . ?
Mo2 Li9 Mo3 141.5(4) . . ?
Mo2 Li9 HG 143.9(18) . . ?
Mo2 Li9 HK 84(2) . . ?
Mo2 Li9 Mo5 114.5(4) . . ?
Mo2 Li9 HN 114(2) . . ?
Mo2 Li9 Mo6 103.0(3) . . ?
Mo2 Li9 HP 93.3(18) . . ?
Mo2 Li9 Li1 55.9(3) . . ?
Mo2 Li9 Li2 87.9(3) . . ?
Mo2 Li9 Li4 158.4(4) . . ?
HD Li9 HG 176(3) . . ?
HD Li9 HK 77(3) . . ?
HD Li9 HN 81(3) . . ?
HD Li9 HP 103(3) . . ?
Mo3 Li9 HA 83.2(19) . . ?
Mo3 Li9 HD 145(2) . . ?
Mo3 Li9 HG 33.5(18) . . ?
Mo3 Li9 HK 69(2) . . ?
Mo3 Li9 Mo5 102.5(3) . . ?
Mo3 Li9 HN 90(2) . . ?
Mo3 Li9 HP 111.3(19) . . ?
Mo3 Li9 Li1 88.5(3) . . ?
Mo3 Li9 Li2 55.7(3) . . ?
Mo3 Li9 Li4 56.4(3) . . ?
HG Li9 HK 101(3) . . ?
HG Li9 HN 102(3) . . ?
HG Li9 HP 78(3) . . ?
Mo4 Li9 Mo1 103.7(3) . . ?
Mo4 Li9 HA 90.5(19) . . ?
Mo4 Li9 Mo2 112.8(3) . . ?
Mo4 Li9 HD 106.8(19) . . ?
Mo4 Li9 Mo3 38.53(13) . . ?
Mo4 Li9 HG 71.7(18) . . ?
Mo4 Li9 HK 30(2) . . ?
Mo4 Li9 Mo5 112.0(3) . . ?
Mo4 Li9 HN 82.1(19) . . ?
Mo4 Li9 Mo6 142.4(4) . . ?
Mo4 Li9 HP 149.8(19) . . ?
Mo4 Li9 Li1 57.5(3) . . ?
Mo4 Li9 Li2 55.6(3) . . ?
Mo4 Li9 Li4 88.7(3) . . ?
HK Li9 HN 83(3) . . ?
HK Li9 HP 175(3) . . ?
Mo5 Li9 HA 150.7(19) . . ?
Mo5 Li9 HD 89.3(19) . . ?
Mo5 Li9 HG 94.5(18) . . ?
Mo5 Li9 HK 118(2) . . ?
Mo5 Li9 HN 34.7(19) . . ?
Mo5 Li9 HP 66.6(18) . . ?
Mo5 Li9 Li1 142.4(5) . . ?
Mo5 Li9 Li2 157.6(5) . . ?
Mo5 Li9 Li4 55.5(3) . . ?

HN Li9 HP 101(3) . . ?
Mo6 Li9 HA 113.0(19) . . ?
Mo6 Li9 HD 96.6(19) . . ?
Mo6 Li9 Mo3 112.8(4) . . ?
Mo6 Li9 HG 86.4(18) . . ?
Mo6 Li9 HK 156(2) . . ?
Mo6 Li9 Mo5 38.09(13) . . ?
Mo6 Li9 HN 72.7(19) . . ?
Mo6 Li9 HP 28.6(18) . . ?
Mo6 Li9 Li1 158.6(5) . . ?
Mo6 Li9 Li2 139.8(5) . . ?
Mo6 Li9 Li4 56.8(3) . . ?
Li1 Li9 HA 65.6(19) . . ?
Li1 Li9 HD 64.1(19) . . ?
Li1 Li9 HG 112.5(19) . . ?
Li1 Li9 HK 33.7(19) . . ?
Li1 Li9 HN 111(2) . . ?
Li1 Li9 HP 142.1(18) . . ?
Li1 Li9 Li2 51.0(4) . . ?
Li2 Li9 HA 35.0(19) . . ?
Li2 Li9 HD 112(2) . . ?
Li2 Li9 HG 64.7(18) . . ?
Li2 Li9 HK 62(2) . . ?
Li2 Li9 HN 138(2) . . ?
Li2 Li9 HP 113.7(18) . . ?
Li4 Li9 HA 109(2) . . ?
Li4 Li9 HD 145(2) . . ?
Li4 Li9 HG 38.9(18) . . ?
Li4 Li9 HK 117(2) . . ?
Li4 Li9 HN 70(2) . . ?
Li4 Li9 HP 65.3(18) . . ?
Li4 Li9 Li1 144.6(5) . . ?
Li4 Li9 Li2 103.3(5) . . ?
Li5 Li9 Mo1 90.2(3) . . ?
Li5 Li9 HA 117.7(19) . . ?
Li5 Li9 Mo2 57.7(3) . . ?
Li5 Li9 HD 40.5(19) . . ?
Li5 Li9 Mo3 158.5(4) . . ?
Li5 Li9 HG 142.6(19) . . ?
Li5 Li9 Mo4 137.9(5) . . ?
Li5 Li9 HK 113(2) . . ?
Li5 Li9 Mo5 56.9(3) . . ?
Li5 Li9 HN 69(2) . . ?
Li5 Li9 Mo6 56.2(3) . . ?
Li5 Li9 HP 68.7(19) . . ?
Li5 Li9 Li1 104.4(5) . . ?
Li5 Li9 Li2 145.3(5) . . ?
Li5 Li9 Li4 108.2(4) . . ?
Li7 Li9 Mo1 67.4(5) . . ?
Li7 Li9 HA 46.3(19) . . ?
Li7 Li9 Mo2 98.5(6) . . ?
Li7 Li9 HD 128(2) . . ?
Li7 Li9 Mo3 80.5(6) . . ?
Li7 Li9 HG 50.9(18) . . ?
Li7 Li9 Mo4 112.4(6) . . ?
Li7 Li9 HK 130(2) . . ?
Li7 Li9 Mo5 105.6(5) . . ?
Li7 Li9 HN 136(2) . . ?

Li7 Li9 Mo6 71.7(5) . . ?
Li7 Li9 HP 45.8(18) . . ?
Li7 Li9 Li1 111.7(6) . . ?
Li7 Li9 Li2 68.5(5) . . ?
Li7 Li9 Li4 69.4(6) . . ?
Li7 Li9 Li5 109.6(6) . . ?
Li7 Li9 Li8 176.4(9) . . ?
Li8 Li9 Mo1 114.3(7) . . ?
Li8 Li9 HA 134(2) . . ?
Li8 Li9 Mo2 84.3(7) . . ?
Li8 Li9 HD 55(2) . . ?
Li8 Li9 Mo3 95.9(6) . . ?
Li8 Li9 HG 125.6(19) . . ?
Li8 Li9 Mo4 64.2(5) . . ?
Li8 Li9 HK 47(2) . . ?
Li8 Li9 Mo5 75.1(6) . . ?
Li8 Li9 HN 44(2) . . ?
Li8 Li9 Mo6 110.0(6) . . ?
Li8 Li9 HP 136.6(19) . . ?
Li8 Li9 Li1 68.0(6) . . ?
Li8 Li9 Li2 109.5(6) . . ?
Li8 Li9 Li4 108.6(7) . . ?
Li8 Li9 Li5 73.8(6) . . ?
C1 N1 Mo1 117.7(4) . . ?
C1 N1 C2 117.9(5) . . ?
C2 N1 Mo1 123.8(4) . . ?
C1 N2 Mo2 116.9(4) . . ?
C1 N2 C14 118.5(5) . . ?
C14 N2 Mo2 124.1(4) . . ?
C26 N3 Mo3 117.5(4) . . ?
C26 N3 C27 118.9(5) . . ?
C27 N3 Mo3 122.8(4) . . ?
C26 N4 Mo4 118.1(4) . . ?
C26 N4 C39 117.5(5) . . ?
C39 N4 Mo4 123.8(4) . . ?
C51 N5 Mo5 117.5(4) . . ?
C51 N5 C52 116.8(5) . . ?
C52 N5 Mo5 124.7(4) . . ?
C51 N6 Mo6 117.3(4) . . ?
C51 N6 C64 117.1(5) . . ?
C64 N6 Mo6 124.9(4) . . ?
C76 O1 Li1 123.2(8) . . ?
C76 O1 C79 107.1(9) . . ?
C79 O1 Li1 128.3(7) . . ?
C80 O2 Li2 129.0(7) . . ?
C83 O2 Li2 122.8(7) . . ?
C83 O2 C80 107.7(7) . . ?
C84 O3 Li3 125.9(6) . . ?
C84 O3 C87 110.6(7) . . ?
C87 O3 Li3 123.4(7) . . ?
C88 O4 Li4 123.3(6) . . ?
C91 O4 Li4 127.0(6) . . ?
C91 O4 C88 109.7(7) . . ?
C92 O5 Li5 123.9(6) . . ?
C92 O5 C95 107.2(6) . . ?
C95 O5 Li5 127.9(5) . . ?
C96 O6 Li6 123.6(7) . . ?
C99 O6 Li6 128.5(7) . . ?

C99 O6 C96 107.8(7) . . ?
C100 O7 Li7 129.4(12) . . ?
C100 O7 C103 107.2(11) . . ?
C103 O7 Li7 118.5(11) . . ?
C104 O8 Li8 116.8(11) . . ?
C104 O8 C107 111.3(10) . . ?
C107 O8 Li8 123.6(11) . . ?
N1 C1 N2 120.3(5) . . ?
N1 C1 H1 119.9 . . ?
N2 C1 H1 119.9 . . ?
C3 C2 N1 117.5(6) . . ?
C7 C2 N1 120.0(6) . . ?
C7 C2 C3 122.5(6) . . ?
C2 C3 C4 115.5(8) . . ?
C2 C3 C8 123.9(7) . . ?
C4 C3 C8 120.6(7) . . ?
C3 C4 H4 118.9 . . ?
C5 C4 C3 122.2(8) . . ?
C5 C4 H4 118.9 . . ?
C4 C5 H5 119.7 . . ?
C4 C5 C6 120.6(8) . . ?
C6 C5 H5 119.7 . . ?
C5 C6 H6 119.3 . . ?
C5 C6 C7 121.3(8) . . ?
C7 C6 H6 119.3 . . ?
C2 C7 C11 122.8(6) . . ?
C6 C7 C2 117.8(7) . . ?
C6 C7 C11 119.4(7) . . ?
C3 C8 H8 106.8 . . ?
C3 C8 C9 112.5(7) . . ?
C3 C8 C10 113.0(8) . . ?
C9 C8 H8 106.8 . . ?
C9 C8 C10 110.5(8) . . ?
C10 C8 H8 106.8 . . ?
C8 C9 H9A 109.5 . . ?
C8 C9 H9B 109.5 . . ?
C8 C9 H9C 109.5 . . ?
H9A C9 H9B 109.5 . . ?
H9A C9 H9C 109.5 . . ?
H9B C9 H9C 109.5 . . ?
C8 C10 H10A 109.5 . . ?
C8 C10 H10B 109.5 . . ?
C8 C10 H10C 109.5 . . ?
H10A C10 H10B 109.5 . . ?
H10A C10 H10C 109.5 . . ?
H10B C10 H10C 109.5 . . ?
C7 C11 H11 107.7 . . ?
C7 C11 C12 112.2(7) . . ?
C7 C11 C13 112.8(8) . . ?
C12 C11 H11 107.7 . . ?
C13 C11 H11 107.7 . . ?
C13 C11 C12 108.6(8) . . ?
C11 C12 H12A 109.5 . . ?
C11 C12 H12B 109.5 . . ?
C11 C12 H12C 109.5 . . ?
H12A C12 H12B 109.5 . . ?
H12A C12 H12C 109.5 . . ?
H12B C12 H12C 109.5 . . ?

C11 C13 H13A 109.5 . . ?
C11 C13 H13B 109.5 . . ?
C11 C13 H13C 109.5 . . ?
H13A C13 H13B 109.5 . . ?
H13A C13 H13C 109.5 . . ?
H13B C13 H13C 109.5 . . ?
C15 C14 N2 120.7(6) . . ?
C19 C14 N2 118.8(6) . . ?
C19 C14 C15 120.3(6) . . ?
C14 C15 C16 117.9(7) . . ?
C14 C15 C20 121.8(7) . . ?
C16 C15 C20 120.3(8) . . ?
C15 C16 H16 119.2 . . ?
C17 C16 C15 121.5(8) . . ?
C17 C16 H16 119.2 . . ?
C16 C17 H17 120.1 . . ?
C16 C17 C18 119.8(8) . . ?
C18 C17 H17 120.1 . . ?
C17 C18 H18 119.3 . . ?
C17 C18 C19 121.5(8) . . ?
C19 C18 H18 119.3 . . ?
C14 C19 C23 121.8(6) . . ?
C18 C19 C14 118.9(7) . . ?
C18 C19 C23 119.2(7) . . ?
C15 C20 H20 107.9 . . ?
C15 C20 C21 110.8(9) . . ?
C15 C20 C22 110.5(8) . . ?
C21 C20 H20 107.9 . . ?
C22 C20 H20 107.9 . . ?
C22 C20 C21 111.7(10) . . ?
C20 C21 H21A 109.5 . . ?
C20 C21 H21B 109.5 . . ?
C20 C21 H21C 109.5 . . ?
H21A C21 H21B 109.5 . . ?
H21A C21 H21C 109.5 . . ?
H21B C21 H21C 109.5 . . ?
C20 C22 H22A 109.5 . . ?
C20 C22 H22B 109.5 . . ?
C20 C22 H22C 109.5 . . ?
H22A C22 H22B 109.5 . . ?
H22A C22 H22C 109.5 . . ?
H22B C22 H22C 109.5 . . ?
C19 C23 H23 107.7 . . ?
C19 C23 C24 111.8(8) . . ?
C19 C23 C25 112.2(7) . . ?
C24 C23 H23 107.7 . . ?
C24 C23 C25 109.6(8) . . ?
C25 C23 H23 107.7 . . ?
C23 C24 H24A 109.5 . . ?
C23 C24 H24B 109.5 . . ?
C23 C24 H24C 109.5 . . ?
H24A C24 H24B 109.5 . . ?
H24A C24 H24C 109.5 . . ?
H24B C24 H24C 109.5 . . ?
C23 C25 H25A 109.5 . . ?
C23 C25 H25B 109.5 . . ?
C23 C25 H25C 109.5 . . ?
H25A C25 H25B 109.5 . . ?

H25A C25 H25C 109.5 . . ?
H25B C25 H25C 109.5 . . ?
N3 C26 H26 120.3 . . ?
N4 C26 N3 119.4(5) . . ?
N4 C26 H26 120.3 . . ?
C28 C27 N3 120.6(6) . . ?
C28 C27 C32 120.9(6) . . ?
C32 C27 N3 118.4(6) . . ?
C27 C28 C29 119.2(7) . . ?
C27 C28 C33 122.0(6) . . ?
C29 C28 C33 118.8(7) . . ?
C28 C29 H29 119.6 . . ?
C30 C29 C28 120.9(7) . . ?
C30 C29 H29 119.6 . . ?
C29 C30 H30 120.2 . . ?
C31 C30 C29 119.5(7) . . ?
C31 C30 H30 120.2 . . ?
C30 C31 H31 119.0 . . ?
C30 C31 C32 122.1(7) . . ?
C32 C31 H31 119.0 . . ?
C27 C32 C36 123.4(6) . . ?
C31 C32 C27 117.4(7) . . ?
C31 C32 C36 119.2(6) . . ?
C28 C33 H33 107.5 . . ?
C34 C33 C28 112.6(8) . . ?
C34 C33 H33 107.5 . . ?
C35 C33 C28 110.6(7) . . ?
C35 C33 H33 107.5 . . ?
C35 C33 C34 110.8(9) . . ?
C33 C34 H34A 109.5 . . ?
C33 C34 H34B 109.5 . . ?
C33 C34 H34C 109.5 . . ?
H34A C34 H34B 109.5 . . ?
H34A C34 H34C 109.5 . . ?
H34B C34 H34C 109.5 . . ?
C33 C35 H35A 109.5 . . ?
C33 C35 H35B 109.5 . . ?
C33 C35 H35C 109.5 . . ?
H35A C35 H35B 109.5 . . ?
H35A C35 H35C 109.5 . . ?
H35B C35 H35C 109.5 . . ?
C32 C36 H36 107.7 . . ?
C37 C36 C32 109.8(6) . . ?
C37 C36 H36 107.7 . . ?
C38 C36 C32 112.1(7) . . ?
C38 C36 H36 107.7 . . ?
C38 C36 C37 111.8(8) . . ?
C36 C37 H37A 109.5 . . ?
C36 C37 H37B 109.5 . . ?
C36 C37 H37C 109.5 . . ?
H37A C37 H37B 109.5 . . ?
H37A C37 H37C 109.5 . . ?
H37B C37 H37C 109.5 . . ?
C36 C38 H38A 109.5 . . ?
C36 C38 H38B 109.5 . . ?
C36 C38 H38C 109.5 . . ?
H38A C38 H38B 109.5 . . ?
H38A C38 H38C 109.5 . . ?

H38B C38 H38C 109.5 . . ?
C40 C39 N4 120.9(7) . . ?
C40 C39 C44 120.1(7) . . ?
C44 C39 N4 118.9(7) . . ?
C39 C40 C45 122.1(7) . . ?
C41 C40 C39 119.2(9) . . ?
C41 C40 C45 118.7(9) . . ?
C40 C41 H41 119.4 . . ?
C42 C41 C40 121.3(10) . . ?
C42 C41 H41 119.4 . . ?
C41 C42 H42 119.9 . . ?
C41 C42 C43 120.2(8) . . ?
C43 C42 H42 119.9 . . ?
C42 C43 H43 119.3 . . ?
C42 C43 C44 121.4(10) . . ?
C44 C43 H43 119.3 . . ?
C39 C44 C43 117.8(9) . . ?
C39 C44 C48 122.7(7) . . ?
C43 C44 C48 119.5(8) . . ?
C40 C45 H45 107.8 . . ?
C46 C45 C40 110.6(9) . . ?
C46 C45 H45 107.8 . . ?
C47 C45 C40 112.2(8) . . ?
C47 C45 H45 107.8 . . ?
C47 C45 C46 110.4(9) . . ?
C45 C46 H46A 109.5 . . ?
C45 C46 H46B 109.5 . . ?
C45 C46 H46C 109.5 . . ?
H46A C46 H46B 109.5 . . ?
H46A C46 H46C 109.5 . . ?
H46B C46 H46C 109.5 . . ?
C45 C47 H47A 109.5 . . ?
C45 C47 H47B 109.5 . . ?
C45 C47 H47C 109.5 . . ?
H47A C47 H47B 109.5 . . ?
H47A C47 H47C 109.5 . . ?
H47B C47 H47C 109.5 . . ?
C44 C48 H48 107.3 . . ?
C44 C48 C49 109.7(8) . . ?
C44 C48 C50 112.0(10) . . ?
C49 C48 H48 107.3 . . ?
C50 C48 H48 107.3 . . ?
C50 C48 C49 112.9(10) . . ?
C48 C49 H49A 109.5 . . ?
C48 C49 H49B 109.5 . . ?
C48 C49 H49C 109.5 . . ?
H49A C49 H49B 109.5 . . ?
H49A C49 H49C 109.5 . . ?
H49B C49 H49C 109.5 . . ?
C48 C50 H50A 109.5 . . ?
C48 C50 H50B 109.5 . . ?
C48 C50 H50C 109.5 . . ?
H50A C50 H50B 109.5 . . ?
H50A C50 H50C 109.5 . . ?
H50B C50 H50C 109.5 . . ?
N5 C51 H51 120.2 . . ?
N6 C51 N5 119.7(5) . . ?
N6 C51 H51 120.2 . . ?

C53 C52 N5 120.1(7) . . ?
C57 C52 N5 119.6(7) . . ?
C57 C52 C53 120.2(7) . . ?
C52 C53 C54 119.1(9) . . ?
C52 C53 C58 121.2(7) . . ?
C54 C53 C58 119.7(9) . . ?
C53 C54 H54 119.6 . . ?
C55 C54 C53 120.9(10) . . ?
C55 C54 H54 119.6 . . ?
C54 C55 H55 120.3 . . ?
C56 C55 C54 119.3(9) . . ?
C56 C55 H55 120.3 . . ?
C55 C56 H56 119.2 . . ?
C55 C56 C57 121.6(9) . . ?
C57 C56 H56 119.2 . . ?
C52 C57 C61 121.5(6) . . ?
C56 C57 C52 118.7(8) . . ?
C56 C57 C61 119.7(8) . . ?
C53 C58 H58 107.5 . . ?
C53 C58 C60 111.7(9) . . ?
C59 C58 C53 111.8(9) . . ?
C59 C58 H58 107.5 . . ?
C59 C58 C60 110.5(9) . . ?
C60 C58 H58 107.5 . . ?
C58 C59 H59A 109.5 . . ?
C58 C59 H59B 109.5 . . ?
C58 C59 H59C 109.5 . . ?
H59A C59 H59B 109.5 . . ?
H59A C59 H59C 109.5 . . ?
H59B C59 H59C 109.5 . . ?
C58 C60 H60A 109.5 . . ?
C58 C60 H60B 109.5 . . ?
C58 C60 H60C 109.5 . . ?
H60A C60 H60B 109.5 . . ?
H60A C60 H60C 109.5 . . ?
H60B C60 H60C 109.5 . . ?
C57 C61 H61 107.4 . . ?
C57 C61 C62 111.3(7) . . ?
C57 C61 C63 112.6(7) . . ?
C62 C61 H61 107.4 . . ?
C62 C61 C63 110.5(7) . . ?
C63 C61 H61 107.4 . . ?
C61 C62 H62A 109.5 . . ?
C61 C62 H62B 109.5 . . ?
C61 C62 H62C 109.5 . . ?
H62A C62 H62B 109.5 . . ?
H62A C62 H62C 109.5 . . ?
H62B C62 H62C 109.5 . . ?
C61 C63 H63A 109.5 . . ?
C61 C63 H63B 109.5 . . ?
C61 C63 H63C 109.5 . . ?
H63A C63 H63B 109.5 . . ?
H63A C63 H63C 109.5 . . ?
H63B C63 H63C 109.5 . . ?
N6 C64 C69 119.2(6) . . ?
C65 C64 N6 120.5(6) . . ?
C65 C64 C69 120.3(6) . . ?
C64 C65 C70 121.2(6) . . ?

C66 C65 C64 118.3(7) . . ?
C66 C65 C70 120.5(7) . . ?
C65 C66 H66 119.0 . . ?
C67 C66 C65 122.1(8) . . ?
C67 C66 H66 119.0 . . ?
C66 C67 H67 120.4 . . ?
C66 C67 C68 119.2(8) . . ?
C68 C67 H67 120.4 . . ?
C67 C68 H68 118.9 . . ?
C67 C68 C69 122.2(8) . . ?
C69 C68 H68 118.9 . . ?
C64 C69 C73 122.1(6) . . ?
C68 C69 C64 118.0(7) . . ?
C68 C69 C73 119.9(7) . . ?
C65 C70 H70 107.5 . . ?
C65 C70 C71 110.9(8) . . ?
C65 C70 C72 112.4(7) . . ?
C71 C70 H70 107.5 . . ?
C72 C70 H70 107.5 . . ?
C72 C70 C71 110.8(8) . . ?
C70 C71 H71A 109.5 . . ?
C70 C71 H71B 109.5 . . ?
C70 C71 H71C 109.5 . . ?
H71A C71 H71B 109.5 . . ?
H71A C71 H71C 109.5 . . ?
H71B C71 H71C 109.5 . . ?
C70 C72 H72A 109.5 . . ?
C70 C72 H72B 109.5 . . ?
C70 C72 H72C 109.5 . . ?
H72A C72 H72B 109.5 . . ?
H72A C72 H72C 109.5 . . ?
H72B C72 H72C 109.5 . . ?
C69 C73 H73 107.2 . . ?
C69 C73 C74 113.0(7) . . ?
C69 C73 C75 111.1(7) . . ?
C74 C73 H73 107.2 . . ?
C75 C73 H73 107.2 . . ?
C75 C73 C74 110.7(7) . . ?
C73 C74 H74A 109.5 . . ?
C73 C74 H74B 109.5 . . ?
C73 C74 H74C 109.5 . . ?
H74A C74 H74B 109.5 . . ?
H74A C74 H74C 109.5 . . ?
H74B C74 H74C 109.5 . . ?
C73 C75 H75A 109.5 . . ?
C73 C75 H75B 109.5 . . ?
C73 C75 H75C 109.5 . . ?
H75A C75 H75B 109.5 . . ?
H75A C75 H75C 109.5 . . ?
H75B C75 H75C 109.5 . . ?
O1 C76 H76A 110.3 . . ?
O1 C76 H76B 110.3 . . ?
O1 C76 C77 107.1(11) . . ?
H76A C76 H76B 108.6 . . ?
C77 C76 H76A 110.3 . . ?
C77 C76 H76B 110.3 . . ?
C76 C77 H77A 111.5 . . ?
C76 C77 H77B 111.5 . . ?

C76 C77 C78 101.5(11) . . ?
H77A C77 H77B 109.3 . . ?
C78 C77 H77A 111.5 . . ?
C78 C77 H77B 111.5 . . ?
C77 C78 H78A 110.8 . . ?
C77 C78 H78B 110.8 . . ?
H78A C78 H78B 108.9 . . ?
C79 C78 C77 104.8(11) . . ?
C79 C78 H78A 110.8 . . ?
C79 C78 H78B 110.8 . . ?
O1 C79 C78 108.8(11) . . ?
O1 C79 H79A 109.9 . . ?
O1 C79 H79B 109.9 . . ?
C78 C79 H79A 109.9 . . ?
C78 C79 H79B 109.9 . . ?
H79A C79 H79B 108.3 . . ?
O2 C80 H80A 110.5 . . ?
O2 C80 H80B 110.5 . . ?
O2 C80 C81 106.3(9) . . ?
H80A C80 H80B 108.7 . . ?
C81 C80 H80A 110.5 . . ?
C81 C80 H80B 110.5 . . ?
C80 C81 H81A 111.1 . . ?
C80 C81 H81B 111.1 . . ?
C80 C81 C82 103.3(10) . . ?
H81A C81 H81B 109.1 . . ?
C82 C81 H81A 111.1 . . ?
C82 C81 H81B 111.1 . . ?
C81 C82 H82A 110.6 . . ?
C81 C82 H82B 110.6 . . ?
H82A C82 H82B 108.7 . . ?
C83 C82 C81 105.7(10) . . ?
C83 C82 H82A 110.6 . . ?
C83 C82 H82B 110.6 . . ?
O2 C83 C82 108.5(9) . . ?
O2 C83 H83A 110.0 . . ?
O2 C83 H83B 110.0 . . ?
C82 C83 H83A 110.0 . . ?
C82 C83 H83B 110.0 . . ?
H83A C83 H83B 108.4 . . ?
O3 C84 H84A 109.6 . . ?
O3 C84 H84B 109.6 . . ?
O3 C84 C85 110.2(8) . . ?
H84A C84 H84B 108.1 . . ?
C85 C84 H84A 109.6 . . ?
C85 C84 H84B 109.6 . . ?
C84 C85 H85A 110.6 . . ?
C84 C85 H85B 110.6 . . ?
C84 C85 C86 105.5(8) . . ?
H85A C85 H85B 108.8 . . ?
C86 C85 H85A 110.6 . . ?
C86 C85 H85B 110.6 . . ?
C85 C86 H86A 110.3 . . ?
C85 C86 H86B 110.3 . . ?
H86A C86 H86B 108.5 . . ?
C87 C86 C85 107.2(8) . . ?
C87 C86 H86A 110.3 . . ?
C87 C86 H86B 110.3 . . ?

O3 C87 C86 105.8(8) . . ?
O3 C87 H87A 110.6 . . ?
O3 C87 H87B 110.6 . . ?
C86 C87 H87A 110.6 . . ?
C86 C87 H87B 110.6 . . ?
H87A C87 H87B 108.7 . . ?
O4 C88 H88A 110.3 . . ?
O4 C88 H88B 110.3 . . ?
H88A C88 H88B 108.6 . . ?
C89 C88 O4 107.0(9) . . ?
C89 C88 H88A 110.3 . . ?
C89 C88 H88B 110.3 . . ?
C88 C89 H89A 110.2 . . ?
C88 C89 H89B 110.2 . . ?
C88 C89 C90 107.4(10) . . ?
H89A C89 H89B 108.5 . . ?
C90 C89 H89A 110.2 . . ?
C90 C89 H89B 110.2 . . ?
C89 C90 H90A 110.6 . . ?
C89 C90 H90B 110.6 . . ?
H90A C90 H90B 108.7 . . ?
C91 C90 C89 105.7(10) . . ?
C91 C90 H90A 110.6 . . ?
C91 C90 H90B 110.6 . . ?
O4 C91 C90 108.1(9) . . ?
O4 C91 H91A 110.1 . . ?
O4 C91 H91B 110.1 . . ?
C90 C91 H91A 110.1 . . ?
C90 C91 H91B 110.1 . . ?
H91A C91 H91B 108.4 . . ?
O5 C92 H92A 110.1 . . ?
O5 C92 H92B 110.1 . . ?
O5 C92 C93 107.9(6) . . ?
H92A C92 H92B 108.4 . . ?
C93 C92 H92A 110.1 . . ?
C93 C92 H92B 110.1 . . ?
C92 C93 H93A 110.9 . . ?
C92 C93 H93B 110.9 . . ?
H93A C93 H93B 108.9 . . ?
C94 C93 C92 104.3(6) . . ?
C94 C93 H93A 110.9 . . ?
C94 C93 H93B 110.9 . . ?
C93 C94 H94A 111.0 . . ?
C93 C94 H94B 111.0 . . ?
C93 C94 C95 103.8(6) . . ?
H94A C94 H94B 109.0 . . ?
C95 C94 H94A 111.0 . . ?
C95 C94 H94B 111.0 . . ?
O5 C95 C94 104.0(6) . . ?
O5 C95 H95A 111.0 . . ?
O5 C95 H95B 111.0 . . ?
C94 C95 H95A 111.0 . . ?
C94 C95 H95B 111.0 . . ?
H95A C95 H95B 109.0 . . ?
O6 C96 H96A 110.9 . . ?
O6 C96 H96B 110.9 . . ?
O6 C96 C97 104.3(8) . . ?
H96A C96 H96B 108.9 . . ?

C97 C96 H96A 110.9 . . ?
C97 C96 H96B 110.9 . . ?
C96 C97 H97A 111.3 . . ?
C96 C97 H97B 111.3 . . ?
H97A C97 H97B 109.2 . . ?
C98 C97 C96 102.3(9) . . ?
C98 C97 H97A 111.3 . . ?
C98 C97 H97B 111.3 . . ?
C97 C98 H98A 111.1 . . ?
C97 C98 H98B 111.1 . . ?
H98A C98 H98B 109.1 . . ?
C99 C98 C97 103.2(8) . . ?
C99 C98 H98A 111.1 . . ?
C99 C98 H98B 111.1 . . ?
O6 C99 C98 110.0(8) . . ?
O6 C99 H99A 109.7 . . ?
O6 C99 H99B 109.7 . . ?
C98 C99 H99A 109.7 . . ?
C98 C99 H99B 109.7 . . ?
H99A C99 H99B 108.2 . . ?
O7 C100 H10D 110.5 . . ?
O7 C100 H10E 110.5 . . ?
O7 C100 C101 106.0(12) . . ?
H10D C100 H10E 108.7 . . ?
C101 C100 H10D 110.5 . . ?
C101 C100 H10E 110.5 . . ?
C100 C101 H10F 111.5 . . ?
C100 C101 H10G 111.5 . . ?
H10F C101 H10G 109.3 . . ?
C102 C101 C100 101.3(12) . . ?
C102 C101 H10F 111.5 . . ?
C102 C101 H10G 111.5 . . ?
C101 C102 H10H 110.9 . . ?
C101 C102 H10I 110.9 . . ?
H10H C102 H10I 108.9 . . ?
C103 C102 C101 104.4(12) . . ?
C103 C102 H10H 110.9 . . ?
C103 C102 H10I 110.9 . . ?
O7 C103 C102 109.5(13) . . ?
O7 C103 H10J 109.8 . . ?
O7 C103 H10K 109.8 . . ?
C102 C103 H10J 109.8 . . ?
C102 C103 H10K 109.8 . . ?
H10J C103 H10K 108.2 . . ?
O8 C104 H10L 110.0 . . ?
O8 C104 H10M 110.0 . . ?
O8 C104 C105 108.5(12) . . ?
H10L C104 H10M 108.4 . . ?
C105 C104 H10L 110.0 . . ?
C105 C104 H10M 110.0 . . ?
O8 C107 H10N 111.9 . . ?
O8 C107 H10O 111.9 . . ?
O8 C107 C106 99.1(13) . . ?
H10N C107 H10O 109.6 . . ?
C106 C107 H10N 111.9 . . ?
C106 C107 H10O 111.9 . . ?
C104 C105 H10P 111.7 . . ?
C104 C105 H10Q 111.7 . . ?

```
 H10P C105 H10Q 109.4 . . ?
 C106 C105 C104 100.6(13) . . ?
 C106 C105 H10P 111.7 . . ?
 C106 C105 H10Q 111.7 . . ?
 C107 C106 H10R 109.8 . . ?
 C107 C106 H10S 109.8 . . ?
 C105 C106 C107 109.2(15) . . ?
 C105 C106 H10R 109.8 . . ?
 C105 C106 H10S 109.8 . . ?
 H10R C106 H10S 108.3 . . ?

loop_
  _geom_torsion_atom_site_label_1
  _geom_torsion_atom_site_label_2
  _geom_torsion_atom_site_label_3
  _geom_torsion_atom_site_label_4
  _geom_torsion
  _geom_torsion_site_symmetry_1
  _geom_torsion_site_symmetry_2
  _geom_torsion_site_symmetry_3
  _geom_torsion_site_symmetry_4
  _geom_torsion_publ_flag
 Mo1 Li7 O7 C100 169.8(14) . . . . ?
 Mo1 Li7 O7 C103 -38.6(17) . . . . ?
 Mo1 N1 C1 N2 -1.5(8) . . . . ?
 Mo1 N1 C2 C3 95.2(6) . . . . ?
 Mo1 N1 C2 C7 -81.6(7) . . . . ?
 Mo2 N2 C1 N1 -2.8(8) . . . . ?
 Mo2 N2 C14 C15 -86.1(7) . . . . ?
 Mo2 N2 C14 C19 89.4(7) . . . . ?
 Mo3 N3 C26 N4 0.0(8) . . . . ?
 Mo3 N3 C27 C28 -86.3(6) . . . . ?
 Mo3 N3 C27 C32 90.8(6) . . . . ?
 Mo4 N4 C26 N3 -2.6(8) . . . . ?
 Mo4 N4 C39 C40 -82.0(8) . . . . ?
 Mo4 N4 C39 C44 93.1(7) . . . . ?
 Mo5 N5 C51 N6 -2.6(8) . . . . ?
 Mo5 N5 C52 C53 -87.6(8) . . . . ?
 Mo5 N5 C52 C57 90.0(7) . . . . ?
 Mo6 Li7 O7 C100 36(2) . . . . ?
 Mo6 Li7 O7 C103 -172.0(11) . . . . ?
 Mo6 N6 C51 N5 -1.9(8) . . . . ?
 Mo6 N6 C64 C65 -86.9(7) . . . . ?
 Mo6 N6 C64 C69 90.9(7) . . . . ?
 Li1 O1 C76 C77 160.0(12) . . . . ?
 Li1 O1 C79 C78 -174.3(13) . . . . ?
 Li2 Li7 O7 C100 -130.0(15) . . . . ?
 Li2 Li7 O7 C103 21.6(16) . . . . ?
 Li2 O2 C80 C81 -144.8(11) . . . . ?
 Li2 O2 C83 C82 158.7(11) . . . . ?
 Li3 O3 C84 C85 179.9(11) . . . . ?
 Li3 O3 C87 C86 176.2(10) . . . . ?
 Li4 Li7 O7 C100 -21.8(18) . . . . ?
 Li4 Li7 O7 C103 129.8(12) . . . . ?
 Li4 O4 C88 C89 -167.3(12) . . . . ?
 Li4 O4 C91 C90 173.9(13) . . . . ?
 Li5 O5 C92 C93 148.5(7) . . . . ?
 Li5 O5 C95 C94 -134.0(7) . . . . ?
```

Li6 Li7 O7 C100 100.6(17) . . . . ?
Li6 Li7 O7 C103 -107.8(13) . . . . ?
Li6 O6 C96 C97 -145.6(9) . . . . ?
Li6 O6 C99 C98 162.5(10) . . . . ?
Li7 O7 C100 C101 119.9(15) . . . . ?
Li7 O7 C103 C102 -123.1(15) . . . . ?
Li8 O8 C104 C105 137.6(14) . . . . ?
Li8 O8 C107 C106 -119.6(14) . . . . ?
Li9 Li7 O7 C100 -69(6) . . . . ?
Li9 Li7 O7 C103 82(6) . . . . ?
N1 C2 C3 C4 179.6(6) . . . . ?
N1 C2 C3 C8 0.5(10) . . . . ?
N1 C2 C7 C6 179.0(6) . . . . ?
N1 C2 C7 C11 -2.8(10) . . . . ?
N2 C14 C15 C16 174.7(6) . . . . ?
N2 C14 C15 C20 -7.8(11) . . . . ?
N2 C14 C19 C18 -174.5(6) . . . . ?
N2 C14 C19 C23 7.2(9) . . . . ?
N3 C27 C28 C29 176.3(6) . . . . ?
N3 C27 C28 C33 -5.5(10) . . . . ?
N3 C27 C32 C31 -177.6(6) . . . . ?
N3 C27 C32 C36 3.4(9) . . . . ?
N4 C39 C40 C41 176.9(7) . . . . ?
N4 C39 C40 C45 -5.0(11) . . . . ?
N4 C39 C44 C43 -176.5(7) . . . . ?
N4 C39 C44 C48 3.1(11) . . . . ?
N5 C52 C53 C54 174.8(8) . . . . ?
N5 C52 C53 C58 -4.5(12) . . . . ?
N5 C52 C57 C56 -176.2(7) . . . . ?
N5 C52 C57 C61 3.9(10) . . . . ?
N6 C64 C65 C66 179.1(6) . . . . ?
N6 C64 C65 C70 -0.2(10) . . . . ?
N6 C64 C69 C68 -180.0(6) . . . . ?
N6 C64 C69 C73 -1.4(10) . . . . ?
O1 C76 C77 C78 31.9(19) . . . . ?
O2 C80 C81 C82 -29.2(17) . . . . ?
O3 C84 C85 C86 1.6(17) . . . . ?
O4 C88 C89 C90 -14(2) . . . . ?
O5 C92 C93 C94 -1.9(10) . . . . ?
O6 C96 C97 C98 -35.5(13) . . . . ?
O7 C100 C101 C102 22.0(19) . . . . ?
O8 C104 C105 C106 -10(2) . . . . ?
O8 C107 C106 C105 -34(2) . . . . ?
C1 N1 C2 C3 -75.6(8) . . . . ?
C1 N1 C2 C7 107.6(7) . . . . ?
C1 N2 C14 C15 102.0(7) . . . . ?
C1 N2 C14 C19 -82.5(7) . . . . ?
C2 N1 C1 N2 169.9(6) . . . . ?
C2 C3 C4 C5 2.1(11) . . . . ?
C2 C3 C8 C9 107.3(9) . . . . ?
C2 C3 C8 C10 -126.7(8) . . . . ?
C2 C7 C11 C12 -109.4(9) . . . . ?
C2 C7 C11 C13 127.5(8) . . . . ?
C3 C2 C7 C6 2.4(10) . . . . ?
C3 C2 C7 C11 -179.4(6) . . . . ?
C3 C4 C5 C6 0.8(13) . . . . ?
C4 C3 C8 C9 -71.7(10) . . . . ?
C4 C3 C8 C10 54.3(10) . . . . ?

C4 C5 C6 C7 -2.3(12) . . . . ?
C5 C6 C7 C2 0.7(11) . . . . ?
C5 C6 C7 C11 -177.5(7) . . . . ?
C6 C7 C11 C12 68.7(10) . . . . ?
C6 C7 C11 C13 -54.4(10) . . . . ?
C7 C2 C3 C4 -3.7(10) . . . . ?
C7 C2 C3 C8 177.2(7) . . . . ?
C8 C3 C4 C5 -178.8(8) . . . . ?
C14 N2 C1 N1 169.7(6) . . . . ?
C14 C15 C16 C17 0.3(12) . . . . ?
C14 C15 C20 C21 -118.5(10) . . . . ?
C14 C15 C20 C22 117.2(9) . . . . ?
C14 C19 C23 C24 109.8(8) . . . . ?
C14 C19 C23 C25 -126.6(8) . . . . ?
C15 C14 C19 C18 1.0(10) . . . . ?
C15 C14 C19 C23 -177.3(6) . . . . ?
C15 C16 C17 C18 -0.1(14) . . . . ?
C16 C15 C20 C21 59.0(12) . . . . ?
C16 C15 C20 C22 -65.3(11) . . . . ?
C16 C17 C18 C19 0.4(14) . . . . ?
C17 C18 C19 C14 -0.9(12) . . . . ?
C17 C18 C19 C23 177.5(8) . . . . ?
C18 C19 C23 C24 -68.5(10) . . . . ?
C18 C19 C23 C25 55.0(10) . . . . ?
C19 C14 C15 C16 -0.7(10) . . . . ?
C19 C14 C15 C20 176.8(7) . . . . ?
C20 C15 C16 C17 -177.3(8) . . . . ?
C26 N3 C27 C28 104.0(7) . . . . ?
C26 N3 C27 C32 -78.8(7) . . . . ?
C26 N4 C39 C40 106.8(8) . . . . ?
C26 N4 C39 C44 -78.1(8) . . . . ?
C27 N3 C26 N4 170.1(6) . . . . ?
C27 C28 C29 C30 0.8(11) . . . . ?
C27 C28 C33 C34 124.5(8) . . . . ?
C27 C28 C33 C35 -110.9(9) . . . . ?
C27 C32 C36 C37 109.6(8) . . . . ?
C27 C32 C36 C38 -125.5(8) . . . . ?
C28 C27 C32 C31 -0.5(9) . . . . ?
C28 C27 C32 C36 -179.4(6) . . . . ?
C28 C29 C30 C31 0.4(12) . . . . ?
C29 C28 C33 C34 -57.3(10) . . . . ?
C29 C28 C33 C35 67.3(10) . . . . ?
C29 C30 C31 C32 -1.8(12) . . . . ?
C30 C31 C32 C27 1.8(11) . . . . ?
C30 C31 C32 C36 -179.2(7) . . . . ?
C31 C32 C36 C37 -69.3(9) . . . . ?
C31 C32 C36 C38 55.5(9) . . . . ?
C32 C27 C28 C29 -0.8(10) . . . . ?
C32 C27 C28 C33 177.4(6) . . . . ?
C33 C28 C29 C30 -177.5(7) . . . . ?
C39 N4 C26 N3 169.1(6) . . . . ?
C39 C40 C41 C42 -0.8(14) . . . . ?
C39 C40 C45 C46 123.0(9) . . . . ?
C39 C40 C45 C47 -113.2(9) . . . . ?
C39 C44 C48 C49 107.3(9) . . . . ?
C39 C44 C48 C50 -126.6(9) . . . . ?
C40 C39 C44 C43 -1.3(11) . . . . ?
C40 C39 C44 C48 178.3(7) . . . . ?

C40 C41 C42 C43 -0.8(16) . . . . ?
C41 C40 C45 C46 -58.8(11) . . . . ?
C41 C40 C45 C47 65.0(11) . . . . ?
C41 C42 C43 C44 1.3(15) . . . . ?
C42 C43 C44 C39 -0.2(13) . . . . ?
C42 C43 C44 C48 -179.8(9) . . . . ?
C43 C44 C48 C49 -73.1(10) . . . . ?
C43 C44 C48 C50 53.0(12) . . . . ?
C44 C39 C40 C41 1.8(11) . . . . ?
C44 C39 C40 C45 180.0(7) . . . . ?
C45 C40 C41 C42 -179.0(9) . . . . ?
C51 N5 C52 C53 103.8(8) . . . . ?
C51 N5 C52 C57 -78.6(8) . . . . ?
C51 N6 C64 C65 102.6(7) . . . . ?
C51 N6 C64 C69 -79.6(8) . . . . ?
C52 N5 C51 N6 166.9(6) . . . . ?
C52 C53 C54 C55 1.4(16) . . . . ?
C52 C53 C58 C59 114.6(10) . . . . ?
C52 C53 C58 C60 -120.9(10) . . . . ?
C52 C57 C61 C62 107.2(8) . . . . ?
C52 C57 C61 C63 -128.1(8) . . . . ?
C53 C52 C57 C56 1.3(11) . . . . ?
C53 C52 C57 C61 -178.6(7) . . . . ?
C53 C54 C55 C56 1.3(18) . . . . ?
C54 C53 C58 C59 -64.6(12) . . . . ?
C54 C53 C58 C60 59.8(13) . . . . ?
C54 C55 C56 C57 -2.8(17) . . . . ?
C55 C56 C57 C52 1.5(14) . . . . ?
C55 C56 C57 C61 -178.6(9) . . . . ?
C56 C57 C61 C62 -72.7(10) . . . . ?
C56 C57 C61 C63 52.0(10) . . . . ?
C57 C52 C53 C54 -2.7(13) . . . . ?
C57 C52 C53 C58 178.0(8) . . . . ?
C58 C53 C54 C55 -179.3(10) . . . . ?
C64 N6 C51 N5 169.3(6) . . . . ?
C64 C65 C66 C67 0.0(12) . . . . ?
C64 C65 C70 C71 111.4(8) . . . . ?
C64 C65 C70 C72 -123.9(8) . . . . ?
C64 C69 C73 C74 -131.2(7) . . . . ?
C64 C69 C73 C75 103.7(8) . . . . ?
C65 C64 C69 C68 -2.1(10) . . . . ?
C65 C64 C69 C73 176.4(7) . . . . ?
C65 C66 C67 C68 -0.3(13) . . . . ?
C66 C65 C70 C71 -67.8(9) . . . . ?
C66 C65 C70 C72 56.8(11) . . . . ?
C66 C67 C68 C69 -0.6(13) . . . . ?
C67 C68 C69 C64 1.9(12) . . . . ?
C67 C68 C69 C73 -176.8(8) . . . . ?
C68 C69 C73 C74 47.4(10) . . . . ?
C68 C69 C73 C75 -77.7(9) . . . . ?
C69 C64 C65 C66 1.2(10) . . . . ?
C69 C64 C65 C70 -178.0(7) . . . . ?
C70 C65 C66 C67 179.3(8) . . . . ?
C76 O1 C79 C78 19(2) . . . . ?
C76 C77 C78 C79 -19(2) . . . . ?
C77 C78 C79 O1 1(2) . . . . ?
C79 O1 C76 C77 -32.6(19) . . . . ?
C80 O2 C83 C82 -13.9(16) . . . . ?

```
 C80 C81 C82 C83 20.4(19) . . . . ?
 C81 C82 C83 O2 -4.8(19) . . . . ?
 C83 O2 C80 C81 27.2(15) . . . . ?
 C84 O3 C87 C86 -7.7(16) . . . . ?
 C84 C85 C86 C87 -6.3(17) . . . . ?
 C85 C86 C87 O3 8.4(17) . . . . ?
 C87 O3 C84 C85 3.8(17) . . . . ?
 C88 O4 C91 C90 -6.9(18) . . . . ?
 C88 C89 C90 C91 10(2) . . . . ?
 C89 C90 C91 O4 -2(2) . . . . ?
 C91 O4 C88 C89 13.4(16) . . . . ?
 C92 O5 C95 C94 34.5(8) . . . . ?
 C92 C93 C94 C95 22.1(10) . . . . ?
 C93 C94 C95 O5 -34.8(9) . . . . ?
 C95 O5 C92 C93 -20.6(9) . . . . ?
 C96 O6 C99 C98 -13.4(16) . . . . ?
 C96 C97 C98 C99 26.8(14) . . . . ?
 C97 C98 C99 O6 -9.6(16) . . . . ?
 C99 O6 C96 C97 30.5(14) . . . . ?
 C100 O7 C103 C102 34(2) . . . . ?
 C100 C101 C102 C103 -1.9(19) . . . . ?
 C101 C102 C103 O7 -18(2) . . . . ?
 C103 O7 C100 C101 -34(2) . . . . ?
 C104 O8 C107 C106 27.6(16) . . . . ?
 C104 C105 C106 C107 28(2) . . . . ?
 C107 O8 C104 C105 -12.0(18) . . . . ?

_shelx_res_file
;
    12_sqp.res created by SHELXL-2014/7

TITL jcm03720a_0m_a.res in P-1
CELL 0.71073 17.0778 17.0833 25.8714 77.107 76.123 60.217
ZERR 64 0.0011 0.0011 0.0017 0.003 0.003 0.002
LATT 1
SFAC C H Li Mo N O
UNIT 214 374 18 12 12 16
DFIX 1.54 C88 C89 C89 C90 C90 C91
DFIX 1.54 C100 C101 C101 C102 C102 C103
DFIX 1.54 C96 C97 C97 C98 C98 C99
DFIX 1.54 C92 C93 C93 C94 C94 C95
DFIX 1.54 C104 C105 C105 C106 C106 C107
DFIX 1.54 C76 C77 C77 C78 C78 C79
DFIX 1.54 C84 C85 C85 C86 C86 C87
DFIX 1.54 C80 C81 C81 C82 C82 C83
DELU O4 C88 C89 C90 C91
DELU 0 0 O7 C100 C101 C102 C103
DELU 0 0 O6 C96 C97 C98 C99
DELU O5 C92 C93 C94 C95
DELU O8 C104 C105 C106 C107
DELU O1 C76 C77 C78 C79
DELU 0 0 O3 C84 C85 C86 C87
DELU O2 C80 C81 C82 C83
SIMU O4 C88 C89 C90 C91
SIMU 0.00001 0.00002 2 O7 C100 C101 C102 C103
SIMU 0.00001 0.00002 2 O6 C96 C97 C98 C99
SIMU O5 C92 C93 C94 C95
SIMU O8 C104 C105 C106 C107
```

```
SIMU O1 C76 C77 C78 C79
SIMU 0.00001 0.00002 2 O3 C84 C85 C86 C87
SIMU O2 C80 C81 C82 C83

L.S. 40 0 59
PLAN  20
TEMP -80.15
CONF
BOND $H
MORE -1
ABIN
fmap 2
acta
OMIT 1 1 3
OMIT 2 2 3
OMIT 1 3 0
OMIT -2 -1 2
OMIT 1 0 2
OMIT 1 -1 2
OMIT -2 -1 4
REM <olex2.extras>
REM <HklSrc "%12_sqp.hkl">
REM </olex2.extras>

WGHT    0.068600    8.645101
FVAR       0.20740
MO1   4    0.519693    0.654100    0.770046    11.00000    0.02369
0.02301 =
         0.03688    0.00188   -0.00687   -0.01245
H     2    0.554928    0.542803    0.754384    11.00000   -1.50000
HA    2    0.408955    0.656014    0.791044    11.00000   -1.50000
HB    2    0.458756    0.739922    0.813202    11.00000   -1.50000
MO2   4    0.511571    0.733632    0.694789    11.00000    0.02459
0.02494 =
         0.03385   -0.00022   -0.00603   -0.01370
HC    2    0.554379    0.653531    0.636934    11.00000   -1.50000
HD    2    0.393386    0.762340    0.681705    11.00000   -1.50000
HE    2    0.450448    0.844978    0.706605    11.00000   -1.50000
MO3   4    0.238013    0.593923    0.793535    11.00000    0.02829
0.02313 =
         0.03381   -0.00669    0.00043   -0.01662
HF    2    0.326233    0.532324    0.842412    11.00000   -1.50000
HG    2    0.252671    0.690999    0.805978    11.00000   -1.50000
HH    2    0.123709    0.693691    0.775727    11.00000   -1.50000
MO4   4    0.319821    0.557594    0.719201    11.00000    0.03351
0.02601 =
         0.03630   -0.01022    0.00418   -0.02011
HI    2    0.247747    0.639557    0.671853    11.00000   -1.50000
HJ    2    0.430557    0.491765    0.734384    11.00000   -1.50000
HK    2    0.369598    0.633000    0.700230    11.00000   -1.50000
MO5   4    0.143702    0.916315    0.709195    11.00000    0.02635
0.02247 =
         0.03610   -0.00330   -0.00564   -0.01111
HL    2    0.209449    0.940603    0.644116    11.00000   -1.50000
HM    2    0.067841    0.877051    0.736555    11.00000   -1.50000
HN    2    0.233948    0.790735    0.693835    11.00000   -1.50000
MO6   4    0.188633    0.933251    0.772667    11.00000    0.02684
0.01876 =
```

```
         0.03706   -0.00462   -0.00520   -0.01070
HO    2    0.273772    0.965269    0.742271    11.00000   -1.50000
HP    2    0.278355    0.829488    0.789661    11.00000   -1.50000
HQ    2    0.136093    0.894133    0.836000    11.00000   -1.50000
LI1   3    0.506151    0.559611    0.690691    11.00000    0.04472
0.05219 =
         0.06230   -0.02001   -0.00358   -0.02570
LI2   3    0.438798    0.522194    0.800023    11.00000    0.04876
0.03938 =
         0.04117    0.00173   -0.01463   -0.02374
LI3   3    0.140644    0.740425    0.706842    11.00000    0.05509
0.04108 =
         0.04988   -0.01544   -0.01155   -0.02431
LI4   3    0.115203    0.794592    0.808059    11.00000    0.06066
0.02845 =
         0.05146   -0.01566    0.01073   -0.02594
LI5   3    0.331860    0.905506    0.675342    11.00000    0.02742
0.03046 =
         0.04968   -0.00013   -0.01109   -0.01198
LI6   3    0.388632    0.860857    0.779517    11.00000    0.03767
0.03674 =
         0.03838   -0.00951   -0.00381   -0.01585
LI7   3    0.319695    0.729568    0.836973    11.00000    0.08756
0.07968 =
         0.03228   -0.00701    0.00527   -0.01997
LI8   3    0.318514    0.725930    0.646421    11.00000    0.17035
0.07238 =
         0.04600   -0.00586   -0.00161   -0.03364
LI9   3    0.321161    0.731174    0.741785    11.00000    0.03775
0.03530 =
         0.05427   -0.01117   -0.01439   -0.01828
N1    5    0.656999    0.624409    0.768246    11.00000    0.02065
0.03147 =
         0.03635    0.00470   -0.00964   -0.01325
N2    5    0.644777    0.719215    0.689315    11.00000    0.02785
0.02502 =
         0.03766   -0.00395   -0.00283   -0.01545
N3    5    0.199784    0.489565    0.802195    11.00000    0.03265
0.02323 =
         0.02837   -0.00422   -0.00059   -0.01717
N4    5    0.293854    0.447591    0.723170    11.00000    0.04075
0.03106 =
         0.03673   -0.01319    0.00594   -0.02652
N5    5    0.030039    1.048923    0.708803    11.00000    0.02548
0.02310 =
         0.04297   -0.00082   -0.00801   -0.00848
N6    5    0.084765    1.071976    0.773802    11.00000    0.02965
0.01907 =
         0.04004   -0.00589    0.00045   -0.01324
O1    6    0.562061    0.481470    0.634866    11.00000    0.09589
0.11652 =
         0.11849   -0.08926    0.06324   -0.07993
O2    6    0.507493    0.437065    0.857712    11.00000    0.05525
0.07530 =
         0.10424    0.03510   -0.03458   -0.04469
O3    6    0.056091    0.779929    0.655824    11.00000    0.13744
0.07199 =
         0.13259   -0.01389   -0.09087   -0.03066
```

```
O4    6    0.033936    0.782320    0.875346    11.00000    0.08470
0.06041 =
         0.07153   -0.02772    0.02988   -0.04563
O5    6    0.375997    0.966604    0.610319    11.00000    0.04004
0.04606 =
         0.06255    0.02278   -0.01333   -0.02076
O6    6    0.377506    0.929497    0.833292    11.00000    0.09830
0.08955 =
         0.11571   -0.05666   -0.05260    0.00522
O7    6    0.306847    0.715757    0.912871    11.00000    0.14335
0.13360 =
         0.09379    0.02112    0.01114   -0.02717
O8    6    0.342042    0.739753    0.570156    11.00000    0.09893
0.11472 =
         0.04396    0.00250   -0.00827   -0.05276
C1    1    0.692839    0.664106    0.727975    11.00000    0.02390
0.02783 =
         0.04470   -0.00167   -0.00771   -0.01241
AFIX  43
H1    2    0.753455    0.653503    0.726357    11.00000   -1.20000
AFIX   0
C2    1    0.716483    0.554046    0.804194    11.00000    0.02389
0.02669 =
         0.05481    0.01235   -0.01588   -0.01222
C3    1    0.775445    0.468947    0.786116    11.00000    0.02747
0.02967 =
         0.07627    0.00375   -0.01120   -0.01290
C4    1    0.833936    0.401106    0.823172    11.00000    0.03467
0.03148 =
         0.09867    0.01294   -0.02036   -0.00773
AFIX  43
H4    2    0.876627    0.342935    0.812437    11.00000   -1.20000
AFIX   0
C5    1    0.830142    0.417328    0.872367    11.00000    0.04836
0.05478 =
         0.07251    0.02421   -0.02539   -0.02829
AFIX  43
H5    2    0.870437    0.371063    0.895499    11.00000   -1.20000
AFIX   0
C6    1    0.768779    0.499879    0.889518    11.00000    0.03657
0.06012 =
         0.05175    0.01281   -0.01499   -0.02273
AFIX  43
H6    2    0.765606    0.509290    0.924953    11.00000   -1.20000
AFIX   0
C7    1    0.710657    0.570655    0.855944    11.00000    0.03145
0.04643 =
         0.04892    0.01218   -0.01106   -0.02255
C8    1    0.780427    0.447743    0.732158    11.00000    0.03575
0.03970 =
         0.08948   -0.01336   -0.01193   -0.00870
AFIX  13
H8    2    0.728082    0.500428    0.715822    11.00000   -1.20000
AFIX   0
C9    1    0.867058    0.439349    0.694655    11.00000    0.06243
0.11322 =
         0.09355   -0.05196    0.01330   -0.01809
AFIX 137
```

```
H9A   2    0.867039    0.498163    0.687462    11.00000   -1.50000
H9B   2    0.869546    0.420738    0.660855    11.00000   -1.50000
H9C   2    0.920404    0.393738    0.711486    11.00000   -1.50000
AFIX   0
C10   1    0.768757    0.362805    0.734896    11.00000    0.11500
0.04035 =
         0.15568   -0.01282   -0.03792   -0.03470
AFIX 137
H10A  2    0.815870    0.310304    0.753531    11.00000   -1.50000
H10B  2    0.774716    0.350871    0.698445    11.00000   -1.50000
H10C  2    0.708280    0.373339    0.754451    11.00000   -1.50000
AFIX   0
C11   1    0.646819    0.661742    0.875856    11.00000    0.04823
0.05842 =
         0.04184   -0.00093   -0.01355   -0.02208
AFIX  13
H11   2    0.605819    0.700549    0.848442    11.00000   -1.20000
AFIX   0
C12   1    0.697507    0.710154    0.882343    11.00000    0.06814
0.08339 =
         0.14178   -0.03397   -0.00531   -0.03400
AFIX 137
H12A  2    0.742109    0.671249    0.906728    11.00000   -1.50000
H12B  2    0.653906    0.767183    0.897196    11.00000   -1.50000
H12C  2    0.729229    0.723284    0.847292    11.00000   -1.50000
AFIX   0
C13   1    0.587076    0.654872    0.928493    11.00000    0.07570
0.11689 =
         0.06617   -0.00627    0.00991   -0.03188
AFIX 137
H13A  2    0.555133    0.622294    0.925159    11.00000   -1.50000
H13B  2    0.542306    0.716045    0.937498    11.00000   -1.50000
H13C  2    0.625104    0.621830    0.956906    11.00000   -1.50000
AFIX   0
C14   1    0.679674    0.772199    0.651444    11.00000    0.02362
0.03262 =
         0.04050    0.00682   -0.00436   -0.01110
C15   1    0.722577    0.746763    0.599841    11.00000    0.04350
0.05319 =
         0.04757    0.00307   -0.00517   -0.02341
C16   1    0.748968    0.806482    0.562424    11.00000    0.05535
0.08390 =
         0.04332    0.00462    0.00307   -0.03788
AFIX  43
H16   2    0.777805    0.790703    0.527327    11.00000   -1.20000
AFIX   0
C17   1    0.734020    0.886586    0.575480    11.00000    0.07189
0.08861 =
         0.06787    0.01071   -0.00385   -0.06187
AFIX  43
H17   2    0.752444    0.925757    0.549633    11.00000   -1.20000
AFIX   0
C18   1    0.692433    0.910189    0.625896    11.00000    0.07698
0.05653 =
         0.07254    0.01343   -0.02082   -0.05077
AFIX  43
H18   2    0.681977    0.966140    0.634557    11.00000   -1.20000
AFIX   0
```

```
C19   1    0.665136    0.854044    0.664741    11.00000    0.04204
0.04292 =
         0.04612    0.00748   -0.01150   -0.03004
C20   1    0.743736    0.656891    0.584987    11.00000    0.08838
0.05371 =
         0.04701   -0.00677    0.00369   -0.02528
AFIX  13
H20   2    0.722763    0.623475    0.617313    11.00000   -1.20000
AFIX   0
C21   1    0.848368    0.597957    0.569111    11.00000    0.11573
0.08406 =
         0.16748   -0.03859    0.06018   -0.00385
AFIX 137
H21A  2    0.870030    0.627693    0.536262    11.00000   -1.50000
H21B  2    0.861119    0.537902    0.562913    11.00000   -1.50000
H21C  2    0.879863    0.591154    0.598160    11.00000   -1.50000
AFIX   0
C22   1    0.691915    0.671829    0.540865    11.00000    0.22532
0.10660 =
         0.07426   -0.01975   -0.02328   -0.10321
AFIX 137
H22A  2    0.627247    0.714059    0.550687    11.00000   -1.50000
H22B  2    0.698134    0.613677    0.535893    11.00000   -1.50000
H22C  2    0.716744    0.697443    0.507389    11.00000   -1.50000
AFIX   0
C23   1    0.623172    0.881590    0.720230    11.00000    0.06817
0.04457 =
         0.05245   -0.00361   -0.01671   -0.03375
AFIX  13
H23   2    0.601376    0.837370    0.740984    11.00000   -1.20000
AFIX   0
C24   1    0.691954    0.877577    0.749497    11.00000    0.10733
0.12882 =
         0.08431   -0.01206   -0.03370   -0.07118
AFIX 137
H24A  2    0.745607    0.817439    0.748910    11.00000   -1.50000
H24B  2    0.664459    0.888504    0.786751    11.00000   -1.50000
H24C  2    0.710423    0.924185    0.731820    11.00000   -1.50000
AFIX   0
C25   1    0.540272    0.977027    0.719392    11.00000    0.12008
0.04043 =
         0.07789   -0.01080   -0.00733   -0.03071
AFIX 137
H25A  2    0.559889    1.022014    0.699791    11.00000   -1.50000
H25B  2    0.513431    0.991003    0.756267    11.00000   -1.50000
H25C  2    0.494709    0.978832    0.701586    11.00000   -1.50000
AFIX   0
C26   1    0.236234    0.435358    0.764258    11.00000    0.03823
0.02693 =
         0.03551   -0.00119   -0.00261   -0.02286
AFIX  43
H26   2    0.221124    0.388032    0.766527    11.00000   -1.20000
AFIX   0
C27   1    0.127008    0.485339    0.841783    11.00000    0.04199
0.02850 =
         0.03489   -0.00446    0.00061   -0.02613
C28   1    0.142653    0.442097    0.893364    11.00000    0.04243
0.04582 =
```

```
         0.03872   -0.00682    0.00151   -0.02990
C29   1    0.069361    0.443753    0.931820    11.00000    0.06077
0.05206 =
         0.03585   -0.00379   -0.00116   -0.03586
AFIX  43
H29   2    0.079488    0.415008    0.967261    11.00000   -1.20000
AFIX   0
C30   1   -0.017824    0.486597    0.919149    11.00000    0.04562
0.06320 =
         0.06284   -0.01629    0.01816   -0.03640
AFIX  43
H30   2   -0.067463    0.487712    0.945746    11.00000   -1.20000
AFIX   0
C31   1   -0.032222    0.527356    0.868134    11.00000    0.03049
0.05677 =
         0.06423   -0.00456    0.00519   -0.02496
AFIX  43
H31   2   -0.092167    0.555188    0.859511    11.00000   -1.20000
AFIX   0
C32   1    0.038690    0.529461    0.828017    11.00000    0.03902
0.03174 =
         0.05071   -0.00066   -0.00359   -0.02312
C33   1    0.237686    0.390704    0.908725    11.00000    0.04768
0.07537 =
         0.04230    0.01127   -0.01404   -0.03381
AFIX  13
H33   2    0.280302    0.401084    0.877370    11.00000   -1.20000
AFIX   0
C34   1    0.244443    0.424008    0.955935    11.00000    0.07845
0.19138 =
         0.06312   -0.02838   -0.01860   -0.07015
AFIX 137
H34A  2    0.199656    0.419444    0.986578    11.00000   -1.50000
H34B  2    0.232067    0.487523    0.946473    11.00000   -1.50000
H34C  2    0.305999    0.386709    0.965449    11.00000   -1.50000
AFIX   0
C35   1    0.268279    0.290322    0.919315    11.00000    0.08276
0.07444 =
         0.16484    0.03328   -0.03248   -0.02436
AFIX 137
H35A  2    0.225635    0.278304    0.948579    11.00000   -1.50000
H35B  2    0.329508    0.258323    0.929436    11.00000   -1.50000
H35C  2    0.269646    0.268869    0.886837    11.00000   -1.50000
AFIX   0
C36   1    0.017231    0.577432    0.771948    11.00000    0.04247
0.05095 =
         0.05209    0.01012   -0.01426   -0.02982
AFIX  13
H36   2    0.073996    0.578037    0.750329    11.00000   -1.20000
AFIX   0
C37   1   -0.007239    0.523795    0.744938    11.00000    0.11984
0.12521 =
         0.07132    0.01868   -0.04663   -0.08750
AFIX 137
H37A  2    0.043912    0.462376    0.742527    11.00000   -1.50000
H37B  2   -0.019946    0.554329    0.708803    11.00000   -1.50000
H37C  2   -0.061466    0.520085    0.766063    11.00000   -1.50000
AFIX   0
```

```
C38   1   -0.056455    0.674761    0.773158    11.00000    0.05244
0.08414 =
         0.09563    0.02763   -0.02627   -0.01044
AFIX 137
H38A  2   -0.070365    0.702011    0.736788    11.00000   -1.50000
H38B  2   -0.035329    0.708967    0.786855    11.00000   -1.50000
H38C  2   -0.111550    0.676545    0.796616    11.00000   -1.50000
AFIX   0
C39   1    0.343467    0.379020    0.688558    11.00000    0.05643
0.03960 =
         0.05473   -0.01856    0.01166   -0.03656
C40   1    0.322160    0.393186    0.637212    11.00000    0.08902
0.05347 =
         0.05326   -0.01268    0.01222   -0.05568
C41   1    0.375391    0.325845    0.603353    11.00000    0.13381
0.07660 =
         0.06031   -0.03897    0.02060   -0.07082
AFIX  43
H41   2    0.360596    0.335000    0.568624    11.00000   -1.20000
AFIX   0
C42   1    0.447666    0.247762    0.619006    11.00000    0.13673
0.07317 =
         0.09417   -0.06018    0.06094   -0.07450
AFIX  43
H42   2    0.482533    0.202479    0.595468    11.00000   -1.20000
AFIX   0
C43   1    0.470770    0.233753    0.668721    11.00000    0.07918
0.04441 =
         0.09332   -0.03643    0.02541   -0.03158
AFIX  43
H43   2    0.522447    0.179138    0.678898    11.00000   -1.20000
AFIX   0
C44   1    0.419466    0.298671    0.705032    11.00000    0.05618
0.03666 =
         0.08177   -0.02854    0.01634   -0.02556
C45   1    0.240379    0.479245    0.617406    11.00000    0.11809
0.06734 =
         0.04627   -0.00714   -0.01057   -0.06129
AFIX  13
H45   2    0.212854    0.521243    0.645555    11.00000   -1.20000
AFIX   0
C46   1    0.271086    0.527571    0.565937    11.00000    0.18804
0.09609 =
         0.05762   -0.00304    0.00314   -0.08334
AFIX 137
H46A  2    0.311581    0.547600    0.573363    11.00000   -1.50000
H46B  2    0.217540    0.580452    0.552737    11.00000   -1.50000
H46C  2    0.303834    0.485659    0.538697    11.00000   -1.50000
AFIX   0
C47   1    0.167108    0.459612    0.608858    11.00000    0.14655
0.12841 =
         0.10884   -0.01314   -0.03390   -0.09898
AFIX 137
H47A  2    0.195297    0.408298    0.587946    11.00000   -1.50000
H47B  2    0.122482    0.513397    0.589402    11.00000   -1.50000
H47C  2    0.136298    0.444483    0.643716    11.00000   -1.50000
AFIX   0
```

```
C48   1    0.447739    0.280330    0.759477    11.00000    0.05516
0.03746 =
         0.10926   -0.02311   -0.01039   -0.00976
AFIX  13
H48   2    0.410139    0.337714    0.776540    11.00000   -1.20000
AFIX   0
C49   1    0.424293    0.206523    0.795730    11.00000    0.16475
0.05007 =
         0.10763   -0.00210   -0.01696   -0.04758
AFIX 137
H49A  2    0.362339    0.220498    0.792887    11.00000   -1.50000
H49B  2    0.427918    0.205754    0.833088    11.00000   -1.50000
H49C  2    0.467861    0.146854    0.784035    11.00000   -1.50000
AFIX   0
C50   1    0.547215    0.256565    0.754910    11.00000    0.05812
0.14757 =
         0.20491   -0.08392   -0.02762   -0.00333
AFIX 137
H50A  2    0.585979    0.202133    0.736656    11.00000   -1.50000
H50B  2    0.562896    0.244861    0.790867    11.00000   -1.50000
H50C  2    0.556983    0.307358    0.734258    11.00000   -1.50000
AFIX   0
C51   1    0.023678    1.103015    0.741521    11.00000    0.02601
0.02153 =
         0.04877    0.00008   -0.00367   -0.01137
AFIX  43
H51   2   -0.024644    1.163544    0.741506    11.00000   -1.20000
AFIX   0
C52   1   -0.047677    1.078925    0.683567    11.00000    0.03552
0.02672 =
         0.05358   -0.00333   -0.01661   -0.00527
C53   1   -0.047041    1.115315    0.629747    11.00000    0.05612
0.06070 =
         0.06687    0.00048   -0.02868   -0.02005
C54   1   -0.120465    1.137206    0.604830    11.00000    0.08655
0.09794 =
         0.07105    0.01742   -0.04560   -0.02942
AFIX  43
H54   2   -0.119868    1.160757    0.567904    11.00000   -1.20000
AFIX   0
C55   1   -0.193899    1.125157    0.633018    11.00000    0.05997
0.10061 =
         0.09540    0.00068   -0.04388   -0.01962
AFIX  43
H55   2   -0.243096    1.139091    0.615541    11.00000   -1.20000
AFIX   0
C56   1   -0.195250    1.092949    0.686390    11.00000    0.03435
0.08044 =
         0.09676   -0.01475   -0.01704   -0.02227
AFIX  43
H56   2   -0.247162    1.087246    0.706115    11.00000   -1.20000
AFIX   0
C57   1   -0.122540    1.068323    0.712534    11.00000    0.02992
0.04225 =
         0.07152   -0.01069   -0.01465   -0.00701
C58   1    0.033136    1.130494    0.597519    11.00000    0.07319
0.07811 =
         0.05176    0.01213   -0.01441   -0.03238
```

```
AFIX  13
H58   2    0.077006    1.113380    0.622631    11.00000   -1.20000
AFIX   0
C59   1    0.083560    1.070417    0.553002    11.00000    0.10608
0.12763 =
         0.07852   -0.01684    0.00492   -0.04118
AFIX 137
H59A  2    0.107677    1.006473    0.568495    11.00000   -1.50000
H59B  2    0.134102    1.082100    0.533224    11.00000   -1.50000
H59C  2    0.041519    1.083813    0.528490    11.00000   -1.50000
AFIX   0
C60   1    0.002229    1.231213    0.574728    11.00000    0.15676
0.09284 =
         0.10866    0.04137   -0.03449   -0.06755
AFIX 137
H60A  2   -0.032353    1.247169    0.545427    11.00000   -1.50000
H60B  2    0.055936    1.240789    0.561127    11.00000   -1.50000
H60C  2   -0.036524    1.269646    0.603158    11.00000   -1.50000
AFIX   0
C61   1   -0.126217    1.031180    0.771128    11.00000    0.03217
0.05270 =
         0.06809   -0.00304   -0.00651   -0.02429
AFIX  13
H61   2   -0.064199    1.007452    0.780610    11.00000   -1.20000
AFIX   0
C62   1   -0.193180    1.105490    0.806124    11.00000    0.07061
0.08475 =
         0.08445   -0.02668    0.02345   -0.03324
AFIX 137
H62A  2   -0.177022    1.154978    0.798290    11.00000   -1.50000
H62B  2   -0.190536    1.080480    0.844008    11.00000   -1.50000
H62C  2   -0.255129    1.128895    0.798624    11.00000   -1.50000
AFIX   0
C63   1   -0.148993    0.950982    0.783684    11.00000    0.07530
0.08127 =
         0.08906    0.00059   -0.01332   -0.05495
AFIX 137
H63A  2   -0.209381    0.971938    0.774528    11.00000   -1.50000
H63B  2   -0.148974    0.928197    0.821965    11.00000   -1.50000
H63C  2   -0.103020    0.902122    0.762493    11.00000   -1.50000
AFIX   0
C64   1    0.084164    1.136119    0.800553    11.00000    0.03260
0.02010 =
         0.05468   -0.01103   -0.00491   -0.00744
C65   1    0.045810    1.143510    0.854798    11.00000    0.04050
0.03487 =
         0.06206   -0.01774   -0.00158   -0.01152
C66   1    0.048191    1.207225    0.879610    11.00000    0.06125
0.04567 =
         0.06238   -0.02307   -0.00213   -0.01609
AFIX  43
H66   2    0.022436    1.212799    0.916311    11.00000   -1.20000
AFIX   0
C67   1    0.086069    1.261085    0.852884    11.00000    0.06557
0.04507 =
         0.09041   -0.02943   -0.00873   -0.02432
AFIX  43
H67   2    0.086522    1.303939    0.870579    11.00000   -1.20000
```

```
AFIX   0
C68   1    0.124076    1.252894    0.799503    11.00000    0.05949
0.03747 =
         0.09483   -0.01529   -0.00539   -0.03141
AFIX  43
H68   2    0.149996    1.291227    0.780819    11.00000   -1.20000
AFIX   0
C69   1    0.125605    1.190745    0.772411    11.00000    0.03118
0.02278 =
         0.06969   -0.00995   -0.00462   -0.00823
C70   1    0.004297    1.084299    0.885861    11.00000    0.06947
0.04855 =
         0.04938   -0.02301    0.00807   -0.03023
AFIX  13
H70   2    0.004486    1.048417    0.860083    11.00000   -1.20000
AFIX   0
C71   1    0.062735    1.015535    0.929186    11.00000    0.15421
0.07756 =
         0.05745    0.00514   -0.01745   -0.05709
AFIX 137
H71A  2    0.125700    0.980417    0.912140    11.00000   -1.50000
H71B  2    0.037335    0.974211    0.946836    11.00000   -1.50000
H71C  2    0.062011    1.048626    0.955893    11.00000   -1.50000
AFIX   0
C72   1   -0.096359    1.140340    0.911464    11.00000    0.08851
0.09461 =
         0.13486   -0.05214    0.04358   -0.05422
AFIX 137
H72A  2   -0.098679    1.175159    0.937711    11.00000   -1.50000
H72B  2   -0.121670    1.099065    0.929445    11.00000   -1.50000
H72C  2   -0.132240    1.182158    0.883391    11.00000   -1.50000
AFIX   0
C73   1    0.172270    1.181353    0.715179    11.00000    0.05208
0.03007 =
         0.07514   -0.00557    0.00595   -0.02648
AFIX  13
H73   2    0.180302    1.123465    0.706349    11.00000   -1.20000
AFIX   0
C74   1    0.267482    1.174390    0.706408    11.00000    0.05166
0.09208 =
         0.09715   -0.00654    0.00661   -0.04790
AFIX 137
H74A  2    0.262731    1.228023    0.717487    11.00000   -1.50000
H74B  2    0.293450    1.171157    0.668334    11.00000   -1.50000
H74C  2    0.307101    1.119541    0.727798    11.00000   -1.50000
AFIX   0
C75   1    0.113048    1.258360    0.676775    11.00000    0.08776
0.05384 =
         0.07603    0.00144   -0.00423   -0.02699
AFIX 137
H75A  2    0.060196    1.251057    0.675425    11.00000   -1.50000
H75B  2    0.148805    1.256905    0.640849    11.00000   -1.50000
H75C  2    0.092019    1.316689    0.689331    11.00000   -1.50000
AFIX   0
C76   1    0.643936    0.467395    0.603097    11.00000    0.09707
0.15802 =
         0.18589   -0.10706    0.06572   -0.07232
AFIX  23
```

```
H76A  2    0.687215    0.462189    0.624778    11.00000   -1.20000
H76B  2    0.634804    0.518781    0.574226    11.00000   -1.20000
AFIX   0
C77   1    0.679770    0.382736    0.580056    11.00000    0.15188
0.13222 =
        0.18260   -0.09205    0.07286   -0.05896
AFIX  23
H77A  2    0.715884    0.329082    0.604259    11.00000   -1.20000
H77B  2    0.717887    0.383356    0.544633    11.00000   -1.20000
AFIX   0
C78   1    0.593463    0.384073    0.575100    11.00000    0.21158
0.22638 =
        0.20182   -0.17154    0.10192   -0.15028
AFIX  23
H78A  2    0.580281    0.405452    0.537810    11.00000   -1.20000
H78B  2    0.598054    0.322720    0.585627    11.00000   -1.20000
AFIX   0
C79   1    0.522881    0.447013    0.611557    11.00000    0.15356
0.21794 =
        0.18089   -0.14859    0.07603   -0.13641
AFIX  23
H79A  2    0.474875    0.497118    0.591599    11.00000   -1.20000
H79B  2    0.494337    0.415076    0.639621    11.00000   -1.20000
AFIX   0
C80   1    0.589875    0.422591    0.870601    11.00000    0.07353
0.10460 =
        0.16271    0.04269   -0.05822   -0.05934
AFIX  23
H80A  2    0.577201    0.459102    0.899241    11.00000   -1.20000
H80B  2    0.625960    0.440077    0.838668    11.00000   -1.20000
AFIX   0
C81   1    0.639985    0.324807    0.889094    11.00000    0.07910
0.12280 =
        0.19306    0.06493   -0.04926   -0.02902
AFIX  23
H81A  2    0.677399    0.313722    0.916407    11.00000   -1.20000
H81B  2    0.680123    0.290230    0.858789    11.00000   -1.20000
AFIX   0
C82   1    0.566492    0.299654    0.912397    11.00000    0.13654
0.10887 =
        0.20782    0.08099   -0.07990   -0.06975
AFIX  23
H82A  2    0.583397    0.239410    0.903209    11.00000   -1.20000
H82B  2    0.554622    0.297418    0.951944    11.00000   -1.20000
AFIX   0
C83   1    0.486143    0.370265    0.889042    11.00000    0.08669
0.07111 =
        0.13545    0.04200   -0.03249   -0.05287
AFIX  23
H83A  2    0.467988    0.343803    0.866605    11.00000   -1.20000
H83B  2    0.434519    0.397266    0.917860    11.00000   -1.20000
AFIX   0
C84   1   -0.002373    0.869817    0.638101    11.00000    0.13746
0.07201 =
        0.13261   -0.01387   -0.09085   -0.03063
AFIX  23
H84A  2    0.033722    0.902038    0.620583    11.00000   -1.20000
H84B  2   -0.044504    0.901075    0.669138    11.00000   -1.20000
```

```
AFIX   0
C85   1   -0.052785    0.872455    0.601619    11.00000    0.13747
0.07203 =
         0.13262   -0.01386   -0.09084   -0.03061
AFIX  23
H85A  2   -0.039129    0.903195    0.566048    11.00000   -1.20000
H85B  2   -0.119079    0.905360    0.614591    11.00000   -1.20000
AFIX   0
C86   1   -0.023938    0.775530    0.598213    11.00000    0.13747
0.07202 =
         0.13262   -0.01386   -0.09085   -0.03062
AFIX  23
H86A  2   -0.075502    0.761864    0.613164    11.00000   -1.20000
H86B  2   -0.003618    0.763207    0.560362    11.00000   -1.20000
AFIX   0
C87   1    0.051107    0.719610    0.629192    11.00000    0.13746
0.07201 =
         0.13260   -0.01388   -0.09086   -0.03064
AFIX  23
H87A  2    0.038892    0.673494    0.655363    11.00000   -1.20000
H87B  2    0.109063    0.688094    0.605190    11.00000   -1.20000
AFIX   0
C88   1   -0.021821    0.853378    0.907934    11.00000    0.08964
0.07909 =
         0.08557   -0.04029    0.03325   -0.04059
AFIX  23
H88A  2    0.013852    0.880779    0.914155    11.00000   -1.20000
H88B  2   -0.074059    0.901412    0.890228    11.00000   -1.20000
AFIX   0
C89   1   -0.053322    0.814358    0.957194    11.00000    0.26217
0.12269 =
         0.08536   -0.04312    0.07966   -0.07352
AFIX  23
H89A  2   -0.119470    0.853050    0.967966    11.00000   -1.20000
H89B  2   -0.020618    0.808689    0.985766    11.00000   -1.20000
AFIX   0
C90   1   -0.035755    0.724498    0.949143    11.00000    0.28594
0.11841 =
         0.13943   -0.03394    0.12783   -0.10961
AFIX  23
H90A  2   -0.004050    0.677783    0.977831    11.00000   -1.20000
H90B  2   -0.093674    0.724267    0.949320    11.00000   -1.20000
AFIX   0
C91   1    0.021492    0.706845    0.897291    11.00000    0.19363
0.09350 =
         0.10758   -0.03665    0.07576   -0.10463
AFIX  23
H91A  2    0.081173    0.652578    0.901563    11.00000   -1.20000
H91B  2   -0.008118    0.695321    0.873388    11.00000   -1.20000
AFIX   0
C92   1    0.319034    1.050999    0.583030    11.00000    0.05891
0.04737 =
         0.07640    0.01885   -0.02560   -0.02095
AFIX  23
H92A  2    0.266060    1.087763    0.608457    11.00000   -1.20000
H92B  2    0.296021    1.040971    0.554744    11.00000   -1.20000
AFIX   0
```

```
C93   1    0.375355    1.100364    0.558115    11.00000    0.08978
0.04772 =
         0.06048    0.01364   -0.01171   -0.03275
AFIX  23
H93A  2    0.380493    1.110244    0.518518    11.00000   -1.20000
H93B  2    0.347619    1.159881    0.571585    11.00000   -1.20000
AFIX   0
C94   1    0.466570    1.038275    0.575284    11.00000    0.08468
0.07691 =
         0.05572    0.01788   -0.02307   -0.05798
AFIX  23
H94A  2    0.472161    1.056867    0.607270    11.00000   -1.20000
H94B  2    0.516349    1.037580    0.546061    11.00000   -1.20000
AFIX   0
C95   1    0.469067    0.946138    0.588213    11.00000    0.04319
0.05124 =
         0.05080    0.01261   -0.00174   -0.02364
AFIX  23
H95A  2    0.489357    0.916057    0.555431    11.00000   -1.20000
H95B  2    0.510617    0.906190    0.614640    11.00000   -1.20000
AFIX   0
C96   1    0.439728    0.897731    0.868548    11.00000    0.09832
0.08958 =
         0.11572   -0.05665   -0.05259    0.00524
AFIX  23
H96A  2    0.485834    0.919375    0.854643    11.00000   -1.20000
H96B  2    0.471341    0.830470    0.874487    11.00000   -1.20000
AFIX   0
C97   1    0.382857    0.936228    0.919185    11.00000    0.09834
0.08960 =
         0.11574   -0.05663   -0.05257    0.00525
AFIX  23
H97A  2    0.355066    0.898091    0.940826    11.00000   -1.20000
H97B  2    0.419293    0.942346    0.941106    11.00000   -1.20000
AFIX   0
C98   1    0.312682    1.025988    0.898884    11.00000    0.09833
0.08959 =
         0.11573   -0.05664   -0.05258    0.00524
AFIX  23
H98A  2    0.253266    1.043523    0.922726    11.00000   -1.20000
H98B  2    0.331335    1.073483    0.895607    11.00000   -1.20000
AFIX   0
C99   1    0.307669    1.012147    0.846585    11.00000    0.09832
0.08957 =
         0.11572   -0.05665   -0.05259    0.00523
AFIX  23
H99A  2    0.248329    1.014459    0.847331    11.00000   -1.20000
H99B  2    0.311972    1.061194    0.819223    11.00000   -1.20000
AFIX   0
C100  1    0.234840    0.750554    0.947186    11.00000    0.14336
0.13361 =
         0.09381    0.02113    0.01115   -0.02716
AFIX  23
H10D  2    0.180649    0.789264    0.928896    11.00000   -1.20000
H10E  2    0.240825    0.788129    0.968590    11.00000   -1.20000
AFIX   0
C101  1    0.225854    0.671565    0.982695    11.00000    0.14336
0.13361 =
```

```
         0.09381    0.02113    0.01115   -0.02717
AFIX  23
H10F  2    0.204977    0.683005    1.020693    11.00000   -1.20000
H10G  2    0.183380    0.657833    0.970949    11.00000   -1.20000
AFIX   0
C102  1    0.320853    0.597835    0.974510    11.00000    0.14336
0.13361 =
         0.09381    0.02113    0.01115   -0.02716
AFIX  23
H10H  2    0.346427    0.574461    1.008550    11.00000   -1.20000
H10I  2    0.323868    0.546991    0.960340    11.00000   -1.20000
AFIX   0
C103  1    0.367016    0.637768    0.938012    11.00000    0.14336
0.13361 =
         0.09380    0.02113    0.01115   -0.02717
AFIX  23
H10J  2    0.402833    0.651008    0.956498    11.00000   -1.20000
H10K  2    0.409891    0.595907    0.911376    11.00000   -1.20000
AFIX   0
C104  1    0.267824    0.785788    0.544006    11.00000    0.12473
0.16187 =
         0.10331   -0.03166   -0.03004   -0.06947
AFIX  23
H10L  2    0.212829    0.821161    0.568842    11.00000   -1.20000
H10M  2    0.257075    0.742368    0.530763    11.00000   -1.20000
AFIX   0
C107  1    0.417157    0.751237    0.539876    11.00000    0.13476
0.25741 =
         0.08213   -0.05750    0.00463   -0.11523
AFIX  23
H10N  2    0.456059    0.703509    0.515922    11.00000   -1.20000
H10O  2    0.454778    0.752655    0.563065    11.00000   -1.20000
AFIX   0
C105  1    0.285386    0.847331    0.498626    11.00000    0.27285
0.14542 =
         0.09855    0.02176   -0.06785   -0.04148
AFIX  23
H10P  2    0.295445    0.826304    0.463782    11.00000   -1.20000
H10Q  2    0.234928    0.910332    0.498907    11.00000   -1.20000
AFIX   0
C106  1    0.367716    0.839992    0.509861    11.00000    0.28911
0.18477 =
         0.14847   -0.02550    0.05951   -0.17167
AFIX  23
H10R  2    0.352144    0.888733    0.531100    11.00000   -1.20000
H10S  2    0.406514    0.847001    0.475839    11.00000   -1.20000
AFIX   0
HKLF 4

REM  jcm03720a_0m_a.res in P-1
REM R1 =  0.0756 for   18339 Fo > 4sig(Fo)  and  0.1379 for all
31329 data
REM   1303 parameters refined using    314 restraints

END

WGHT      0.0685      8.6386
```

```
REM Highest difference peak  2.108,  deepest hole -1.523,  1-sigma
level  0.124
Q1    1   0.4144  0.9985  0.8872  11.00000  0.05    0.81
Q2    1   0.2042  0.9440  0.7194  11.00000  0.05    0.79
Q3    1   0.3278  0.7230  0.9797  11.00000  0.05    0.71
Q4    1   0.0375  0.7759  0.5744  11.00000  0.05    0.70
Q5    1   0.5193  0.6611  0.7013  11.00000  0.05    0.69
Q6    1   0.4384  0.7712  0.6594  11.00000  0.05    0.69
Q7    1   0.1378  0.9006  0.7649  11.00000  0.05    0.67
Q8    1   0.5196  0.7093  0.7551  11.00000  0.05    0.63
Q9    1   0.0622  0.7649  0.5722  11.00000  0.05    0.62
Q10   1   0.0977  1.0169  0.7751  11.00000  0.05    0.61
Q11   1   0.3004  0.9422  0.9313  11.00000  0.05    0.61
Q12   1   0.4997  0.7817  0.7430  11.00000  0.05    0.60
Q13   1   0.4353  0.5116  0.7271  11.00000  0.05    0.60
Q14   1   0.3003  0.7195  0.9983  11.00000  0.05    0.58
Q15   1   0.2555  0.5386  0.7057  11.00000  0.05    0.57
Q16   1   0.3237  0.5849  0.7729  11.00000  0.05    0.57
Q17   1   0.3580  0.8250  0.4868  11.00000  0.05    0.55
Q18   1   0.4593  0.7456  0.7431  11.00000  0.05    0.55
Q19   1   0.2225  0.6120  0.7331  11.00000  0.05    0.54
Q20   1   0.6019  0.7171  0.6792  11.00000  0.05    0.54
;
_shelx_res_checksum              62541
_shelx_hkl_file
;
```
