# Supplementary material for: Coordination of LiH Molecules to Mo≣Mo Bonds: Experimental and Computational Studies on Mo2LiH2, Mo2Li2H4, and Mo6Li9H18 Clusters
Source: J Am Chem Soc. 2021 Mar 23;143(13):5222–30. doi: 10.1021/jacs.1c01602 (PMC9157502; doi:10.1021/jacs.1c01602)
Supplement: Supplementary file 2 — ja1c01602_si_006.pdf [file ja1c01602_si_006.pdf]

SUPPORTING INFORMATION

Coordination of LiH Molecules to Mo≣Mo Bonds. Experimental and Computational Studies on Mo_2LiH_2, $Mo_2Li_2H_4$ and $Mo_6Li_9H_{18}$ Clusters.

Marina Perez-Jimenez,[a] Natalia Curado,[a] Celia Maya,[a] Jesus Campos,[a] Jesus Jover,[b] Santiago Alvarez*[b] and Ernesto Carmona*[a]

pages

1. Figure S1. Solid-state molecular structure of complex **2·IMe₄** 1
2. Figure S2. Solid-state molecular structure of complex **3·thf** 2
3. Tables S1 and S2 3
4. Attempts to generate the putative complex $\{Mo_2(H)_8[Li(thf)]_4\}$ 4
5. Organization of Mo_6, Li_6 and the three H_6 polyhedra around the Li_3 central unit of complex **5·thf**. Figure S3 5-6
6. X-ray structural characterization of new compounds 7-9
7. Synthesis and characterisation of new complexes 10-22
8. NMR spectra of new complexes 23-40
9. IR spectra 41-44
10. Computational details 45
11. Cartesian coordinates of computed complexes in xyz format 46-50
12. References 51

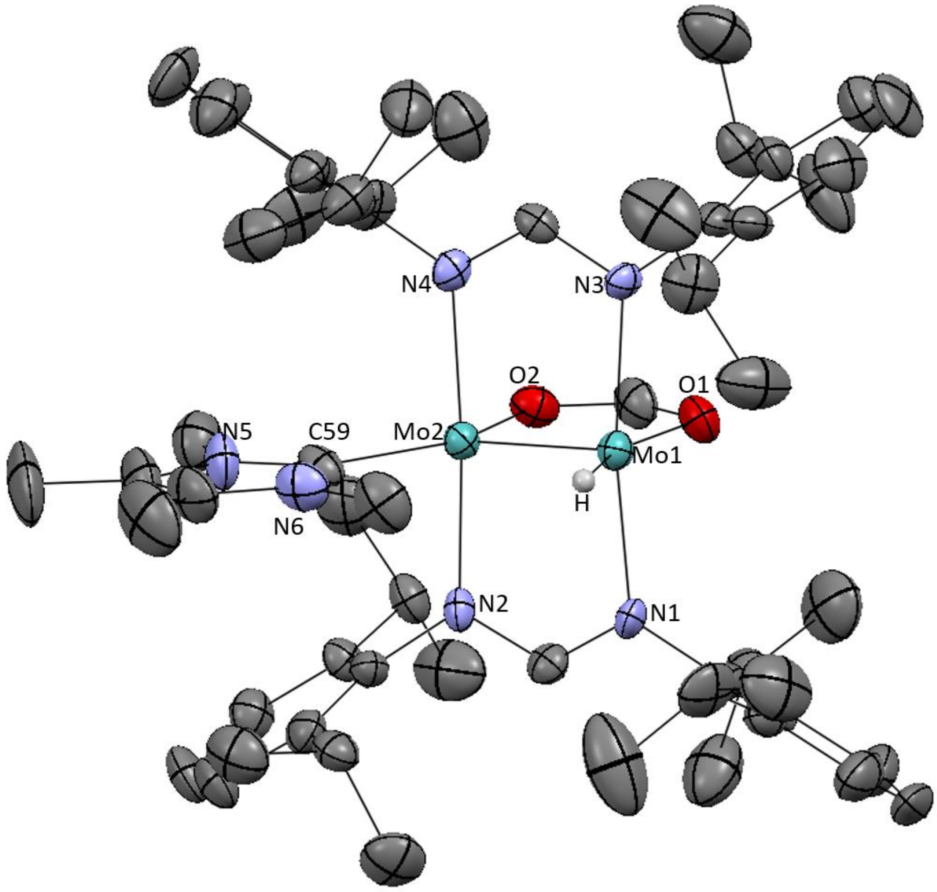

Figure S1. Solid-state molecular structure of complex **2·IMe$_4$**

Selected bond distances:
d(Mo1–Mo2) = 2.119(1) Å, d(Mo1–H) = 1.79(8) Å, d(Mo1–O1) = 2.209(4) Å, d(Mo2–O2) = 2.197(4) Å, d(Mo2–C59) = 2.275(7) Å.

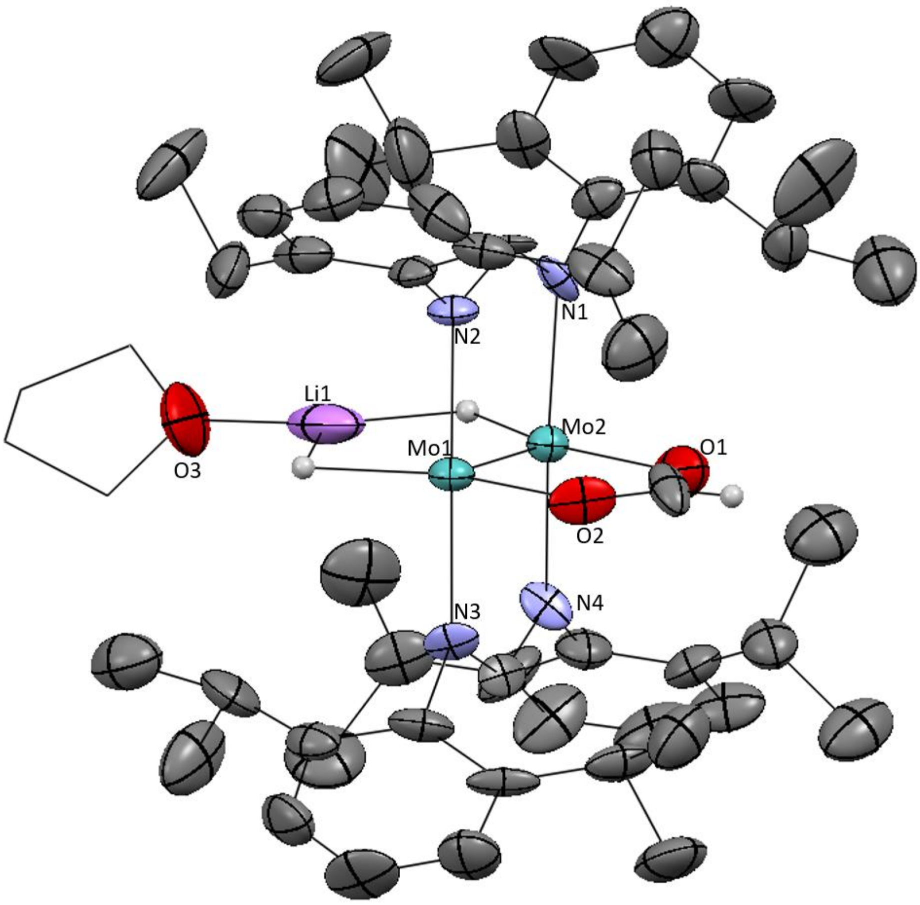

Figure S2. Solid-state molecular structure of complex **3·thf**

Selected bond distances:
d(Mo1–Mo2) = 2.092(2) Å, d(Mo1–Li1) = 2.85(4) Å, d(Mo2–Li1) = 2.89(4) Å, d(Mo1/Mo2–H) = 1.9 Å, d(Li1–H) = 2.1 Å, d(Mo2–O1) = 2.13(1) Å, d(Mo1–O2) = 2.191(9) Å.

Table S1. Experimental and calculated bond distances (Å) of the Mo_2H–Li–H core in **3·thf** and **4·thf**.

	3·thf	4·thf	
	calcd.	calcd.	exp.
Mo–Mo	2.127	2.134	2.1006 (7)
Mo–H	1.814	1.852	2.05
Li–H	1.783	1.784	1.74
		1.787	1.85
Mo–Li	2.945	2.968	2.91 (2)
		2.971	2.97 (2)

Table S2. Geometrical parameters of **5·thf**, distances in Å, angles in degrees.

	calcd.	exp.
Li9–H^{cent}	1.97 - 2.07	1.81 - 2.09
Li7/8–H^{cent} (sum 1.59)	1.87 - 1.92	1.67 - 2.20
Li1-6–H	1.90 - 2.11	1.74 - 2.29
Mo–H^{cent} (sum 1.85)	1.83 - 1.84	1.67 - 2.04
Mo–H^{ext}	1.81 - 1.82	1.70 - 2.08
Mo–H^{int}	1.83 - 1.84	1.61 - 1.97
Li9–Li7/8 (sum 2.56 Å)	2.44 - 2.46	2.45 - 2.50
Li1–Li2; Li3–Li4; Li5–Li6	2.87 - 2.90	2.83 - 2.90
Li9–Li1-6	3.26 - 3.34	3.14 - 3.37
Li7/8–Li1-6	3.17 - 3.25	2.95 - 3.47
Mo–Mo (sum 3.08)	2.14 - 2.15	2.10
Mo–Li9 (sum 2.82 Å)	3.21 - 3.25	3.15 - 3.24
Mo–Li7/8	3.39 - 3.53	3.04 - 3.90
Mo–Li1-6	3.03 - 3.10	2.99 - 3.13
Li7–Li9–Li8	176.5	176.3

Attempts to generate the putative {$Mo_2(H)_8[Li(thf)]_4$} complex

The essayed trials to generate the unknown {$Mo_2(H)_8[Li(thf)]_4$} complex included reaction of [$Mo_2(O_2CCH_3)_4$] with $LiAlH_4$, as well as the treatment of the octamethyl dimolybdate tetralithium complex with $LiAlH_4$, $PhSiH_3$ and H_2. Unfortunately, these efforts proved fruitless. Slow addition of $LiAlH_4$ (8 equiv.) at -40 °C to a suspension of [$Mo_2(O_2CCH_3)_4$] in thf afforded a black precipitate that was insoluble in thf, benzene or toluene. No reaction occured upon adding the same reagent to the [$Li(OEt_2)_4$][$Mo_2(CH_3)_8$] complex in thf and stirring the solution at either 25 °C or 50 °C for several hours. The latter complex was also stirred in thf under H_2 atmosphere (1 bar) for 24 hours at 25 °C without any changes being observed. The reaction of [$Li(OEt_2)_4$][$Mo_2(CH_3)_8$] with an excess of $PhSiH_3$ (8 equiv.) led mostly to unreacted starting material and an undetermined mixture of silane by-products. Similarly, reaction of complexes **3·thf** - **5·thf** with $LiAlH_4$ led to decomposition, once again with elimination of $LiAd^{Dipp2}$.

Organization of the Mo_6, Li_6 and three H_6 polyhedra around the Li_3 central unit of complex 5·thf

It is interesting to analyze how the approximate C_3 symmetry around the central Li_3 rod propagates through the concentric rings: $Li_3 \subset H_6^{cent} \subset (Mo_2)_3 \subset H_6^{ext} \subset H_6^{int} \subset Li_6$, where the symbol $\subset$ means "is contained in". Moreover, the C_2 axes of the LiH_6 octahedron passing through the edges spanned by Mo–Mo groups are retained, leading to the effective D_3 symmetry already observed in the NMR spectra, thus pointing to a chiral structure of the cluster, both in the solid state and in solution. The fact that **5·thf** crystallizes in the centrosymmetric P-1 space group also implies that the clusters exist as a racemic mixture of the two enantiomers.

In spite of the conservation of the trigonal symmetry, however, the successive polyhedra present different shapes, as shown by the shape maps of Figure S3 (left). There it can be seen that the shapes of four of the five groups can be roughly described as being along the minimal distortion path[1] from the octahedron (H_6^{cent}) to flattened trigonal prisms (Li_6 and Mo_6). A flattened trigonal prism is a shape (f-TPR) taken *ex profeso* as a reference in the study of the first and second coordination spheres of tris(chelated) six-coordinated metal complexes,[2] topologically equivalent to the $Li(Mo_2H_2)_3$ unit (Figure 7 main text, right). The H_6^{int} group, however, is far from such a minimal distortion path, apparently because it is much flatter than our f-TPR reference shape. In fact, it is close to planarity, and can be better described as being along the distortion path from the planar hexagon (HP-6) to the f-TPR-6 (Figure S3, right).

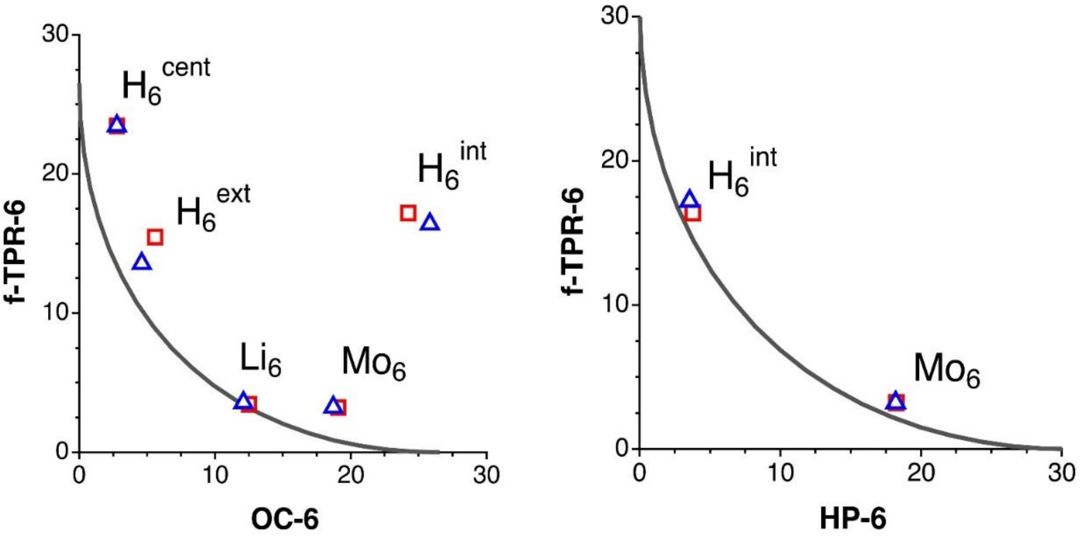

Figure S3: Shape maps that show the position of the concentric groups of the $Mo_6Li_9H_{18}$ core of **5·thf** relative to the octahedron (OC-6) to flattened trigonal prism (fTPR-6), left, and the hexagonal planar (HP-6) to fTPR-6, right, minimal distortion paths. Experimental and computational data represented by squares and triangles, respectively.

X-ray structural characterization of new compounds

Single crystals of suitable size, coated with dry perfluoropolyether or FLOMBLIN oil were mounted on a glass fiber and fixed in a cold nitrogen stream [T = 193 K] to the goniometer head. Data collection was performed on a Bruker SMART APEX II CCD area detector on a D8 goniometer, using graphite-monochromated and 0.5 mm-Monocap-collimated Mo-Kα radiation (λ = 0.71073 Å) for complex **3·thf** and a Bruker D8 Quest APEX-III CCD area detector PhotonIII using monochromatic radiation λ (Mo Kα1) = 0.71073 Å by a IµS 3.0 microfocus X-ray source for complexes **2·IMe$_4$**, **4·thf**, **5·thf**. Data collections were processed with APEX-W2D-NT (Bruker, 2004), cell refinement and data reduction with SAINT-Plus (Bruker, 2004) and the absorption was corrected by multiscan method applied by SADABS[3]. The space-group assignment was based upon systematic absences, E statistics, and successful refinement of the structure. The structure was solved by direct methods and expanded through successive difference Fourier maps, F^2 (SHELXTL)[4]. In the last cycles of refinement, ordered non-hydrogen atoms were refined anisotropically. Hydrogen atoms connected to carbon atoms were included in idealised positions, and a riding model was used for their refinement. Hydride atoms of complexes **5·thf** and **2·IMe$_4$** were located in the Fourier Map and refined freely. Hydrides of complexes **3·thf** and **4·thf** were located in the Fourier Map and their positions were fixed. In structure **5·thf**, the option SQUEEZE of the program package PLATON[5] was used to create an hkl file taking into account the residual electron density in the void areas that showed residual electron density due to heavily disordered thf solvent molecules which could not be modelled.

	**2·IMe$_4$**	**3·thf**
Formula	$C_{65}H_{94}Mo_2N_6O_3$	$C_{55}H_{81}LiMo_2N_4O_3$
fw	1199.34	1045.05
Crystal size, mm	0.21x0.12x0.1	0.25x0.21x0.17
Crystal system	Monoclinic	Monoclinic
Space group	P 1 21/n 1	P 21/c
a, Å	12.2265(13)	11.8411(17)
b, Å	21.140(3)	34.818(5)
c, Å	25.890(3)	17.612(3)
α, deg	90	90
β, deg	93.096(5)	105.388(5)
γ, deg	90	90
V, Å^3	6682.0(13)	7000.9(18)
T, K	193.0	173(2)
Z	4	4
ρ_{calc}, g·cm^{-3}	1.192	0.992
μ, mm^{-1} (Mo1Kα)	0.420	0.392
F (000)	2536	2200
Absoption correction	multi-scan 0.6612 - 0.7452	multi-scan 0.6129 - 0.7454
θ range, deg	1.926 - 24.779	1.170 - 26.462
No. of rflns measd	11403	13053
R_{int}	0.1752	0.1343
No. of rlfns unique	11403	13053
No. of params/restraints	713/40	608/8
R1 (I > 2σ(I))	0.0705	0.1359
R1 (all data)	0.1596	0.1771
wR2 (I > 2σ(I))	0.1307	0.3393
wR2 (all data)	0.1696	0.3675
Diff. Fourier peaks min/max, eÅ^{-3}	-0.638/0.730	-2.216/1.430
CCDC number	2059186	2059185

	**4·thf**	**5·thf**
Formula	$C_{29}H_{45}LiMoN_2O$	$C_{107}H_{187}Li_9\ Mo_6N_6O_8$
fw	540.55	2323.74
Crystal size, mm	0.18x0.16x0.13	0.28x0.25x0.10
Crystal system	Tetragonal	Triclinic
Space group	I 41 c d	P -1
a, Å	17.1105(5)	17.0778(11)
b, Å	17.1105(5)	17.0833(11)
c, Å	48.188(2)	25.8714(17)
α, deg	90	77.107(3)
β, deg	90	76.123(3)
γ, deg	90	60.217(2)
V, $Å^3$	14108.0(11)	6311.1(7)
T, K	193(2)	193(2)
Z	16	2
ρ_{calc}, $g·cm^{-3}$	1.018	1.223
μ, mm^{-1} (Mo1Kα)	0.390	0.626
F (000)	4576	2428
Absoption correction	multi-scan 0.5998 - 0.7452	multi-scan 0.6666 - 0.7457
θ range, deg	1.883 - 25.355	2.007- 28.314
No. of rflns measd	6457	31329
R_{int}	0.0345	0.1242
No. of rlfns unique	6457	31329
No. of params/restraints	316/2	1303/ 314
R1 (I > 2σ(I))	0.0548	0.0756
R1 (all data)	0.0606	0.1379
wR2 (I > 2σ(I))	0.1369	0.1548
wR2 (all data)	0.1431	0.1808
Diff. Fourier peaks min/max, $eÅ^{-3}$	-0.756/3.706	-1.524/2.107
CCDC number	2059184	2059187

Synthesis and characterization of new complexes

General considerations: All manipulations were carried out using standard Schlenk (under argon) and glovebox techniques (under hight purity nitrogen). All solvents were dried and degassed prior to use. Toluene (C_7H_8) and *n*-pentane (C_5H_{12}) were distilled under nitrogen over sodium. Tetrahydrofuran (thf) was distilled under nitrogen over sodium/benzophenone. Benzene-d_6 and toluene-d_8 were dried over 4 Å molecular sieves. Thf-d_8 was distilled over sodium under argon atmosphere and stored under 4 Å molecular sieves. Solution NMR spectra were recorded on Bruker DRX-400 and DRX-500 spectrometers. Spectra were referenced to external $SiMe_4$ (δ: 0 ppm) using the residual proton solvent peaks as internal standards (^1H NMR experiments), or the characteristic resonances of the solvent nuclei (^{13}C NMR experiments), while ^{31}P was referenced to H_3PO_4. Spectral assignments were made by routine one- and two-dimensional NMR experiments, where appropriate. For elemental analyses a LECO TruSpec CHN elementary analyser was utilised. Infrared spectra were recorded on a Bruker Vector 22 spectrometer and sampling preparation was made in Nujol. Complex **1·thf** and precursor $[Mo_2(\mu\text{-}O_2CH)_2(\mu\text{-}Ad^{Dipp2})_2]$ were prepared as reported in the literature[6,7].

[$Mo_2(H)(\mu\text{-}O_2CH)(\mu\text{-}Ad^{Dipp2})_2(thf)$] **(2·thf)**

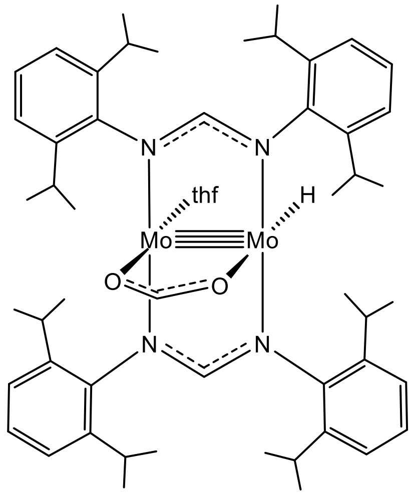

Complex [$Mo_2(\mu\text{-}O_2CH)_2(\mu\text{-}Ad^{Dipp2})_2$] (150 mg, 0.15 mmol) was dissolved in thf (30 mL) and 0.64 mL of LiEt (0.35 M in 10 cyclohexane: 90 benzene) was added at -20 °C. Ar atmosphere was evacuated and substituted by H_2 (1 bar). The mixture was allowed to reach room temperature and stirred for 4 hours. A yellow solid precipitated that was isolated by filtration, washed with 5 mL of thf and 5 mL of pentane and finally dried under vacuum for a few minutes (117 mg, 75 % yield).

^{1}H NMR (400 MHz, C_6D_6, 25 °C) δ (ppm): 1.03, 1.23, 1.30, 1.38 (d, 48 H, 12 H each, $^3J_{HH}$ = 6.9 Hz, CH*Me*$_2$), 1.31 (OCH$_2$C*H*$_2$), 3.47 (OC*H*$_2$CH$_2$), 3.47, 4.30 (sept, 8 H, 4 H each, $^3J_{HH}$ = 6.9 Hz, C*H*Me$_2$), 4.76 (br s, 1 H, Mo–H), 7.03-7.07 (m, 12 H, *m, p*-Dipp), 8.31 (s, 2 H, NC(H)N), 8.75 (d, 1 H, O_2CH, $^4J_{HH}$ = 2.2 Hz).

^{13}C{^{1}H} NMR (100 MHz, C_6D_6, 25 °C) δ (ppm): 24.5, 25.1, 26.2, 26.8, (CH*Me*$_2$), 25.7 (OCH$_2$*C*H$_2$), 28.3, 28.4 (*C*HMe$_2$), 68.7 (O*C*H$_2$CH$_2$), 124.0, 124.1, 126.2 (*m*-Dipp, *p*-Dipp), 145.1, 144.7, 144.7 (*ipso*-Dipp, *o*-Dipp), 163.3 (NC(H)N), 171.1 (O_2CH).

Elemental analysis (%): Calc. for $C_{55}H_{80}Mo_2N_4O_3$: C, 63.7; H, 7.8; N, 5.4; Expt.: C, 63.8; H, 7.8; N, 5.3.

IR (Nujol): 1711, 1670, 1627, 1609, 1551, 1519 cm^{-1}.

[$Mo_2(D)(\mu\text{-}O_2CH)(\mu\text{-}Ad^{Dipp2})_2(thf)$]: 20 mg of complex **2·thf** was dissolved in benzene under N_2 atmosphere, the solution was frozen and N_2 removed under vacuum. Then D_2 (1 bar) was added and the mixture was stirred for 1 hour at room temperature. The solvent was removed under vacuum and a yellow solid was isolated.

Monohydride Complexes with composition [Mo_2(H)(μ-O_2CH)(μ-Ad^{Dipp2})$_2$(L)] (L = dmap, PMe_3, IMe_4, the latter being short notation for 1,3,4,5-tetramethylimidazol-2-ylidene)

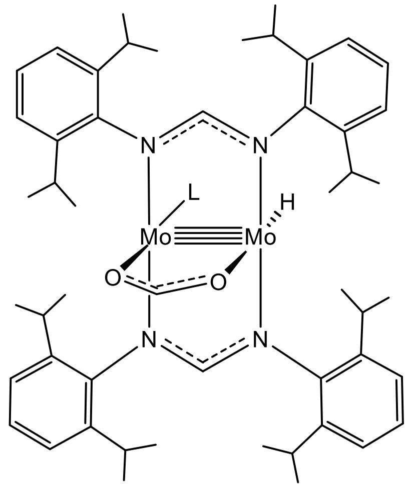

A general procedure for the synthesis of dmap, PMe_3 and IMe_4 adducts is given bellow for the IMe_4 derivative. Particular details for other adducts are provided separately.

[Mo_2(H)(μ-O_2CH)(μ-Ad^{Dipp2})$_2$(IMe_4)] **(2·IMe_4)**

A mixture of complex [Mo_2(H)(μ-O_2CH)(μ-Ad^{Dipp2})(thf)] (40 mg g, 0.038 mmol) and 1,3,4,5-tetramethylimidazol-2-ylidene (0.007 g, 0.057 mmol) was dissolved in toluene (8 mL) and stirred for 15 minutes, the solution changed in colour from yellow to orange. The solvent was removed under vacuum and pentane was added. After stirring for a few minutes the solvent was evaporated again under vacuum to afford the new complex as an orange solid in 95 % yield (38 mg). Crystals suitable for X-ray diffraction studies were obtained by slow evaporation of a benzene solution at room temperature.

^{1}H NMR (400 MHz, C_6D_6, 25 °C) δ (ppm): 0.27 (s, 3 H, N–Me), 0.51 (d, 6 H, $^3J_{HH}$ = 6.60 Hz, CH*Me*$_2$), 0.93 (s, 3 H, C–*Me*), 1.10, 1.25, 1.31, 1.33 (d, 6 H each, $^3J_{HH}$ = 6.60 Hz, CH*Me*$_2$), 1.40-1.47 (m, 18 H, CH*Me*$_2$), 1.57 (s, 3H, C–*Me*), 3.08, 3.69, 4.07 (sept, 2 H each, $^3J_{HH}$ = 6.60 Hz, C*H*Me$_2$), 4.74 (s, 3 H, N–Me), 4.96 (sept, 2 H, $^3J_{HH}$ = 6.60 Hz, C*H*Me$_2$), 6.80 (d, 2 H, *m*-Dipp), 6.95-7.10 (m, 10 H, *m, p*-Dipp), 7.81 (s, 1 H, Mo–H), 8.25 (s, 2 H, NC(H)N), 8.79 (s, 1 H, O_2CH).

$^{13}C\{^1H\}$ NMR (100 MHz, C_6D_6, 25 °C) δ (ppm): 7.6, 9.4 (C*Me*), 22.6, 23.6, 25.0, 25.6, 26.1, 26.5, 27.3 (CH*Me*$_2$), 27.7, 27.8, 27.9, 28.1 (*C*HMe$_2$), 30.0 (CH*Me*$_2$), 32.3, 37.1 (N*Me*), 123.1, 123.2, 123.8, 124.5, 125.2, 126.0 (*m*-Dipp, *p*-Dipp), 123.4, 126.4 (*C*Me), 144.8, 145.0, 145.2, 145.5, 145.6, 147.3 (*ipso*-Dipp, *o*-Dipp), 162.6 (NC(H)N), 167.5 (O_2CH), 195.0 (NCN). *C*Me and N*C*N signals from the IMe_4 ligand were detected by HMBC ^1H-^{13}C experiments.

Elemental analysis (%): Calc. for $C_{58}H_{84}Mo_2N_6O_2$·1/2(tol): C, 65.1; H, 7.8; N, 7.4; Expt.: C, 64.7; H, 7.8; N, 6.9.

IR (Nujol): 1764, 1665, 1613, 1599, 1549, 1514 cm^{-1}.

[Mo_2(H)(μ-O_2CH)(μ-Ad^{Dipp2})$_2$(PMe$_3$)] **(2·PMe$_3$)**

Using the above procedure, the title complex was obtained in 90 % yield (36 mg).

^1H NMR (400 MHz, C_6D_6, 25 °C) δ (ppm): 0.66 (m, 6 H, CH*Me*$_2$), 1.03 (m, 6 H, CH*Me*$_2$), 1.12 (m, 6 H, CH*Me*$_2$), 1.20-1.21 (m, 24 H, CH*Me*$_2$), 1.28 (d, 9 H, $^2J_{PH}$ = 7.2 Hz, PMe$_3$, becomes a broad singlet in $^1H\{^{31}P\}$), 1.33 (m, 6 H, CH*Me*$_2$), 3.09, 3.56, 3.73, 4.58 (m, 2 H each, C*H*Me$_2$), 7.09-7.02 (m, 12 H, *m, p*-Dipp), 8.38 (1 H, br d, Mo–H, $^3J_{PH}$ = 20 Hz, becomes a broad singlet in $^1H\{^{31}P\}$ spectrum), 8.44 (br s, 2 H, NC(H)N), 8.80 (br s, 1 H, O_2CH).

$^{31}P\{^1H\}$ NMR (160 MHz, C_6D_6, 25 °C) δ (ppm): -11.3.

$^{13}C\{^1H\}$ NMR (100 MHz, C_6D_6, 25 °C) δ (ppm): 19.5 (d, $^2J_{CP}$ = 24.7 Hz, PMe$_3$), 24.2, 24.5, 25.1, 25.7, 25.8, 25.9, 26.6, 27.6 (CH*Me*$_2$), 28.1, 28.3, 29.0, 30.2 (*C*HMe$_2$), 124.1, 124.3, 124.6, 124.9, 125.4, 126.2 (*m*-Dipp, *p*-Dipp), 142.1, 144.0, 144.2, 145.2, 145.3, 146.5 (*ipso*-Dipp, *o*-Dipp), 165.6 (NC(H)N), 168.7 (O_2CH).

Elemental analysis (%): Calc. for $C_{54}H_{81}Mo_2N_4O_2P$·1/2(tol): C, 63.5; H, 7.9; N, 5.1; Expt.: C, 63.2; H, 8.3; N, 4.5.

[Mo_2(H)(μ-O_2CH)(μ-Ad^{Dipp2})$_2$(dmap)] **(2·dmap)**

Using the above procedure, the title complex was obtained in 62 % yield (26 mg). In this case, the product was insoluble in toluene and precipitated as an orange solid that was further washed with pentane.

^{1}H NMR (400 MHz, thf-*d*8 25 °C) δ (ppm): 0.50, 1.06, 1.08, 1.11, 1.15, 1.20, 1.31, 1.33 (d, 48 H, 6 H each, $^3J_{HH}$ = 6.8 Hz, CH*Me*$_2$), 2.93 (6 H, s, py-NMe$_2$) 3.07, 3.45, 3.71, 4.81 (sept, 8 H, 2 H each, $^3J_{HH}$ = 6.8 Hz, C*H*Me$_2$), 6.50 (2 H, CH, py-NMe$_2$, $^2J_{HH}$ = 6.2 Hz), 6.86-7.07 (m, 12 H, *m, p*-Dipp), 7.71 (br s, 1 H, Mo–H), 8.08 (s, 2 H, NC(H)N), 8.10 (d, 2 H, CH, py-NMe$_2$, $^2J_{HH}$ = 6.2 Hz), 8.88 (d, 1 H, O_2CH, $^4J_{HH}$ = 2.2 Hz).
^{13}C{^{1}H} NMR (100 MHz, thf-*d*8, 25 °C) δ (ppm): 24.1, 25.4, 25.6, 25.7, 25.9, 26.8, 27.1, 27.8 (CH*Me*$_2$), 28.1, 28.2, 28.3, 29.4 (*C*HMe$_2$), 38.7 (py-N*Me*$_2$), 106.9 (*C*H, py-NMe$_2$), 123.0, 123.4, 124.0, 124.3, 125.2, 125.5 (*m*-Dipp, *p*-Dipp), 144.7, 145.2, 145.3, 145.5, 145.8, 146.8 (*ipso*-Dipp, *o*-Dipp), 150.3 (*C*H, py-NMe$_2$), 155.6 (3-py), 163.5 (NC(H)N), 168.4 (O_2CH).
Elemental analysis (%): Calc. for $C_{58}H_{82}Mo_2N_6O_2$: C, 64.1; H, 7.6; N, 7.7; Expt.: C, 64.4; H, 8.3; N, 7.8.

[Mo_2(μ-O_2CMe)$_3$(μ-Ad^{Dipp2})]

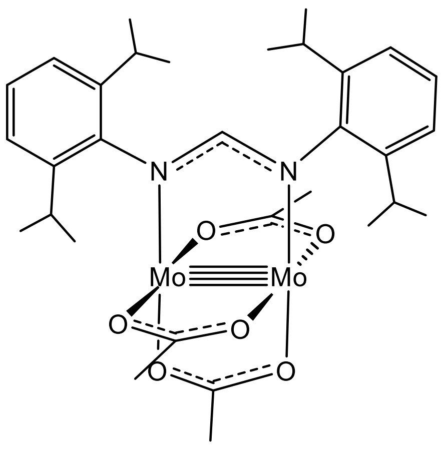

[Mo_2(μ-O_2CMe)$_4$] (100 mg, 0.23 mmol) and Li(thf)$_2$(μ-Ad^{Dipp2}) (118 mg, 0.23 mmol) were placed in an ampoule and dissolved in 20 mL of thf at -40 °C. The mixture was stirred at low temperature for 3 hours and then warmed up to room temperature. Removal of the solvent under vacuum led to a yellow solid that was washed with thf (1 x 5 mL) and pentane (1 x 5 mL) and dried for a few minutes. The complex was obtained in 86 % yield (145 mg).

^{1}H NMR (400 MHz, C_6D_6, 25 °C) δ (ppm): 1.28, 1.41 (d, 24 H, $^3J_{HH}$ = 6.8 Hz, CH*Me$_2$*), 2.52 (s, 3 H, O_2C*Me*), 2.60 (s, 6 H, O_2C*Me*), 3.48 (sept, 4 H, $^3J_{HH}$ = 6.8 Hz, C*H*Me$_2$), 7.14-7.23 (m, 6 H, *m*-Dipp, *p*-Dipp), 7.99 (s, 1 H, NC(H)N).
^{13}C{^{1}H} NMR (100 MHz, C_6D_6, 25 °C) δ (ppm): 23.3, 23.9 (O_2C*Me*), 24.8, 26.8 (CH*Me$_2$*), 27.9 (*C*HMe$_2$), 123.9 (*m*-Dipp), 126.6 (*p*-Dipp), 143.5 (*ipso*-Dipp), 145.6 (*o*-Dipp), 160.6 (NC(H)N), 180.6, 181.5 (O_2*C*Me).
Elemental Analysis (%) for $C_{31}H_{44}Mo_2N_2O_6$: Calc. C, 50.8; H, 6.1; N, 3.8; Exp. C, 50.9; H, 6.3; N, 3.4.

$[Mo_2\{(H)_2Li(thf)\}(\mu\text{-}O_2CH)(\mu\text{-}Ad^{Dipp2})_2]$ **(3·thf)**

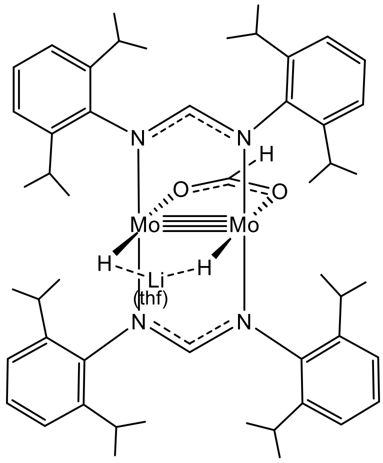

<u>Method A</u>: $LiAlH_4$ (0.50 mmol, 0.50 mL of a 1 M solution in thf) was added to a suspension of $[Mo_2(\mu\text{-}O_2CH)_2(\mu\text{-}Ad^{Dipp2})_2]$ (150 mg, 0.15 mmol) in 15 mL of thf at 0°C. The starting yellow suspension went orange and the solid solubilised in 30 minutes. After further stirring of 15 minutes at room temperature a bright yellow solid precipitated that was filtered, washed with thf (2x10 mL) and dried under vacuum for few minutes to obtain the final product (102 mg) in 65% isolated yield.

<u>Method B</u>: $LiAlH_4$ (0.10 mmol, 0.10 mL of a 1 M solution in thf) was added slowly to a solution of $[Mo_2(H)(\mu\text{-}O_2CH)(\mu\text{-}Ad^{Dipp2})_2(thf)]$, **2·thf**, (104 mg, 0.10 mmol) in thf (10 mL) at -20 °C. The solution was stirred for one hour while reaching ambient temperature. A yellow solid precipitated that was isolated by filtration and dried under vacuum to obtain a yellow solid (73 mg, 70 %). By NMR spectroscopy the crude product obtained consisted of a *ca.* 80:20 mixture of complexes $[Mo_2\{(H)_2Li(thf)\}(\mu\text{-}O_2CH)(\mu\text{-}Ad^{Dipp2})_2]$ and $[Mo_2\{(H)_2Li(thf)\}_2(\mu\text{-}Ad^{Dipp2})_2]$.

Monocrystals suitable for X-ray diffraction studies were obtained from a saturated solution of the complex in toluene kept in the freezer at -35 °C for three days.

^1H NMR (400 MHz, C_6D_6, 25 °C) δ (ppm): 1.19, 1.27, 1.30, 1.34 (d, 48 H, 12 H each, $^3J_{HH}$ = 6.8 Hz, CH*Me*$_2$), 1.42 (m, OCH_2CH_2), 3.59 (m, OCH_2CH_2), 3.78, 4.11 (sept, 8 H, 4 H each, $^3J_{HH}$ = 6.8 Hz, C*H*Me$_2$), 4.33 (br m, 2 H, Mo–H, becomes a singlet in the $^1H\{^7Li\}$ spectrum), 7.03-7.18 (m, 12 H, *m, p*-Dipp), 8.12 (s, 2 H, NC(H)N), 8.69 (s, 1 H, O_2CH).

$^{13}C\{^1H\}$ NMR (100 MHz, C_6D_6, 25 °C) δ (ppm): 25.0, 25.5, 26.4, 26.6 (CH*Me*$_2$), 27.9, 28.4 (*C*HMe$_2$), 25.8 (OCH$_2$*C*H$_2$), 68.0 (O*C*H$_2$CH$_2$), 123.6, 124.3 (*m*-Dipp), 126.1 (*p*-Dipp), 144.5, 145.2 (*o*-Dipp), 146.1 (*ipso*-Dipp), 161.8 (NC(H)N), 166.9 (O_2CH).

^7Li NMR (161 MHz, C_6D_6, 25 °C) δ (ppm): 3.6 (t) $^1J_{LiH}$ = 16 Hz.

$^7Li\{^1H\}$ NMR (161 MHz, C_6D_6, 25 °C) δ (ppm): 3.6 (s).

Elemental Analysis (%): Calc. for $C_{63}H_{97}Mo_2N_4O_5Li{\cdot}3thf$: C, 63.6; H, 8.2; N, 4.7; Expt.: C, 63.8; H, 8.4; N, 4.5.

IR (Nujol): 1671, 1627, 1612, 1596, 1531, 1514 cm^{-1}.

[$Mo_2\{(D)_2Li(thf)\}(\mu\text{-}O_2CH)(\mu\text{-}Ad^{Dipp2})_2$]: method B was employed using $LiAlD_4$. The **3·thf** isotopologue complex was detected by the absent in the ^1H NMR spectrum of the DLiD signal and the presence of a broad multiplet in the ^2H NMR spectrum at 4.35 ppm.

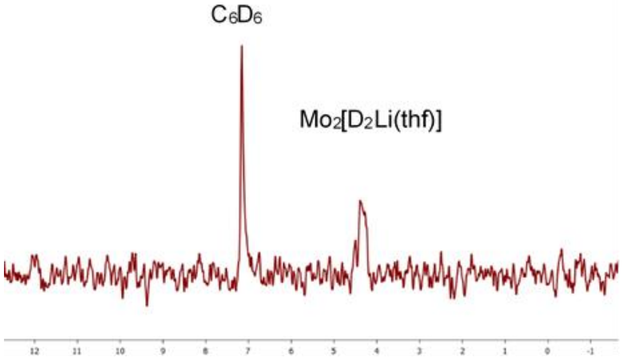

^2H NMR (400 MHz, C_6H_6 ,25 °C)

$[Mo_2\{(H)_2Li(thf)\}_2(\mu\text{-}Ad^{Dipp2})_2]$ **(4·thf)**

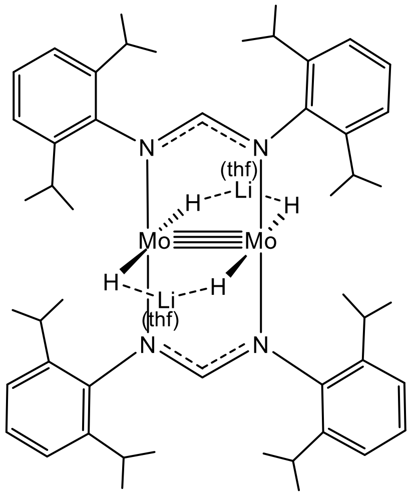

Method A: A suspension of complex $[Mo_2(H)_2(\mu\text{-}Ad^{Dipp2})_2(thf)_2]$ (100 mg, 0.10 mmol) in thf (10 mL) was stirred at 25 °C and $LiAlH_4$ (0.30 mmol, 0.30 mL of a 1 M solution in thf) was added. After 30 minutes of stirring a bright yellow solid precipitated, which was filtered and dried under vacuum for a few minutes to obtain the final compound in 71% isolated yield (77 mg). Crystals were obtained by adding slowly to the suspension of the starting material in thf a solution of $LiAlH_4$ in thf and leaving the system unstirred for 2 days at room temperature.

Method B: $LiAlH_4$ (2.40 mmol, 2.40 mL of a 1 M solution in thf) was added to a suspension of $[Mo_2(\mu\text{-}O_2CR)_2(\mu\text{-}Ad^{Dipp2})_2]$, R = H, CH_3 (400 mg, 0.40 mmol) in a mixture of 20 mL of thf and 20 mL of toluene at room temperature. After 12 hours of stirring at 70 °C, the mixture was concentrated to a volume of *ca*. 20 mL and then centrifuged. The very insoluble yellow solid was separated from the orange solution and washed twice with thf and pentane, and finally dried under vacuum for a few minutes, to obtain the desired product (360 mg, 83 %). The product is highly insoluble in common solvents such as benzene, toluene or thf and decomposes in dichloromethane. Higher solubility was observed in fluorobenzene.

^1H NMR (500 MHz, C_6D_5F, 25 °C) δ (ppm): 1.55, 1.61 (d, 24 H each, $^3J_{HH}$ = 6.8 Hz, $CHMe_2$), 1.97 (m, 4 H, OCH_2CH_2), 4.10 (m, 4 H, OCH_2CH_2), 4.40 (sept, 8 H, $^3J_{HH}$ = 6.8 Hz, $CHMe_2$)

4.70 (br m, 4 H, Mo–H, becomes a singlet in the $^{1}H\{^{7}Li\}$ spectrum), 7.16-7.43 (m, 12 H, *m*, *p*-Dipp), 8.17 (s, 2 H, NC(H)N).

^{7}Li NMR (161 MHz, C_6D_5F, 25 °C) δ (ppm): 4.5 (t) $^{1}J_{LiH}$ = 17 Hz.

$^{7}Li\{^{1}H\}$ NMR (161 MHz, C_6D_5F, 25 °C) δ (ppm): 4.5 (s).

$^{13}C\{^{1}H\}$ NMR (100 MHz, C_6D_5F, 25 °C) δ (ppm): 25.1, 26.6, (CH*Me*$_2$), 25.5 (OCH$_2$*C*H$_2$), 28.2 (*C*HMe$_2$), 68.6 (br, O*C*H$_2$CH$_2$), 125.2 (*m*-Dipp or *p*-Dipp), 145.0, 146.6 (*o*-Dipp, *ipso*-Dipp), 158.4 (NC(H)N). One *m*-Dipp or *p*-Dipp signal was not observed due to overlap with C_6D_5F signals.

Elemental Analysis (%): Calc. for $C_{50}H_{74}Mo_2N_4Li_2$·2thf: C, 64.4; H, 8.4; N, 5.2; Expt.: C, 64.4; H, 8.5; N, 5.4.

IR (Nujol): 1590, 1514 cm^{-1}.

[$Mo_2\{(D)_2Li(thf)\}_2(\mu\text{-}Ad^{Dipp2})_2$]: method B was employed using $LiAlD_4$.

$[Mo_2(H_2Li)_3(\mu\text{-}Ad^{Dipp2})]_3\cdot(thf)_8$ **(5·thf)**

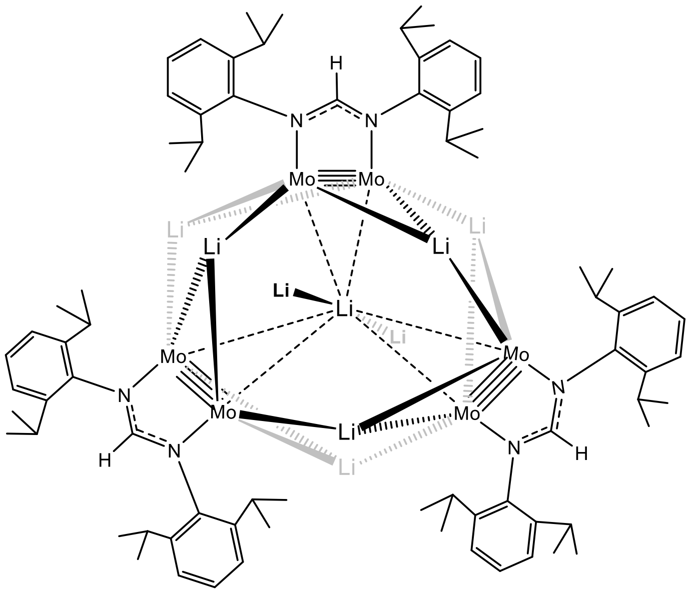

Hydrides and thf molecules have been removed for clarity in this representation.

100 mg (0.14 mmol) of $[Mo_2(\mu\text{-}O_2CMe)_3(\mu\text{-}Ad^{Dipp2})]$ were dissolved in thf (10 mL) at -60 °C. $LiAlH_4$ (0.40 mL of a 1 M solution in thf) was added slowly. After 30 minutes of stirring at low temperature the solution turned dark red. The mixture was further stirred for 30 minutes and warmed up to -20 °C. Then, the red solution was filtered, concentrated to half its initial volume and kept overnight at -30 °C, to achieve a red microcrystalline solid (27 mg, 25% yield). The synthesis generated by-products such as ($[Mo_2(\mu\text{-}O_2CMe)_2(\mu\text{-}Ad^{Dipp2})_2]$). Attempts to perform the reaction in a larger scale led to decomposition and the formation of $LiAd^{Dipp2}$. By slow evaporation of the solvent (thf) under nitrogen at room temperature, red crystals suitable for X-ray diffraction were obtained.

^{1}H NMR (500 MHz, thf-*d*8, 25 °C) δ (ppm): 0.90, 1.14, 1.27, 1.53 (d, 18 H each, $^3J_{HH}$ = 6.8 Hz, CH*Me*$_2$), 1.72, 3.57 (thf), 4.10, 4.59 (sept, 6 H each, $^3J_{HH}$ = 6.8 Hz, C*H*Me$_2$), 4.43, 4.50 (br m, 12 H, 6 H each, Li–H, become 2 doublets, $^2J_{HH}$ = 4 Hz, in ^{1}H{^{7}Li} spectrum), 6.83-6.90 (m, 12 H, *m*, *p*-Dipp), 7.03 (d, 6 H, $^3J_{HH}$ = 7.6 Hz, *m*, *p*-Dipp), 7.30 (s, 3 H, NC(H)N).
^{7}Li{^{1}H} NMR (194 MHz, thf-*d*8, 25 °C) δ (ppm): 3.8, 1.6 (7:1).
^{1}H NMR (500 MHz, C_6D_6, 25 °C) δ (ppm): 0.96, 1.47, 1.56, 1.76 (d, 18 H each, $^3J_{HH}$ = 6.8 Hz, CH*Me*$_2$), 1.28, 3.31 (thf), 4.38, 4.73 (sept, 6 H each, $^3J_{HH}$ = 6.8 Hz, C*H*Me$_2$), 4.48, 4.63

(br m, 12 H, 6 H each, Li–H, become 2 doublets, $^{2}J_{HH}$ = 4 Hz, in $^{1}H\{^{7}Li\}$ spectrum), 7.08-7.26 (m, 18 H, *m*, *p*-Dipp), 7.68 (s, 3 H, NC(H)N).

$^{7}Li\{^{1}H\}$ NMR (194 MHz, C_6D_6, 25 °C) δ, ppm: 4.6, 4.2, 1.8 (6:1:2).

$^{13}C\{^{1}H\}$ NMR (100 MHz, thf-*d*8, 25 °C) δ (ppm): 25.8, 26.6, 27.4, 28.1 (CH*Me*$_2$, *C*HMe$_2$), 122.7, 123.7, 124.7 (*m*-Dipp, *p*-Dipp), 144.5, 146.5, 147.4 (*o*-Dipp, *ipso*-Dipp), 154.7 (N*C*(H)N). Two CH*Me*$_2$ signals were overlapped with the thf.

Elemental Analysis for $C_{107}H_{187}Li_9Mo_6N_6O_8$: Calc. C, 55.3; H, 8.1; N, 3.8. Expt. C, 55.5; H, 8.1; N, 3.4.

IR (Nujol): 1596 (br), 1512 cm^{-1}.

$[Mo_2(H_2Li)_3(\mu\text{-}Ad^{Dipp2})]_3\cdot(py)_8$ **(5·py)**

20 mg (0.009 mmol) of $[Mo_2(H_2Li)_3(\mu\text{-}Ad^{Dipp2})]_3\cdot(thf)_8$ were dissolved in toluene and an excess of pyridine (0.09 mmol) was added at room temperature. The mixture was stirred for 10 minutes and the solvent was removed under vacuum. A red solid appeared that was dried under vacuum and isolated in 93 % yield (21 mg).

^{1}H NMR (400 MHz, C_6D_6, 25 °C) δ (ppm): 0.81, 1.38, 1.56, 1.59 (d, 18 H each, $^{3}J_{HH}$ = 6.8 Hz, CH*Me*$_2$), 2.01 (br m, 6 H, Li–H, becomes a broad singlet in $^{1}H\{^{7}Li\}$ spectrum), 4.57, 4.98 (sept, 6 H each, $^{3}J_{HH}$ = 6.8 Hz, C*H*Me$_2$), 5.18, 5.33 (br m, 12 H, 6 H each, Li–H, become 2 doublets in $^{1}H\{^{7}Li\}$ spectrum, $^{2}J_{HH}$ = 4 Hz), 6.47 (br, 16 H, *m*-py), 6.82 (br, 8 H, *p*-py), 7.19-7.30 (18 H, *m*, *p*-Dipp), 7.77 (s, 3 H, NC(H)N), 8.12 (br, 16 H, *o*-py).

^{1}H NMR and $^{1}H\{^{7}Li\}$ (500 MHz, C_6D_6, 25 °C) δ (ppm): 2.04 (br m, 6 H, Li–H, becomes a broad singlet in $^{1}H\{^{7}Li\}$ spectrum), 5.21, 5.41 (br m, 12 H, 6 H each, Li–H, become 2 doublets in $^{1}H\{^{7}Li\}$ spectrum, $^{2}J_{HH}$ = 4 Hz).

$^{7}Li\{^{1}H\}$ NMR (161 MHz, C_6D_6, 25 °C) δ (ppm): 5.4, 4.7, 2.7 (6:1:2).

$^{13}C\{^{1}H\}$ NMR (100 MHz, C_6D_6, 25 °C) δ (ppm): 24.6, 26.0, 26.1, 27.5, 28.0, 28.2 (CH*Me*$_2$, *C*HMe$_2$),123.4 (br, *m*-py), 122.9, 124.3, 125.1 (*m*-Dipp, *p*-Dipp), 136.2 (br, *p*-py), 144.5, 146.9, 147.3 (*o*-Dipp, *ipso*-Dipp), 150.8 (br, *o*-py), 155.4 (NC(H)N).

Elemental Analysis for $C_{115}H_{163}Li_9Mo_6N_{14}$: Calc. C, 58.0; H, 6.9; N, 8.2. Expt. C, 58.0; H, 7.4; N, 7.8.

$[Mo_2(H_2Li)_3(\mu\text{-}Ad^{Dipp2})]_3\cdot(dmap)_8$ **(5·dmap)**

20 mg (0.009 mmol) of $[Mo_2(H_2Li)_3(\mu\text{-}Ad^{Dipp2})]_3\cdot(thf)_8$ and dimethylaminopyridine (8.8 mg, 0.072 mmol) were dissolved at room temperature. The mixture was stirred for 10 minutes and the solvent was removed under vacuum. The resulting red solid was dried under vacuum and isolated in 95 % yield (23 mg).

^{1}H NMR (400 MHz, C_6D_6, 25 °C) δ (ppm): 1.05 (d, 18 H, $^3J_{HH}$ = 6.7 Hz, CH*Me*$_2$), 1.61 (d, 18 H, $^3J_{HH}$ = 6.7 Hz, CH*Me*$_2$), 1.69 (m, 36 H, CH*Me*$_2$), 2.13 (s, 48 H, NMe$_2$, dmap), 4.73, 5.15 (sept, 6 H each, $^3J_{HH}$ = 6.7 Hz, C*H*Me$_2$), 5.29, 5.36 (br m, 12 H, 6 H each, Li–H, become 2 doublets, $^2J_{HH}$ = 4 Hz, in ^{1}H{^{7}Li} spectrum), 5.88 (br, 16 H, dmap), 7.23 (m, 12 H, *m*, *p*-Dipp), 7.35 (m, 6 H, *m*, *p*-Dipp), 7.81 (s, 3 H, NC(H)N), 8.01 (br, 16 H, dmap).

^{1}H NMR (400 MHz, thf-*d*8, 25 °C) δ (ppm): 0.84, 0.93, 1.20, 1.56 (d, 18 H each, $^3J_{HH}$ = 6.8 Hz, CH*Me*$_2$), 2.98 (s, 48 H, NMe$_2$, dmap), 4.16, 4.65 (sept, 6 H each, $^3J_{HH}$ = 6.8 Hz, C*H*Me$_2$), 4.51-4.58 (br m, 12 H, Li–H), 6.49 (d, 16 H, dmap, $^3J_{HH}$ = 5.8 Hz), 6.89 (d, 6 H, *m*-Dipp, $^3J_{HH}$ = 7.9 Hz), 6.95 (t, 6 H, *p*-Dipp, $^3J_{HH}$ = 7.9 Hz), 7.06 (d, 6 H, *m*-Dipp, $^3J_{HH}$ = 7.9 Hz), 7.35 (s, 3 H, NC(H)N), 8.10 (d, 16 H, dmap, $^3J_{HH}$ = 5.8 Hz).

^{7}Li{^{1}H} NMR (161 MHz, C_6D_6, 25 °C) δ (ppm): 5.4, 4.7, 2.7 (6:1:2).

^{13}C{^{1}H} NMR (100 MHz, thf-*d*8, 25 °C) δ (ppm): 22.6, 24.9, 25.7, 26.5, 27.2, 29.7(CH*Me*$_2$, *C*HMe$_2$), 38.0 (NMe$_2$, dmap), 106.2 (CH, dmap), 121.8, 122.8, 123.8 (*m*-Dipp, *p*-Dipp), 143.6, 145.6, 146.6 (*o*-Dipp, *ipso*-Dipp), 149.7 (CH, dmap), 153.7 (NC(H)N), 154.1 (*C*NMe$_2$, dmap). Some CH*Me*$_2$ signals were overlapped with the thf and detected by ^{1}H-^{13}C HSQC experiments.

Elemental Analysis for $C_{131}H_{203}Li_9Mo_6N_{22}$: Calc. C, 57.8; H, 7.5; N, 11.3. Expt. C, 57.7; H, 7.7; N, 11.0.

NMR spectra of new complexes

[$Mo_2(H)(\mu\text{-}O_2CH)(\mu\text{-}Ad^{Dipp2})_2(thf)$] **(2·thf)**

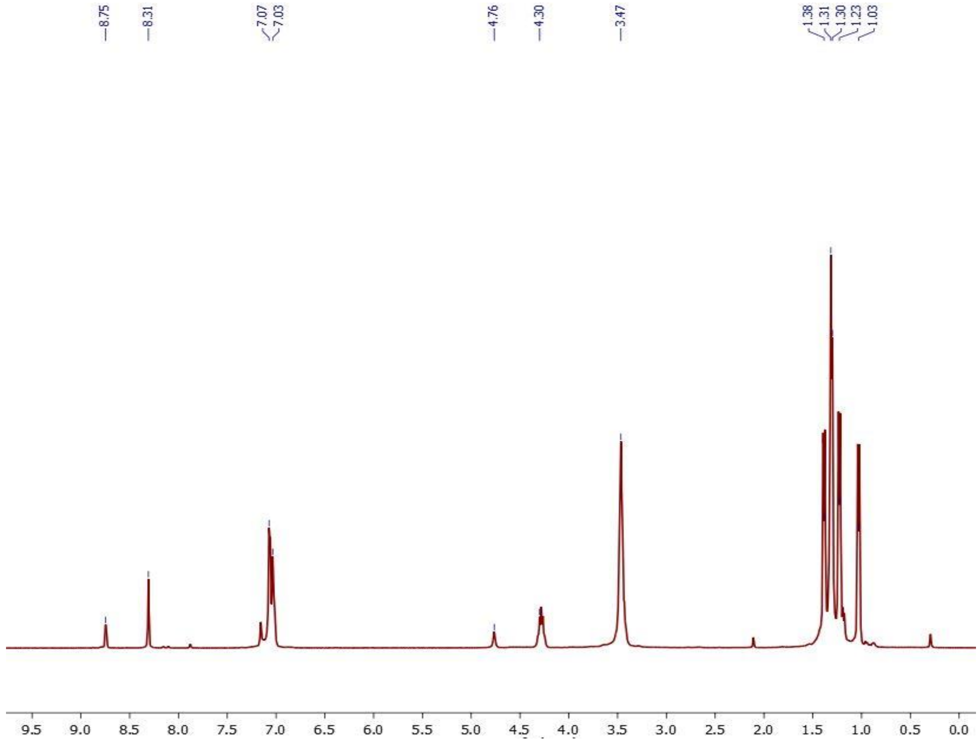

^1H NMR (400 MHz, C_6D_6, 25 °C)

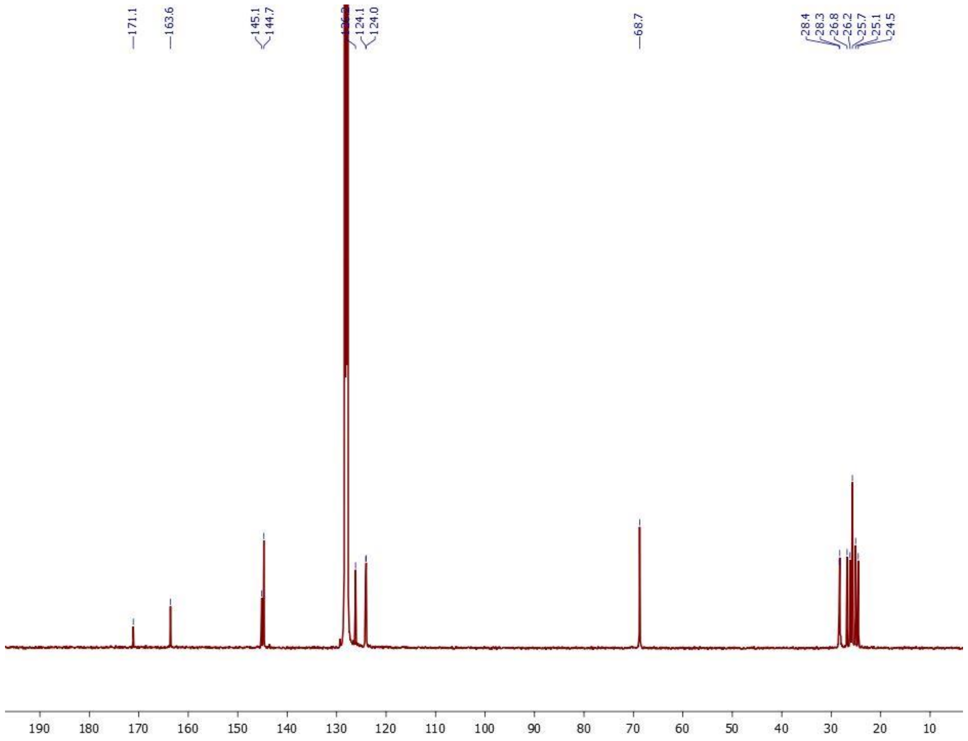

$^{13}C\{^1H\}$ NMR (100 MHz, C_6D_6, 25 °C)

[$Mo_2(H)(\mu\text{-}O_2CH)(\mu\text{-}Ad^{Dipp2})_2(IMe_4)$] **(2·IMe₄)**

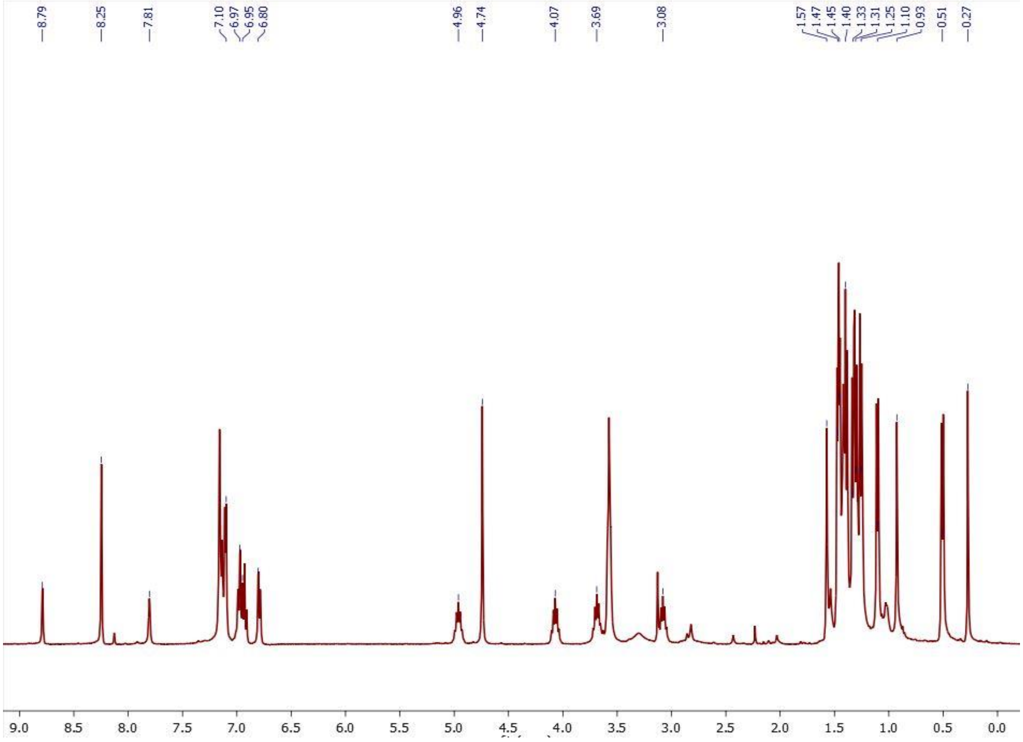

^1H NMR (400 MHz, C_6D_6, 25 °C)

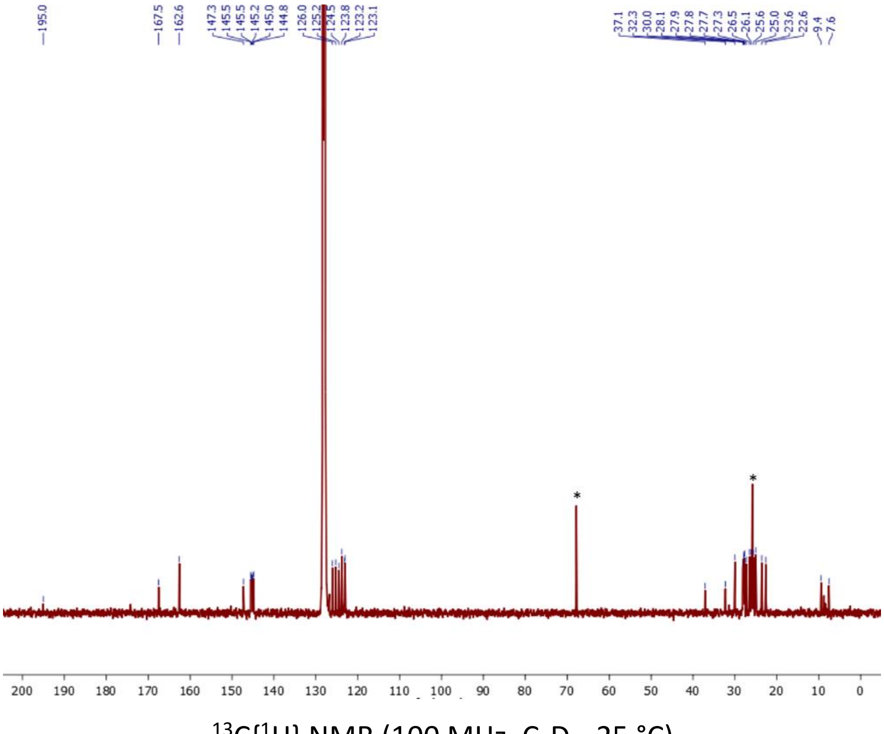

$^{13}C\{^1H\}$ NMR (100 MHz, C_6D_6, 25 °C)

[$Mo_2(H)(\mu\text{-}O_2CH)(\mu\text{-}Ad^{Dipp2})_2(PMe_3)$] **(2·PMe$_3$)**

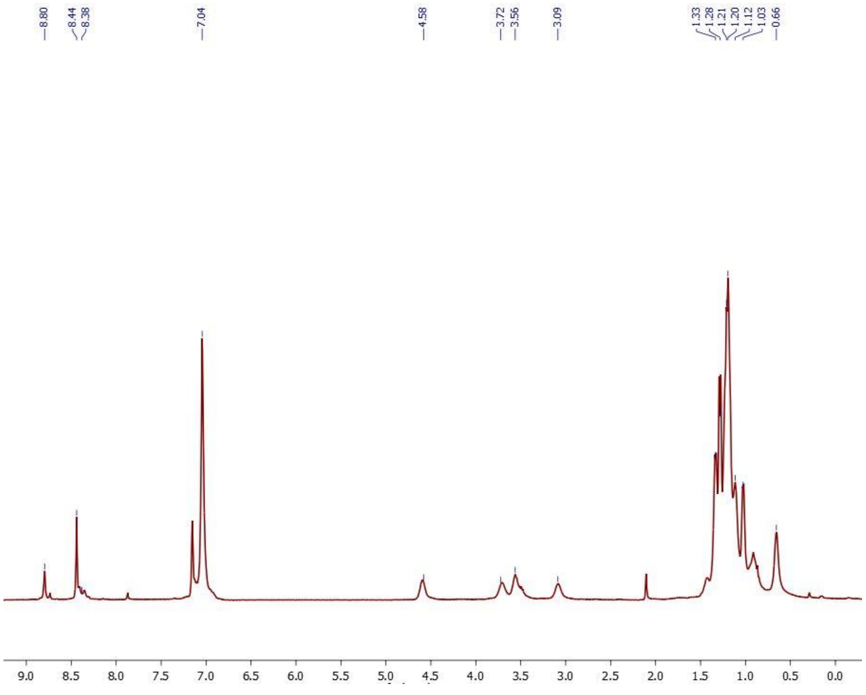

^1H NMR (400 MHz, C_6D_6, 25 °C)

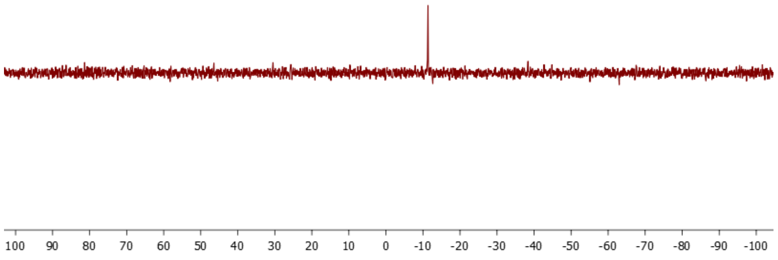

$^{31}P\{^1H\}$ NMR (160 MHz, C_6D_6, 25 °C)

[Mo_2(H)(μ-O_2CH)(μ-Ad^{Dipp2})$_2$(PMe_3)] **(2·PMe$_3$)**

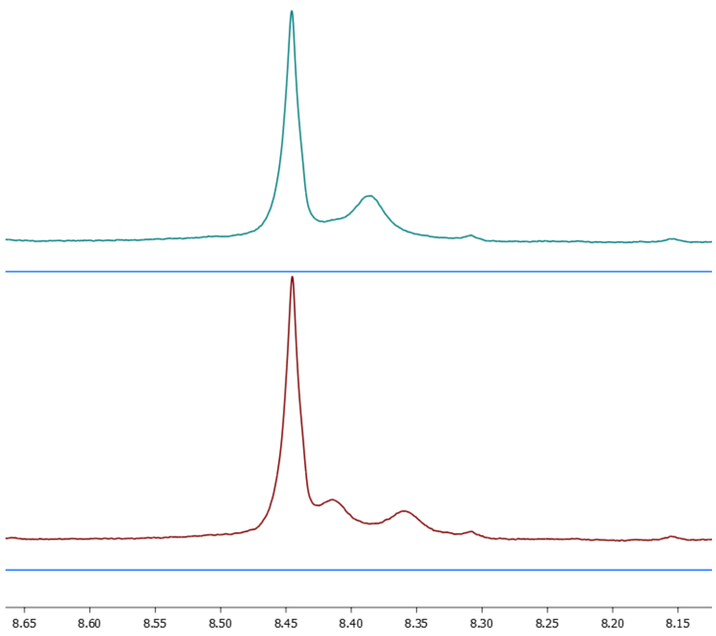

^{1}H{^{31}P} (top) and ^{1}H (bottom) NMR (400 MHz, C_6D_6, 25 °C)

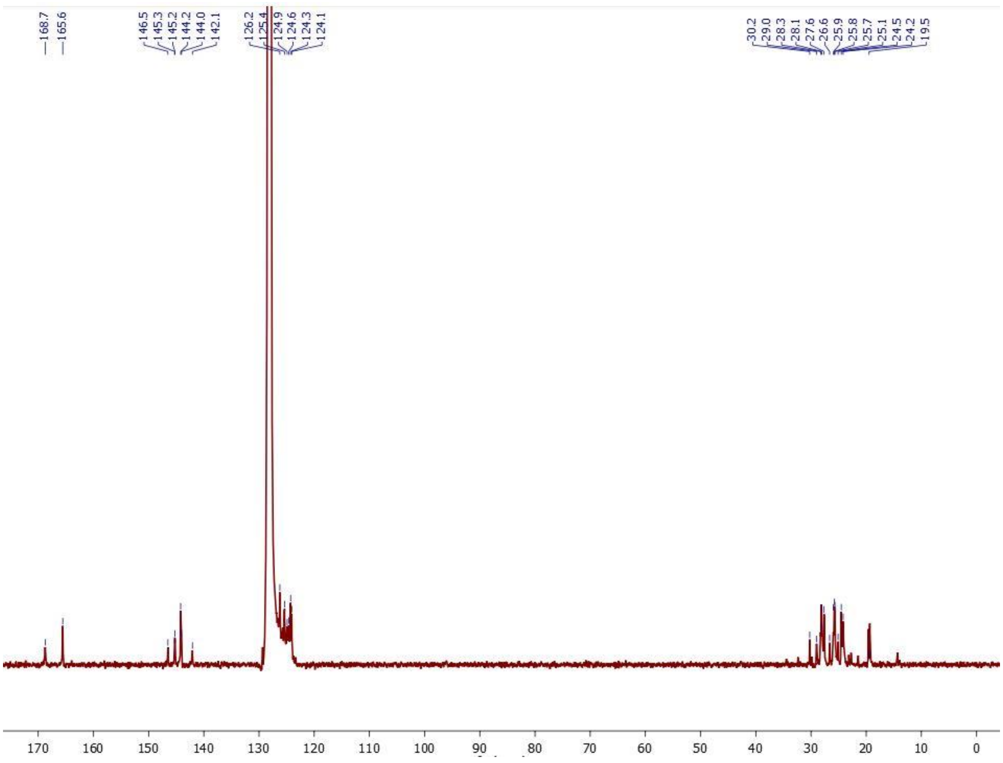

^{13}C{^{1}H} NMR (100 MHz, C_6D_6, 25 °C)

[Mo_2(H)(μ-O_2CH)(μ-Ad^{Dipp2})$_2$(dmap)] **(2·dmap)**

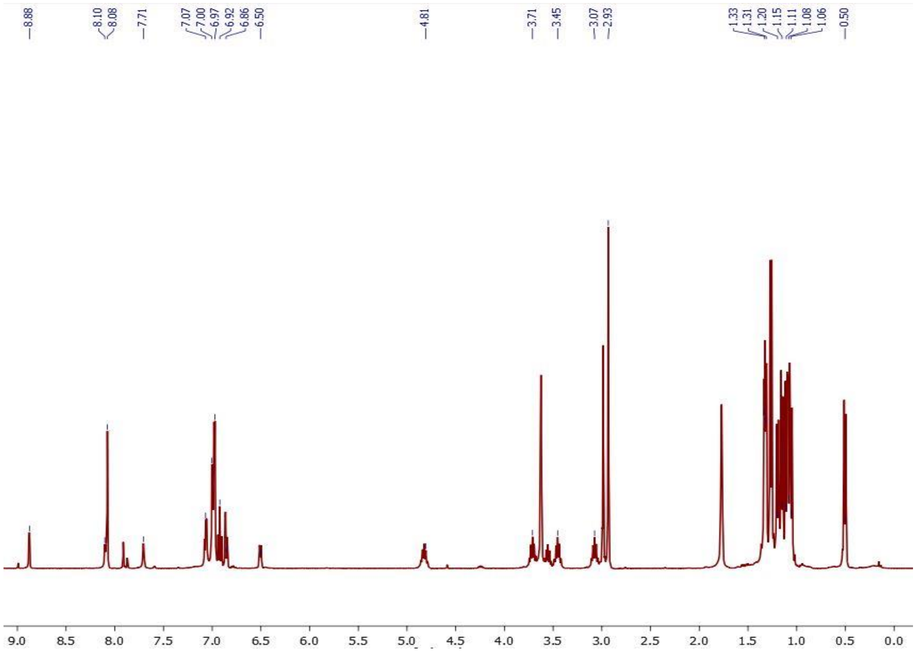

^{1}H NMR (400 MHz, thf-*d*8, 25 °C)

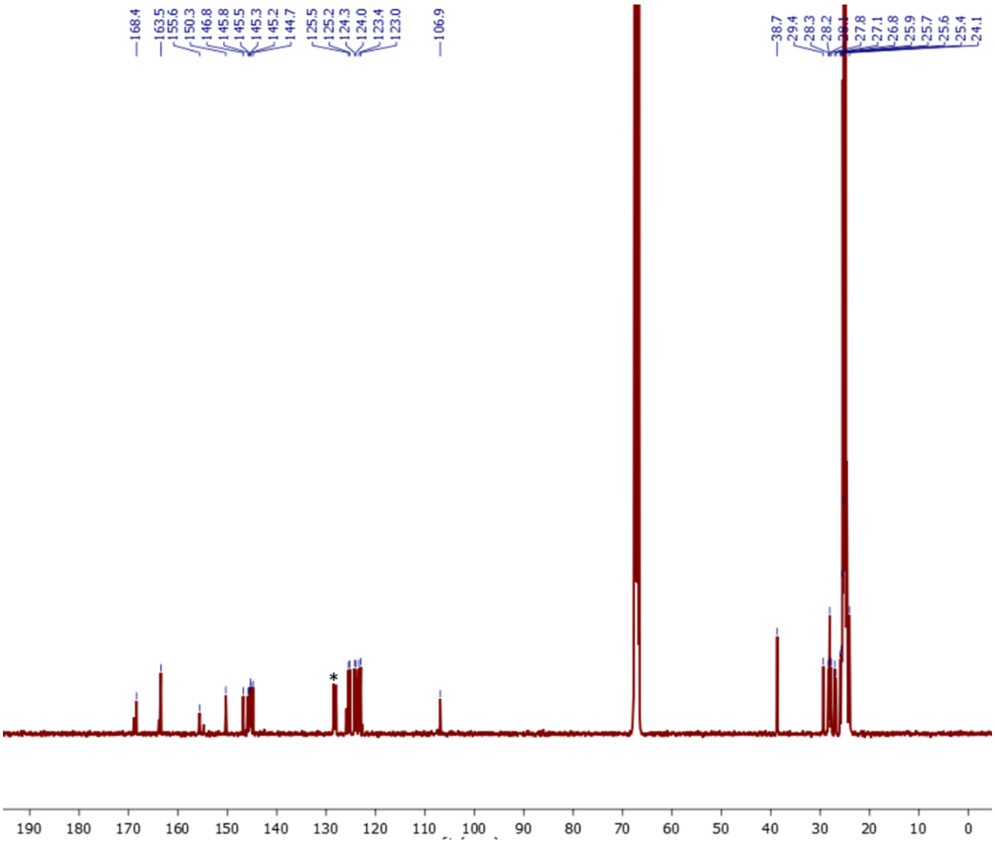

^{13}C{^{1}H} NMR (100 MHz, thf-*d*8, 25 °C) *C_6D_6

[$Mo_2(\mu\text{-}O_2CMe)_3(\mu\text{-}Ad^{Dipp2})$]

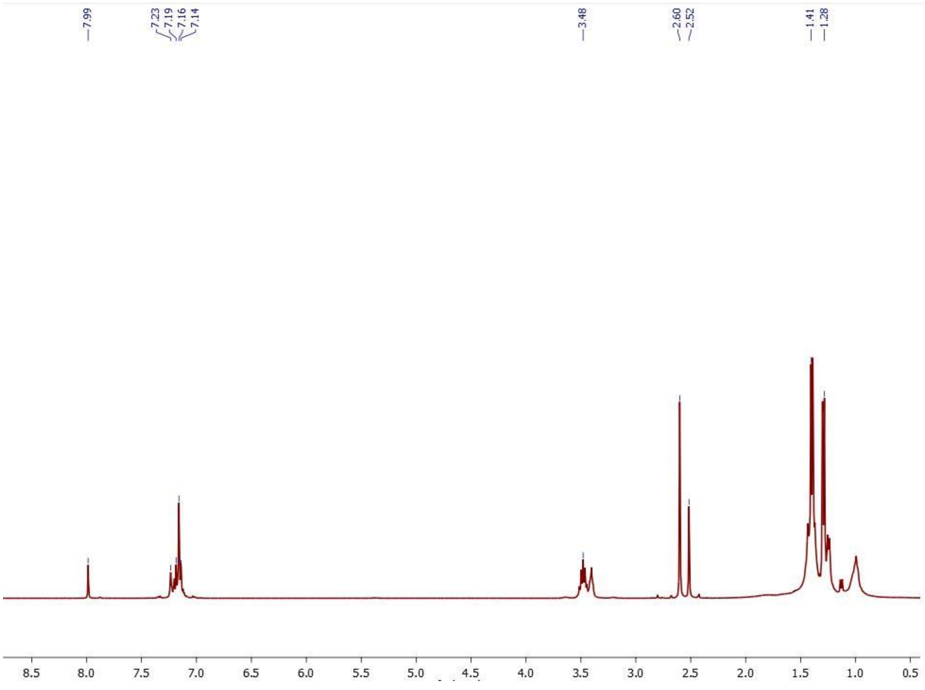

^1H NMR (400 MHz, C_6D_6, 25 °C)

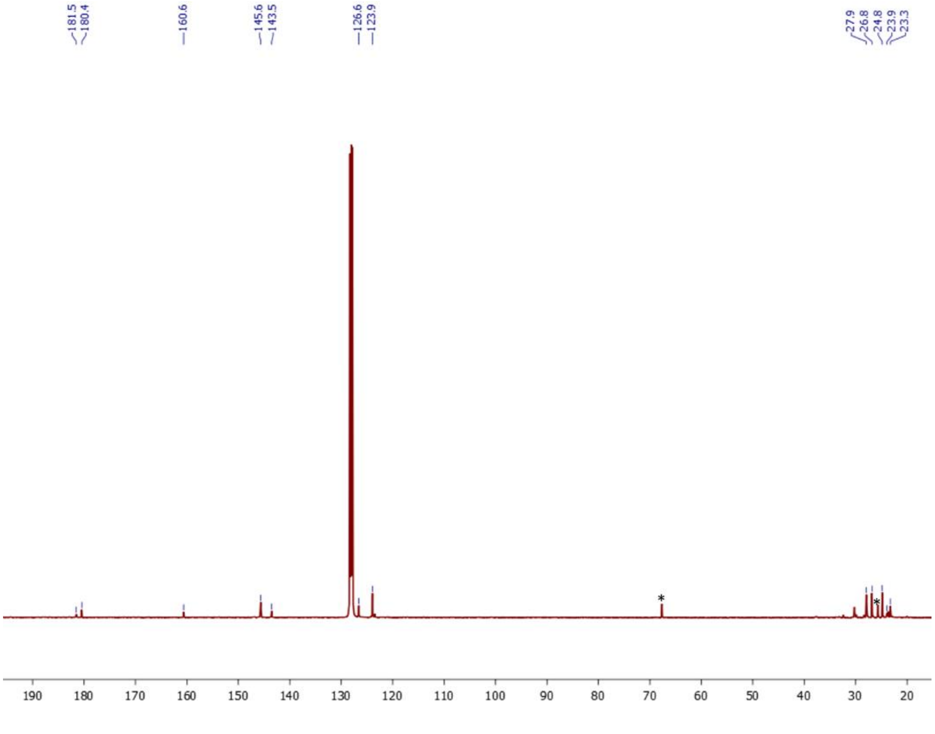

$^{13}C\{^1H\}$ NMR (100 MHz, C_6D_6, 25 °C) *thf

$[Mo_2\{(H)_2Li(thf)\}(\mu\text{-}O_2CH)(\mu\text{-}Ad^{Dipp2})_2]$ (**3·thf**)

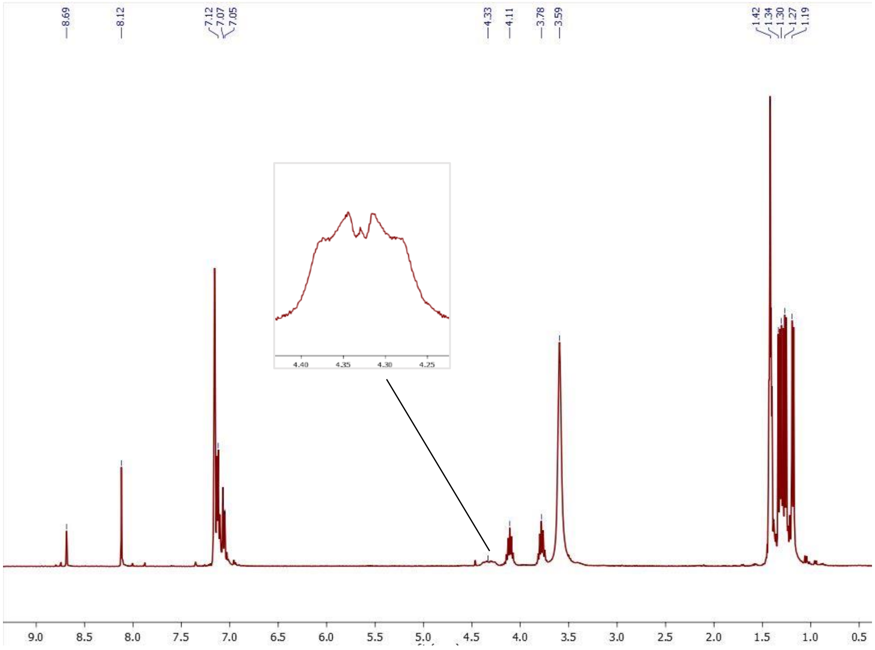

^{1}H NMR (400 MHz, C_6D_6, 25 °C)

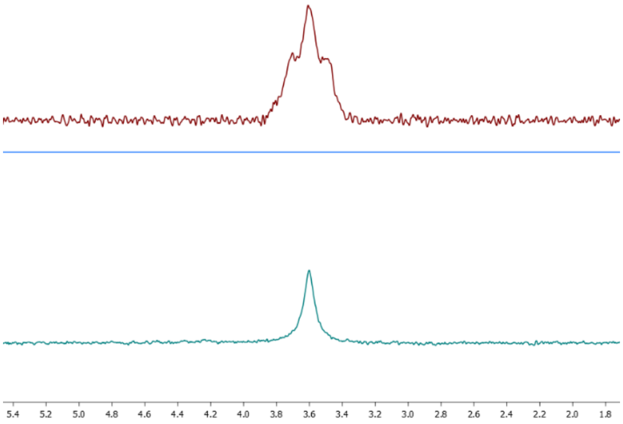

^{7}Li and $^{7}Li\{^{1}H\}$ NMR (161 MHz, C_6D_6, 25 °C)

[$Mo_2\{(H)_2Li(thf)\}(\mu\text{-}O_2CH)(\mu\text{-}Ad^{Dipp2})_2$] (**3·thf**)

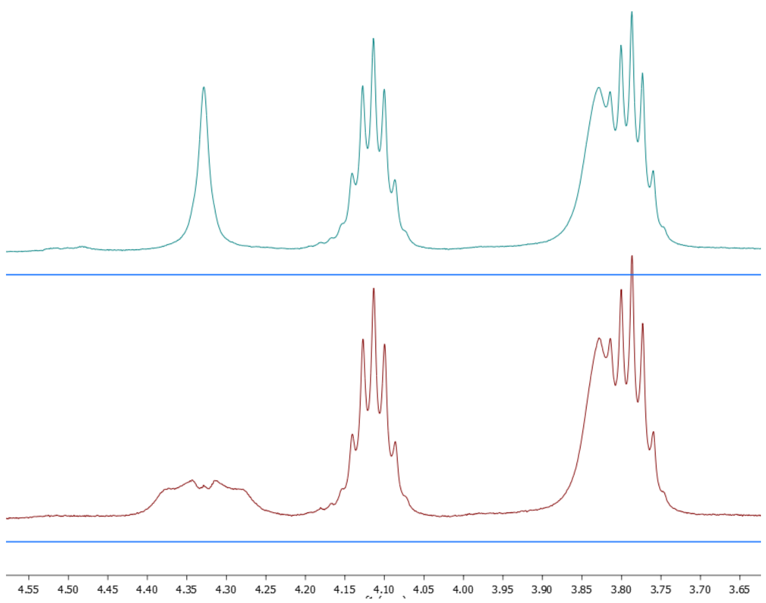

$^{1}H\{^{7}Li\}$ NMR (top) and ^{1}H NMR (bottom) (500 MHz, C_6D_6, 25 °C)

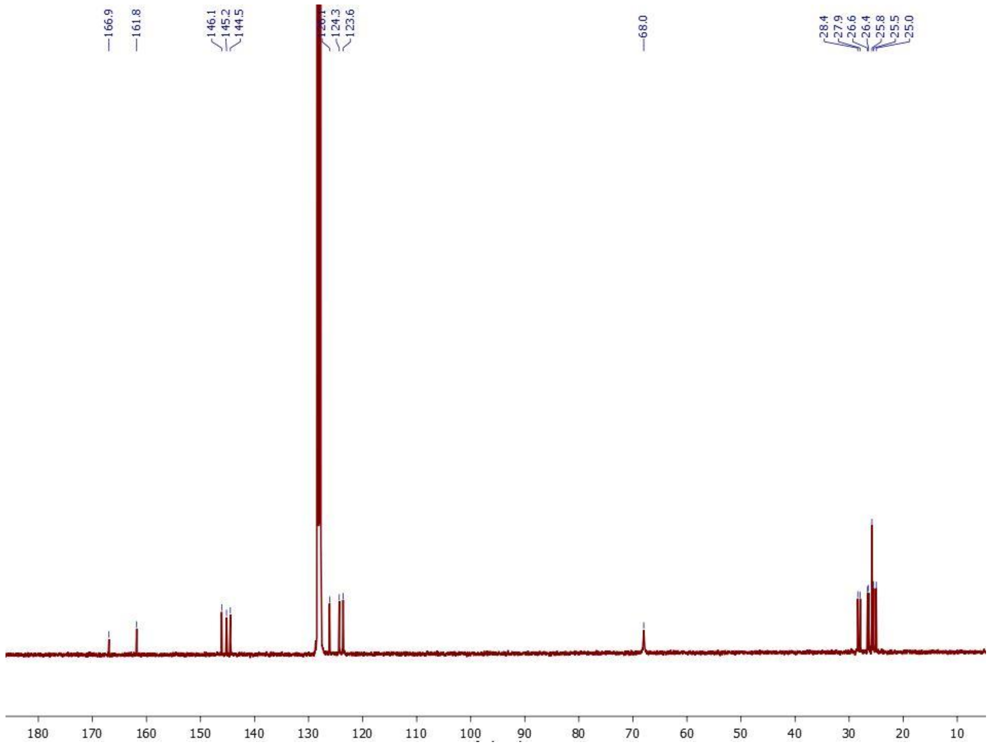

$^{13}C\{^{1}H\}$ NMR (100 MHz, C_6D_6, 25 °C)

[$Mo_2\{(H)_2Li(thf)\}_2(\mu\text{-}Ad^{Dipp2})_2$] (**4·thf**)

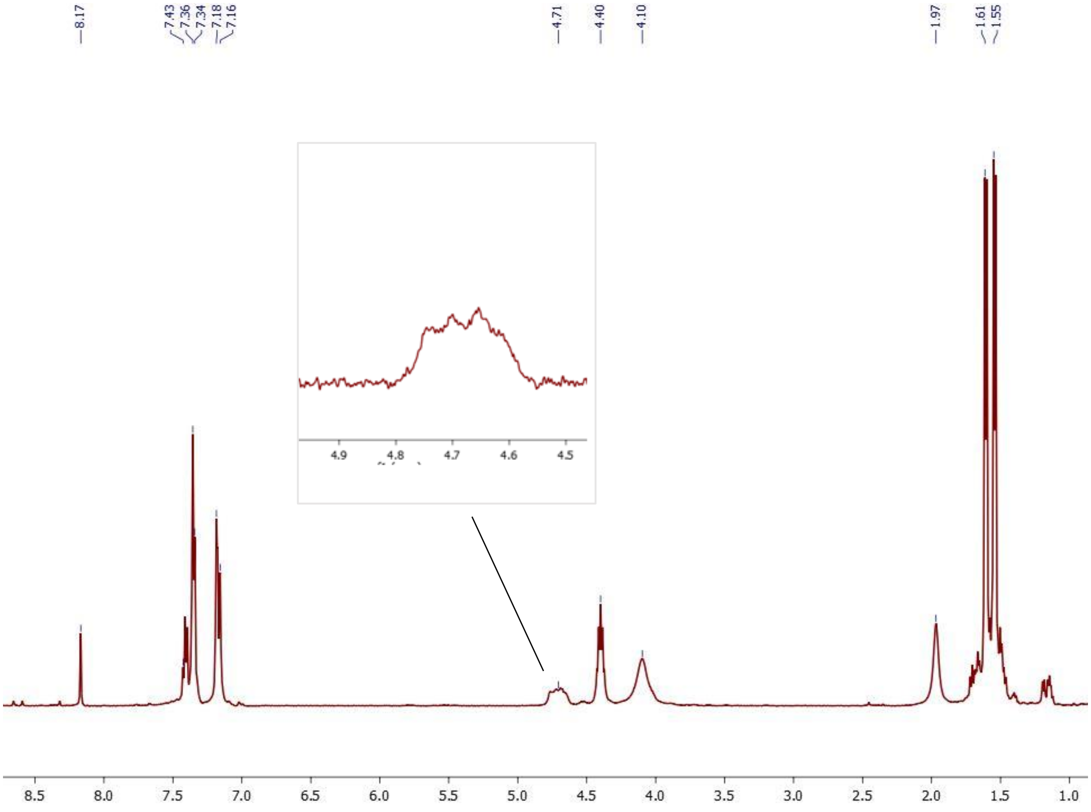

^1H NMR (500 MHz, C_6D_5F, 25 °C)

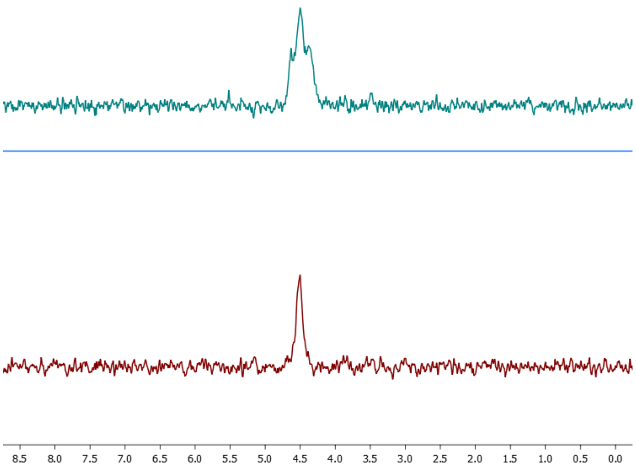

^7Li and $^7Li\{^1H\}$ NMR (161 MHz, C_6D_5F, 25 °C)

$[Mo_2\{(H)_2Li(thf)\}_2(\mu\text{-}Ad^{Dipp2})_2]$ (**4·thf**)

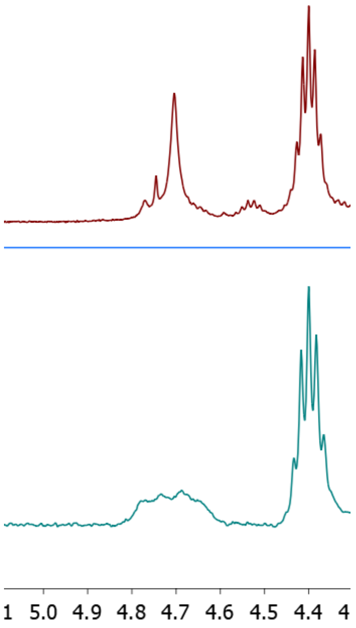

$^{1}H\{^{7}Li\}$ NMR (top) and ^{1}H NMR (bottom) (500 MHz, C_6D_5F, 25 °C)

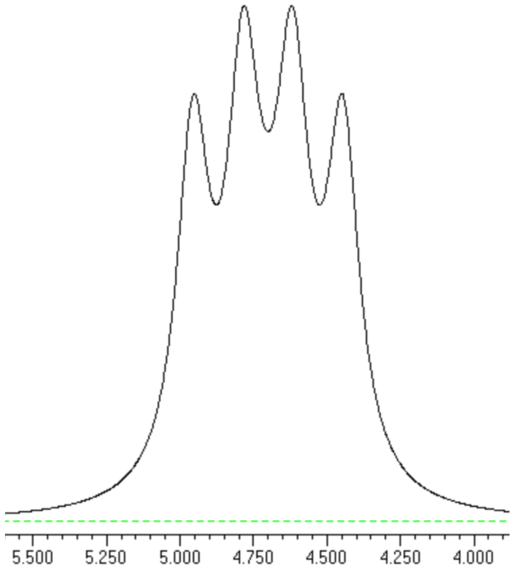

Simulated ^{1}H NMR of the Mo–H signal by using the gNMR program.
The relation of the abundance of 7Li:6Li was considered as 93:7.
The $^{1}J_{HLi}$ value (17 Hz) was obtained experimentally from the ^{7}Li NMR.

[$Mo_2\{(H)_2Li(thf)\}_2(\mu\text{-}Ad^{Dipp2})_2$] (**4·thf**)

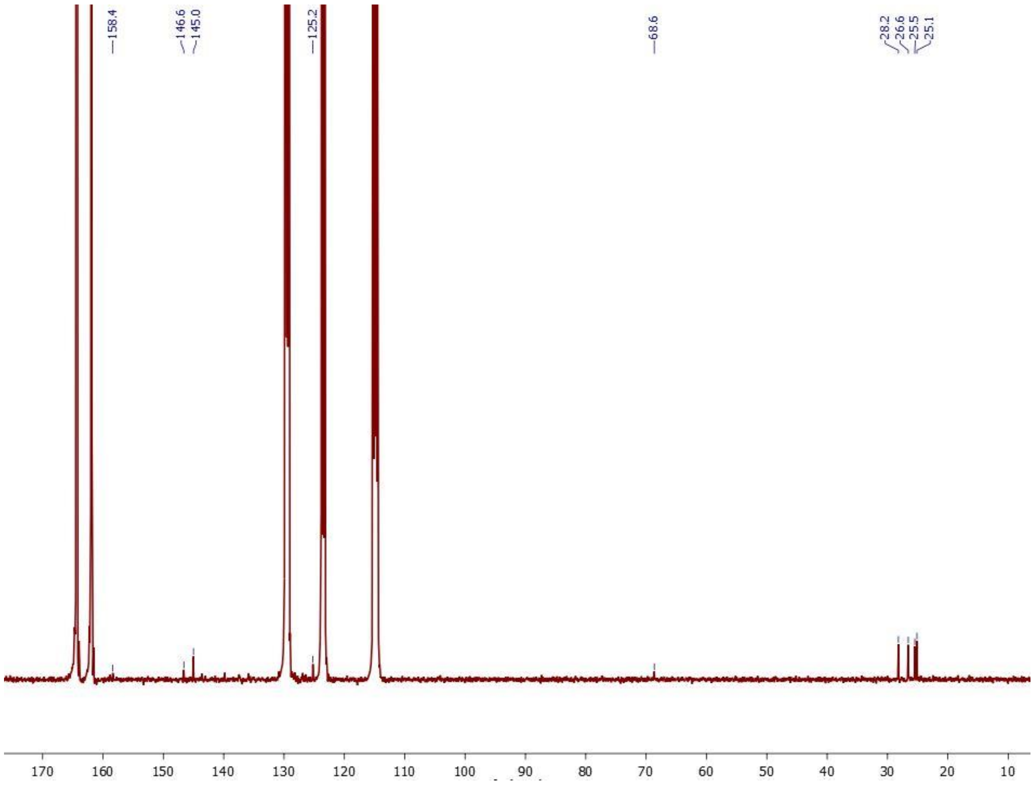

$^{13}C\{^1H\}$ NMR (100 MHz, C_6D_5F, 25 °C)

$[Mo_2(H_2Li)_3(\mu\text{-}Ad^{Dipp2})]_3\cdot(thf)_8$ (**5·thf**)

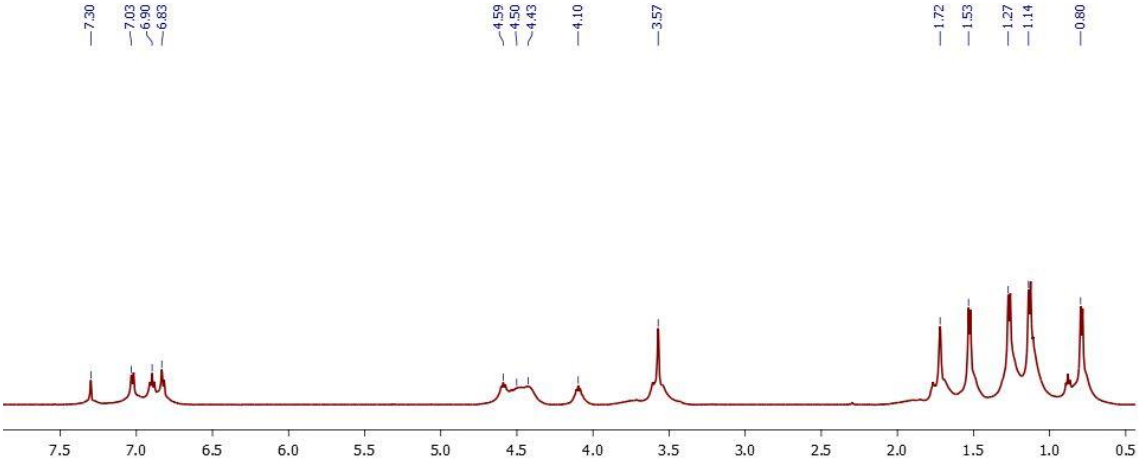

^1H NMR (500 MHz, thf-*d*8, 25 °C)

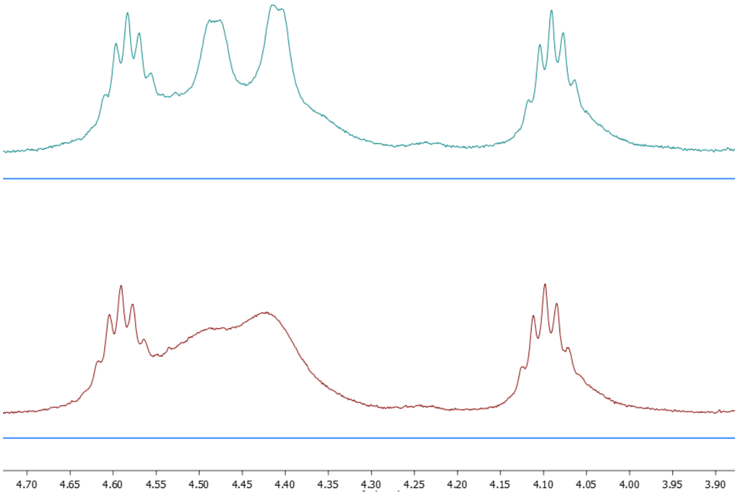

$^1H\{^7Li\}$ NMR (top) and ^1H NMR (bottom), (500 MHz, thf-*d*8, 25 °C)

$[Mo_2(H_2Li)_3(\mu\text{-}Ad^{Dipp2})]_3\cdot(thf)_8$ (**5·thf**)

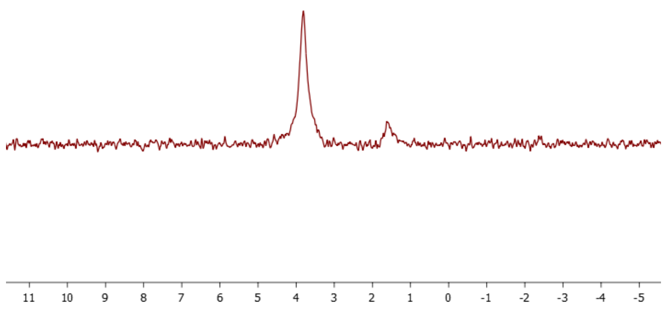

$^{7}Li\{^{1}H\}$ NMR (194 MHz, thf-*d*8, 25 °C)

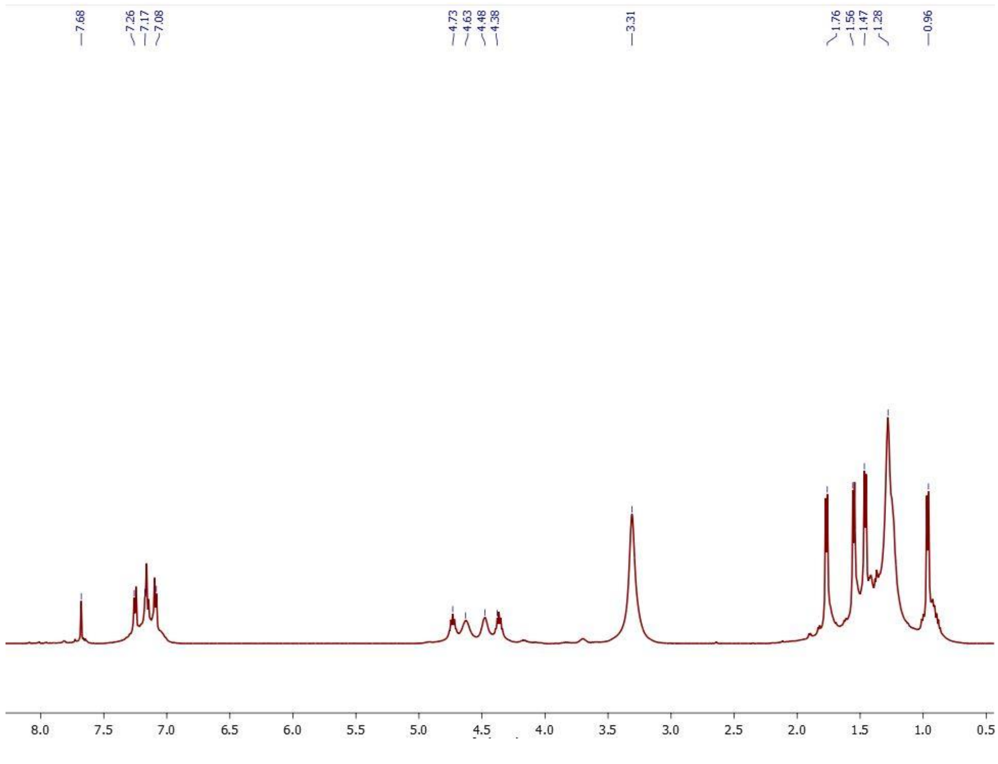

^{1}H NMR (500 MHz, C_6D_6, 25 °C)

$[Mo_2(H_2Li)_3(\mu\text{-}Ad^{Dipp2})]_3\cdot(thf)_8$ (**5·thf**)

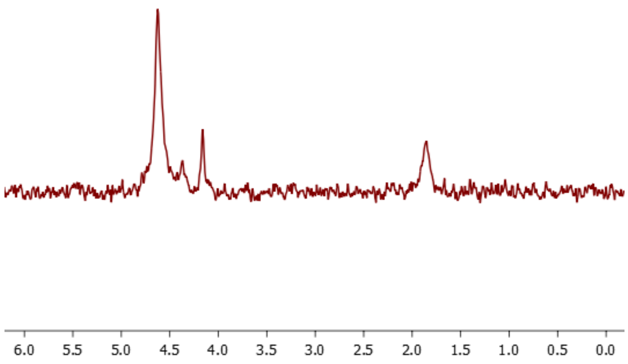

$^{7}Li\{^{1}H\}$ NMR (194 MHz, C_6D_6, 25 °C)

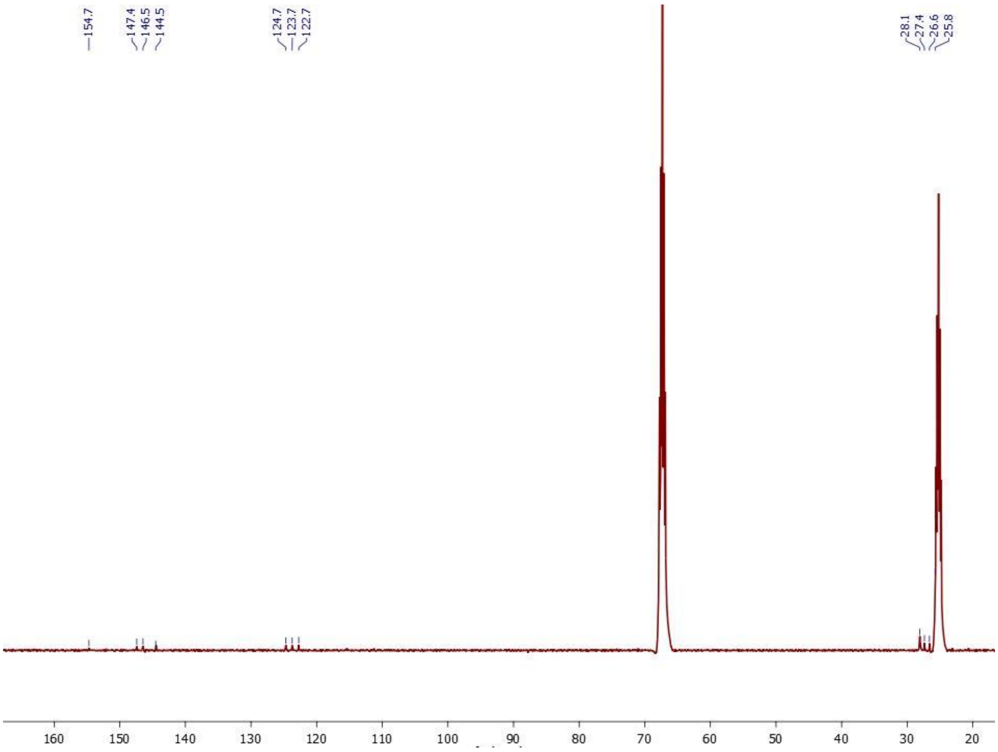

$^{13}C\{^{1}H\}$ NMR (100 MHz, thf-*d*8, 25 °C)

$[Mo_2(H_2Li)_3(\mu\text{-}Ad^{Dipp2})]_3\cdot(py)_8$ (**5·py**)

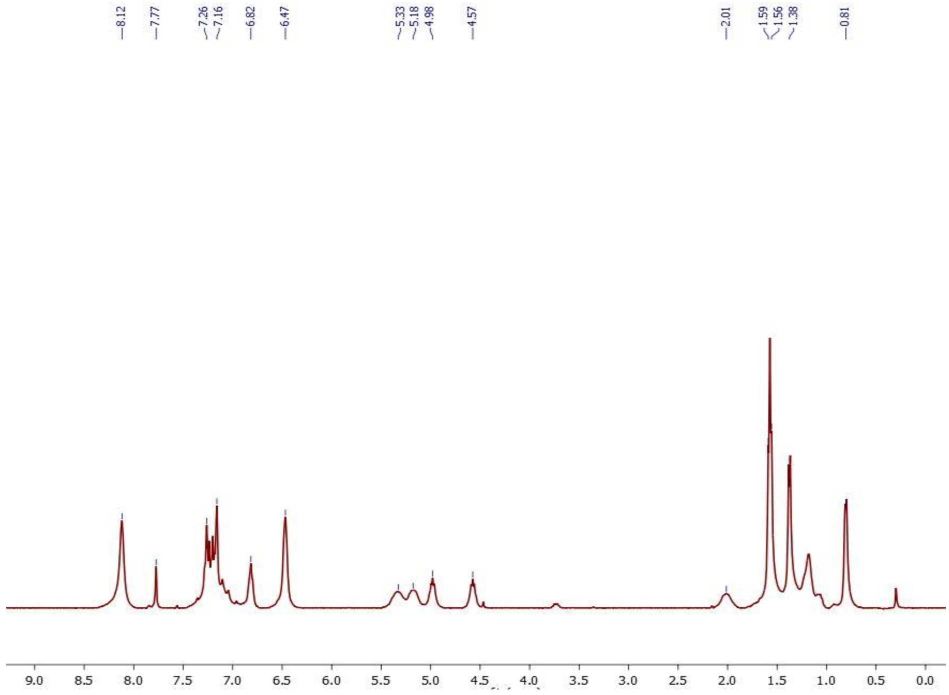

^1H NMR (400 MHz, C_6D_6, 25 °C)

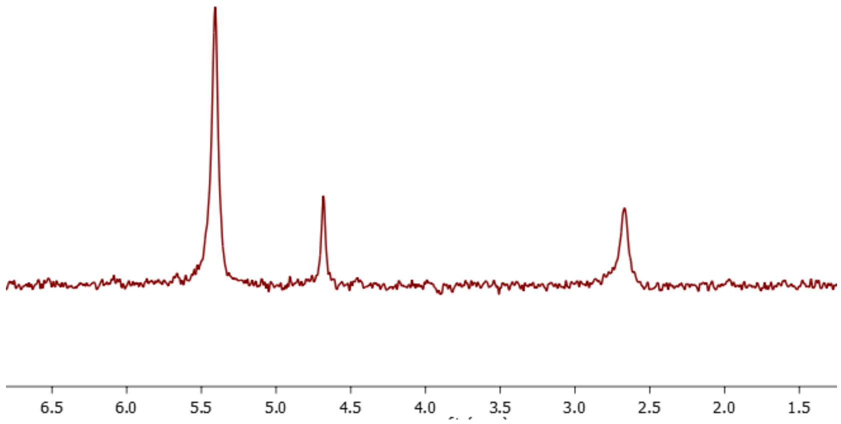

$^7Li\{^1H\}$ NMR (161 MHz, C_6D_6, 25 °C)

$[Mo_2(H_2Li)_3(\mu\text{-}Ad^{Dipp2})]_3\cdot(py)_8$ (**5·py**)

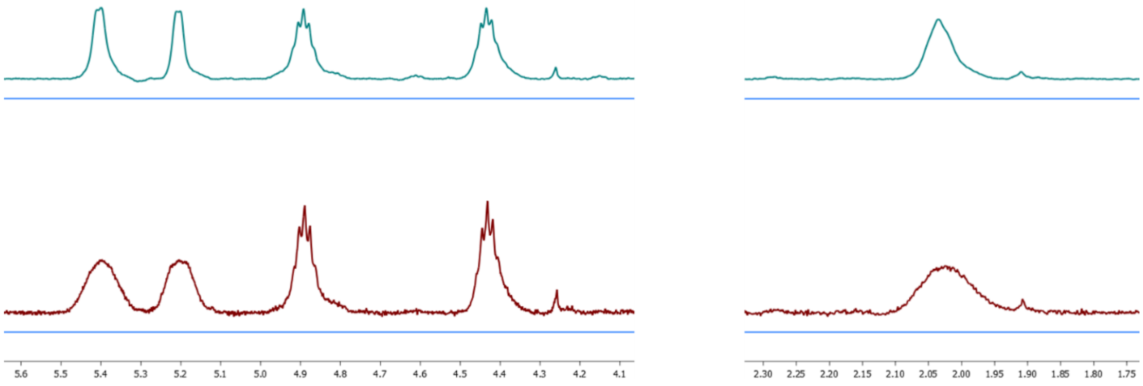

$^{1}H\{^{7}Li\}$ NMR (top) and ^{1}H NMR (bottom), (500 MHz, C_6D_6, 25 °C)

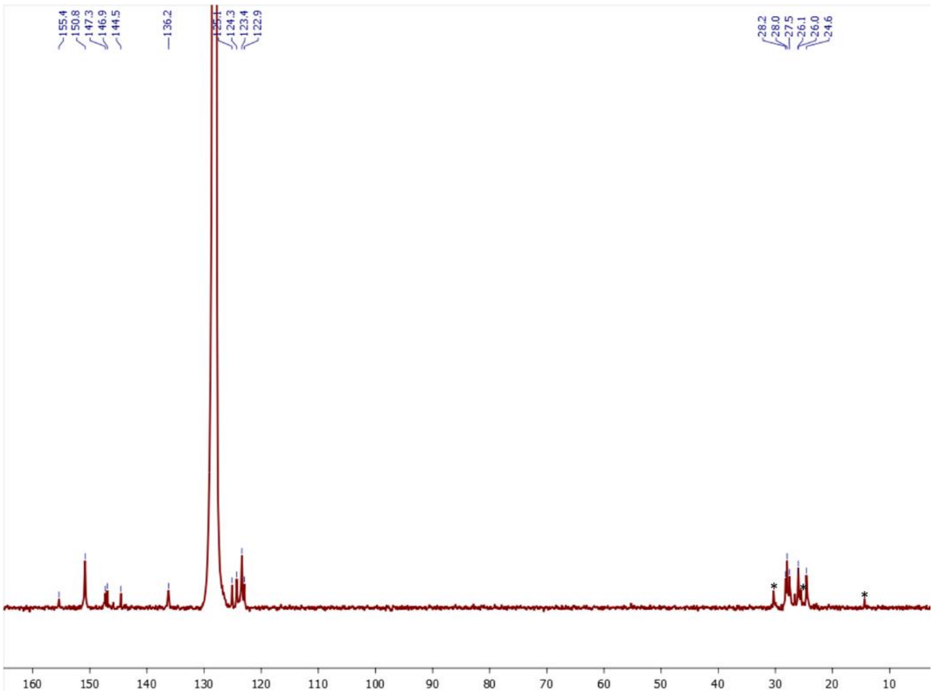

$^{13}C\{^{1}H\}$ NMR (100 MHz, C_6D_6, 25 °C) *pentane

$[Mo_2(H_2Li)_3(\mu\text{-}Ad^{Dipp2})]_3\cdot(dmap)_8$ (**5·dmap**)

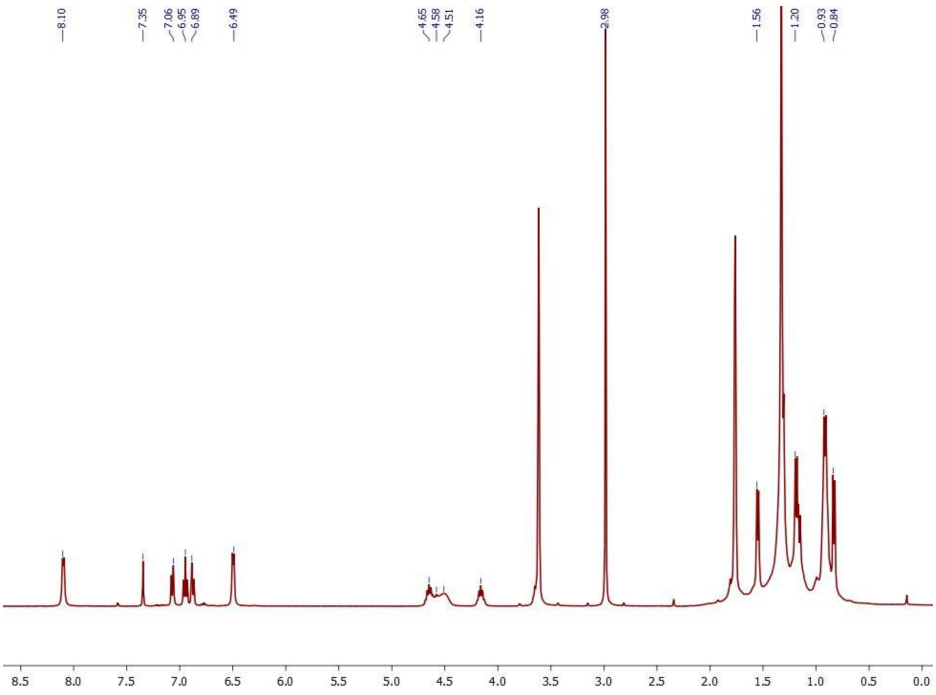

^{1}H NMR (400 MHz, thf-*d*8, 25 °C)

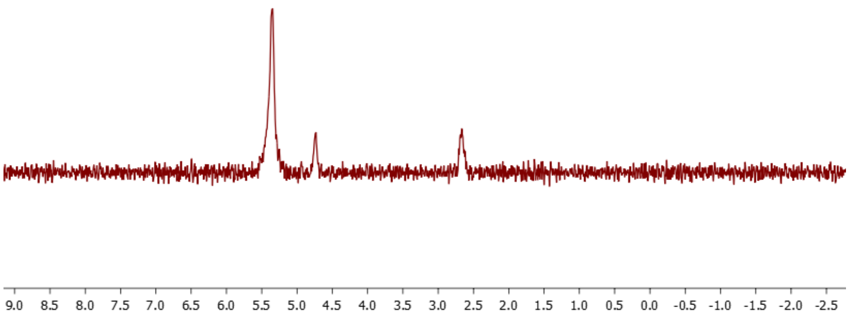

$^{7}Li\{^{1}H\}$ NMR (161 MHz, C_6D_6, 25 °C)

$[Mo_2(H_2Li)_3(\mu\text{-}Ad^{Dipp2})]_3\cdot(dmap)_8$ (**5·dmap**)

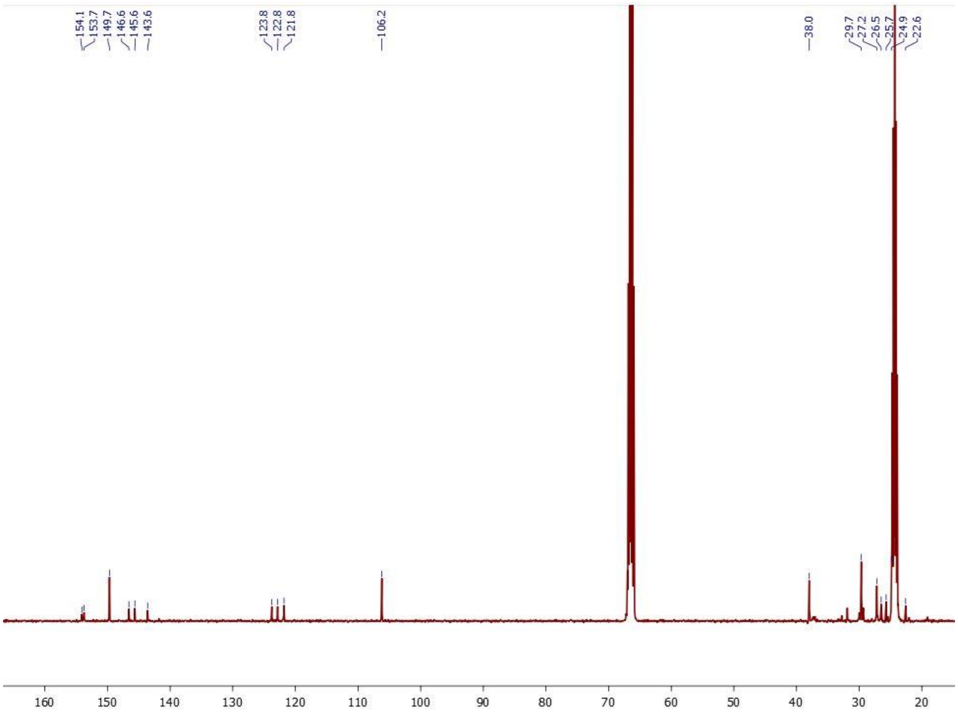

$^{13}C\{^1H\}$ NMR (100 MHz, thf-*d*8, 25 °C)

IR spectra (Nujol):

[Mo_2{(H)$_2$Li(thf)}(μ-O_2CH)(μ-Ad^{Dipp2})$_2$] (**3·thf**)

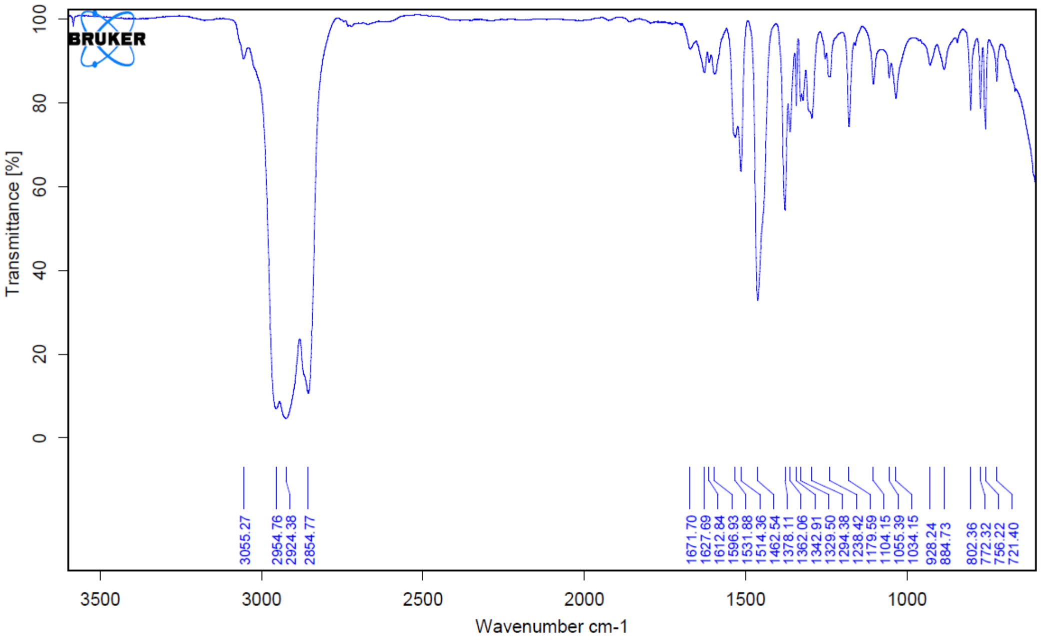

[Mo_2{(D)$_2$Li(thf)}(μ-O_2CH)(μ-Ad^{Dipp2})$_2$]

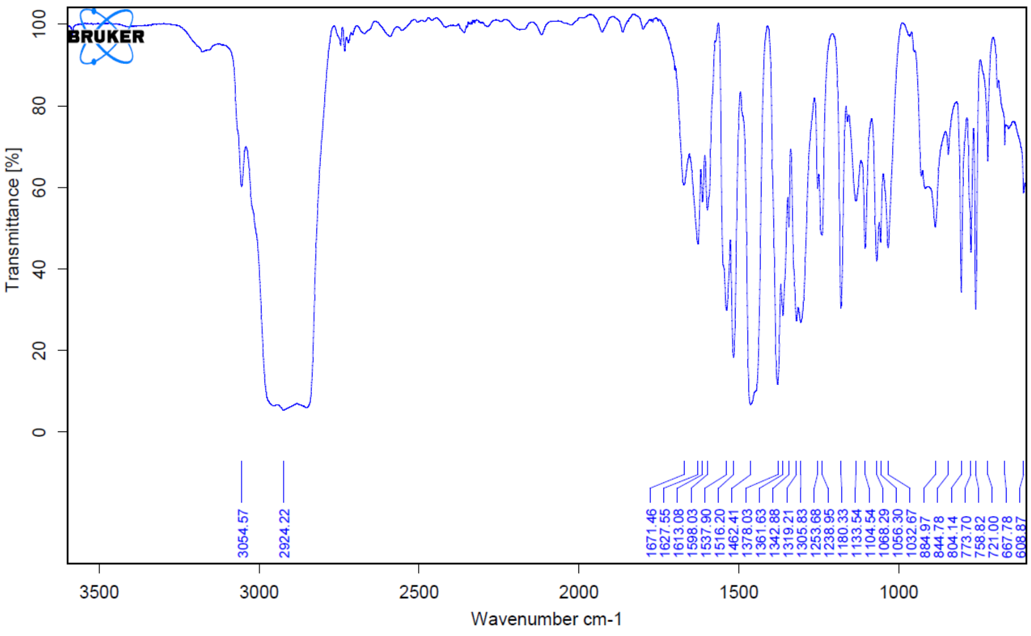

[$Mo_2\{(H)_2Li(thf)\}_2(\mu\text{-}Ad^{Dipp2})_2$] (**4·thf**)

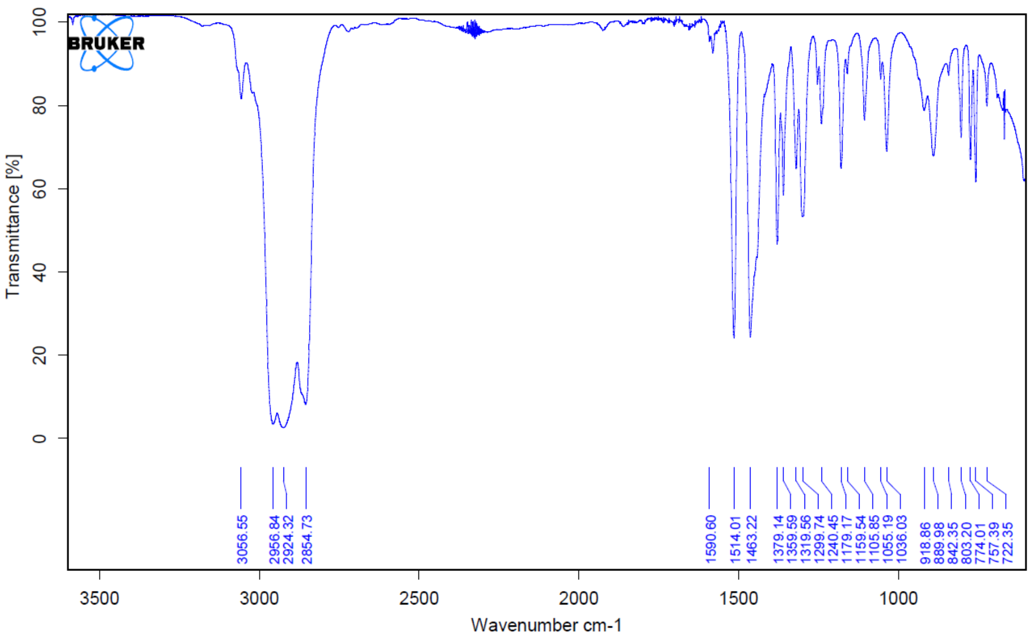

[$Mo_2\{(D)_2Li(thf)\}_2(\mu\text{-}Ad^{Dipp2})_2$]

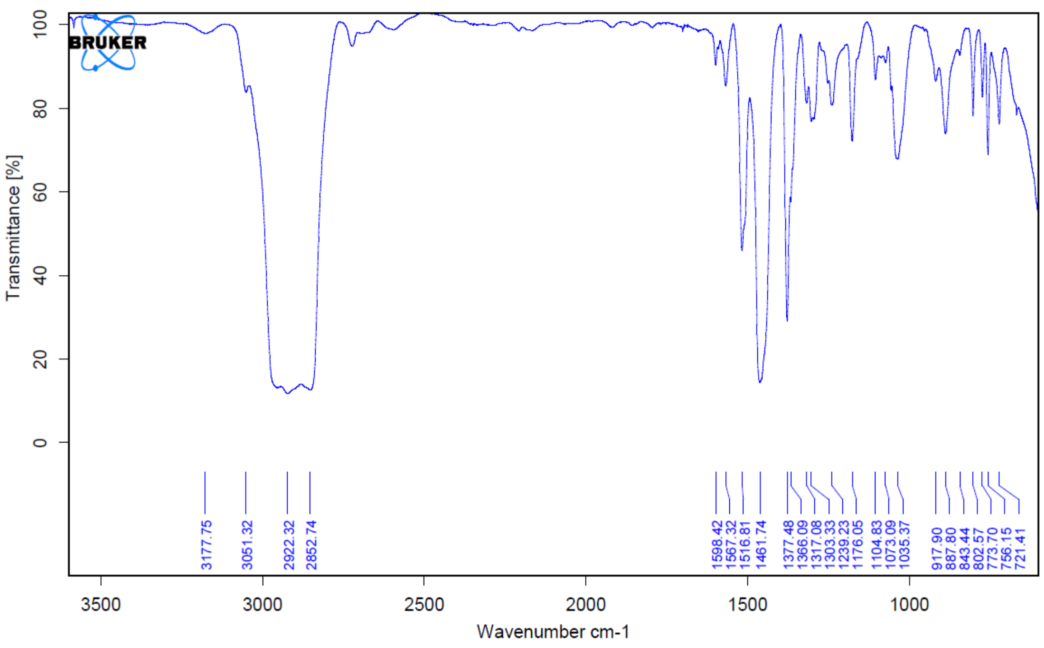

$[Mo_2(LiH_2)_3(\mu\text{-}Ad^{Dipp2})]_3\cdot(thf)_8$ (**5·thf**)

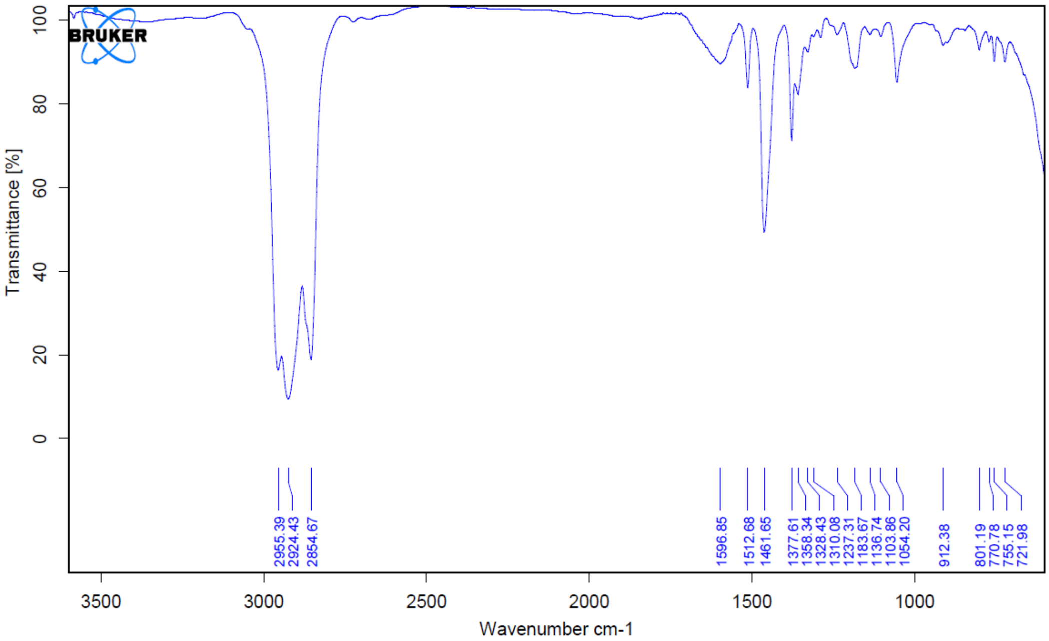

$[Mo_2(H)(\mu\text{-}O_2CH)(\mu\text{-}Ad^{Dipp2})_2(thf)]$ (**2·thf**)

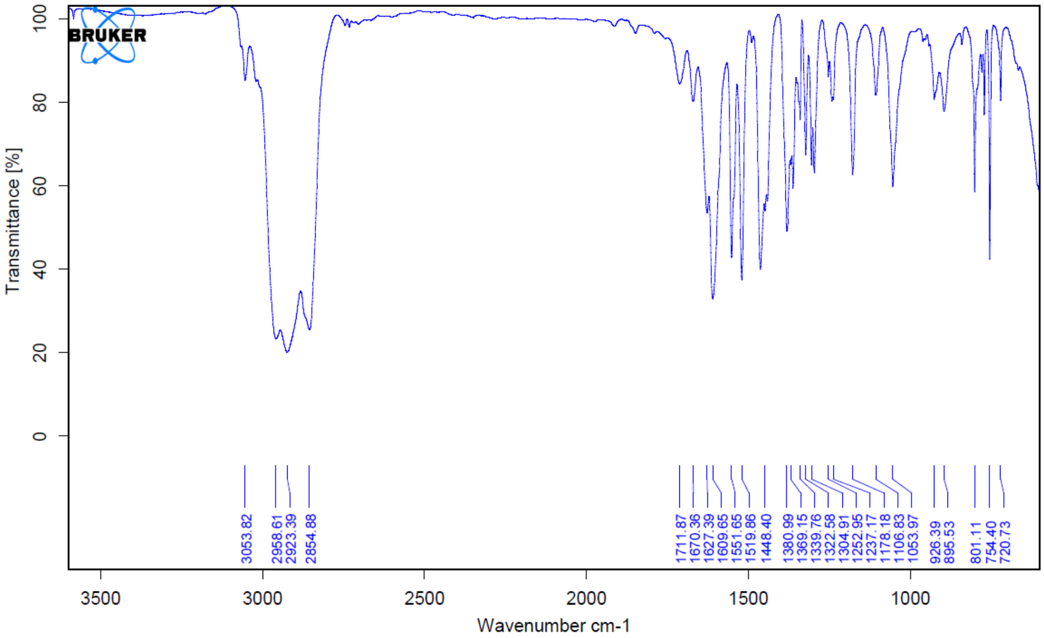

[Mo_2(D)(μ-O_2CH)(μ-Ad^{Dipp2})$_2$(thf)]

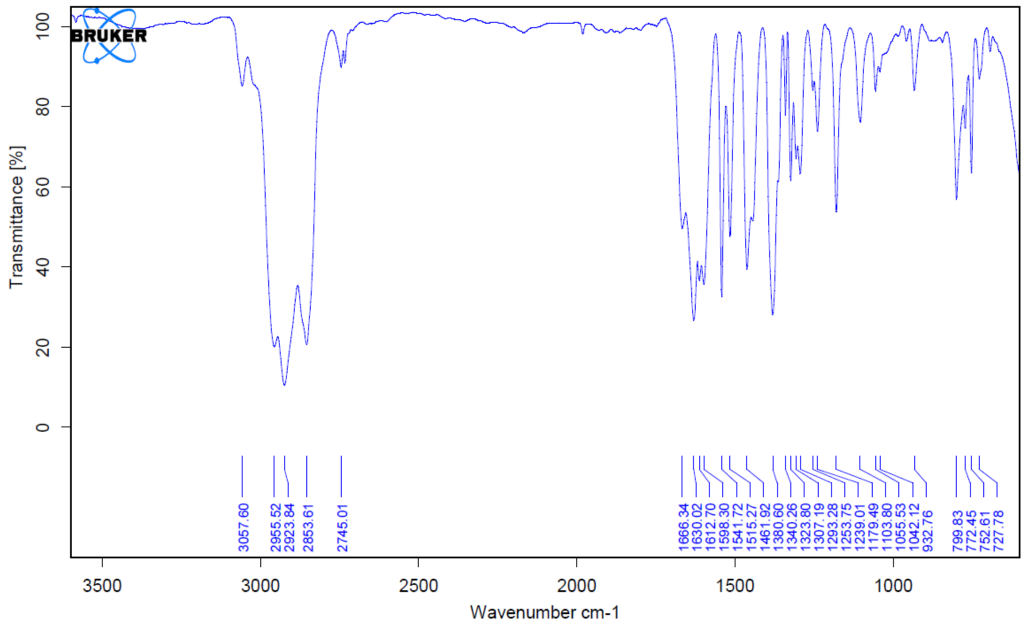

[Mo_2(H)(μ-O_2CH)(μ-Ad^{Dipp2})$_2$(IMe_4)] (**2·IMe_4**)

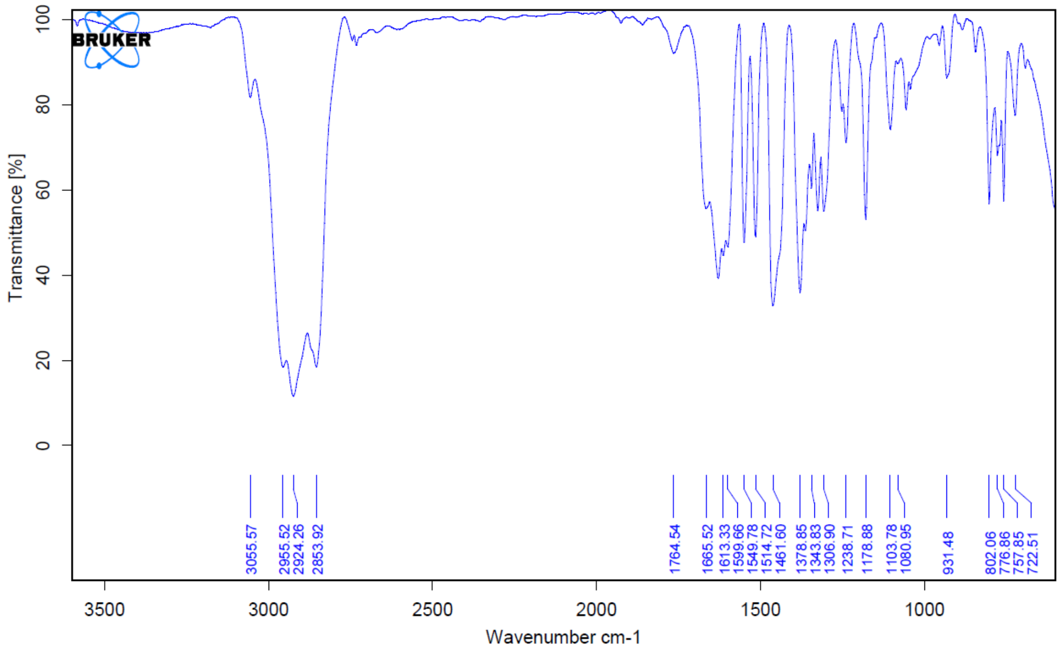

Computational details

All the structures have been fully optimized in gas phase employing the Gaussian09 suite of programs[6] along with the hybrid B3LYP functional.[9-11] In the case of **2-thf** and **3-thf** the all-electron triple-ζ basis set proposed by Schäfer et al.[12,13] was employed to describe the light atoms, whereas an all-electron basis set with a {84211111/641111/51111} contraction was used for the molybdenum atoms.[14] In the case of **4-thf**, we had to slightly modify the methodology due to its large size; thus, the light atoms were described with the standard 6-31G* basis set[15-17] while the Stuttgart basis set (SDD),[18,19] along with the associated ECP for the core electrons, was used for molybdenum. The NBO analyses have been carried out at the same level of theory as above with the NBO 3.1 program included in Gaussian09.[20]

Cartesian coordinates of computed complexes in xyz format

146
3-thf
Mo -1.067241 -0.019570 0.00247500
Mo 1.059226 -0.041426 0.00331200
Li -0.032822 -2.777375 0.00093600
N -1.165949 0.028382 2.18912900
N -1.169740 -0.042371 -2.17766500
N 1.163157 -0.076830 2.18272800
N 1.160419 -0.000343 -2.18127300
O -0.085605 -4.665453 0.00829600
C 0.000787 -0.011097 2.84722400
C 0.850413 -5.561584 -0.72058700
C 1.370954 3.179596 -4.61146800
C 1.186701 -3.251607 4.53238100
C 2.776262 3.196995 3.96199200
C 2.603201 -3.266117 -4.01614600
C 1.817142 -2.777416 3.20375100
C 1.899412 2.686391 -3.24504100
C 2.383952 -0.269579 2.94770300
C 2.390315 0.159856 -2.93920200
C 2.840935 -2.379045 -2.77343400
C 2.924716 2.245850 2.75379000
C 2.720207 -1.563804 3.41876100
C 2.772222 1.442647 -3.41065000
C 3.226299 -0.958661 -3.18014600
C 3.249095 0.822757 3.19829900
C 2.563037 -3.938512 2.51300100
C 2.637306 3.824551 -2.50833600
C 3.889263 -3.016804 -1.83787800
C 3.971392 2.789292 1.75811100
C 3.916727 -1.736939 4.12274800
C 3.991443 1.576640 -4.08523200
C 4.438136 0.593156 3.90097800
C 4.437007 -0.766635 -3.85531100
C 4.777064 -0.672249 4.36161900
C 4.826901 0.488981 -4.30222800
C -0.005673 -0.007827 -2.84171600
C 0.207378 -6.948800 -0.65812100
C -0.557879 -6.917334 0.68152500
C -1.086336 -5.482508 0.74471900
C -1.377122 -3.230505 -4.52222600
C -1.185775 3.188201 4.64576400
C -2.814651 -3.185100 3.97978800
C -2.570729 3.270702 -4.02001900
C -1.757075 2.740556 3.28085800
C -1.958764 -2.712876 -3.18766300
C -2.384013 0.246249 2.95150400
C -2.398708 -0.178664 -2.94227300
C -2.800684 2.366436 -2.78832900
C -2.977382 -2.260647 2.75239900
C -2.692454 1.542091 3.44143600
C -2.802969 -1.457756 -3.40081100
C -3.205901 0.955136 -3.20372900
C -3.279966 -0.826988 3.18151300
C -2.444119 3.924385 2.56696900
C -2.749245 -3.828756 -2.47289400
C -3.827122 3.005337 -1.82914600
C -4.049521 -2.811236 1.78903200
C -3.898047 1.733844 4.12611800
C -4.011745 -1.575949 -4.09548100
C -4.475056 -0.577225 3.86568500
C -4.409332 0.780245 -3.89751800
C -4.791723 0.691394 4.33314400
C -4.817631 -0.470912 -4.34025700
H 0.004772 0.005924 3.93722000
H 0.950320 -7.743906 -0.69560600
H 0.121345 -7.113133 1.51237300
H 0.706463 4.035478 -4.47589100
H 0.615029 -2.453372 5.00772900
H 2.031426 2.827769 4.66836000
H 1.011506 -2.469686 2.53870300
H 0.513898 -4.095513 4.36110600
H 1.042430 2.413502 -2.63521200
H 1.811220 -5.528978 -0.20839200
H 1.908405 -2.313180 -2.21709300
H 1.464827 -1.809963 0.00174300
H 1.844925 -2.835078 -4.67076000
H 0.815201 2.397053 -5.13097200
H 1.973775 2.219626 2.23085700
H 2.269137 -4.264290 -3.71939100
H 2.464592 4.188693 3.62553300
H 2.190636 3.491876 -5.26222400
H 1.873291 -4.763374 2.31751000
H 1.964814 4.673867 -2.36290800
H 1.955206 -3.573973 5.23809900
H 3.571057 -4.016821 -1.53261300
H 3.719520 3.311226 4.50066700
H 3.518184 -3.385201 -4.59999100
H 2.989052 -3.613098 1.56385000
H 3.678142 3.781088 1.40850200
H 4.056126 2.141210 0.88560900
H 2.979891 3.492954 -1.52960400
H 4.023381 -2.416992 -0.93792400
H 3.498185 4.181219 -3.07732000
H 3.372036 -4.325244 3.13514600
H 4.859342 -3.116784 -2.32877100
H 4.958920 2.871708 2.21837700
H 4.178508 -2.720354 4.48991500
H 4.288912 2.551861 -4.44615600
H 5.080129 -1.616613 -4.03966200
H 5.104455 1.423147 4.09337700
H 5.699398 -0.827160 4.90591500
H 5.767310 0.616995 -4.82196600
H -0.007792 0.008904 -3.93180400
H 0.961345 -5.171567 -1.72907700
H -1.158900 -5.083845 1.75330200
H -0.736828 -4.099680 -4.35272600
H -1.362847 -7.649523 0.71986900
H -0.484939 -7.089123 -1.48937600
H -0.660518 2.372618 5.14604700
H -2.044885 -2.812366 4.65650100
H -0.922926 2.431342 2.65694400
H -0.482987 4.013264 4.51210000
H -1.126976 -2.439974 -2.53991400
H -1.862985 2.293930 -2.24659300
H -1.833425 2.838271 -4.69781400
H -0.782712 -2.463412 -5.02027700
H -2.033937 -2.243895 2.21097000
H -2.209616 4.252813 -3.70557800
H -2.533698 -4.194844 3.66753900
H -1.510369 -1.778773 0.00400100
H -2.044103 -5.371735 0.23844500
H -2.173023 -3.529619 -5.20762900
H -1.730523 4.739735 2.42256800
H -2.098025 -4.685276 -2.28107100
H -1.979904 3.529660 5.31332400
H -3.473900 3.984511 -1.49958900
H -3.745384 -3.263518 4.54530400
H -3.494162 3.421193 -4.58359900
H -2.816594 3.622104 1.58941700
H -3.792033 -3.823770 1.46830900
H -4.129753 -2.186713 0.89938800
H -3.141729 -3.474034 -1.51991700
H -3.971618 2.389093 -0.94190300
H -3.587235 -4.180253 -3.07757500
H -3.277978 4.318267 3.15121200
H -4.797423 3.143001 -2.31205500
H -5.032051 -2.855996 2.26322600
H -4.139128 2.718956 4.50188900
H -4.325404 -2.548099 -4.45190000
H -5.031411 1.642057 -4.09742900
H -5.163869 -1.392637 4.04155100
H -5.720607 0.864145 4.86059800
H -5.750555 -0.583520 -4.87684200
O -1.115519 2.192256 -0.00041300
C 0.019294 2.798869 0.00841700
O 1.144092 2.173986 0.01441900
H 0.027968 3.887511 0.01149800

158

4-thf

Mo	-1.066820	0.017396	0.004162
Mo	1.066775	-0.017318	0.003327
Li	-0.048900	-2.771107	0.001691
Li	0.048795	2.771219	0.001526
N	-1.162846	0.053492	2.194086
N	-1.165621	-0.011673	-2.186065
N	1.164626	-0.053559	2.193138
N	1.163823	0.011835	-2.186992
O	-0.101925	-4.668388	0.002937
O	0.101769	4.668501	0.002781
C	0.001163	-0.000026	2.858348
C	-0.206218	6.949525	-0.664448
C	0.565466	6.922486	0.671675
C	0.839445	-5.557369	-0.725279
C	1.101986	5.490206	0.731617
C	1.352478	3.215207	-4.522286
C	1.181667	-3.257241	4.511140
C	2.759568	3.175621	4.031863
C	2.576149	-3.281470	-4.030823
C	1.809197	-2.768200	3.186647
C	1.936416	2.690705	-3.191530
C	2.381162	-0.258890	2.956177
C	2.388248	0.157876	-2.951147
C	2.822323	-2.383030	-2.798268
C	2.939794	2.257429	2.802650
C	2.714469	-1.559171	3.415447
C	2.783934	1.439520	-3.412952
C	3.207846	-0.966723	-3.216818
C	3.253563	0.824455	3.224194
C	2.553265	-3.922073	2.482215
C	2.726715	3.803827	-2.472281
C	3.877863	-3.011540	-1.864422
C	4.016035	2.820056	1.850659
C	3.908331	-1.745586	4.120586
C	3.987747	1.567340	-4.114534
C	4.439790	0.582679	3.927348
C	4.405909	-0.783404	-3.917342
C	4.773130	-0.688671	4.375174
C	4.801681	0.469955	-4.365076
C	-0.001160	0.000062	-2.851542
C	0.205190	-6.949226	-0.665245
C	-0.565610	-6.922534	0.671396
C	-0.840168	5.557529	-0.724650
C	-1.101720	-5.490130	0.732313
C	-1.356483	-3.215522	-4.520964
C	-1.176958	3.256595	4.512144
C	-2.755294	-3.175124	4.034809
C	-2.580126	3.281715	-4.028443
C	-1.806185	2.768105	3.188244
C	-1.939077	-2.690684	-3.189758
C	-2.378692	0.258909	2.958172
C	-2.390672	-0.157812	-2.949195
C	-2.824880	2.383047	-2.795781
C	-2.937421	-2.257426	2.805500
C	-2.711455	1.559213	3.417761
C	-2.786723	-1.439487	-3.410608
C	-3.210598	0.966730	-3.214107
C	-3.250894	-0.824386	3.227049
C	-2.550791	3.922373	2.485039
C	-2.728804	-3.803591	-2.469538
C	-3.879561	3.011207	-1.860724
C	-4.014813	-2.820660	1.855186
C	-3.904660	1.745737	4.123979
C	-3.991159	-1.567377	-4.111109
C	-4.436456	-0.582509	3.931288
C	-4.409278	0.783339	-3.913563
C	-4.769301	0.688884	4.379370
C	-4.805372	-0.470039	-4.360950
H	0.001604	-0.000010	3.948947
H	-0.950457	5.166507	-1.732812
H	1.183651	5.092214	1.739749
H	0.953258	-7.739761	-0.700199
H	0.111753	-7.114344	1.504798
H	0.710737	4.081999	-4.346500
H	1.366332	7.659472	0.706719
H	0.481850	7.094466	-1.498540
H	0.614222	-2.463176	4.998257
H	1.992425	2.790659	4.704466
H	1.005065	-2.450806	2.524739
H	0.505452	-4.096506	4.331023
H	1.108069	2.411381	-2.542428
H	1.799991	-5.519179	-0.212987
H	1.895599	-2.310034	-2.232995
H	1.465136	-1.826866	0.002776
H	1.817396	-2.853838	-4.686954
H	0.758816	2.450022	-5.024090
H	2.002749	2.232783	2.250150
H	2.238161	-4.274705	-3.722176
H	2.465185	4.181943	3.720958
H	1.532204	1.775476	0.005641
H	2.057674	5.384878	0.220031
H	2.147011	3.520654	-5.206822
H	1.862740	-4.745313	2.282657
H	2.075051	4.659478	-2.278227
H	1.951445	-3.591751	5.210140
H	3.562601	-4.009280	-1.548759
H	3.687048	3.266616	4.601293
H	3.488347	-3.412324	-4.616956
H	2.971409	-3.586200	1.533536
H	3.755382	3.833542	1.535490
H	4.101525	2.202327	0.957052
H	3.114932	3.444299	-1.519614
H	4.013399	-2.403951	-0.970118
H	3.565947	4.157174	-3.074557
H	3.365930	-4.313839	3.096756
H	4.845406	-3.113148	-2.360716
H	4.995395	2.865378	2.331978
H	4.164335	-2.734781	4.476288
H	4.291461	2.542633	-4.471171
H	5.034868	-1.639873	-4.120059
H	5.109444	1.407369	4.132169
H	5.693330	-0.853549	4.920305
H	5.730379	0.589753	-4.907552
H	-0.001602	0.000021	-3.942195
H	0.949310	-5.166001	-1.733352
H	-1.182427	-5.092515	1.740672
H	-0.954507	7.739885	-0.698593
H	-0.111394	7.113760	1.505608
H	-0.714528	-4.082238	-4.345600
H	-1.366648	-7.659322	0.706667
H	-0.483455	-7.093650	-1.498951
H	-0.608997	2.462293	4.998273
H	-1.987201	-2.789812	4.706125
H	-1.002908	2.450705	2.525298
H	-0.500871	4.095849	4.331497
H	-1.110079	-2.411332	-2.541496
H	-1.800437	5.518918	-0.211879
H	-1.897555	2.310125	-2.231487
H	-1.465180	1.826934	0.004302
H	-1.822001	2.854297	-4.685445
H	-0.763371	-2.450452	-5.023592
H	-2.001137	-2.232752	2.251708
H	-2.241951	4.274944	-3.719984
H	-2.461269	-4.181540	3.723870
H	-1.532377	-1.775371	0.006881
H	-2.057810	-5.384353	0.221579
H	-2.151708	-3.521196	-5.204599
H	-1.860295	4.745502	2.284924
H	-2.077029	-4.659257	-2.275907
H	-1.945839	3.590931	5.212211
H	-3.564182	4.008970	-1.545253
H	-3.681926	-3.265964	4.605642
H	-3.492950	3.412561	-4.613602
H	-2.970131	3.586894	1.536746
H	-3.754370	-3.834220	1.540087
H	-4.101634	-2.203319	0.961437
H	-3.116107	-3.443821	-1.516590
H	-4.013991	2.403456	-0.966363
H	-3.568617	-4.156980	-3.070975
H	-3.362639	4.314169	3.100647
H	-4.847659	3.112656	-2.355968
H	-4.993521	-2.866016	2.337819
H	-4.160262	2.734952	4.479906
H	-4.295163	-2.542700	-4.467421
H	-5.038487	1.639763	-4.115692
H	-5.105951	-1.407153	4.136816
H	-5.688982	0.853833	4.925356
H	-5.734547	-0.589901	-4.902594

143

4·thf′

Mo	-1.047501	0.267805	0.011438
Mo	1.052919	0.231454	-0.001883
Li	0.044162	2.929008	0.009380
N	-1.145285	0.163780	2.165470
N	-1.178259	0.092804	-2.144701
N	1.177364	0.041721	2.154393
N	1.147431	0.124152	-2.157256
O	0.102046	4.810218	0.018395
C	0.015501	0.031370	2.824113
C	-0.300102	7.077973	-0.623439
C	0.766895	7.067991	0.491446
C	1.259127	5.617337	0.491971
C	1.284164	3.065135	-4.837482
C	1.034484	-3.255872	4.255518
C	2.865420	3.078226	4.177228
C	2.700507	-3.404304	-3.446350
C	1.710825	-2.754150	2.958995
C	1.876849	2.670413	-3.466794
C	2.392952	-0.261266	2.887317
C	2.368495	0.191621	-2.936975
C	2.861958	-2.283887	-2.395390
C	3.025450	2.240195	2.889191
C	2.673779	-1.602873	3.252594
C	2.732835	1.408648	-3.564742
C	3.203743	-0.945109	-3.045099
C	3.302351	0.773674	3.212055
C	2.398238	-3.929154	2.232103
C	2.663658	3.848487	-2.853345
C	3.916685	-2.684630	-1.341232
C	4.112787	2.842415	1.974766
C	3.868148	-1.876305	3.929010
C	3.922653	1.457266	-4.298865
C	4.484693	0.443133	3.884055
C	4.386563	-0.840719	-3.787096
C	4.772656	-0.868434	4.241027
C	4.748435	0.345187	-4.413137
C	-0.018293	0.037934	-2.815244
C	-0.974248	5.714448	-0.462305
C	-1.184985	-3.201741	-4.269065
C	-1.143845	3.089826	4.870406
C	-2.864973	-3.282536	3.430890
C	-2.736212	3.200953	-4.151976
C	-1.744970	2.734072	3.492700
C	-1.825805	-2.675778	-2.964200
C	-2.363551	0.288093	2.942041
C	-2.406690	-0.157462	-2.876146
C	-2.935746	2.367629	-2.866596
C	-2.983317	-2.154816	2.381827
C	-2.666751	1.518274	3.577201
C	-2.743343	-1.485008	-3.245834
C	-3.274547	0.914977	-3.193410
C	-3.258646	-0.803251	3.036981
C	-2.463456	3.955316	2.879381
C	-2.553560	-3.822745	-2.231835
C	-3.997286	3.014190	-1.952236
C	-4.061961	-2.506334	1.334474
C	-3.856200	1.624407	4.305622
C	-3.951098	-1.706965	-3.917200
C	-4.438585	-0.641651	3.773407
C	-4.472456	0.635407	-3.861014
C	-4.740684	0.557052	4.406696
C	-4.815344	-0.662031	-4.221171
H	0.013226	-0.089185	3.906764
H	-1.362946	5.297330	-1.387559
H	1.539432	5.248487	1.475460
H	0.635697	3.938728	-4.737655
H	1.576155	7.771386	0.301917
H	0.172491	7.158246	-1.603462
H	0.490467	-2.455894	4.760218
H	2.085217	2.669380	4.820368
H	0.935279	-2.381841	2.291282
H	0.328913	-4.058142	4.030590
H	1.048809	2.454978	-2.793241
H	1.907613	-2.173964	-1.881151
H	-0.032015	-1.503705	0.002801
H	1.961211	-3.134940	-4.200998
H	0.694260	2.252087	-5.263302
H	2.085905	2.279315	2.341733
H	2.375051	-4.329060	-2.964476
H	2.597809	4.109708	3.931594
H	1.599211	1.980529	-0.008712
H	2.080618	5.455745	-0.204060
H	2.071827	3.312957	-5.552343
H	1.654757	-4.679156	1.953813
H	2.008298	4.715730	-2.735753
H	1.775673	-3.646150	4.956754
H	3.640634	-3.630507	-0.871819
H	3.792519	3.106114	4.753915
H	3.642947	-3.609945	-3.958738
H	2.894367	-3.590745	1.323810
H	3.872100	3.879870	1.728468
H	4.187845	2.285221	1.041013
H	3.058029	3.577513	-1.873574
H	4.005686	-1.929329	-0.558244
H	3.500209	4.150043	-3.486832
H	3.142728	-4.419405	2.862824
H	4.902615	-2.812445	-1.793122
H	5.092506	2.836378	2.456942
H	4.092081	-2.894765	4.215970
H	4.207536	2.379966	-4.786782
H	5.030175	-1.705325	-3.878918
H	5.186451	1.226915	4.137270
H	5.691455	-1.102688	4.762510
H	5.664620	0.402426	-4.986074
H	-0.021643	-0.082918	-3.897882
H	-1.008022	7.898573	-0.517548
H	0.314452	7.320603	1.451485
H	-0.511342	-4.033611	-4.053955
H	-0.596613	2.246179	5.293932
H	-2.106643	-3.048846	4.178510
H	-0.923390	2.478286	2.825387
H	-0.453547	3.931963	4.781843
H	-1.028690	-2.337390	-2.304080
H	-1.763129	5.731245	0.289317
H	-1.996931	2.364239	-2.316614
H	-1.535575	2.034874	0.025164
H	-1.970680	2.761770	-4.792802
H	-0.613276	-2.423679	-4.777512
H	-2.028211	-2.087733	1.861529
H	-2.427592	4.220117	-3.903021
H	-2.588285	-4.221538	2.946099
H	-1.949184	-3.558568	-4.963402
H	-1.762934	4.788108	2.773566
H	-1.840666	-4.607449	-1.969739
H	-1.923738	3.369785	5.581858
H	-3.711592	4.039340	-1.702080
H	-3.811187	-3.443027	3.952362
H	-3.658916	3.267850	-4.732408
H	-2.861747	3.711913	1.894017
H	-3.831645	-3.462854	0.861878
H	-4.122459	-1.746651	0.553003
H	-3.018386	-3.467288	-1.313510
H	-4.097763	2.457834	-1.020401
H	-3.330173	-4.274485	-2.852328
H	-3.289052	4.295423	3.507541
H	-4.975605	3.053137	-2.435714
H	-5.049666	-2.591459	1.792438
H	-4.094478	2.557560	4.798745
H	-4.217465	-2.714440	-4.206756
H	-5.143037	1.447912	-4.107780
H	-5.127580	-1.471556	3.854615
H	-5.655901	0.658784	4.974961
H	-5.745442	-0.856759	-4.738849

323
5-thf

Mo -3.095596 0.178546 1.026264
Mo -2.903269 1.243864 -0.828128
Mo 2.437583 1.808018 -1.045459
Mo 1.682697 2.601451 0.798181
Mo 1.424094 -2.739226 0.885366
Mo 0.259398 -3.074416 -0.884626
Li -2.496777 -1.780923 -1.214112
Li -1.592789 -2.478247 1.446115
Li -1.360082 2.700063 1.328842
Li -0.101000 -0.081519 -2.443629
Li -0.403428 2.973238 -1.384408
Li -0.052534 0.019531 0.013060
Li 3.055704 -0.146341 1.191808
Li 2.674955 -1.239311 -1.434721
Li 0.133014 0.065455 2.444571
O -3.149415 -2.950958 -2.694102
O -2.612565 -3.287226 2.983572
O -1.433229 4.088142 2.769709
O -1.033208 4.193611 -2.850811
O -0.170074 -0.000259 -4.361087
O 4.425866 -0.632159 2.579198
O 4.018673 -1.394641 -2.927309
O 0.194544 0.034603 4.358436
N -5.219114 0.563253 1.124562
N -4.977544 1.835729 -0.789650
N 3.996237 3.299943 -1.144236
N 3.097424 4.242650 0.769797
N 2.128474 -4.779994 0.910519
N 0.756301 -5.176210 -0.911011
C -7.845041 -1.517059 3.742817
C -8.161645 -1.491515 -0.623410
C -7.711087 -1.911822 2.414947
C -7.131157 -0.413166 4.197305
C -6.563157 2.659954 4.221774
C -7.375979 0.639002 -3.698073
C -6.867248 -1.231339 1.527903
C -6.526595 4.940912 1.108154
C -6.804672 -1.705860 0.077190
C -6.484390 4.926021 -3.282915
C -6.272316 0.303916 3.352450
C -6.405181 3.620517 -3.757448
C -6.130610 -0.113899 2.005023
C -6.101596 5.198674 -1.972335
C -6.367002 -3.178115 -0.040131
C -5.559641 1.532235 3.908118
C -5.752413 1.353010 0.186329
C -5.935175 2.574060 -2.951042
C -5.623821 4.191683 -1.124538
C -5.935061 1.155625 -3.511637
C -5.528647 2.865356 -1.624788
C -5.262467 4.551255 0.315237
C -4.725656 1.192808 5.157746
C -5.159122 1.054182 -4.837911
C -4.360529 -3.448564 -4.645983
C -4.206736 5.669374 0.401769
C -4.191631 -3.694584 4.692930
C -3.332208 -2.492534 -4.046269
C -4.010695 -3.090599 3.302433
C -3.953910 -4.784268 -4.000294
C -3.218182 -4.882493 4.644677
C -3.472800 -4.362814 -2.602002
C -2.745112 -6.616281 0.655357
C -2.114599 3.884434 4.028295
C -2.386877 4.658067 -3.074262
C -2.346416 5.368559 -4.424679
C -2.048407 -4.314079 3.832402
C -2.135072 5.251829 4.718188
C -1.595236 -7.929429 -1.855367
C -1.249458 -6.902562 0.416200
C -1.343501 -8.064077 -3.217442
C -0.946248 1.019900 -5.029772
C -0.784649 0.713918 -6.516142
C -0.946577 6.001508 -4.400036
C -0.912800 -6.989994 -1.071760
C -0.817264 -8.183290 1.158835
C -0.953271 -0.330649 5.159482
C -0.857271 5.906787 4.171809
C -0.385096 -7.250927 -3.813549
C -0.842576 5.409759 2.727077
C -0.482361 -0.194221 6.605794
C -0.112107 4.927029 -3.692831
C 7.512628 3.439358 0.668062
C 7.476487 2.686321 -2.373342
C 7.360157 2.999154 -3.724681
C 7.140969 0.989031 0.140802
C 6.591711 2.418841 -0.031269
C 6.142258 -0.371484 4.229347
C 6.385440 2.783146 -1.500457
C 6.132099 3.426717 -4.218402
C 5.864816 -4.087695 -0.622595
C 5.587080 -5.831284 1.870949
C 5.467141 4.370791 4.042538
C 5.438512 -6.094320 3.229732
C 5.768259 -1.854884 4.064221
C 5.042832 0.360117 3.434926
C 5.382749 -2.431249 -4.597398
C 5.764666 -0.950183 -4.438038
C 5.135572 3.225334 -2.013330
C 5.069558 -6.433801 -1.157553
C 5.010465 3.552396 -3.387182
C 4.751415 -5.129483 -0.398695
C 5.170326 -1.866087 2.661508
C 4.510211 -5.402962 1.084762
C 5.254166 -0.651833 -3.032341
C 4.188594 -5.934370 3.818914
C 4.012368 4.135264 3.589637
C 3.863466 5.490458 -4.550192
C 4.008457 4.255584 -0.209128
C 4.055075 -2.529946 -3.826807
C 3.706859 4.062027 -3.991950
C 3.421650 5.383871 2.943102
C 3.147460 3.649179 4.768661
C 3.303891 6.546396 3.717526
C 3.235624 -5.247907 1.694228
C 3.074820 -5.517275 3.076716
C 3.180609 3.105013 -5.078484
C 3.014077 5.420490 1.585791
C 2.805211 7.730078 3.184037
C 2.968212 7.639344 -1.235007
C 2.541308 -6.370579 -4.314671
C 2.492753 6.623030 1.034728
C 2.407303 7.758193 1.850759
C 1.728763 -5.399298 3.783027
C 1.777259 -4.357660 4.917425
C 2.027189 6.732096 -0.416464
C 1.251529 -6.764803 4.316141
C 1.627463 -5.622567 0.000861
C 1.401810 -5.476831 -3.786181
C 1.411683 -0.071052 5.140024
C 0.801207 -4.629820 -4.924244
C 0.987832 -0.634894 6.501613
C 0.853489 -0.514036 -5.252097
C 0.572379 7.230736 -0.522480
C 0.334025 -6.300872 -3.074835
C 0.677097 0.238021 -6.580039
C 0.067208 -6.165925 -1.688930
H -8.951659 -2.093275 -0.157145
H -8.510475 -2.056072 4.412808
H -8.474834 -0.442647 -0.575462
H -8.281448 -2.763810 2.053456
H -7.149881 2.927125 3.337336
H -7.937593 0.680210 -2.759791
H -8.101140 -1.779803 -1.679494
H -7.264837 2.362774 5.011239
H -7.252136 -0.089303 5.227885
H -7.273760 4.139672 1.087099
H -7.921049 1.235793 -4.440271
H -6.996298 5.841349 0.693019
H -6.857076 5.721657 -3.923540
H -7.371017 -0.402294 -4.044941
H -6.816966 1.603649 0.219302
H -6.038369 3.561076 4.564683
H -7.053485 -3.856237 0.481976
H -6.729083 3.403513 -4.772116
H -6.278395 5.146433 2.156326
H -6.183217 6.215093 -1.595233
H -6.346141 -3.478823 -1.095064
H -6.050487 -1.112957 -0.445497
H -5.371687 -3.157734 -4.336306
H -5.354008 0.846835 5.987598
H -4.868877 1.888690 3.138850
H -5.620622 1.655431 -5.630969
H -5.228287 -3.983812 4.888363
H -5.428733 0.512955 -2.786041
H -5.361257 -3.316312 0.368586
H -4.329911 -3.472072 -5.740221
H -4.549125 6.600771 -0.066363
H -4.820066 3.667945 0.782284
H -3.647190 -1.449296 -3.992063
H -4.624240 -3.608620 2.556905
H -3.996483 0.406505 4.936556
H -4.178058 2.076516 5.507951
H -5.135120 0.014725 -5.188018
H -3.678817 -5.727212 4.118413
H -4.774210 -5.506938 -3.952670
H -3.983872 5.892123 1.452819
H -4.125941 1.395743 -4.714719
H -4.255849 -4.467738 -1.844560
H -4.224524 -2.022155 3.243873
H -3.886578 -2.977246 5.465218
H -3.387485 -7.404309 0.243672
H -3.274135 5.356844 -0.077666
H -3.103831 3.474976 3.816259
H -3.033396 -5.661816 0.204777
H -3.155154 6.096786 -4.534640
H -2.950831 -6.556359 1.731599
H -3.252595 -1.581813 0.530511
H -2.373614 -2.549481 -4.583474
H -2.905238 -5.232559 5.633547
H -3.054299 -0.097363 -2.041998
H -2.666664 5.345250 -2.268479
H -2.556468 1.433375 2.223299
H -3.052788 3.794318 -3.038327
H -3.133993 -5.246021 -4.561358
H -2.150946 5.167556 5.809544
H -1.548908 3.145653 4.610741

H -2.338548 -8.569319 -1.386707
H -3.018381 5.824233 4.409519
H -1.968566 0.943420 -4.655226
H -2.236123 2.885356 -0.355471
H -2.427890 4.642789 -5.243554
H -2.585062 -4.906860 -2.273177
H -1.468526 -0.088766 -6.816759
H -1.883187 -8.801556 -3.806395
H -1.550867 -5.052907 3.199903
H -1.353359 -9.064078 0.784194
H -1.239712 -1.362082 4.918099
H -0.980120 1.585021 -7.149184
H -0.960929 6.927013 -3.811886
H -1.436956 -3.325652 -0.252175
H -1.420891 -0.467347 1.438791
H -1.292586 -3.846569 4.473143
H -0.541430 2.005102 -4.767748
H -1.068247 -0.807716 7.297144
H -0.696717 -6.060416 0.839608
H -1.142421 1.090373 -1.341949
H -1.027721 -8.097312 2.232017
H -1.769346 0.338464 4.881260
H -0.556795 6.241239 -5.394460
H -0.531496 -1.470151 -1.272208
H 0.025448 5.540137 4.708391
H -0.863635 6.998974 4.236096
H -1.458214 6.044398 2.076710
H -0.175205 -7.365885 -4.874123
H -0.111015 6.550636 -0.005342
H 1.056937 -1.728483 6.502095
H 0.255197 -8.373083 1.038107
H 0.044079 -3.938937 -4.536743
H 0.265883 7.279985 -1.574838
H 8.515521 3.443182 0.223334
H 8.438304 2.361404 -1.984849
H 8.220903 2.918659 -4.383997
H 8.113587 0.857325 -0.348939
H 7.621017 3.197568 1.732508
H 7.113388 4.456729 0.590834
H 7.127093 -0.180147 3.787567
H 7.279477 0.763541 1.205709
H 6.826555 -4.413123 -0.207539
H 6.180191 -0.049350 5.274702
H 6.561777 -5.963118 1.407986
H 6.287217 -6.428484 3.821449
H 6.622815 -2.530737 4.163349
H 5.897755 3.447781 4.451996
H 5.994532 -6.894534 -0.789250
H 6.095529 4.695862 3.206692
H 6.837579 -0.764049 -4.543407
H 6.442138 0.254000 -0.270247
H 6.145004 -3.068221 -4.133650
H 6.041677 3.684932 -5.270638
H 6.013337 -3.919180 -1.696549
H 5.529187 5.138232 4.824009
H 5.434936 1.171156 2.814753
H 5.594844 -3.130208 -0.166700
H 5.616849 2.442435 0.462642
H 5.957043 -1.009495 -2.268211
H 5.956065 -1.871053 1.893537
H 5.278653 -2.743256 -5.641355
H 5.010238 -2.153128 4.798385
H 5.198809 -6.237039 -2.228930
H 4.762897 5.046599 -0.244911
H 4.210969 6.182712 -3.775990
H 4.582653 5.527874 -5.377886
H 4.256766 0.769431 4.074434
H 4.265373 -7.169121 -1.043038
H 5.236731 -0.328630 -5.171269
H 4.067228 -6.151408 4.877268
H 4.010335 3.339967 2.840057
H 3.963922 -3.448565 -3.241237
H 5.030943 0.397758 -2.840430
H 3.896430 2.992781 -5.902661
H 3.064841 4.410131 5.554989
H 3.626204 6.524329 4.755585
H 3.835353 -4.703612 -0.815606
H 4.478581 -2.684651 2.462221
H 4.001446 7.276437 -1.203941
H 3.588693 2.752926 5.223440
H 3.550197 0.435238 -0.560600
H 3.331460 -5.758814 -4.769037
H 2.903205 5.861900 -4.931005
H 2.981984 2.113927 -4.656210
H 3.183816 -2.456117 -4.484240
H 2.960130 4.085475 -3.194559
H 2.997853 -2.015006 0.279305
H 2.990981 -6.958049 -3.507270
H 2.732928 8.623894 3.798759
H 2.516792 1.543186 2.021700
H 2.653529 7.676667 -2.285229
H 2.033907 -3.368140 4.523295
H 2.966791 8.666668 -0.849875
H 2.137138 3.393775 4.430453
H 2.514921 -4.625123 5.684559
H 1.927277 -7.158802 5.085353
H 1.839922 0.932854 5.221369
H 2.242349 3.484459 -5.502951
H 2.186269 -7.072220 -5.079827
H 1.824291 -4.781706 -3.056397
H 1.928599 -6.674225 0.002038
H 2.048852 5.730600 -0.854466
H 1.194062 -7.506029 3.512078
H 2.022295 8.683990 1.431047
H 1.583886 -4.035144 -5.412174
H 1.831536 -0.349793 -4.790028
H 1.294523 2.535831 -2.247630
H 2.112508 -0.714550 4.600435
H 1.381470 -2.476429 -2.192866
H 1.610842 -0.255309 7.317244
H 1.404208 0.339470 -1.417592
H 1.000601 -5.043300 3.050105
H 1.203815 -0.968516 1.310793
H 0.799588 -4.281062 5.410018
H 1.352636 1.099388 -6.625693
H 0.154175 5.320469 2.293881
H 0.333539 -5.255323 -5.695142
H 0.254729 -6.675356 4.767132
H 0.685783 5.331848 -3.065978
H -0.550629 0.850205 6.933307
H 0.884334 -0.399775 -7.444756
H 0.333842 4.221128 -4.402061
H 0.238563 3.613255 0.300105
H 0.446685 8.235253 -0.100181
H 0.156113 -2.896142 2.171940
H 0.290224 1.510351 1.257151
H 0.686298 -1.590367 -5.354144

References

1. Casanova, D.; Cirera, J.; Llunell, M.; Alemany, P.; D. Avnir, D.; Alvarez, S. Minimal Distortion Pathways in Polyhedral Rearrangements**.** *J. Am. Chem. Soc.* **2004**, *126*, 1755-1763.
2. Alvarez, S. Distortion Pathways of Transition Metal Coordination Polyhedra Induced by Chelating Topology. *Chem. Rev*. **2015**, *115*, 13447-13483.
3. Sheldrick, G. M. SADABS, *Program for Empirical Absorption Correction of Area Detector Data*. Göttingen, University of Göttingen, **1996**.
4. Sheldrick, G. M. SHELXTL, version 6.14. *Program for solution and refinement of crystal structures*, Universität Göttingen, Germany, **2000**.
5. Spek, A. L. *Acta Crystallogr*. **2015**, C71, 9-18.
6. Perez-Jimenez, M.; Curado, N.; Maya, C.; Campos, J.; Ruiz, E., Álvarez, S.; Carmona, E. Experimental and Computational Studies on Quadruply Bonded Dimolybdenum Complexes with Terminal and Bridging Hydride Ligands. *Chem. Eur. J*. **2021**. doi: 10.1002/chem.202004948.
7. Mendoza, I.; Curado, N.; Carrasco, M.; Alvarez, E.; Peloso, R.; Rodriguez, A.; Carmona, E. Synthesis and structure of mixed carboxylate-aminopyridinate and –amidinate complexes of dimolybdenum and ditungsten. *Inorg. Chim. Acta*. **2015**, *424*, 120-128.
8. Frisch, M. J.; Trucks, G. W.; Schlegel, H. B.; Scuseria, G. E.; Robb, M. A.; Cheeseman, J. R.; Scalmani, G.; Barone, V.; Mennucci, B.; Petersson, G. A.; Nakatsuji, H.; Caricato, M.; Li, X.; Hratchian, H. P.; Izmaylov, A. F.; Bloino, J.; Zheng, G.; Sonnenberg, J. L.; Hada, M.; Ehara, M.; Toyota, K.; Fukuda, R.; Hasegawa, J.; Ishida, M.; Nakajima, T.; Honda, Y.; Kitao, O.; Nakai, H.; Vreven, T.; Montgomery, J. A. Jr.; Peralta, J. E.; Ogliaro, F.; Bearpark, M.; Heyd, J. J.; Brothers, E.; Kudin, K. N.; Staroverov, V. N.; Keith, T.; Kobayashi, R.; Normand, J.; Raghavachari, K.; Rendell, A.; Burant, J. C.; Iyengar, S. S.; Tomasi, J.; Cossi, M.; Rega, N.; Millam, J. M.; Klene, M.; Knox, J. E.; Cross, J. B.; Bakken, V.; Adamo, C.; Jaramillo, J.; Gomperts, R.; Stratmann, R. E.; Yazyev, O.; Austin, A. J.; Cammi, R.; Pomelli, C.; Ochterski, J. W.; Martin, R. L.; Morokuma, K.; Zakrzewski, V. G.; Voth, G. A.; Salvador, P.; Dannenberg, J. J.; Dapprich, S.; Daniels, A. D.; Farkas, O.; Foresman, J. B.; Ortiz, J. V.; Cioslowski, J.; Fox, D. J. Gaussian 09, Revision D.01, Gaussian, Inc., Wallingford CT, **2013**.
9. Lee, C.; Yang W.; Parr, R. G. *Phys. Rev. B*, **1988**, 785-789.
10. Miehlich, B.; Savin, A.; Stoll, H.; Preuss, H. *Chem. Phys. Lett.* **1989**, 200-206.
11. Becke, A. D. *J. Chem. Phys.* **1993**, 5648-5652.
12. Schaefer, A.; Horn, H.; Ahlrichs, R. *J. Chem. Phys.* **1992**, *97*, 2571-2577.
13. Schaefer, A.; Huber, C.; Ahlrichs, R. *J. Chem. Phys.* **1994**, *100*, 5829-5835.
14. Ahlrichs, R.; May, K. *Phys. Chem. Chem. Phys.* **2000**, *2*, 943-945.
15. Ditchfield, R.; Hehre, W. J.; Pople, J. A. *J. Chem. Phys.* **1971**, *54*, 724-728.
16. Hehre, W. J.; Ditchfield, R.; Pople, J. A. *J. Chem. Phys.* **1972**, *56*, 2257-2261.
17. Hariharan, P. C.; Pople, J. A. *Theor. Chem. Acc.* **1973**, *28*, 213-222.
18. Dunning, T. H.; Hay, P. J. *Modern Theoretical Chemistry* (Schaefer, H. F., III, Ed.; Plenum: New York, 3, 1-28, **1976**).
19. Bergner, A.; Dolg, M.; Küchle, W.; Stoll, H.; Preuss, H. *Mol. Phys.* **1993**, *80*, 1431-1441.
20. Glendening, E. D.; Reed, A. E.; Carpenter, J. E.; Weinhold, F. *NBO*, Version 3.1.
